# Inhibition of FLT3-ITD Kinase in Acute Myeloid Leukemia by New Imidazo[1,2-*b*]pyridazine Derivatives Identified by Scaffold Hopping

**DOI:** 10.1021/acs.jmedchem.3c00575

**Published:** 2023-08-03

**Authors:** Petra Břehová, Eva Řezníčková, Kryštof Škach, Radek Jorda, Milan Dejmek, Veronika Vojáčková, Michal Šála, Markéta Kovalová, Martin Dračínský, Alexandra Dolníková, Timotej Strmeň, Monika Kinnertová, Karel Chalupský, Alexandra Dvořáková, Tomáš Gucký, Helena Mertlíková Kaiserová, Pavel Klener, Radim Nencka, Vladimír Kryštof

**Affiliations:** † Institute of Organic Chemistry and Biochemistry of the Czech Academy of Sciences, Flemingovo nám. 2, 16000 Prague, Czech Republic; ‡ Department of Experimental Biology, Faculty of Science, Palacký University Olomouc, Šlechtitelů 27, 78371 Olomouc, Czech Republic; § Institute of Pathological Physiology, First Faculty of Medicine, Charles University, 12108 Prague, Czech Republic; ∥ Institute of Molecular and Translational Medicine, Faculty of Medicine and Dentistry, Palacký University Olomouc, Hněvotínská 5, 77900 Olomouc, Czech Republic

## Abstract

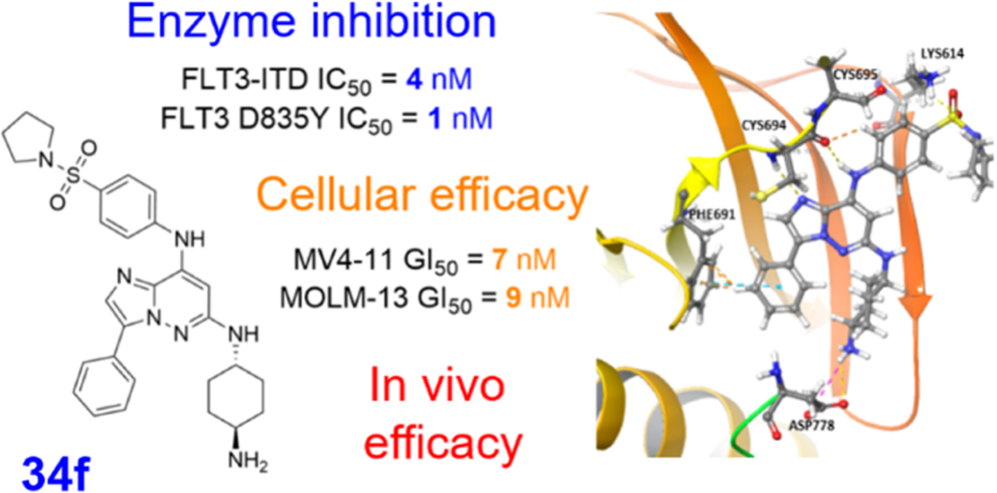

FLT3 kinase is a potential drug target in acute myeloid leukemia (AML). Patients with FLT3 mutations typically have higher relapse rates and worse outcomes than patients without FLT3 mutations. In this study, we investigated the suitability of various heterocycles as central cores of FLT3 inhibitors, including thieno[3,2-*d*]pyrimidine, pyrazolo[1,5-*a*]pyrimidine, imidazo[4,5-*b*]pyridine, pyrido[4,3-*d*]pyrimidine, and imidazo[1,2-*b*]pyridazine. Our assays revealed a series of imidazo[1,2-*b*]pyridazines with high potency against FLT3. Compound **34f** showed nanomolar inhibitory activity against recombinant FLT3-ITD and FLT3-D835Y (IC_50_ values 4 and 1 nM, respectively) as well as in the FLT3-ITD-positive AML cell lines MV4-11, MOLM-13, and MOLM-13 expressing the FLT3-ITD-D835Y mutant (GI_50_ values of 7, 9, and 4 nM, respectively). In contrast, FLT3-independent cell lines were much less sensitive. In vitro experiments confirmed suppression of FLT3 downstream signaling pathways. Finally, the treatment of MV4-11 xenograft-bearing mice with **34f** at doses of 5 and 10 mg/kg markedly blocked tumor growth without any adverse effects.

## Introduction

Acute myeloid leukemia (AML) is a malignant clonal disorder of the hematopoietic system characterized by infiltration of the bone marrow, peripheral blood, and other tissues by abnormally differentiated blasts of myeloid lineage.^1,2^ Although AML can occur at any age, it is the most common acute type of leukemia in adults and increases in incidence with age. The five-year overall survival of patients diagnosed with AML is estimated to be less than 50%.^3^ Despite the growing number of drugs available for the treatment of AML, the need for efficient therapeutic strategies persists. Advances in molecular cancer biology have resulted in the identification of potential therapeutic targets for the treatment of AML. Mutations of the FMS-like tyrosine kinase 3 (FLT3) gene occur in approximately 30% of AML cases. Patients with FLT3 mutations have higher relapse rates and worse outcomes for both overall survival and disease-free survival in comparison with patients without FLT3 mutations.

FLT3 is a membrane-bound cytokine receptor closely related to KIT, FMS, and PDGFR. Binding to an extracellular ligand results in receptor dimerization and autophosphorylation of tyrosine residues in the intracellular domain, which activates downstream signaling pathways, including RAS/MAPK, JAK/STAT5, and PI3K/AKT/mTOR. These pathways promote the growth, proliferation, survival, and differentiation of myeloid cells.^4,5^

Internal tandem duplication (ITD), which represents the most common group of FLT3 mutations, occurs in 20–25% of all AML patients. ITD promotes ligand-independent dimerization and downstream signaling.^6,7^ Point mutations in the tyrosine kinase domain (FLT3-TKD) are approximately twice less prevalent. TKD mutations stabilize the kinase in its active conformation. Both FLT3-ITD and FLT3-TKD mutations can cause ligand-independent FLT3 kinase activation and promote cell proliferation, resulting in a high leukemic burden.

As mutated FLT3 is considered an attractive target for the treatment of AML, several small-molecule inhibitors have been investigated as potential therapeutics.^8−10^ First-generation inhibitors comprise nonspecific receptor tyrosine kinase inhibitors, such as sunitinib, sorafenib, and midostaurin, originally developed for other indications. A second generation of more selective and efficient inhibitors, which exhibit lower toxicity and off-target effects, has also been developed. These inhibitors, which include quizartinib, crenolanib, and gilteritinib, produce significant responses in AML patients.

**Figure 1:**
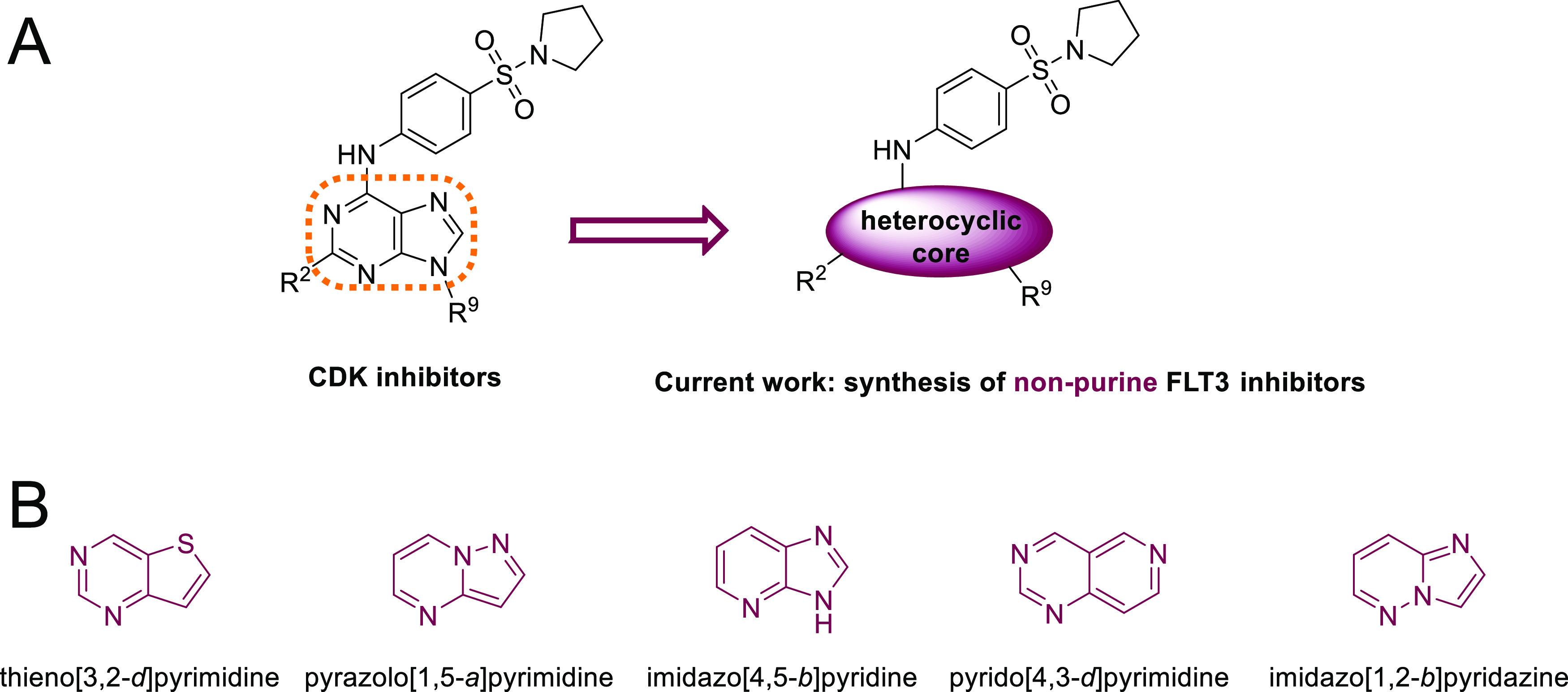
Structural modifications of kinase inhibitors leading to (A) FLT3 inhibitors and (B) heterocyclic cores explored in this study.

In our previous studies, we investigated trisubstituted purines as kinase inhibitors and carbocyclic nucleoside derivatives with CDK2 inhibitory activity, among other analogues.^11^ We revealed that some of these compounds display nanomolar inhibitory potency toward FLT3 kinase (unpublished observation, Supporting Information, Table S1). These findings are substantiated in another of our studies, which found that trisubstituted purine derivatives are potent FLT3 inhibitors that selectively block the proliferation of AML cell lines harboring FLT3-ITD mutations.^12^ In order to identify potent and selective FLT3 inhibitors, we focused on synthesizing heterocyclic mimics of the purine base bearing similar substitution patterns as the parent purine derivatives. We designed trisubstituted derivatives containing various heterocyclic cores (Figure 1B) and then evaluated their inhibitory effects on FLT3 kinase in vitro and in vivo.

## Results and Discussion

Trisubstituted purines were previously described as potent CDK and FLT3-ITD kinase inhibitors.^12−15^ In order to explore this understudied chemical space and generate new active compounds, we designed new isosteric trisubstituted derivatives of several heterocyclic cores, including thieno[3,2-*d*]pyrimidine,^16−18^ pyrazolo[1,5-*a*]pyrimidine,^19−21^ imidazo[4,5-*b*]pyridine,^22^ pyrido[4,3-*d*]pyrimidine,^23^ and imidazo[1,2-*b*]pyridazine^24,25^ (Figure 1B). All of the prepared compounds were tested for their inhibitory activity against recombinant FLT3-ITD and CDK2/E. The most active compounds were screened against the FLT3-D835Y mutant, which is the most common resistance initiator in AML patients treated with clinically approved FLT3 inhibitors. To evaluate the FLT3-dependent mechanism of action, compounds were further screened for antiproliferative activity in a panel of human leukemia cell lines. Two AML cell lines, MV4-11 and MOLM-13, characterized by the presence of FLT3-ITD (full FLT3-dependency) and SEM, an acute lymphoblastic leukemia (ALL) cell line overexpressing FLT3-wt (with partial dependency on FLT3 signaling), were supplemented with four FLT3-independent cell lines. These included the AML-derived cell lines NOMO-1 and ML-2, the ALL-derived cell line CEM, and chronic myeloid leukemia (CML)-derived K562 cells.

### Synthesis and Activity of Thieno[3,2-*d*]pyrimidines

**Scheme 1:**
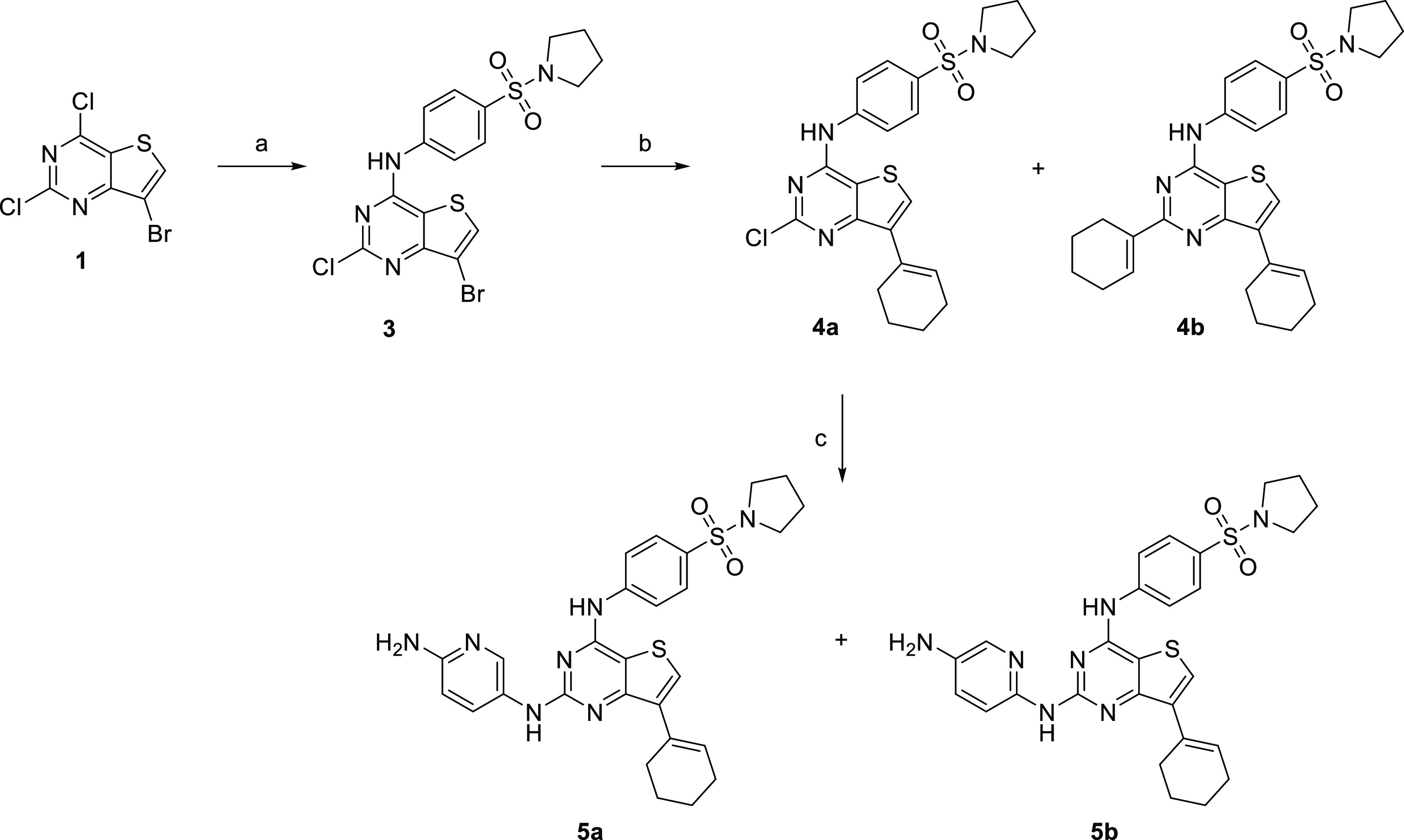
Synthesis of Thieno[3,2-*d*]pyrimidine Derivatives Reagents and conditions: (a) 4-(pyrrolidin-1-ylsulfonyl)aniline (**2**), *t*-BuOK, *N*,*N*-dimethylformamide (DMF), 0 °C; (b) cyclohex-1-en-1-ylboronic acid, Cs_2_CO_3_, Pd(dppf)Cl_2_·dichloromethane (DCM), dioxane, water, 95 °C; and (c) 2,5-diaminopyridine hydrochloride, Cs_2_CO_3_, XPhos Pd G2, DMF, 95 °C.

Thieno[3,2-*d*]pyrimidine derivatives (Scheme 1) were prepared from 7-bromo-2,4-dichlorothieno[3,2-*d*]pyrimidine (**1**).^26^ Reaction with 4-(pyrrolidin-1-ylsulfonyl)aniline^11,27^ (**2**) in the presence of *t*-BuOK at 0 °C afforded substituted derivative **3**. Subsequent Suzuki cross-coupling proceeded smoothly and afforded monoderivative **4a** as a major product together with dialkylated compound **4b**. Buchwald–Hartwig cross-coupling of chloro derivative **4a** produced a mixture of two isomers **5a** and **5b** in 10 and 25% yield, respectively. Heating of **4a** with *trans*-1,4-cyclohexyldiamine led to the decomposition of the starting material; only traces of the product were detected (data not shown).

Compounds containing the thieno[3,2-*d*]pyrimidine core did not display significant inhibitory activity against recombinant FLT3-ITD. Their antiproliferative activities against leukemic cell lines varied mainly within the micromolar range (see Table 1).

**Table 1:** Kinase-Inhibitory and Antiproliferative Activities of Thieno[3,2-*d*]pyrimidine Derivatives

	IC_50_ (μM)^a^			GI_50_ (μM)^a^						
	FLT3-ITD	FLT3-D835Y	CDK2/E	MV4-11	MOLM-13	SEM	CEM	NOMO-1	ML-2	K562
**4a**	>20	NT	>20	1.765	3.260	>6.25	>6.25	>10	>10	9.890
**4b**	>20	NT	>20	2.570	6.010	4.400	>6.25	8.945	6.775	8.533
**5a**	5.098	NT	17.204	1.665	2.180	1.467	7.490	8.030	4.243	6.595
**5b**	>20	NT	>20	2.705	6.075	2.595	>10	>10	7.335	>10

a For standard deviation (SD) values, see Table S3 in the Supporting Information. NT = not tested.

### Synthesis and Activity of Pyrazolo[1,5-*a*]pyrimidines

**Scheme 2:**
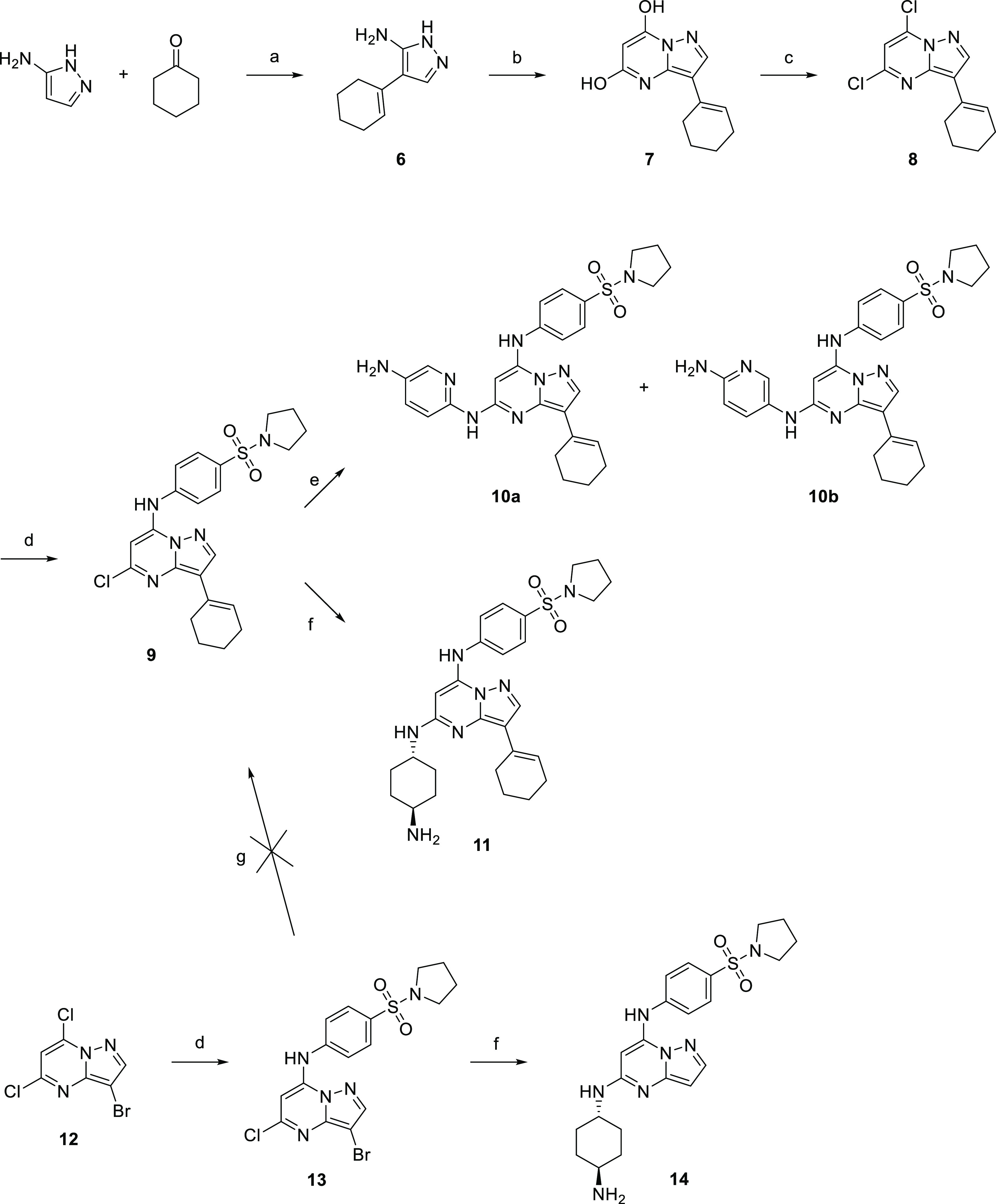
Synthesis of Pyrazolo[1,5-*a*]pyrimidine Derivatives Reagents and conditions: (a) AcOH, RT; (b) Na, CH_2_(COOEt)_2_, EtOH, reflux; (c) POCl_3_, *N*,*N*-dimethylaniline, 80 °C; (d) 4-(pyrrolidin-1-ylsulfonyl)aniline (**2**), *t*-BuOK, DMF, 0 °C; (e) 2,5-diaminopyridine hydrochloride, Cs_2_CO_3_, Pd_2_(dba)_3_, Xantphos, DMF, 120 °C; (f) *trans*-1,4-diaminocyclohexane, *N*-methylpyrrolidone (NMP), 210 °C; and (g) cyclohex-1-en-1-ylboronic acid, Cs_2_CO_3_, Pd(dppf)Cl_2_·DCM, dioxane, water, 95 °C.

First, the cyclohexenyl ring in pyrazolo[1,5-*a*]pyrimidine derivatives (Scheme 2) had to be installed by cyclization of 4-(cyclohex-1-en-1-yl)-1*H*-pyrazol-5-amine^28^ (**6**) with diethyl malonate^29,30^ due to the low reactivity of bromo derivative **13** in the subsequent Suzuki cross-coupling reaction. Dihydroxy derivative **7** was refluxed in POCl_3_ to give dichloro derivative **8**, which was further converted to sulfonamide **9**. Buchwald–Hartwig cross-coupling of **9** with 2,5-diaminopyridine afforded derivatives **10a** and **10b** in moderate yields. Heating of **9** with *trans*-1,4-cyclohexyldiamine gave compound **11**. However, heating of **13** with *trans*-1,4-cyclohexyldiamine at 210 °C overnight afforded debrominated amino derivative **14**.

Compounds from the pyrazolo[1,5-*a*]pyrimidine series showed poor potency against the tested recombinant kinases. Their antiproliferative activities did not reach measurable values in most of the compounds (GI_50_ > 10 μM; Table 2). Only disubstituted pyrazolo[1,5-*a*]pyrimidine derivative **14** bearing 4-(pyrrolidin-1-ylsulfonyl)aniline and aminocyclohexylamino substituents in positions 7 and 5, respectively, and trisubstituted derivative **11** bearing an additional cyclohexenyl in position 3 showed submicromolar activities against FLT3-ITD and CDK2. Also, FLT3-ITD-positive MV4-11 and MOLM-13 cell lines were more sensitive to treatment than FLT3-independent cell lines, indicating the FLT3-dependent mechanism of action (Table 2).

**Table 2:** Kinase-Inhibitory and Antiproliferative Activities of Pyrazolo[1,5-*a*]pyrimidine Derivatives

	IC_50_ (μM)^a^			GI_50_ (μM)^a^						
	FLT3-ITD	FLT3-D835Y	CDK2/E	MV4-11	MOLM-13	SEM	CEM	NOMO-1	ML-2	K562
**9**	>20	NT	>20	7.360	3.285	1.575	3.595	>10	>10	>10
**10a**	10.612	NT	>20	>10	>10	>10	>10	>10	>10	>10
**10b**	2.507	NT	>20	5.737	3.280	9.840	>10	>10	>10	>10
**11**	0.540	0.109	0.774	1.817	2.553	5.510	>10	>10	>10	>10
**14**	0.623	0.272	0.100	0.690	1.413	7.905	>10	>10	>10	>10

a For SD values, see Table S3 in the Supporting Information. NT = not tested.

### Synthesis and Activity of Imidazo[4,5-*b*]pyridine Derivatives

**Scheme 3:**
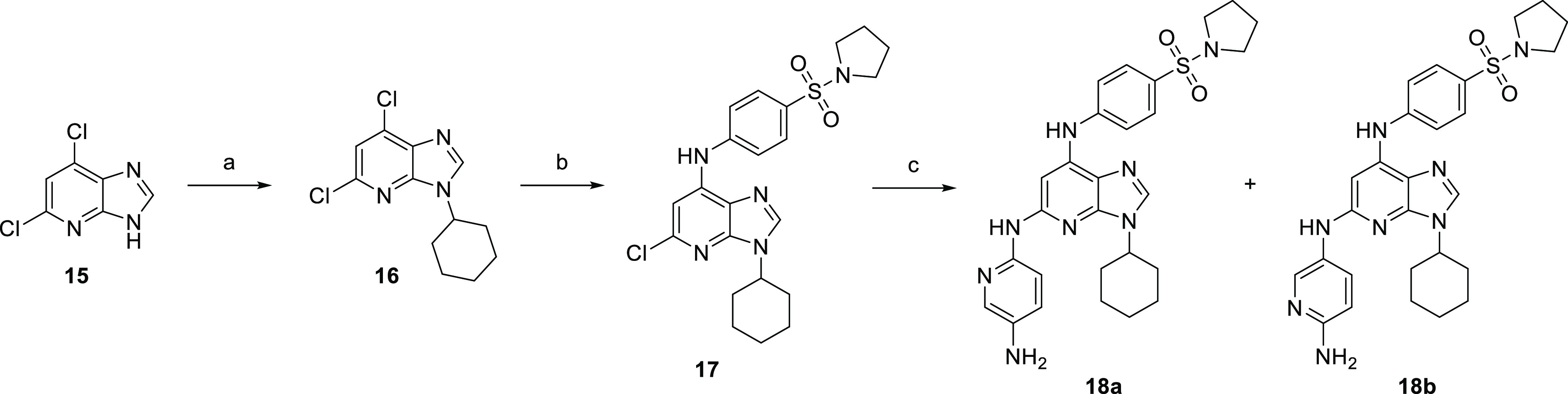
Synthesis of Imidazo[4,5-*b*]pyridine Derivatives Reagents and conditions: (a) cyclohexanol, Ph_3_P, diisopropyl azodicarboxylate (DIAD), dioxane, RT; (b) 4-(pyrrolidin-1-ylsulfonyl)aniline (**2**), *t*-BuOK, DMF, 0 °C; and (c) 2,5-diaminopyridine hydrochloride, Cs_2_CO_3_, Pd_2_(dba)_3_, Xantphos, DMF, 115 °C.

Imidazo[4,5-*b*]pyridine derivatives (Scheme 3) were prepared by alkylation of commercially available 5,7-dichloro-1*H*-imidazo[4,5-*b*]pyridine (**15**) under Mitsunobu conditions.^31,32^ Further substitution with aniline substituent and Buchwald–Hartwig amination with 2,5-diaminopyridine afforded derivatives **18a** and **18b** (Scheme 3).

Imidazo[4,5-*b*]pyridine derivatives **18a** and **18b** with 4-(pyrrolidin-1-ylsulfonyl)aniline substituent in position 7, cyclohexyl moiety in position 3, and 6-aminopyridin-2/3-yl in position 5 displayed submicromolar activities against recombinant FLT3-ITD and FLT3-D835Y (Table 3). Moreover, antiproliferative activities established in the panel of leukemia cell lines indicated an FLT3-dependent mechanism of action. Nevertheless, the rather weak potency of these molecules required further modification of the heterocycle core.

**Table 3:** Kinase-Inhibitory and Antiproliferative Activities of Imidazo[4,5-*b*]pyridine Derivatives

	IC_50_ (μM)^a^			GI_50_ (μM)^a^						
	FLT3-ITD	FLT3-D835Y	CDK2/E	MV4-11	MOLM-13	SEM	CEM	NOMO-1	ML-2	K562
**17**	2.453	NT	>20	5.520	3.075	>10	>10	>10	9.530	>10
**18a**	0.430	0.479	>20	1.877	1.540	2.380	>10	>10	>10	>10
**18b**	0.134	0.392	>20	0.735	0.335	1.565	>10	8.565	5.835	8.180

a For SD values, see Table S3 in the Supporting Information. NT = not tested.

### Synthesis and Activity of Pyrido[4,3-*d*]pyrimidines

**Scheme 4:**
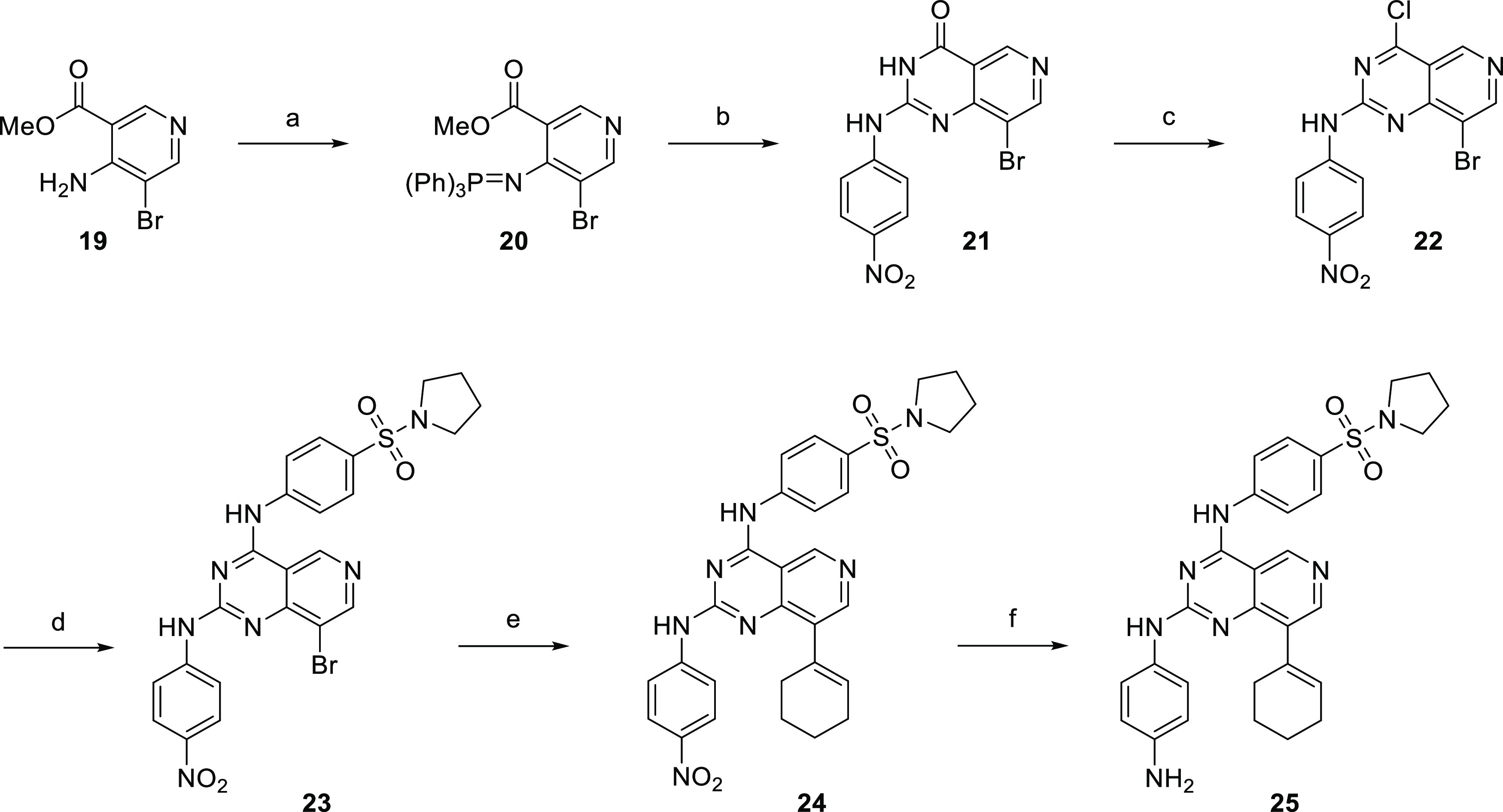
Synthesis of Pyrido[4,3-*d*]pyrimidine Derivatives Reagents and conditions: (a) Ph_3_P, Br_2_, triethylamine (TEA), DCM, 0 °C to RT; (b) (i) 4-nitrophenyl isocyanate, tetrahydrofuran (THF), RT; (ii) NH_3_, RT; (c) POCl_3_, *N*,*N*-dimethylaniline; (d) 4-(pyrrolidin-1-ylsulfonyl)aniline (**2**), *t*-BuOK, DMF, 0 °C; (e) cyclohex-1-en-1-ylboronic acid, Cs_2_CO_3_, Pd(dppf)Cl_2_·DCM, DMF, water, 80 °C; and (f) Pd/C (10% wt), H_2_, 15 bar, RT.

Pyrido[4,3-*d*]pyrimidine derivatives (Scheme 4) were prepared according to a procedure described by Jansa et al.^33^ Activation of bromonicotinate with triphenylphosphine and ring closure with isocyanate afforded derivative **21**. Chlorination and subsequent substitution with an aniline derivative afforded compound **23**, which reacted with cyclohexene-1-boronic acid to give derivative **24** in a high yield. Final reduction with H_2_ (15 bar) on Pd/C for 2 days afforded amino derivative **25** with an unsaturated cyclohexene ring. However, modification of the pyrido[4,3-*d*]pyrimidine core proved counterproductive, given the prepared compounds failed to show any promising activity (Table 4).

**Table 4:** Kinase-Inhibitory and Antiproliferative Activities of Pyrido[4,3-*d*]pyrimidine Derivatives

	IC_50_ (μM)^a^			GI_50_ (μM)^a^						
	FLT3-ITD	FLT3-D835Y	CDK2/E	MV4-11	MOLM-13	SEM	CEM	NOMO-1	ML-2	K562
**23**	>20	NT	>20	3.900	1.675	1.340	3.365	7.510	8.295	8.710
**24**	>20	NT	>20	>10	>10	>10	>10	>10	>10	>10
**25**	1.907	NT	>20	6.560	4.520	7.350	>10	>10	>10	9.530

a For SD values, see Table S3 in the Supporting Information. NT = not tested.

### Synthesis and Activity of Imidazo[1,2-*b*]pyridazines

**Scheme 5:**
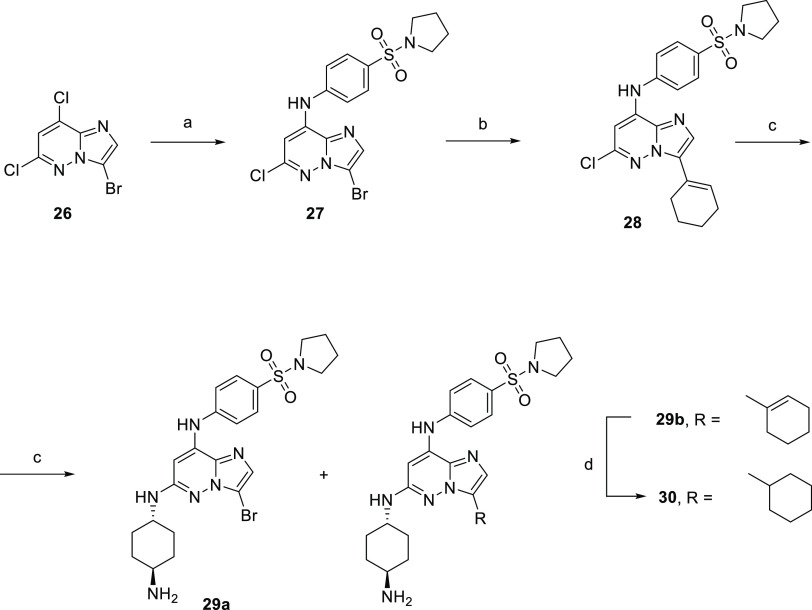
Synthesis of Imidazo[1,2-*b*]pyridazine Derivatives Reagents and conditions: (a) 4-(pyrrolidin-1-ylsulfonyl)aniline (**2**), *t*-BuOK, DMF, 0 °C; (b) cyclohex-1-en-1-ylboronic acid, Cs_2_CO_3_, Pd(dppf)Cl_2_·DCM, dioxane, water, 95 °C; (c) *trans*-1,4-diaminocyclohexane, NMP, 210 °C; and (d) Pd/C (10% wt), H_2_, EtOAc, MeOH, 15 bar, RT.

Finally, we focused on imidazo[1,2-*b*]pyridazine derivatives (Scheme 5). We started with 3-bromo-6-chloro derivative **27**. However, it showed very poor reactivity under Suzuki cross-coupling conditions and afforded only a small amount of **28** together with the starting material as an inseparable mixture. Next, we treated the mixture with 1,4-*trans*-cyclohexendiamine, separated the products by reverse phase chromatography, and isolated compounds **29a** and **29b**. Unsaturated derivative **29b** was hydrogenated by H_2_ on Pd/C to give **30** (Scheme 5). Attempts to prepare 2,5-diaminopyridine derivatives using the Buchwald–Hartwig reaction failed, and 6-chloro derivative **28** proved poorly reactive.

The first candidates of the imidazo[1,2-*b*]pyridazine series shared the 4-(pyrrolidin-1-ylsulfonyl)aniline substituent in position 8 and the *trans*-1,4-diaminocyclohexyl substituent in position 6. Substituents in position 3 included Br (**29a**), cyclohexyl (**30**), and cyclohexenyl (**29b**). These compounds displayed promising inhibitory activities against the tested recombinant kinases within the nanomolar range. Antiproliferative activities confirmed that these compounds employed an FLT3-dependent mechanism of action: FLT3-dependent MV4-11 and MOLM-13, as well as SEM cell lines, were several times more sensitive than FLT3-independent cell lines (Table 5).

**Table 5:** Kinase-Inhibitory and Antiproliferative Activities of Imidazo[1,2-*b*]pyridazine Derivatives

	IC_50_ (μM)^a^			GI_50_ (μM)^a^						
	FLT3-ITD	FLT3-D835Y	CDK2/E	MV4-11	MOLM-13	SEM	CEM	NOMO-1	ML-2	K562
**29a**	0.002	0.002	0.003	0.0001	0.004	0.008	0.623	0.240	0.118	0.320
**29b**	0.005	0.004	0.037	0.001	0.024	0.225	1.073	1.380	0.596	0.722
**30**	0.006	0.012	0.211	0.279	0.070	0.655	1.910	4.875	3.100	1.363

a For SD values, see Table S3 in the Supporting Information.

**Figure 2:**
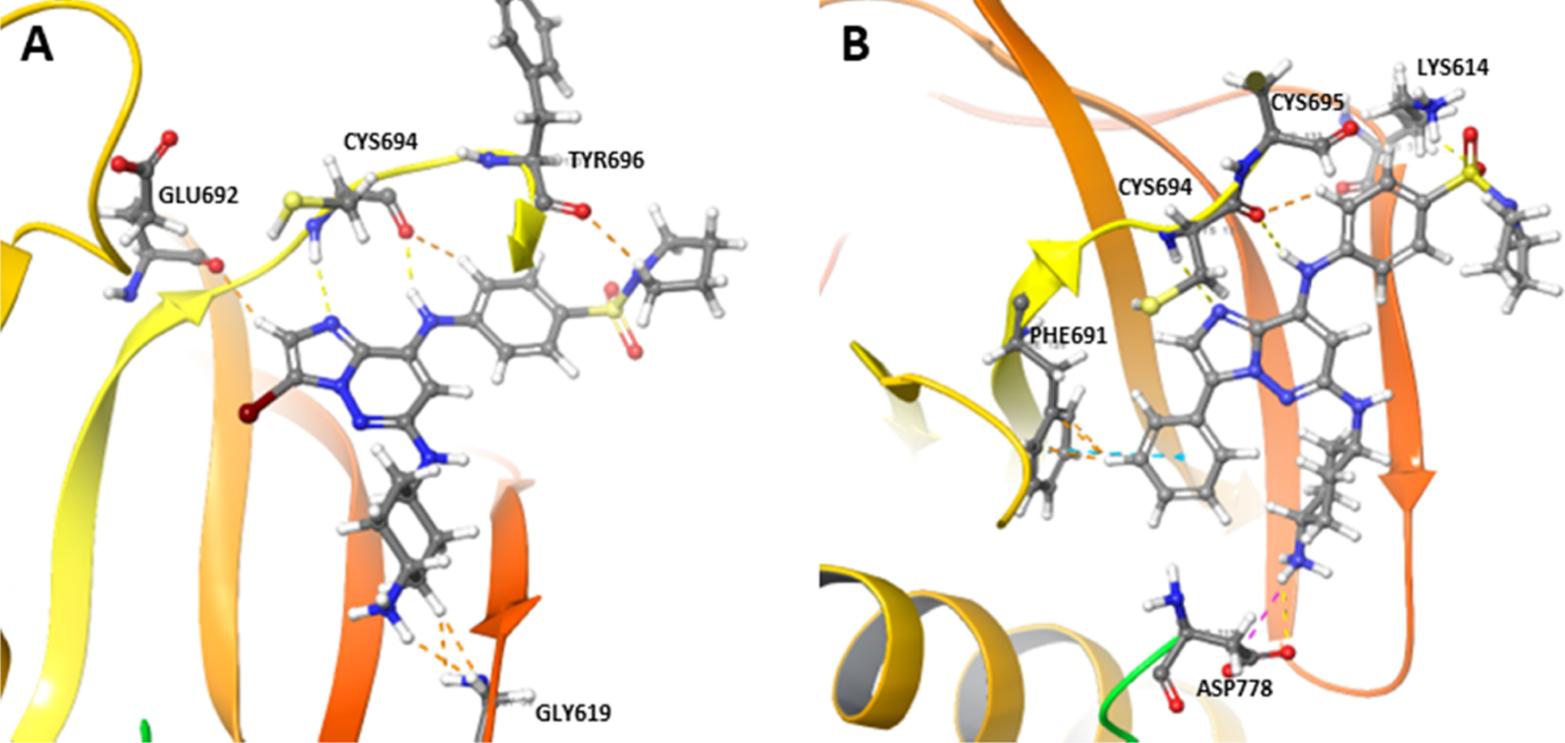
Docked binding poses of (A) compound **29a** and (B) its phenyl derivative **34f** in the active FLT3 site.

In the first part of this study, we identified new compounds by scaffold hopping and evaluated central cores as suitable replacements for the purine scaffold. As the most promising inhibitory activities were observed for imidazo[1,2-*b*]pyridazine derivatives, we decided to extend the series and modify the substituent in position 3 of the core. We performed a docking study using the active site of FLT3 to predict the binding poses of imidazo[1,2-*b*]pyridazine derivatives bearing aliphatic and aromatic substituents. We based the structures of the proposed ligands on the most potent inhibitor identified up to this point in the study, compound **29a** (Figure 2A). Various aliphatic and aromatic substituents were placed in position 3 of the heterocycle to induce interaction with a pocket lined by A642, K644, V675, F691, and L767. Proposed analogues of **29a** were docked in silico, and the binding affinity of each compound was evaluated using Glide built-in scoring functions (Table S2, Supporting Information). In agreement with previously published docking studies,^12^ our results confirmed that the most important residues participating in the interaction are K614, C694, N765, and D778. Another residue that proved important was F691 in the hydrophobic cavity, which presumably interacts with aromatic residues such as phenyl in **34f** (Figure 2B), pyrazole, or other hydrophobic species. A docking study also suggests that the binding mode of our molecules is similar to that of type I FLT3 inhibitors.

**Scheme 6:**
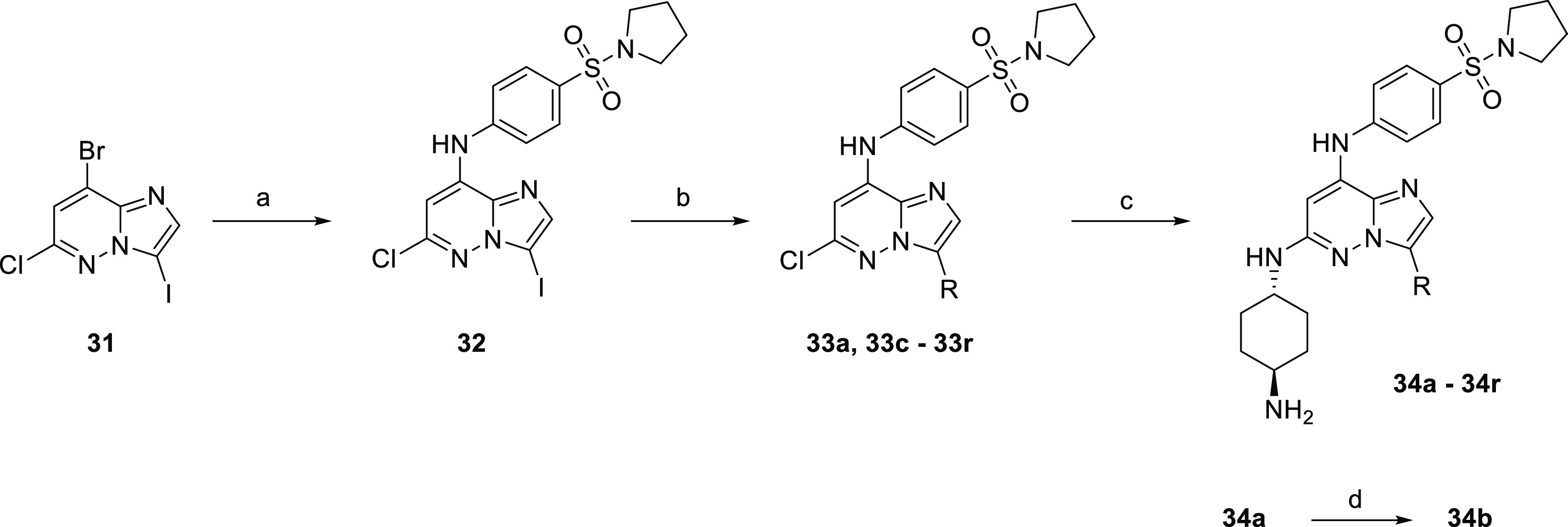
Synthesis of Imidazo[1,2-*b*]pyridazine Derivatives Modified in Position **3** Reagents and conditions: (a) 4-(pyrrolidin-1-ylsulfonyl)aniline (**2**), *t*-BuOK, DMF, 0 °C; (b) for **33a** and **33f**–**33r**: boronic acid, Cs_2_CO_3_, Pd(dppf)Cl_2_·DCM, dioxane, water, 95 °C; for **33c** and **33d**: alkylzinc bromide, Pd(dppf)Cl_2_·DCM, THF, 45 °C; for **33e**: Pd_2_(dba)_3_, XPhos, DABAL-Me_3_, 60 °C; (c) *trans*-1,4-diaminocyclohexane, NMP, 210 °C; and (d) Pd/C (10% wt), H_2_, EtOAc, MeOH, 15 bar, RT.

**Scheme 7:**
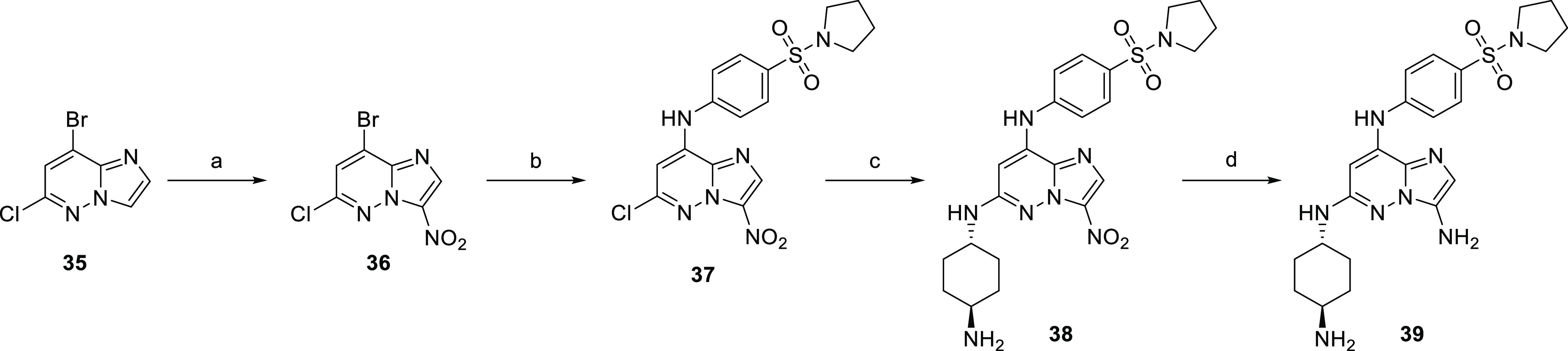
Synthesis of the 3-Aminoimidazo[1,2-*b*]pyridazine Derivative Reagents and conditions: (a) H_2_SO_4_, HNO_3_, 0 °C to RT; (b) 4-(pyrrolidin-1-ylsulfonyl)aniline (**2**), *t*-BuOK, DMF, 0 °C; (c) *trans*-1,4-diaminocyclohexane, NMP, 210 °C; and (d) SnCl_2_, EtOH, reflux.

**Scheme 8:**
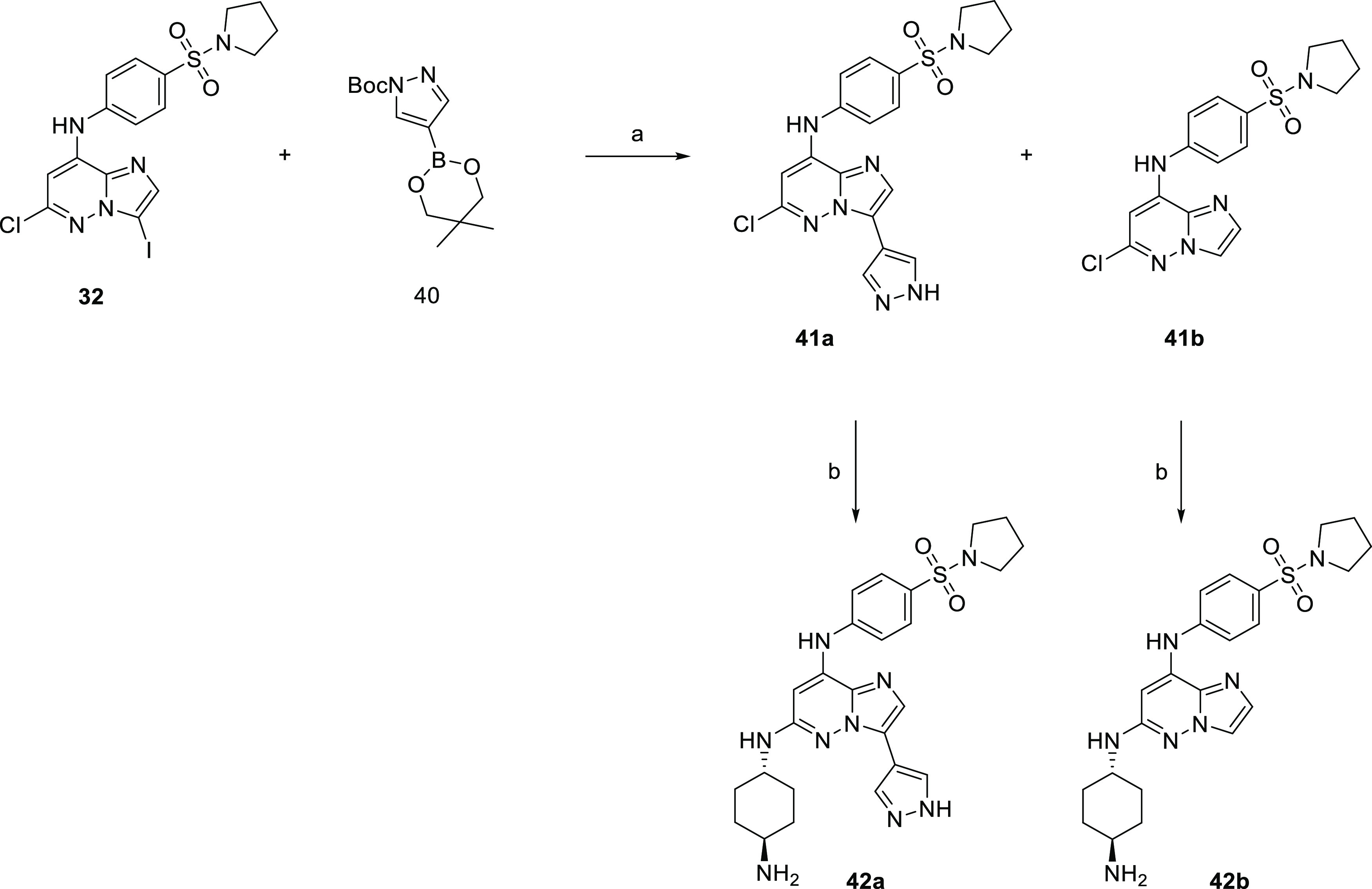
Synthesis of the 3-(1*H*-Pyrazole-4-yl)imidazo[1,2-*b*]pyridazine Derivative Reagents and conditions: (a) Na_2_CO_3_, Pd(dppf)Cl_2_·DCM, dioxane, water, 100 °C and (b) *trans*-1,4-diaminocyclohexane, NMP, 210 °C.

Based on our preliminary biological results and in silico docking analysis, we extended the imidazo[1,2-*b*]pyridazine series and prepared derivatives substituted in position 3 with various aliphatic and aromatic substituents (Table 6, Schemes 6–8). As the phenyl derivative **34f** showed activity toward FLT3-ITD-positive kinase in the single-digit nanomolar range together with high selectivity in comparison with CDK2, we extended our study to phenyl derivatives substituted at various positions in the phenyl ring (Table 6, entries g–p). The series was prepared from 3-iodo derivative **32** (Scheme 6),^34^ which is more reactive than 3-bromo derivative **27** used in the previous synthesis (Scheme 5).

**Table 6:**
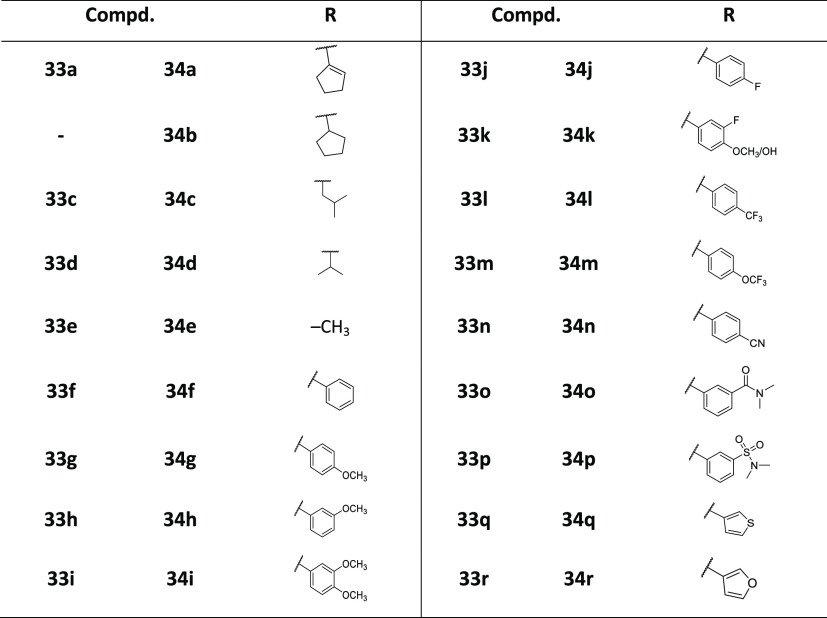
Substituted Imidazo[1,2-*b*]pyridazine Derivatives in Position 3 of the Heterocyclic Core

While the reaction of **32** with sodium azide did not proceed (data not shown), 3-amino derivative **39** was synthesized via 3-nitro intermediate **36** by nitration^35^ of imidazo[1,2-*b*]pyridazine **35** and further substitution of the heterocyclic core (Scheme 7).

Finally, the 3-pyrazolo derivative was prepared by Suzuki coupling of 3-iodo derivative **32** and protected 1*H*-pyrazole-4-yl-boronic acid (**40**). The reaction gave a mixture of deprotected product **41a** and dehalogenated starting material **41b**. Reaction with *trans*-1,4-diaminocyclohexane afforded compounds **42a** and **42b**, respectively (Scheme 8).

We explored the structure–activity relationship using a diverse series of compounds bearing the conserved 4-(pyrrolidin-1-ylsulfonyl)aniline substituent in position 8 (Table 7). Although a number of compounds featuring chloro substitution in position 6 were tested against FLT3-ITD as well, our results confirmed (Table S4, Supporting Information) that the introduction of the *trans*-1,4-diaminocyclohexyl substituent into this position is crucial for the anti-FLT3 activity of imidazo[1,2-*b*]pyridazines.

**Table 7:** Kinase-Inhibitory and Antiproliferative Activities of Imidazo[1,2-*b*]pyridazine Derivatives

	IC_50_ (μM)^a^			GI_50_ (μM)^a^						
	FLT3-ITD	FLT3-D835Y	CDK2	MV4-11	MOLM-13	SEM	CEM	NOMO-1	ML-2	K562
**34a**	0.007	0.004	0.196	0.005	0.007	0.372	3.800	8.430	5.780	6.870
**34b**	0.005	0.002	0.005	0.008	0.007	0.235	0.917	0.620	0.480	0.885
**34c**	0.008	0.002	0.083	0.028	0.030	0.453	3.727	2.435	1.078	1.725
**34d**	0.004	0.001	0.011	0.005	0.009	0.210	0.810	0.848	0.490	0.635
**34e**	0.009	0.002	0.093	0.005	0.007	0.132	0.683	0.700	0.455	1.310
**34f**	0.004	0.001	0.493	0.007	0.009	0.140	1.768	4.275	3.030	1.525
**34g**	0.001	0.002	2.119	0.045	0.040	0.283	2.645	5.223	3.505	1.640
**34h**	0.002	0.002	0.961	0.073	0.176	1.060	6.170	>10	6.865	1.010
**34i**	0.002	0.006	1.435	0.060	0.027	0.877	6.380	5.085	4.220	2.255
**34j**	0.001	0.001	0.234	0.023	0.030	0.150	1.690	5.030	2.050	1.063
**34k**	0.001	0.002	0.037	0.017	0.019	0.590	1.940	0.865	0.325	2.120
**34l**	0.004	0.011	1.443	0.156	0.200	1.031	1.570	5.025	3.923	1.505
**34m**	0.013	0.025	6.587	0.790	0.410	1.335	4.750	4.260	3.310	1.550
**34n**	0.001	0.002	0.366	0.042	0.025	0.168	0.760	0.393	0.245	1.655
**34o**	0.231	0.569	3.428	0.710	0.935	2.525	8.990	9.035	4.475	>10
**34p**	0.142	0.834	2.435	1.183	1.160	1.500	8.400	2.385	2.000	4.128
**34q**	0.001	0.003	0.082	0.002	0.004	0.381	1.400	1.455	0.440	1.470
**34r**	0.001	0.002	0.031	0.001	0.001	0.399	1.030	0.755	0.270	0.570
**39**	0.333	0.268	1.116	1.557	2.213	>10	>10	>10	>10	>10
**42a**	0.002	0.001	0.031	0.006	0.011	0.823	7.363	1.745	0.370	9.850
**42b**	0.106	0.014	0.178	0.039	0.097	0.523	1.423	4.733	3.650	5.545

a For SD values, see Table S3 in the Supporting Information.

Compounds lacking the substituent in position 3 (**42b**) or containing a small polar amino group (**39**) are among the less potent in the series displaying IC_50_ values against FLT3-ITD within a high nanomolar range. The introduction of small aliphatic substituents (methyl in **34e**, isopropyl in **34d**, isobutyl in **34c**), cyclic aliphatic substituents (**34b**, **34a**, **30**, **29b**), or furanyl (**34r**) and thienyl (**34q**) resulted in low nanomolar activity against recombinant FLT3-ITD as well as FLT3-D835Y. These results correspond with the potent antiproliferative activities in MV4-11 and MOLM-13 cell lines within a nanomolar concentration range. In contrast, FLT3-independent cell lines were several orders of magnitude less sensitive. On the other hand, these compounds showed very strong potency against CDK2, where the inhibitory ratio of CDK2 and FLT3-ITD was between 1 and 82. These results indicate the lower selectivity of these molecules.

As described in the literature,^13^ larger substituents in position 9 of the purine ring lead to decreased activity of trisubstituted purines toward CDK2. Further, our docking analysis suggests that phenyl substituent may successfully bind to the FLT3-ITD active site. Therefore, we focused on derivatives bearing phenyl substituent (**34f**) and its substituted derivatives (**34g–34p**) in position 3 of the imidazo[1,2-*b*]pyridazine ring to improve the selectivity of the compounds over CDK2. Compounds containing phenyl (**34f**), 4- and 3-methoxyphenyl (**34g** and **34h**, respectively), 3,4-dimethoxyphenyl (**34i**), 4-fluorophenyl (**34j**), 3-fluoro-4-hydroxyphenyl (**34k**), 4-cyanophenyl (**34n**), and 4-trifluoromethyl- or 4-trifluoromethoxyphenyl (**34l** and **34m**, respectively) retained FLT3 inhibitory activity within low nanomolar ranges. However, as expected, CDK2 inhibitory activity dropped dramatically to micromolar or submicromolar concentrations in most compounds (except for **34k**, which displayed an anti-CDK2 IC_50_ value of 37 nM). A ratio between CDK2 and FLT3-ITD of more than 200 highlighted the improved selectivity of these substances, albeit accompanied by a slight decrease in antiproliferative activity. Nevertheless, these compounds still exhibited potency against FLT3-ITD-positive AML cell lines, whereas FLT3-independent cell lines were far less sensitive. This confirms the FLT3-dependent mechanism of action.

On the other hand, dimethylcarbamoyl and dimethylsulfamoyl substituents of compounds **34o** and **34p**, respectively, probably affected the binding of compounds into active sites of the tested kinases and resulted in reduced activity. For example, IC_50_ values increased more than 100-fold in comparison with the values of other members of the group.

From all of the prepared compounds, **34f** was selected as the tool compound for further biochemical and mechanistic evaluation. This molecule showed single-digit nanomolar IC_50_ values against recombinant FLT3-ITD and FLT3-D835Y (0.004 and 0.001 μM, respectively), whereas CDK2 was nearly 250 times less sensitive. FLT3-ITD-inhibitory activity of **34f** was comparable to the standards quizartinib (0.010 ± 0.004 μM) and gilteritinib (0.012 ± 0.001 μM). Although **34f** shows promising potency also against FLT3-D835Y (0.001 μM), comparable to the clinically approved gilteritinib (0.002 ± 0.0003 μM), quizartinib is more than 100 times less potent against FLT3-D835Y than **34f** (0.136 ± 0.002 μM for quizartinib). The same trend was also observed for FLT3-ITD-F691L. While **34f** and gilteritinib showed low nanomolar IC_50_ values (0.004 ± 0.003 and 0.010 ± 0.005 μM, respectively), quizartinib lost its potency against this mutant variant of FLT3 (>5 μM).

In addition to its outstanding FLT3 inhibitory activity, **34f** displayed strong antiproliferative efficacy in the FLT3-ITD-positive MV4-11 and MOLM-13 cell lines (0.013 and 0.020 μM, respectively). In contrast, GI_50_ values measured in FLT3-independent cell lines were in the micromolar range (Table 7). Our results of antiproliferative activities were comparable to the data obtained for quizartinib and gilteritinib. The GI_50_ values for FLT3-ITD-positive cell lines varied in the nanomolar range for both quizartinib (MV4-11: 0.003 ± 0.001 μM; MOLM-13: 0.004 ± 0.004 μM) and gilteritinib (MV4-11: 0.026 ± 0.009 μM; MOLM-13: 0.034 ± 0.013 μM). FLT3-independent cell lines treated with quizartinib were not significantly affected by concentrations up to 10 μM, and the GI_50_ values obtained for gilteritinib varied in the micromolar range (CEM: 2.771 ± 0.229 μM, NOMO-1: 1.601 ± 0.226 μM, K562: 2.254 ± 0.486 μM).

### Synthesis of **34f** on a Larger Scale

**Scheme 9:**
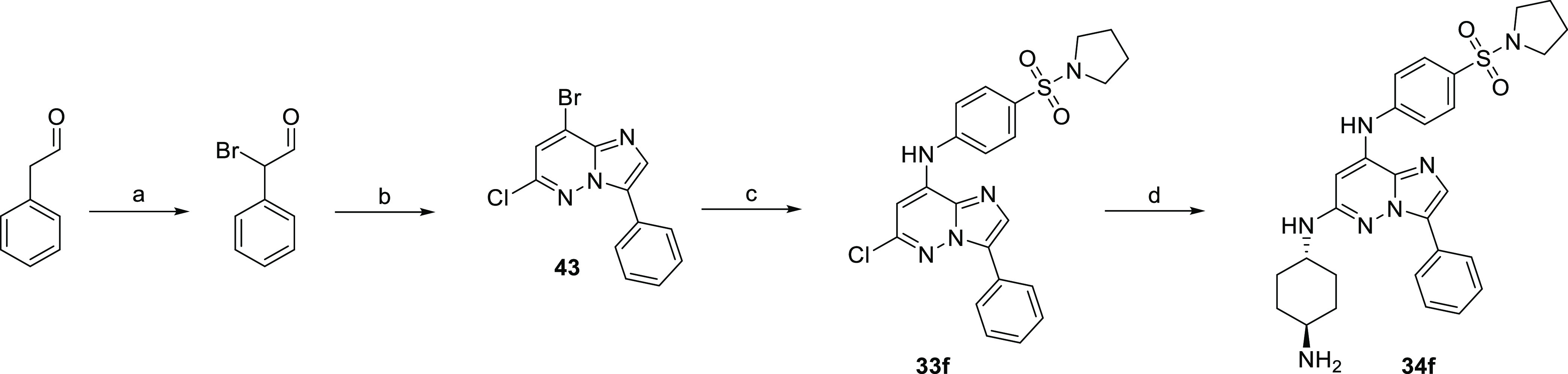
Synthesis of **34f** on a Larger Scale Reagents and conditions: (a) Br_2_, 1,4-dioxane, 0 °C to RT; (b) 3-amino-4-bromo-6-chloropyridazine, EtOH, 85 °C; (c) *t*-BuOK, amine (**2**), DMF, 0 °C; and (d) *trans*-1,4-diaminocyclohexane, NMP, 200 °C.

Compound **34f** was selected for in vivo experiments in mice, requiring the preparation of hundreds of milligrams of the material. Given that Suzuki cross-coupling of 3-iodo derivative **32** gave products in relatively low yields from 15 to 30% and upscaling of the reaction proved problematic, we developed an alternative synthetic procedure employing phenylacetaldehyde (Scheme 9). Bromination^36^ and subsequent cyclization with 3-aminopyridazine gave compound **43** in a 50% yield after two steps. Further substitution with aniline and amine gave **34f** in a 20% overall yield after four steps. This synthetic strategy, starting from cheap substituted acetaldehyde, proved suitable for the synthesis of larger quantities of 3-substituted imidazo[1,2-*b*]pyridazine derivatives.

#### Cellular Effects of **34f**

**Figure 3:**
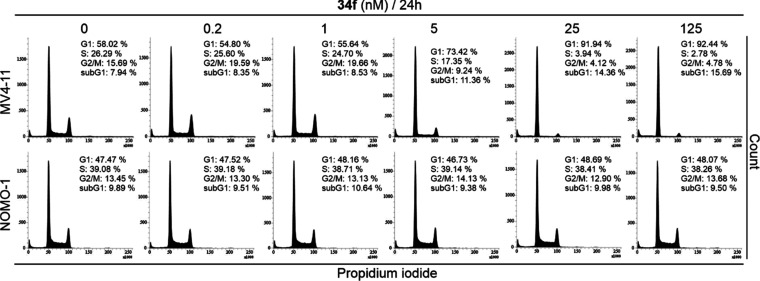
Cell cycle analysis of MV4-11 and NOMO-1 cells treated with **34f** for 24 h.

**Figure 4:**
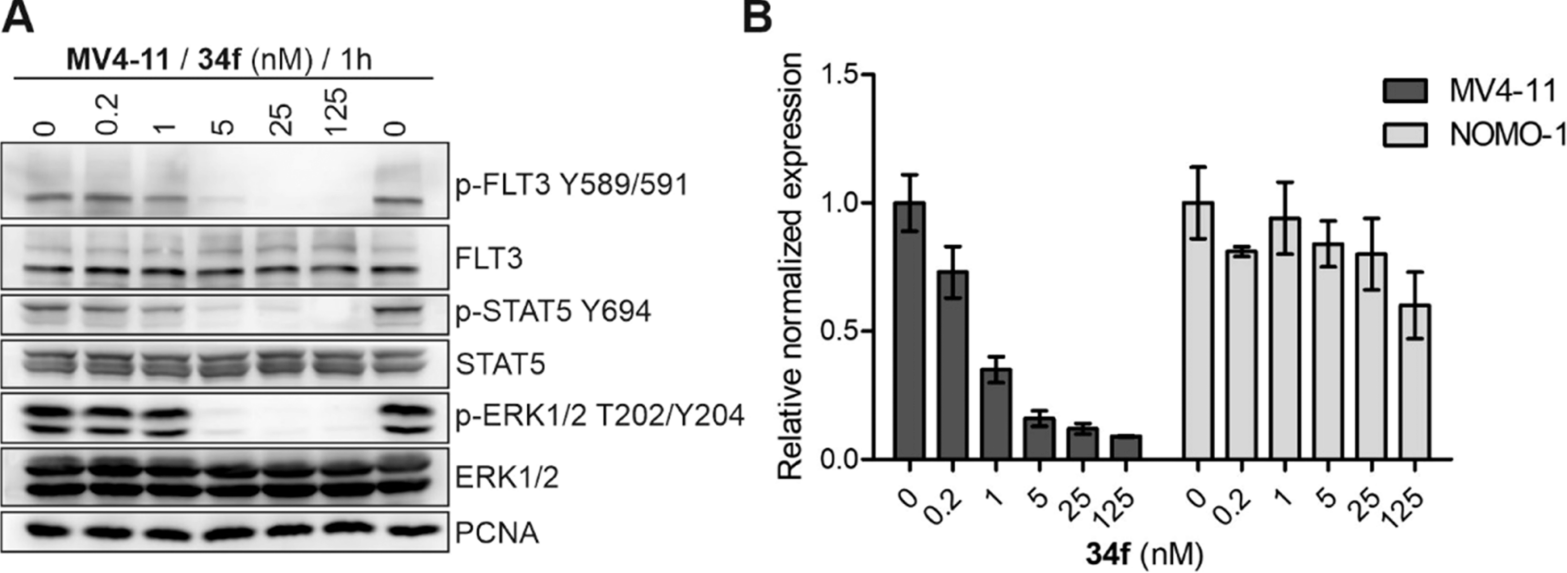
(A) Immunoblotting analysis of FLT3 and its downstream signaling pathways in MV4-11 treated with **34f** for 1 h. (B) Relative normalized expression of the MYC gene in MV4-11 and NOMO-1 cells treated with **34f** for 4 h.

Specific FLT3 inhibition induces G1 arrest of FLT3-dependent AML cells but does not affect other cell lines (FLT3-independent). This was validated by flow cytometry analysis of FLT3-ITD-positive MV4-11 cells treated for 24 h with nanomolar concentrations of **34f**. The number of G1 cells increased in a dose-dependent manner (Figure 3), but the NOMO-1 cell line was not affected within the same **34f** concentration range (Figure 3). These results were comparable to the effects seen in MV4-11 and NOMO-1 cells treated with both quizartinib and gilteritinib (Figures S1, S2). The primary cause of this phenomenon is the blocking of FLT3-subordinate signaling pathways, which are of crucial importance in cell proliferation. Dose-dependent attenuation of phosphorylation of FLT3 as well as its downstream targets, Y694 of STAT5 and T202/Y204 of ERK1/2, was confirmed after 1 h of treatment with **34f** in MV4-11 cells. This demonstrated the FLT3-dependent mechanism of action (Figure 4) and efficacy comparable to quizartinib and gilteritinib (Figures S3A, S4A).

Expression analysis of the MYC gene, a key transcription factor and common oncogene whose deregulation often contributes to the development of hematological malignancies, further confirmed the FLT3-dependent mechanism of action of **34f**. MYC transcript levels were significantly reduced by **34f** in treated MV4-11 cells, whereas they remained more stable in FLT3-independent NOMO-1 cells. Comparable effects were also observed in cells treated with quizartinib (Figure S3B) as well as gilteritinib (Figure S4B), a finding consistent with previously reported studies.^37^

**Figure 5:**
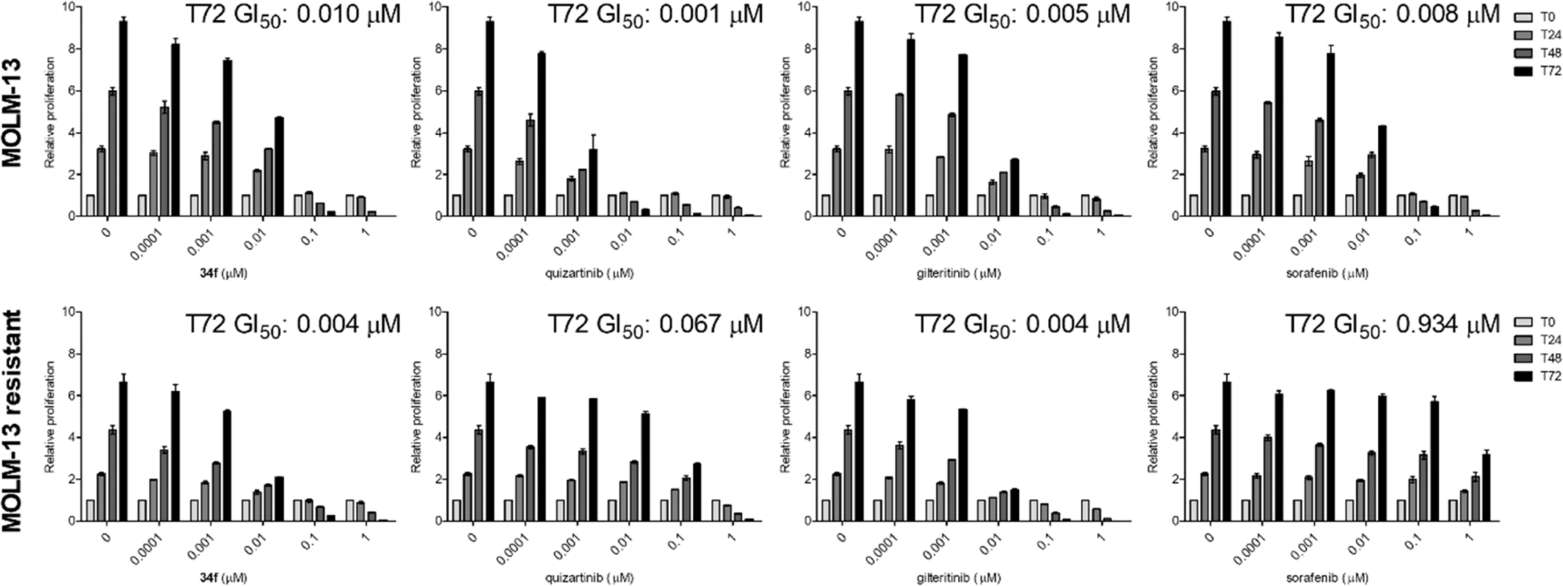
Antiproliferative activity of **34f** in the MOLM-13 cell line and its clone expressing FLT3-ITD-D835Y (MOLM-13 resistant). Quizartinib, gilteritinib, and sorafenib were used as standards. T72 GI_50_ = 50% growth inhibition concentration determined at the final point of the experiment after 72 h of treatment.

One of the most common obstacles to FLT3-inhibitor therapy of AML is the development of drug resistance. Therefore, we used the MOLM-13 cell line and its resistant clone expressing the FLT3-ITD-D835Y mutant to evaluate the effect of the lead compound **34f** on proliferation. FLT3 inhibitors sorafenib, gilteritinib, and quizartinib were used for comparison. The graphs of the relative proliferation of MOLM-13 cells (Figure 5) show that compound **34f**, as well as quizartinib, gilteritinib, and sorafenib, blocked proliferation in a time-dependent manner at low nanomolar concentrations. The antiproliferative ability of compound **34f** was also confirmed in MOLM-13-resistant cells with the D835Y mutation; the GI_50_ value obtained after 72 h treatment did not change significantly (0.010 and 0.004 μM in MOLM-13 and its resistant variant, respectively). A similar outcome was also observed for clinically approved gilteritinib. In contrast, the efficacy of quizartinib and sorafenib dramatically decreased, and the GI_50_ values increased significantly.

#### Kinase Selectivity of **34f**

**Figure 6:**
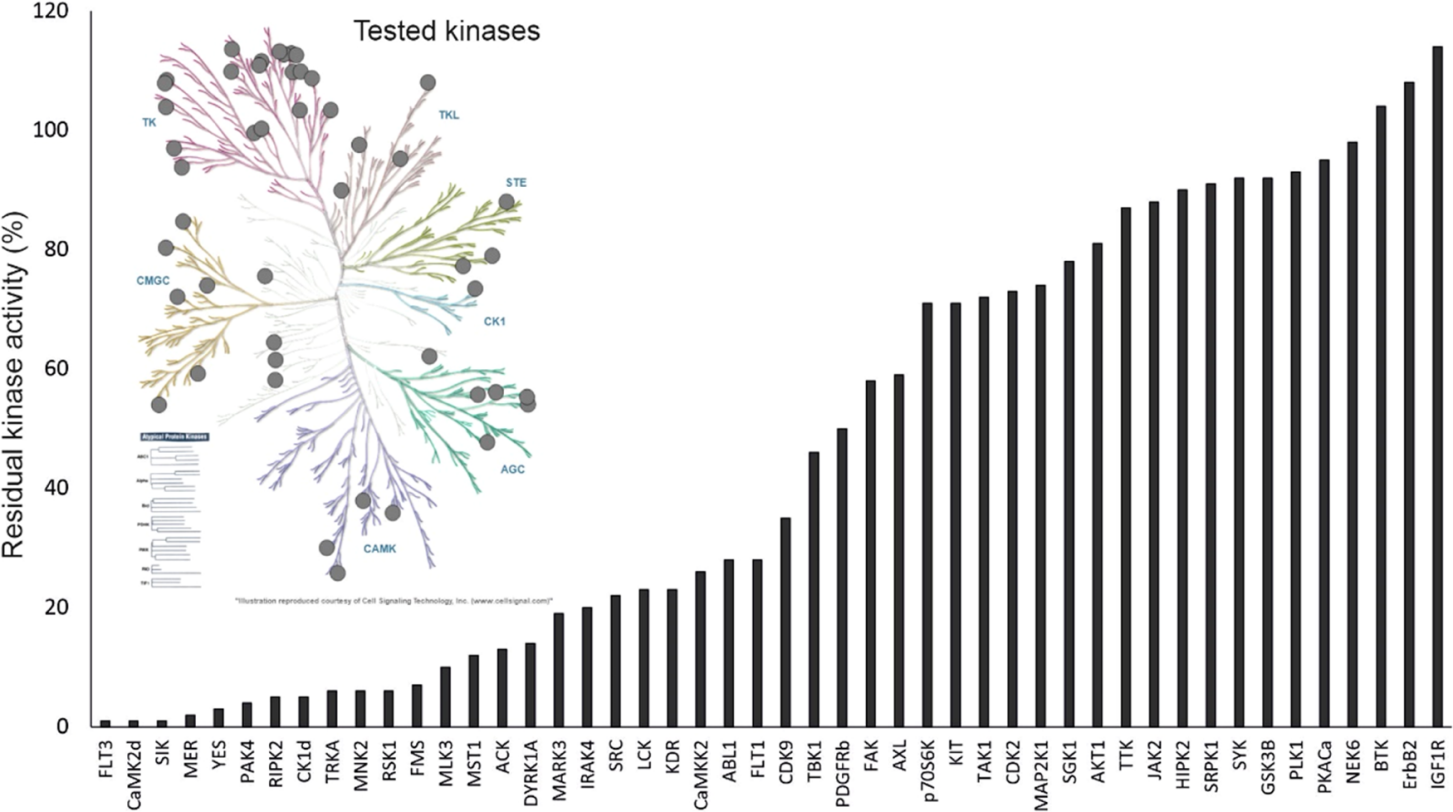
Kinase selectivity profiling of **34f**. The efficacy of **34f** at 100 nM concentration was compared with 48 human kinases across the kinome (coverage shown in the phylogenetic tree).

The preliminary kinase selectivity of **34f** in the panel of 48 kinases selected across the human kinome demonstrated the outstanding inhibitory activity of this compound against FLT3 (Figure 6). The IC_50_ values for the most important off-targets were determined (Table S5). Although **34f** also inhibits other kinases, it notably does not target KIT kinase, which is one of the most common off-targets of the known FLT3 inhibitors. Simultaneous inhibition of FLT3 and KIT results in myelosuppression,^38^ which complicates the clinical use of these compounds. Therefore, avoiding KIT inhibition is a crucial goal in the development of novel FLT3 inhibitors. Kinase selectivity profiling demonstrated that **34f** at a concentration of 100 nM reduced KIT activity to 71% (in comparison with 1% obtained for FLT3). A subsequent concentration-dependent experiment showed that the IC_50_ value of **34f** for KIT kinase is 680 nM (Table S5), a hundred times higher than for FLT3. These results indicate a favorable inhibitory ratio among these kinases. Hence, we decided to verify this finding using the Kasumi-1 cell line, which is characterized by activating N822K point mutation in KIT. Based on an evaluation of the antiproliferative properties of **34f**, the GI_50_ value measured in Kasumi-1 was 0.188 ± 0.019 μM, which was more than 18 times higher than the GI_50_ values obtained for quizartinib in Kasumi-1 (0.010 ± 0.004 μM) and for **34f** in FLT3-ITD MV4-11 cells (0.007 ± 0.004 μM). The GI_50_ value determined for gilteritinib in Kasumi-1 cells was 0.124 ± 0.013 μM. The limited ability of **34f** to block KIT activity was also confirmed by immunoblotting. At a concentration of 125 nM, **34f** only partially reduced the phosphorylation of two tyrosine residues (Y703 and Y719) of the KIT kinase and T202/Y204 in the KIT downstream ERK1/2 (Figure S5).

#### Plasma and Microsomal Stability of **34f**

In vitro stability of **34f** in blood plasma and liver microsomes (from human and mouse sources) was tested prior to in vivo experiments in order to predict the clearance of compounds in the whole organism. Propantheline bromide and verapamil were used as reference compounds for plasma and microsomal stability, respectively, demonstrating the usual stability profiles.

**Figure 7:**
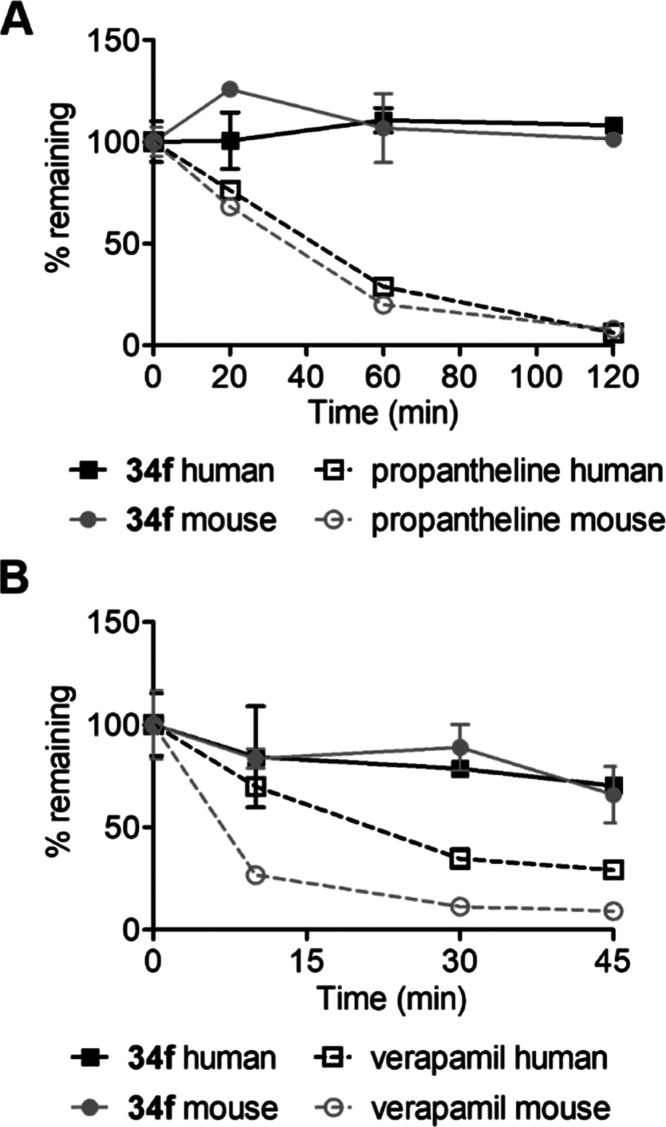
(A) Plasma and (B) microsomal stability of **34f**. Propantheline bromide and verapamil were used as standards to determine plasma stability and microsomal stability, respectively.

Compound **34f** was stable in both human and mouse plasma for up to 120 min of incubation (Figure 7A). As for microsomal stability, a slow decay by approximately 25% at 45 min was observed (Figure 7B). The calculated intrinsic clearance (CL_int_; Table S6) values were 18 μmol/min/mL for human microsomes and 13 μmol/min/mL for mouse microsomes, indicating that the compound falls within the low-to-moderate clearance category. Overall, the metabolic stability of **34f** was considered acceptable for in vivo experiments.

#### In Vivo Efficacy of **34f**

**Figure 8:**
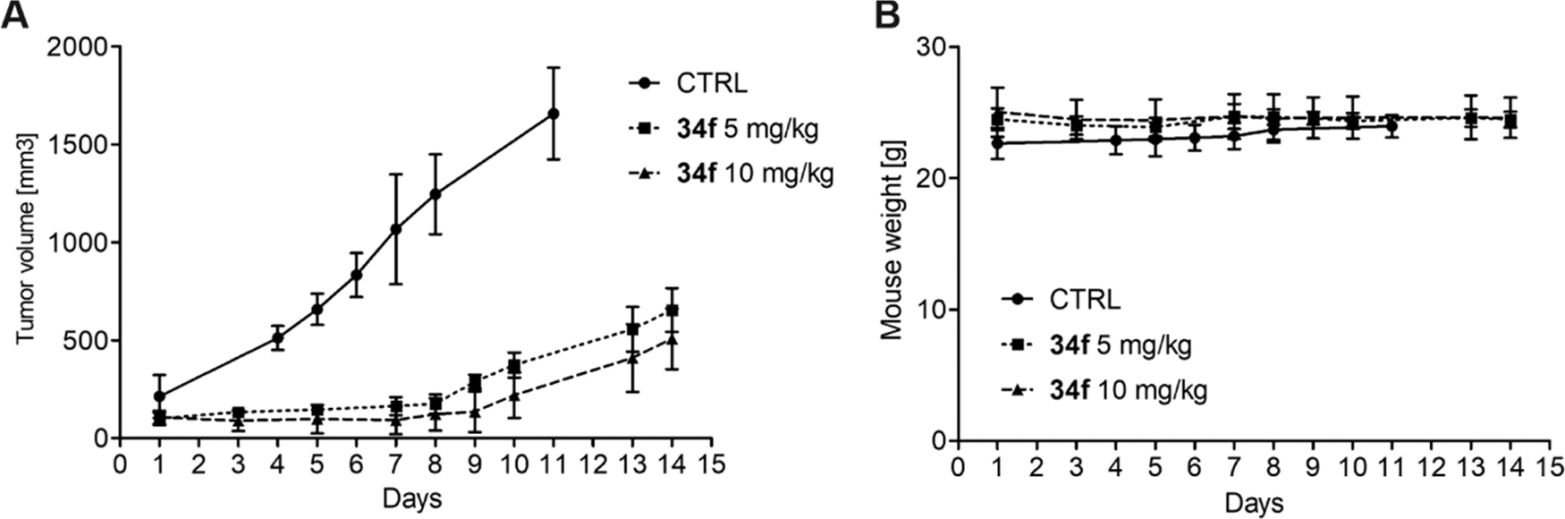
In vivo efficacy of **34f**. (A) Growth of subcutaneous MV4-11 xenografts (mean volume ± SD) in groups of mice treated with **34f** (5 and 10 mg/kg) or a vehicle only every other day until day 7 (4 doses) by intraperitoneal administration. (B) The weight of mice (mean ± SD) during the experiment.

Encouraged by these results, we performed in vivo experiments on immunodeficient mice bearing subcutaneous MV4-11 xenografts, the widely accepted simple in vivo model. As shown in Figure 8A, tumor growth was blocked in groups of mice treated repeatedly with intraperitoneal injections of **34f** at doses of 5 and 10 mg/kg. By the end of the drug administration (day 7), the tumor growth rate remained restricted. On the other hand, our vehicle-treated control group of mice exhibited a steep increase in tumor size. For this reason, the experiment in this cohort had to be terminated prematurely. In addition to displaying strong anticancer efficacy in vivo, **34f** administration had no adverse effect on mouse weight during the experiment (Figure 8B).

**Figure 9:**
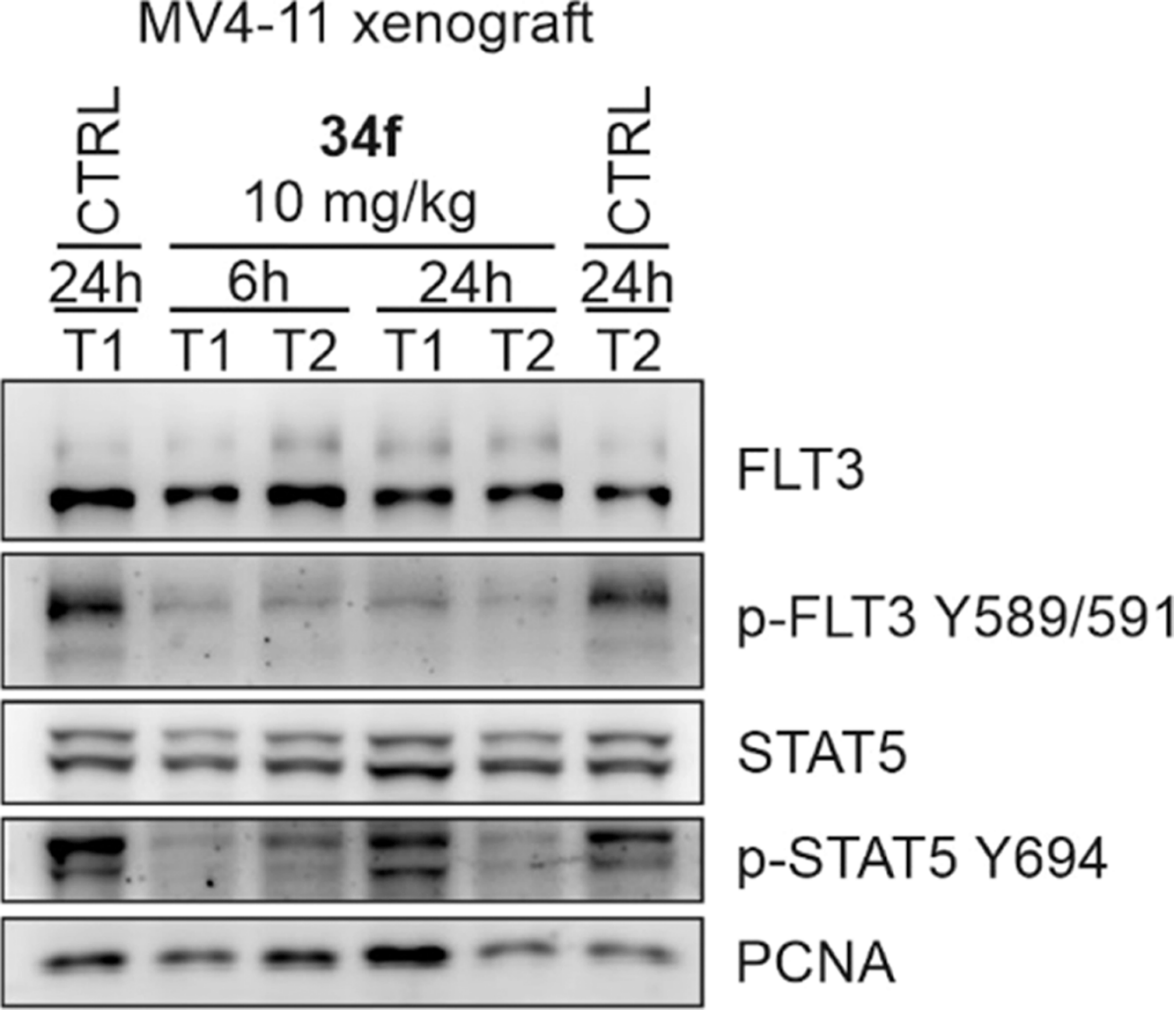
Immunoblotting analysis of MV4-11 subcutaneous xenografts exposed to **34f** for 6 or 24 h.

Moreover, immunoblotting analysis of MV4-11 xenografts exposed for 6 or 24 h to **34f** at a dose of 10 mg/kg revealed reduced phosphorylation of FLT3 at Y589/591 and of STAT5 at Y694 in most of the analyzed tumors in comparison with vehicle-treated mice, thus confirming the FLT3-dependent mechanism of action of **34f** in vivo (Figure 9).

In addition, the pharmacokinetic properties of **34f** were determined in mice following intraperitoneal administration at a dose of 10 mg/kg. The results showed that **34f** has a half-life of 71.3 min, including the absorption and elimination phases. The compound reaches a maximal plasma concentration of 384 pg/mL (722 nmol/L) after approximately 49 min following administration. For the details, see the Supporting information.

## Conclusions

In this study, we investigated the suitability of several series of heterocyclic derivatives as potential FLT3 kinase inhibitors. Compounds derived from the imidazo[1,2-*b*]pyridazine heterocyclic core proved to be potent inhibitors of FLT3 kinase, and modification of position 3 resulted in a pronounced effect on activity and selectivity in comparison with CDK2. In the extensive structure–activity relationship (SAR), the 3-phenyl substituent and some of its derivatives (e.g., 3- or 4-methoxyphenyl, 4-fluorophenyl, or 4-(trifluoromethyl)phenyl) displayed activity toward FLT3 within a single-digit nanomolar range, where the selectivity ratio for CDK2/FLT3-ITD was more than 200. Candidate compound **34f** showed high antiproliferative efficacy in the FLT3-ITD-positive AML cell lines MV4-11 and MOLM-13 (7 and 9 nM, respectively) as well as in the MOLM-13 variant bearing the FLT3-ITD-D835Y mutation (4 nM) in comparison with low sensitivity of FLT3-independent cell lines, proving the FLT3-dependent mechanism of action. Immunoblotting and flow cytometry analysis confirmed the blocking of signaling pathways subordinate to FLT3 as well as induced G1 arrest of FLT3-dependent MV4-11 AML cells. As the derivative **34f** showed sufficient plasma and microsomal stability, we continued with in vivo experiments in immunodeficient mice bearing subcutaneous MV4-11 xenografts. We observed a strong effect of **34f** on tumor growth without any side effects on mouse weight. Additionally, immunoblotting analysis of MV4-11 xenografts confirmed reduced phosphorylation of FLT3 at Y589/591 and of STAT5 at Y694 in the analyzed tumors, confirming the FLT3-dependent mode of action in vivo. In summary, we found a novel substitution pattern of imidazo[1,2-*b*]pyridazine that shows excellent potency toward FLT3 kinase in vitro and in vivo without any pronounced side effects. The activity displayed by this series of compounds, mainly the derivatives **34f**, **34g**, **34h**, **34i**, **34j**, **34l**, **34n**, and **34m**, indicates their suitability for further development as potential AML drug candidates.

## Experimental Section

Starting compounds and reagents were purchased from commercial suppliers (Sigma-Aldrich, Fluorochem, Acros Organics, Carbosynth, TCI) and used without further purification. Dry tetrahydrofuran was distilled with lithium aluminum hydride pellets under an argon atmosphere. Analytical thin-layer chromatography (TLC) was performed on silica gel pre-coated aluminum plates with a fluorescent indicator (Merck 60 F_254_). Flash column chromatography was carried out using Teledyne ISCO CombiFlash Nextgen. Preparative HPLC purification was performed on the INGOS HPLC system (LCD5000 and LCP5020 modules, chromatography column: Luna 5 μm C18(2) 100 Å). Mass spectra, UV absorbency, and purity of compounds were measured on the Waters UPLC-MS system, consisting of the Waters UPLC H-Class Core System (Waters Acquity UPLC BEH C18 1.7 mm column, 2.1 mm × 100 mm), the Waters Acquity UPLC PDA detector, and the Waters SQD2 mass spectrometer. The universal LC method was used (eluent H_2_O/CH_3_CN, gradient 0–100%, run length 4 min or 7 min) in conjunction with the MS method (ESI+ and/or ESI–, cone voltage = 30 V, mass detector range 100–1000 Da for standard cases and 500–1600 Da for larger molecules). High-resolution mass spectra were measured on the LTQ Orbitrap XL spectrometer (Thermo Fisher Scientific). NMR spectra were obtained using the Bruker Avance III HD 500 MHz spectrometer operating at 125.7 MHz for ^13^C and 500 MHz for ^1^H. The spectra were referenced to solvent residual signals (dimethyl sulfoxide (DMSO): 2.50 for ^1^H and 39.70 for ^13^C, CDCl_3_: 7.26 for ^1^H and 77.16 for ^13^C). The assignment of hydrogen and carbon spectra was based on a combination of one-dimensional (1D) and two-dimensional (2D) experiments (^1^H–^13^C APT, ^1^H–^1^H COSY, ^1^H–^13^C HSQC, and ^1^H–^13^C HMBC). The purity of the final compounds was determined by ultra-performance liquid chromatography–mass spectrometry (UPLC-MS) and was 95% or higher, with the exception of compounds **5b**, **10**, **25**, **34n**, and **39** due to the problematic separation of highly polar compounds; nevertheless, the purity was still higher than 90%.

### Molecular Docking

A docking study was performed using Schrödinger built-in modules. The homology model of the active DFG-in conformation of FLT3 (based on the crystal structure of FLT3 kinase,^39^ PDB 1RJB, resolution 2.10 Å) was used.^12^ The structure was optimized prior to docking using the Schrödinger Protein Preparation Wizard Maestro Suite (version 12.9.123, release 2021-3). Inconsistencies in the structure, such as missing hydrogens, incorrect bond orders, and poor orientation of amino-acid side chains, were rectified during the optimization process. The LigPrep module was used to convert two-dimensional structures to three-dimensional (3D), correct improper bond distances and bond orders, ionize compounds to correspond with pH 7 ± 1, and minimize ligand energy. Structures generated by LigPrep were then used for ligand docking. Ligand docking was performed using the Schrödinger Grid-based Ligand Docking with Energetics (Glide) Suite 2021 application. Receptor grid generation was based on the ligand from the original PDB structure. The default selection of 20 poses per ligand was set for Glide. Extra precision (XP) mode was selected for the Glide redocking stage.

#### General Procedure 1 (**GP1**): Reaction with 4-(1-Pyrrolidinylsulfonyl)aniline (**2**)

The heterocyclic derivative (1 mmol) and aniline (**2**) (1.25 mmol) in DMF (5 mL) were treated dropwise with *t*-BuOK (1 M in THF, 2.5 mL, 2.5 mmol) at 0 °C; the resulting mixture was stirred at the same temperature for 30 min. The mixture was diluted with EtOAc, washed with saturated NH_4_Cl and water, dried over MgSO_4_, and evaporated. The residue was purified by RP FC (H_2_O/ACN) and dried.

#### General Procedure 2 (**GP2**): Reaction with *Trans*-1,4-diaminocyclohexane

The heterocyclic derivative (1 mmol) and *trans*-1,4-diaminocyclohexane (10 mmol) in NMP (2.5 mL) were heated in a tightly sealed 4 mL vial at 210 °C overnight. The mixture was diluted with DMSO (2 mL) and directly applied to the RP FC (H_2_O/ACN + 0.1% of formic acid). Products containing fractions were evaporated and codistilled with water; the final compound was dried in vacuo or freeze-dried from dioxane.

#### General Procedure 3 (**GP3**): Suzuki Cross-Coupling with Boronic Acids

Heteroaryl bromide or iodide (1 equiv), boronic acid (1.2 equiv), Cs_2_CO_3_ (3 equiv), and Pd(dppf)Cl_2_·DCM (0.1 equiv) in dioxane/water (9:1, 5 mL to 1 mmol of the aryl halogenide) under an argon atmosphere were heated at 95 °C for 4 to 12 h. After the solvent was evaporated, the residue was purified by FC (c-hexane/EtOAc + 10% of MeOH, 0–50%), repurified by RP FC (H_2_O/ACN + 0.1% formic acid), evaporated, and codistilled with water and EtOH.

#### 7-Bromo-2-chloro-*N*-[4-(1-pyrrolidinylsulfonyl)phenyl]thieno[3,2-*d*]pyrimidin-4-amine (**3**)

Prepared from 7-bromo-2,4-dichlorothieno[3,2-*d*]pyrimidine according to **GP1**. Pale brown solid, yield 54%. MS (ESI): *m*/*z* = 472.9 [M + H]^+^. ^1^H NMR (DMSO-*d*_6_): δ = 10.65 (s, 1H, NH); 8.54 (s, 1H, H-6); 8.08–8.02 (m, 2H, H-2′); 7.87–7.82 (m, 2H, H-3′); 3.16 (m, 4H, NCH_2_); 1.67 (m, 4H, CH_2_) ppm. ^13^C NMR (DMSO-*d*_6_): δ = 158.02 (C-7a); 156.41 (C-4); 155.65 (C-2); 142.42 (C-1′); 133.62 (C-6); 130.98 (C-4′); 128.35 (C-3′); 121.41 (C-2′); 115.06 (C-4a); 107.49 (C-7); 47.83 (N-CH_2_); 24.72 (CH_2_-pyrrol.) ppm. HRMS (ESI): *m*/*z* calculated for C_16_H_15_O_2_N_4_BrClS_2_ 472.95028, found 472.95051.

#### 2-Chloro-7-(cyclohex-1-en-1-yl)-*N*-[4-(1-pyrrolidinylsulfonyl)phenyl]thieno[3,2-*d*]pyrimidin-4-amine (**4a**) and 2,7-Bis-(Cyclohex-1-en-1-yl)-*N*-[4-(1-pyrrolidinylsulfonyl)phenyl]thieno[3,2-*d*]pyrimidin-4-amine (**4b**)

Compound **3** (500 mg, 1.05 mmol), 1-cyclohex-1-en-1-ylboronic acid (140 mg, 1.11 mmol), and Cs_2_CO_3_ (923 mg, 3.15 mmol) in degassed dioxane (18 mL) and water (2 mL) were treated with Pd(dppf)Cl_2_·CH_2_Cl_2_ (73 mg, 0.1 mmol) under an argon atmosphere and heated at 95 °C for 4 h. The solvent was evaporated, after which the residue was purified by FC (c-hex/EtOAc + 10% MeOH) and repurified by RP FC (H_2_O/ACN) to give **4a** (340 mg, 68%) and disubstituted product **4b** (60 mg, 10%).

**4a.** White solid. MS (ESI): *m*/*z* = 475.1 [M + H]^+^. ^1^H NMR (DMSO-*d*_6_): δ = 10.39 (s, 1H, NH); 8.09 (s, 1H, H-6); 8.05 (d, 2H, *J* = 8.7 Hz, H-2′); 7.82 (d, 2H, *J* = 8.7 Hz, H-3′); 7.05 (s, 1H, H-2″); 3.17 (m, 4H, NCH_2_); 2.45 (m, 2H, H-6″); 2.22 (m, 2H, H-3″); 1.74 (m, 2H, H-5″); 1.69–1.58 (m, 6H, CH_2_-pyrrol., H-4″) ppm. ^13^C NMR (DMSO-*d*_6_): δ = 159.31 (C-7a); 155.74 (C-4); 155.14 (C-2); 142.78 (C-1′); 136.22 (C-1″); 130.54 (C-4′); 130.04 (C-6); 128.33 (C-3′); 127.79 (C-2″); 121.19 (C-2′); 116.24 (C-4a); 47.86 (NCH_2_); 27.32 (C-6″); 25.17 (C-3″); 24.74 (CH_2_-pyrrol.); 22.41 (C-5″); 21.65 (C-4″) ppm. HRMS (ESI): *m*/*z* calculated for C_22_H_24_O_2_N_4_ClS_2_ 475.10237, found 475.10219.

**4b.** White solid. MS (ESI): *m*/*z* = 521.1 [M + H]^+^. ^1^H NMR (DMSO-*d*_6_): δ = 9.84 (s, 1H, NH); 8.20 (m, 2H, H-2′); 7.94 (s, 1H, H-6); 7.80 (m, 2H, H-3′); 7.38 (m, 1H, H-2″); 7.21 (m, 1H, H-2‴); 3.15 (m, 4H, NCH_2_); 2.58 (m, 2H, H-6‴); 2.50 (m, 2H, H-6″); 2.29 (m, 2H, H-3″); 2.24 (m, 2H, H-3‴); 1.72 (m, 4H, H-5″, H-5‴); 1.65 (m, 8H, CH_2_-pyrrol., H-4″, H-4‴) ppm. ^13^C NMR (DMSO-*d*_6_): δ = 160.62 (C-2); 158.81 (C-7a); 154.14 (C-4); 144.10 (C-1′); 136.70 (C-7); 136.10 (C-1″); 132.00 (C-6); 130.52 (C-1‴); 128.92 (C-4′); 128.23 (C-3′); 127.73 (C-2‴); 127.36 (C-2″); 120.04 (C-2′); 114.99 (C-4a); 47.79 (NCH2); 27.26 (C-6″); 25.61 (C-3″); 25.33 (C-6‴); 25.18 (C-3‴); 24.66 (CH2-pyrrol.); 22.49 (C-5″, C-5‴); 21.83 (C-4″); 21.79 (C-4‴) ppm. HRMS (ESI): *m*/*z* calculated for C_28_H_33_O_2_N_4_S_2_ 521.20394, found 521.20358.

#### *N*^2^-(6-Aminopyridin-3-yl)-7-(cyclohex-1-en-1-yl)-*N*^4^-[4-(1-pyrrolidinylsulfonyl)phenyl]thieno[3,2-*d*]pyrimidin-2,4-diamine (**5a**) and *N*^2^-(5-Aminopyridin-2-yl)-7-(cyclohex-1-en-1-yl)-*N*^4^-[4-(1-pyrrolidinylsulfonyl)phenyl]thieno[3,2-*d*]pyrimidin-2,4-diamine (**5b**)

Compound **4a** (300 mg, 0.63 mmol), 2,5-diaminopyridine hydrochloride (185 mg, 1 mmol), and Cs_2_CO_3_ (1012.5 mg, 3.15 mmol) in dry DMF were treated with the XPhos Pd G2 precatalyst (50 mg, 0.063 mmol) under an argon atmosphere; the resulting mixture was heated at 95 °C overnight. The mixture was diluted with EtOAc, washed with sat. NH_4_Cl, and dried over MgSO_4_. EtOAc was evaporated, and the residue was purified by FC (c-hex/EtOAc + 10% MeOH). Final separation by RP FC (H_2_O/ACN with 0.1% of formic acid) afforded **5a** (pale brown solid, 40 mg, yield 10%) and **5b** (pale brown solid, 87 mg, yield 25%).

**5a.** MS (ESI): *m*/*z* = 548.14 [M + H]^+^. ^1^H NMR (DMSO-*d*_6_): δ = 9.74 (bs, 1H, NH-1′); 8.87 (bs, 1H, NH-py); 8.18 (m, 3H, H-2′, H-6py); 7.84 (s, 1H, H-6); 7.81 (m, 1H, H-4py); 7.70 (d, 2H, *J* = 8.8 Hz, H-3′); 7.18 (m, 1H, H-2′); 6.52 (d, 1H, *J* = 8.9 Hz, H-3py); 3.14 (m, 4H, NCH_2_); 2.45 (m, 2H, H-6″); 2.22 (m, 2H, H-3″); 1.74 (m, 2H, H-5″); 1.65 (m, 6H, H-4″, CH_2_-pyrrol.) ppm. ^13^C NMR (DMSO-*d*_6_): δ = 159.95 (C-2py); 157.86 (C-2); 154.92 (C-7a); 144.16 (C-1′); 135.99 (C-1″); 138.38 (C-6py); 130.63 (C-7); 132.23 (C-4py); 127.74 (C-4′); 128.06 (C-3′); 127.69 (C-6); 127.46 (C-5py); 126.82 (C-2″); 120.32 (C-2′); 108.66 (C-4a); 107.89 (C-3py); 47.89 (NCH_2_); 27.32 (C-6″); 25.26 (C-3″); 24.71 (CH_2_-pyrrol.); 22.58 (C-5″); 21.82 (C-4″) ppm. HRMS (ESI): *m*/*z* calculated for C_27_H_30_O_2_N_7_S_2_ 548.18969, found 548.18942.

**5b.** MS (ESI): *m*/*z* = 548.16 [M + H]^+^. ^1^H NMR (DMSO-*d*_6_): δ = 11.38 (bs, 1H, NH-1′); 10.65 (bs, 1H, NH-py); 8.17 (s, 1H, H-6); 8.16 (m, 2H, H-2′); 7.83 (m, 2H, H-3′); 7.64 (d, 1H, *J* = 2.7 Hz, H-6py); 7.52 (dd, 1H, *J* = 9.1, 2.7 Hz, H-4py); 7.38 (d, 1H, *J* = 9.1 Hz, H-3py); 6.55 (m, 1H, H-2″); 3.17 (m, 4H, NCH_2_); 2.46 (m, 2H, H-6″); 2.35 (m, 2H, H-3″); 1.80 (m, 2H, H-5″); 1.73 (m, 2H, H-4″); 1.67 (m, 4H, CH_2_-pyrrol.) ppm. ^13^C NMR (DMSO-*d*_6_): δ = 155.44 (C-2); 153.53 (C-7a); 151.88 (C-4); 142.52 (C-1′); 141.57 (C-2py); 140.91 (C-5py); 134.74 (C-1″); 131.35 (C-6); 131.13 (C-4′); 130.40 (C-7); 130.21 (C-4py); 128.23 (C-3′); 127.48 (C-2″); 122.50 (C-6py); 122.09 (C-2′); 115.73 (C-3py); 111.64 (C-4a); 47.89 (NCH_2_); 28.08 (C-6″); 25.38 (C-3″); 24.77 (CH_2_-pyrrol.); 22.43 (C-5″); 21.63 (C-4″) ppm. HRMS (ESI): *m*/*z* calculated for C_27_H_30_O_2_N_7_S_2_ 548.18969, found 548.18945.

#### 3-Amino-4-(cyclohex-1-en-1-yl)pyrazole (**6**)

Compound **6** was prepared according to a previously described procedure.^28,29^ White solid, yield 23%. MS (ESI): *m*/*z* = 164.1 [M + H]^+^. ^1^H NMR (350 K, DMSO-*d*_6_): δ = 11.25 (bs, NH); 7.24 (s, 1H, H-3); 5.75 (m, 1H, H-2′); 4.27 (bs, 2H, NH_2_); 2.21 (m, 2H, H-6′); 2.12 (m, 2H, H-3′); 1.67 (m, 2H, H-5′); 1.58 (m, 2H, H-4′) ppm. ^13^C NMR (350 K, DMSO-*d*_6_): δ = 150.49 (C-5); 129.41 (C-1′); 125.95 (C-3); 118.98 (C-2′); 108.32 (C-4); 27.84 (C-6′); 24.89 (C-3′); 22.54 (C-5′); 21.86 (C-4′) ppm. HRMS (ESI): *m*/*z* calculated for C_9_H_14_N_3_ 164.11822, found 164.11821.

#### 3-(Cyclohex-1-en-1-yl)-5,7-dichloropyrazolo[1,5-*a*]pyrimidine (**8**)

Sodium (0.394 g, 17.15 mmol, 2.5 equiv) was dissolved in dry EtOH (17 mL), followed by the addition of **6** (1.12 g, 6.86 mmol, 1 equiv) and diethyl malonate (1.0 mL, 6.86 mmol, 1 equiv); the resulting mixture was refluxed for 8 h. The mixture was cooled to RT, and the precipitated solids were filtered, dissolved in water, and acidified with 1 M HCl to pH 2. Solids were then filtered off, washed with water, and dried in vacuo. The mother liquor was evaporated to half its volume and acidified with 1 M HCl. Next, a further portion of the white solid product was filtered, washed with water, and dried. White solid, yield 1.23 g (76%). MS (ESI): *m*/*z* = 232.2 [M + H]^+^. Next, the product was directly treated with POCl_3_ (5 mL) and *N*,*N*-dimethylaniline (0.74 mL, 5.8 mmol, 1.5 equiv). The mixture was heated at 80 °C for 5 h, cooled down to RT, and carefully poured over crushed ice under vigorous stirring. The product was extracted with DCM, which was then washed with water and dried over MgSO_4_. Purification by FC (c-hex/EtOAc + 10% MeOH, 0–20%) provided a yellow solid of **8** (0.60 g, 46%). MS (ESI): *m*/*z* = 268.08 [M + H]^+^. ^1^H NMR (CDCl_3_): δ = 8.17 (s, 1H, H-8); 6.92 (s, 1H, H-1); 6.64 (m, 1H, H-2′); 2.51 (m, 2H, H-6′); 2.26 (m, 2H, H-3′); 1.81 (m, 2H, H-5′); 1.69 (m, 2H, H-4′) ppm. ^13^C NMR (CDCl_3_): δ = 148.18 and 139.83 (C-5 and C-7); 143.60 (C-2); 127.15 (C-1′); 126.01 (C-2′); 114.69 (C-3); 108.40 (C-6); 27.57 (C-6′); 25.75 (C-3′); 22.86 (C-5′); 22.23 (C-4′) ppm. HRMS (ESI): *m*/*z* calculated for C_12_H_12_N_3_Cl_2_ 268.04028, found 268.04033.

#### 5-Chloro-3-(cyclohex-1-en-1-yl)-*N*^7^-(4-(1-pyrrolidinylsulfonyl)phenyl)pyrazolo[1,5-*a*]pyrimidin-7-amine (**9**)

Prepared from **8** according to **GP1**. White solid, yield 59%. MS (ESI): *m*/*z* = 458.03 [M + H]^+^. ^1^H NMR (CDCl_3_): δ = 8.36 (s, 1H, NH); 8.04 (s, 1H, H-2); 7.94 (d, 2H, *J* = 8.7 Hz, H-3″); 7.50 (d, 2H, *J* = 8.7 Hz, H-2″); 6.68 (m, 1H, H-2′); 6.48 (s, 1H, H-6); 3.30 (m, 4H, NCH_2_); 2.52 (m, 2H, H-6′); 2.26 (m, 2H, H-3′); 1.84–1.78 (m, 6H, H-5′, CH_2_-pyrrol.); 1.70 (m, 2H, H-4′) ppm. ^13^C NMR (CDCl_3_): δ = 150.90 (C-7); 143.61 (C-3a); 143.29 (C-5); 141.66 (C-2); 140.27 (C-1″); 134.55 (C-4″); 129.65 (C-3″); 127.54 (C-1′); 124.87 (C-2′); 122.23 (C-2″); 113.22 (C-3); 87.33 (C-6); 48.12 (NCH_2_); 27.63 (C-6′); 25.74 (C-3′); 25.46 (CH_2_-pyrrol.); 22.95 (C-5′); 22.34 (C-4′) ppm. HRMS (ESI): *m*/*z* calculated for C_22_H_25_O_2_N_5_ClS 458.14120, found 458.14112.

#### *N*^5^-(5-Aminopyridin-2-yl)-3-(cyclohex-1-en-1-yl)-*N*^7^-(4-(1-pyrrolidinylsulfonyl)phenyl)pyrazolo[1,5-*a*]pyrimidine-5,7-diamine (**10a**) and *N*^5^-(6-Aminopyridin-3-yl)-3-(cyclohex-1-en-1-yl)-*N*^7^-(4-(1-pyrrolidinylsulfonyl)phenyl)pyrazolo[1,5-*a*]pyrimidine-5,7-diamine (**10b**)

Compound **9** (450 mg, 0.98 mmol), 2,5-diaminopyridine (537 mg, 2.95 mmol), and Cs_2_CO_3_ (2.56 g, 7.8 mmol) in dry DMF (20 mL) under an argon atmosphere were treated with Pd_2_(dba)_3_ (90 mg, 0.098 mmol) and Xantphos (114 mg, 0.2 mmol); the resulting mixture was heated at 120 °C overnight. The mixture was diluted with EtOAc, washed with sat. NH_4_Cl, dried over MgSO_4_, and evaporated. After the residue was purified by FC (c-hex/EtOAc + 10% MeOH), isomers were separated by RP FC (H_2_O/ACN + 0.1% TFA), evaporated, codistilled with water, and freeze-dried from dioxane.

**10a.** Off-white foam, yield 126 mg (24%). MS (ESI): *m*/*z* = 531.50 [M + H]^+^. ^1^H NMR (DMSO-*d*_6_): δ = 9.85 (s, 1H, NH-7); 9.40 (s, 1H, NH-5); 7.99 (H-2); 7.97 (d, 1H, *J*(py-3,4) = 9.1 Hz, py-3); 7.82 (m, 2H, H-3″); 7.70–7.67 (m, 3H, H-2″, py-6); 7.00 (dd, *J*(py-4,3) = 8.9 Hz, *J*(py-4,6) = 2.9 Hz, py-4); 6.75 (s, 1H, H-6); 6.64 (m, 1H, H-2′); 4.93 (s, 2H, NH_2_); 3.17 (m, 4H, NCH_2_); 2.50 (m, 2H, H-6″); 2.22 (m, 2H, H-3″); 1.79–1.66 (m, 8H, H-5″, H-4″, CH_2_-pyrrol.) ppm. ^13^C NMR (DMSO-*d*_6_): δ = 153.73 (C-5); 144.42 (C-3a); 144.11 (py-5); 143.27 (C-1″); 143.11 (C-7); 140.31 (C-8); 140.25 (py-2); 133.52 (py-6); 130.75 (C-4″); 128.93 (C-3″); 128.76 (C-1′); 123.07 (py-4); 121.86 (C-2″); 120.17 (C-2′); 113.68 (py-3); 108.07 (C-3); 78.57 (C-6); 48.08 (NCH_2_); 27.23 (C-6′); 25.31 (C-3′); 24.96 (CH_2_-pyrrol.); 22.83 (C-5′); 22.36 (C-4′) ppm. HRMS (ESI): *m*/*z* calculated for C_27_H_31_O_2_N_8_S 531.22852, found 531.22812. Purity was 90% due to complications with purification.

**10b**. Off-white foam, yield 100 mg (19%). MS (ESI): *m*/*z* = 531.4 [M + H]^+^. ^1^H NMR (DMSO-*d*_6_): δ = 9.81 (s, 1H, NH-7); 8.96 (s, 1H, NH-5); 8.29 (d, 1H, *J*(py-6,4) = 2.7 Hz, py-6); 7.96 (s, 1H, H-2); 7.83 (m, 2H, H-3″); 7.76 (dd, 1H, *J*(py-4,3) = 8.8 Hz, *J*(py-4,6) = 2.7 Hz, py-4); 7.68 (m, 2H, H-2″); 6.58 (m, 1H, H-2′); 6.45 (d, 1H, *J*(py-3,4) = 8.9 Hz, py-3); 6.12 (s, 1H, H-6); 5.62 (bs, 2H, NH_2_); 3.17 (m, 4H, NCH_2_); 2.45 (m, 2H, H-6′); 2.19 (m, 2H, H-3′); 1.74–1.61 (m, 8H, H-5′, 4′, CH_2_-pyrrol.) ppm. ^13^C NMR (DMSO-*d*_6_): δ = 155.59 (py-2); 154.57 (C-5); 144.60 (C-3a); 143.40 (C-7); 143.12 (C-1″); 140.29 (C-2); 137.50 (C-6); 130.97 (C-4″); 130.56 (py-4); 129.04 (C-3″); 128.75 (C-1′); 127.58 (py-5); 122.11 (C-2″); 119.98 (C-2′); 107.91 (C-3); 107.69 (py-3); 77.87 (C-6); 48.07 (NCH_2_); 27.20 (C-6′); 25.36 (C-3′); 24.97 (CH_2_-pyrrol); 22.83 (C-5′); 22.36 (C-4′) ppm. HRMS (ESI): *m*/*z* calculated for C_27_H_31_O_2_N_8_S 531.22852, found 531.22831.

#### *N*^5^-((1*r*,4*r*)-4-Aminocyclohexyl)-3-(cyclohex-1-en-1-yl)-*N*^7^-(4-(pyrrolidin-1-ylsulfonyl)phenyl)pyrazolo[1,5-*a*]pyrimidine-5,7-diamine (**11**)

Prepared from **9** according to **GP2**. Brown solid, yield 68%. MS (ESI): *m*/*z* = 536.3 [M + H]^+^. ^1^H NMR (DMSO-*d*_6_): δ = 9.66 (bs, 1H, NH); 7.88 (s, 1H, H-2); 7.85 (bd, 2H, *J* = 5.4 Hz, NH_2_); 7.80 (m, 2H, H-3′); 7.63 (m, 2H, H-2′); 7.02 (bs, 1H, NH); 6.62 (m, 1H, H-2″); 5.96 (s, 1H, H-6); 3.75 (m, 1H, c-hex-1); 3.16 (m, 4H, NCH_2_); 3.04 (m, 1H, c-hex-4); 2.45 (m, 2H, H-6″); 2.17 (m, 2H, H-3″); 2.11 (m, 2H, c-hex-2a); 1.99 (m, 2H, c-hex-3a); 1.73–1.66 (m, 6H, H-5″, CH_2_-pyrrol.); 1.62 (m, 2H, H-4″); 1.42 (m, 2H, c-hex-3b); 1.23 (m, 2H, c-hex-2b) ppm. ^13^C NMR (DMSO-*d*_6_): δ = 156.18 (C-7); 143.04 (C-1′); 139.76 (C-2); 130.55 (C-4′); 128.78 (C-3′); 128.74 (C-1″); 121.63 (C-2′); 119.23 (C-2″); 106.78 (C-8); 76.73 (C-6); 48.76 and 48.60 (c-hex-1,4); 47.83 (NCH_2_); 29.76 and 29.19 (c-hex-2,3); 26.85 (C-6″); 25.12 (C-3″); 24.74 (CH2-pyrrol.); 22.65 (C-5″); 22.20 (C-4″) ppm.

#### 3-Bromo-5-chloro-*N*-[4-(1-pyrrolidinylsulfonyl)phenyl]pyrazolo[1,5-*a*]pyrimidine-7-amine (**13**)

Prepared from 3-bromo-5,7-dichloropyrazolo[1,5-*a*]pyrimidine^30^ according to **GP1**. Off-white solid, yield 85%. MS (ESI): *m*/*z* = 456.01 [M + H]^+^. ^1^H NMR (DMSO-*d*_6_): δ = 10.80 (bs, 1H, NH); 8.44 (s, 1H, H-2); 7.88 (m, 2H, H-3′); 7.72 (m, 2H, H-2′); 6.54 (s, 1H, H-6); 3.18 (m, 4H, NCH_2_); 1.69 (m, 4H, CH_2_-pyrrol.) ppm. ^13^C NMR (DMSO-*d*_6_): δ = 151.83 (C-7); 145.44 (C-5); 144.57 (C-3a); 144.26 (C-2); 140.92 (C-1′); 133.0 (C-4′); 128.91 (C-3′); 123.83 (C-2′); 88.02 (C-6); 82.07 (C-3); 47.84 (NCH_2_); 24.75 (CH_2_-pyrrol.) ppm. HRMS (ESI): *m*/*z* calculated for C_16_H_16_O_2_N_5_BrClS 455.98911, found 455.98889.

#### *N*^5^-((1*r*,4*r*)-4-Aminocyclohexyl)-*N*^7^-(4-(1-pyrrolidinylsulfonyl)phenyl)pyrazolo[1,5-*a*]pyrimidine-5,7-diamine (**14**)

Prepared from **13** according to **GP2**. Off-white foam, yield 41%. MS (ESI): *m*/*z* = 456.34 [M + H]^+^. ^1^H NMR (DMSO-*d*_6_): δ = 10.23 (bs, NH-1′); 7.99 (d, 1H, *J*(2,3) = 2.1 Hz, H-2); 7.94 (m, 3H, NH, NH_2_); 7.86 (d, 2H, *J*(3′,2′) = 8.7 Hz, H-3′); 7.66 (d, 2H, *J*(2′,3′) = 8.7 Hz, H-2′); 6.17 (d, 1H, *J*(3,2) = 2.1 Hz, H-3); 5.89 (s, 1H, H-6); 3.69 (m, 1H, N-CH-1″); 3.17 (m, 4H, NCH_2_); 3.04 (m, 1H, N-CH-4″); 2.03–1.95 (m, 4H, H-2″a, H-3″a); 1.69 (m, 4H, CH_2_-pyrrol.); 1.43 (m, 2H, H-3″b); 1.28 (m, 2H, H-2″b) ppm. ^13^C NMR (DMSO-*d*_6_): δ = 158.52 (C-7); 158.19 (C-5); 154.33 (C-9); 143.35 (C-2); 141.97 (C-1′); 132.05 (C-4′); 128.85 (C-3′); 123.20 (C-2′); 91.28 (C-2); 75.08 (C-6); 48.42 (N-CH-1′, 4′); 47.85 (NCH_2_); 29.87 and 28.94 (C-2″, 3″) ppm. HRMS (ESI): *m*/*z* calculated for C_22_H_30_O_2_N_7_S 456.21762, found 456.21743.

#### 5,7-Dichloro-3-cyclohexyl-3*H*-imidazo[4,5-*b*]pyridine (**16**)

5,7-Dichloro-3*H*-imidazo[4,5-*b*]pyridine (0.9 g, 4.8 mmol) was codistilled with toluene and dry dioxane. Cyclohexanol (4.9 mL, 47.8 mmol) and Ph_3_P (3.77 g, 14.36 mmol) were added, and the mixture was then flushed with argon. Dry degassed dioxane (24 mL) was added together with DIAD (2.8 mL, 14.36 mmol) in a dropwise manner; the resulting mixture was stirred at RT overnight. The solvent was evaporated, the product was isolated by FC (c-hex/EtOAc + 10% MeOH), and the final compound was repurified by RP FC (H_2_O/ACN). White solid, 490 mg (38%). MS (ESI): *m*/*z* = 270.1 [M + H]^+^. ^1^H NMR (DMSO-*d*_6_): δ = 8.71 (s, 1H, H-8); 7.61 (s, 1H, H-1); 4.45 (tt, 1H, *J* = 11.8, 3.8 Hz, H-1′); 2.03 (m, 2H, H-2′a); 1.93–1.79 (m, 4H, H-2′b, H-3′a); 1.71 (m, 1H, H-4′a); 1.47 (m, 2H, H-3′b); 1.27 (m, 1H, H-4′b) ppm. ^13^C NMR (DMSO-*d*_6_): δ = 146.01 (C-4); 144.83 (C-8); 143.89 (C-2); 135.05 (C-6); 131.93 (C-5); 117.76 (C-1); 54.15 (C-1′); 32.17 (C-2′); 25.02 (C-3′); 24.75 (C-4′) ppm. HRMS (ESI): *m*/*z* calculated for C_12_H_14_N_3_Cl_2_ 270.05593, found 270.05579.

#### 5-Chloro-3-cyclohexyl-*N*-(4-(pyrrolidin-1-ylsulfonyl)phenyl)-3*H*-imidazo[4,5-*b*]pyridin-7-amine (**17**)

Prepared according to **GP1**. White solid, yield 80%. MS (ESI): *m*/*z* = 460.1 [M + H]^+^. ^1^H NMR (DMSO-*d*_6_): δ = 9.78 (s, 1H, NH); 8.44 (s, 1H, H-8); 7.75 (d, 2H, *J* = 8.8 Hz, H-3′); 7.56 (d, 2H, *J* = 8.8 Hz, H-2′); 6.98 (s, 1H, H-1); 4.40 (tt, 1H, *J* = 12.2, 4.0 Hz, H-1″); 3.14 (m, 2H, NCH_2_); 2.03 (m, 2H, H-2″a); 1.84–1.94 (m, 4H, H-2″b, 3″a); 1.66–1.73 (m, 5H, H-4″b, CH_2_-pyrrol.); 1.47 (m, 2H, H-3″b); 1.26 (m, 1H, H-4″a) ppm. ^13^C NMR (DMSO-*d*_6_): δ = 146.11 (C-4); 145.36 (C-2); 144.49 (C-1′); 142.69 (C-6); 140.42 (C-8); 129.26 (C-4′); 128.81 (C-3′); 123.94 (C-5); 119.74 (C-2′); 101.31 (C-1); 53.48 (C-1″); 47.78 (NCH_2_); 32.38 (C-2″); 25.11 (C-3″); 24.82 (C-4″); 24.68 (CH_2_-pyrrol.) ppm. HRMS (ESI): *m*/*z* calculated for C_22_H_27_O_2_N_5_ClS 460.15685, found 460.15658.

#### *N*^5^-(5-Aminopyridin-2-yl)-3-cyclohexyl-*N*^7^-(4-(pyrrolidin-1-ylsulfonyl)phenyl)-3*H*-imidazo[4,5-*b*]pyridine-5,7-diamine (**18a**) and *N*^5^-(6-Aminopyridin-3-yl)-3-cyclohexyl-*N*^7^-(4-(pyrrolidin-1-ylsulfonyl)phenyl)-3*H*-imidazo[4,5-*b*]pyridine-5,7-diamine (**18b**)

Compound **17** (250 mg, 0.54 mmol), 2,5-diaminopyridine (297 mg, 1.63 mmol), and Cs_2_CO_3_ (1.42 g, 4.35 mmol) in dry DMF (12 mL) under an argon atmosphere were treated with Pd_2_(dba)_3_ (50 mg, 0.054 mmol) and Xantphos (63 mg, 0.11 mmol); the resulting mixture was then heated at 115 °C overnight. The mixture was diluted with EtOAc, washed with sat. NH_4_Cl, dried over MgSO_4_, and evaporated. After the residue was purified by FC (c-hex/EtOAc + 10% MeOH), isomers were separated by RP FC (H_2_O/ACN + 0.1% TFA), evaporated, codistilled with water, and freeze-dried from dioxane.

**18a.** Pale brown foam, yield 47 mg (16%). MS (ESI): *m*/*z* = 533.2 [M + H]^+^. ^1^H NMR (DMSO-*d*_6_): δ = 10.97 (s, 1H, NH); 9.76 (s, 1H, NH); 8.38 (s, 1H, H-8); 7.78 (3, 2H, H-3′); 7.70 (d, 1H, *J* = 2.7 Hz, py-6); 7.66 (dd, 1H, *J* = 9.27, 2.7 Hz, py-4); 7.59 (m, 2H, H-2′); 7.34 (d, 1H, *J* = 9.4 Hz, py-3); 6.85 (s, 1H, H-1); 4.72 (m, 1H, H-1″); 3.16 (m, 2H, NCH_2_); 2.12 (m, 2H, H-2″a); 1.87–1.92 (m, 4H, H-2″b, 3″a); 1.78 (m, 1H, H-4″b); 1.59 (m, 4H, CH_2_-pyrrol.); 1.56 (m, 2H, H-3″b); 1.29 (m, 1H, H-4″a) ppm. ^13^C NMR (DMSO-*d*_6_): δ = 150.57 (C-2); 144.73 (C-1′); 143.62 (C-4); 142.86 (C-6); 138.34 (C-8); 133.50 (py-4); 129.23 (C-4′); 129.14 (py-6); 128.87 (C-3′); 121.30 (C-5); 119.91 (C-2′); 115.63 (py-3); 89.99 (C-1); 53.32 (C-1″); 47.82 (NCH_2_); 32.45 (C-2″); 25.08 (C-3″); 24.73 (C-4″, CH_2_-pyrrol.) ppm. HRMS (ESI): *m*/*z* calculated for C_27_H_33_O_2_N_8_S 533.24417, found 533.24380.

**18b.** Pale brown foam, yield 40 mg (14%). MS (ESI): *m*/*z* = 533.2 [M + H]^+^. ^1^H NMR (DMSO-*d*_6_): δ = 9.46 (s, 1H, NH); 9.33 (s, 1H, NH); 8.83 (d, 1H, *J* = 2.5 Hz, py-6); 8.34 (s, 1H, H-8); 7.90 (dd, 1H, *J* = 9.5, 2.5 Hz, py-4); 7.75 (d, 2H, *J* = 8.8 Hz, H-3′); 7.68 (bs, 2H, NH_2_); 7.53 (d, 2H, *J* = 8.8 Hz, H-2′); 7.03 (d, 1H, *J* = 9.5 Hz, py-3); 6.65 (s, 1H, H-1); 4.41 (m, 1H, H-1″); 3.14 (m, 2H, NCH_2_); 2.08 (m, 2H, H-2″a); 1.86–1.95 (m, 4H, H-2″b, 3″a); 1.67–1.75 (m, 5H, H-4″b, CH_2_-pyrrol.); 1.52 (m, 2H, H-3″b); 1.32 (m, 1H, H-4″a) ppm. ^13^C NMR (DMSO-*d*_6_): δ = 152.71 (C-2); 149.42 (py-2); 145.34 (C-1′); 144.88 (C-4); 141.41 (C-6); 137.33 (py-4); 137.10 (C-8); 129.36 (py-5); 128.92 (C-3′); 128.35 (C-4′); 120.67 (py-4); 119.00 (C-2′); 118.78 (C-5); 114.06 (py-3); 90.10 (C-1); 54.03 (C-1″); 47.81 (NCH_2_); 32.21 (C-2″); 25.17 (C-3″); 24.95 (C-4″); 24.71 (CH_2_-pyrrol.) ppm. HRMS (ESI): *m*/*z* calculated for C_27_H_33_O_2_N_8_S 533.24417, found 533.24380.

#### 8-Bromo-2-((4-nitrophenyl)amino)pyrido[4,3-*d*]pyrimidin-4(3*H*)-one (**21**)

Compound **20** was prepared as previously described.^33^ In freshly distilled THF (100 mL), **20** (6.2 g, 13 mmol) was treated with 4-nitrophenyl isocyanate (4.14 g, 25 mmol) under an argon atmosphere at RT. The mixture was stirred at RT for 2 h (TLC showed complete consumption of the starting material) and diluted with 100 mL of THF. Next, NH_3_ was bubbled through the mixture for 2 min, and the resulting mixture was stirred at RT overnight. Poorly soluble solids were filtered, washed with THF, and dried. Bright yellow powder, yield 3.1 g (68%). MS (ESI): *m*/*z* = 362 [M + H]^+^. ^1^H NMR (DMSO-*d*_6_, 353 K): δ = 8.97 (s, 1H, H-7); 8.73 (s, 1H, H-5); 8.28 (m, 2H, H-2′); 8.20 (m, 2H, H-3′) ppm. ^13^C NMR (DMSO-*d*_6_, 353 K): δ = 165.55 (C-4); 155.35 (C-2); 153.56 (C-8a); 152.37 (C-5); 147.89 (C-7); 146.20 (C-1′); 141.12 (C-4′); 124.45 (C-3′); 118.81 (C-2′); 116.57 and 116.45 (C-8 and C-4a) ppm.

#### 8-Bromo-*N*^2^-(4-nitrophenyl)-*N*^4^-(4-(1-pyrrolidinylsulfonyl)phenyl)pyrido[4,3-*d*]pyrimidine-2,4-diamine (**23**)

Compound **20** (3 g, 8 mmol) in POCl_3_ (75 mL) was treated with *N*,*N*-dimethylaniline (2.1 mL, 17 mmol). The reaction mixture was refluxed for 20 h, cooled to RT, and poured slowly over ice. The solids were filtered and washed with water. Red solid, yield 2.8 g (89%). MS (ESI): *m*/*z* = 379.9 [M + H]^+^. The chloro derivative was converted to **23** according to **GP1**. Dark red solid, yield 32%. MS (ESI): *m*/*z* = 570.0 [M + H]^+^. ^1^H NMR (DMSO-*d*_6_): δ = 10.52 (bs, 1H, NH-2); 10.48 (bs, 1H, NH-4); 9.62 (s, 1H, H-5); 8.93 (s, 1H, H-7); 8.21–8.30 (m, 6H, H-2′, H-2″, H-3″); 7.87 (d, 2H, *J* = 8.8 Hz, H-3′); 3.19 (m, 4H, NCH2); 1.68 (m, 4H, CH_2_-pyrrol.) ppm. ^13^C NMR (DMSO-*d*_6_): δ = 158.58 (C-2); 158.14 (C-4); 153.20 (C-8a); 151.46 (C-7); 146.38 (C-1″); 146.36 (C-5); 142.62 (C-1′); 141.20 (C-4″); 128.15 (C-3′); 124.72 (C-3″); 122.26 (C-2′); 119.08 (C-2″); 117.43 (C-8); 110.45 (C-4a); 47.85 (NCH_2_); 24.74 (CH_2_-pyrrol.) ppm. HRMS (ESI): *m*/*z* calculated for C_23_H_21_O_4_N_7_BrS 570.05536, found 570.05564.

#### 8-(1-Cyclohexen-1-yl)-*N*^2^-(4-nitrophenyl)-*N*^4^-(4-(1-pyrrolidinylsulfonyl)phenyl)pyrido[4,3-*d*]pyrimidine-2,4-diamine (**24**)

Compound **23** (170 mg, 0.3 mmol), cyclohex-1-en-1-ylboronic acid (113 mg, 0.9 mmol), Cs_2_CO_3_ (583 mg, 1.8 mmol), and Pd(dppf)Cl_2_·DCM (49 mg, 0.06 mmol) in DMF (13 mL) and water (2 mL) under an argon atmosphere were heated at 80 °C overnight. The mixture was diluted with EtOAc, washed with sat. NH_4_Cl, and dried over MgSO_4_. Purification by RP FC (H_2_O/ACN + 0.1% TFA) afforded a yellow solid (160 mg, 94%). MS (ESI): *m*/*z* = 572.2 [M + H]^+^. ^1^H NMR (DMSO-*d*_6_): δ = 10.33 (bs, 1H, NH); 10.26 (bs, 1H, NH); 9.58 (s, 1H, H-5); 8.44 (s, 1H, H-7); 8.30 (d, 2H, *J* = 8.2 Hz, H-2′); 8.23 (d, 2H, *J* = 9.1 Hz, H-2″); 8.14 (d, 2H, *J* = 8.9 Hz, H-3″); 7.84 (d, 2H, *J* = 8.2 Hz, H-3′); 5.89 (m, 1H, H-2‴); 3.18 (m, 4H, NCH_2_); 2.50 (m, 2H, H-6‴); 2.26 (m, 2H, H-3‴); 1.82 (m, 2H, H-5‴); 1.76 (m, 2H, H-4‴); 1.68 (m, 4H, CH_2_-pyrrol.) ppm. ^13^C NMR (DMSO-*d*_6_): δ = 158.62 (C-2); 156.86 (C-4); 153.18 (C-8a); 148.91 (C-7); 146.94 (C-1″); 146.31 (C-5); 143.07 (C-1′); 140.77 (C-4″); 134.61 (C-8); 130.50 (C-1‴); 128.14 (C-3′); 127.83 (C-2‴); 124.58 (C-3″); 121.83 (C-2′); 118.42 (C-2″); 108.56 (C-4a); 47.85 (NCH_2_); 28.79 (C-6‴); 25.35 (C-3‴); 24.74 (CH_2_-pyrrol.); 22.74 (C-5‴); 21.90 (C-4‴) ppm. HRMS (ESI): *m*/*z* calculated for C_29_H_30_O_4_N_7_S 572.20745, found 572.20707.

#### 8-(1-Cyclohexen-1-yl)-*N*^2^-(4-aminophenyl)-*N*^4^-(4-(1-pyrrolidinylsulfonyl)phenyl)pyrido[4,3-*d*]pyrimidine-2,4-diamine (**25**)

Compound **24** (60 mg, 0.1 mmol) in EtOAc (5 mL) and EtOH (1 mL) was treated with Pd/C (10% wt, 14 mg) and stirred under a hydrogen atmosphere (15 bar) for 2 days. The mixture was filtered, the solvent was evaporated, and the residue was purified by RP FC (H_2_O/ACN + 0.1% TFA) to give an off-white solid (35 mg, 58%). MS (ESI): *m*/*z* = 542.2 [M + H]^+^. ^1^H NMR (DMSO-*d*_6_): δ = 10.55 (bs, 1H, NH); 10.25 (bs, 1H, NH); 9.68 (s, 1H, H-5); 8.42 (s, 1H, H-7); 8.34 (d, 2H, *J* = 8.2 Hz, H-2′); 7.93 (d, 2H, *J* = 9.1 Hz, H-2″); 7.83 (m, 2H, H-3″); 7.11 (m, 2H, H-3′); 6.01 (m, 1H, H-2‴); 3.17 (m, 4H, NCH_2_); 2.50 (m, 2H, H-6‴); 2.25 (m, 2H, H-3‴); 1.79–1.71 (m, 4H, H-5‴, H-4‴); 1.67 (m, 4H, CH_2_-pyrrol.) ppm. ^13^C NMR (DMSO-*d*_6_): δ = 158.37 (C-2); 158.05 (C-4); 157.21 (C-8a); 140.91 (C-5); 139.61 (C-7); 133.27 (C-8); 128.92 (C-2‴); 127.95 (C-3″); 121.47 (C-2′); 121.15 (C-2″); 120.18 (C-3′); 115.36 (C-4a); 47.87 (NCH_2_); 28.06 (C-6‴); 25.29 (C-3‴); 24.74 (CH_2_-pyrrol.); 22.48 (C-5‴); 21.65 (C-4‴) ppm. HRMS (ESI): *m*/*z* calculated for C_29_H_32_O_2_N_7_S 542.23327, found 542.23275. Purity was 93% due to complications with purification.

#### 3-Bromo-6-chloro-*N*-(4-(1-pyrrolidinylsulfonyl)phenyl)imidazo[1,2-*b*]pyridazin-8-amine (**27**)

Prepared according to **GP1** from 3-bromo-6,8-dichloroimidazo[1,2-*b*]pyridazine.^40,41^ White solid, yield 52%. MS (ESI): *m*/*z* = 455.9 [M + H]^+^. ^1^H NMR (DMSO-*d*_6_): δ = 10.34 (bs, 1H, NH); 7.83 (m, 3H, H-2, H-3′); 7.70 (m, 2H, H-2′); 6.84 (s, 1H, H-7); 3.16 (m, 4H, NCH_2_); 1.68 (m, 4H, CH_2_-pyrrol.) ppm. ^13^C NMR (DMSO-*d*_6_): δ = 148.54 (C-6); 142.63 (C-1′); 139.75 (C-8); 133.16 (C-9); 131.64 (C-2); 131.49 (C-4′); 128.88 (C-3′); 122.01 (C-2′); 101.16 (C-3); 95.23 (C-7); 47.81 (NCH_2_); 24.72 (CH_2_-pyrrol.) ppm. HRMS (ESI): *m*/*z* calculated for C_16_H_16_O_2_N_5_BrClS 455.98911, found 455.98873.

#### *N*^6^-((1*r*,4*r*)-4-Aminocyclohexyl)-3-bromo-*N*^8^-(4-(1-pyrrolidinylsulfonyl)phenyl)imidazo[1,2-*b*]pyridazine-6,8-diamine (**29a**) and *N*^6^-((1*r*,4*r*)-4-Aminocyclohexyl)-3-(1-cyclohexene-1-yl)-*N*^8^-(4-(1-pyrrolidinylsulfonyl)phenyl)imidazo[1,2-*b*]pyridazine-6,8-diamine (**29b**)

Compound **27** (520 mg, 1.14 mmol), cyclohex-1-en-1-ylboronic acid (158 mg, 1.25 mmol), and XPhos Pd G2 (45 mg, 0.057 mmol) in degassed dioxane (18 mL) were treated with an aqueous solution of K_3_PO_4_ (0.5 M, 4.56 mL, 2.28 mmol) under an argon atmosphere and heated at 95 °C overnight. After the solvent was evaporated, the residue was purified by FC (c-hex/EtOAc + 10% MeOH) and repurified by RP FC (H_2_O/ACN) to give an inseparable mixture of starting material and compound **28** in a ratio of approximately 5:3, 490 mg. MS (ESI): *m*/*z* = 458.4 [M + H]^+^. The resulting mixture (310 mg) was converted according to **GP2** to the corresponding aminocyclohexyl derivatives **29a** and **29b**, which were finally separated by RP FC (H_2_O/ACN + 0.1% formic acid).

**29a**. Off-white solid, 10 mg. MS (ESI): *m*/*z* = 534.3 [M + H]^+^. ^1^H NMR (DMSO-*d*_6_): δ = 9.48 (s, 1H, NH); 7.83 (d, 2H, *J* = 5.4 Hz, NH_2_); 7.77 (m, 2H, H-3′); 7.59 (m, 2H, H-2′); 7.47 (s, 1H, H-2); 6.66 (d, 1H, *J* = 7.0 Hz, NH); 6.45 (s, 1H, H-7); 3.57 (m, 1H, H-1″); 3.15 (m, 4H, NCH_2_); 3.04 (m, 1H, H-4″); 2.15 (m, 2H, H-2″a); 1.99 (m, 2H, H-3″a); 1.67 (m, 4H, CH_2_-pyrrol.); 1.43 (m, 2H, H-3″b); 1.25 (m, 2H, H-2″b) ppm. ^13^C NMR (DMSO-*d*_6_): δ = 154.83 (C-6); 144.41 (C-1′); 137.03 (C-8); 132.27 (C-9); 129.39 (C-4′); 128.7 (C-3′, C-2); 120.26 (C-2′); 99.62 (C-3); 88.36 (C-7); 48.71 and 48.64 (C-1″, C-4″); 47.78 (NCH_2_); 29.69 (C-2″); 29.15 (C-3″); 24.70 (CH_2_-pyrrol.) ppm. HRMS (ESI): *m*/*z* calculated for C_22_H_29_O_2_N_7_BrS 534.12813, found 534.12767.

#### **29b**·HCOOH

Off-white solid, 30 mg. MS (ESI): *m*/*z* = 536.2 [M + H]^+^. ^1^H NMR (DMSO-*d*_6_): δ = 8.45 (s, 1H, HCOOH); 7.75 (m, 2H, H-3′); 7.59 (m, 2H, H-2′); 7.35 (s, 1H, H-2); 7.17 (m, 1H, H-2-c-hex); 6.52 (d, 1H, *J* = 6.5 Hz, NH); 6.42 (s, 1H, H-7); 3.46 (m, 1H, H-1″); 3.14 (m, 4H, NCH_2_); 2.89 (m, 1H, H-4″); 2.46 (m, 2H, H-6-c-hex); 2.25 (m, 2H, H-3-c-hex); 2.14 (m, 2H, H-2″a); 1.96 (m, 2H, H-3″a); 1.74 (m, 2H, H-5-c-hex); 1.64–1.69 (m, 6H, H-4-c-hex, CH_2_-pyrrol.); 1.35 (m, 2H, H-3″b); 1.22 (m, 2H, H-2″b) ppm. ^13^C NMR (DMSO-*d*_6_): δ = 165.59 (HCOOH); 153.79 (C-6); 144.73 (C-1′); 136.87 (C-8); 132.41 (C-9); 128.90 (C-4′); 128.76 (C-3′); 126.61 (C-2); 125.75 (C-3); 123.94 (C-2-c-hex); 119.85 (C-2′); 87.79 (C-7); 49.66 (C-1″); 48.91 (C-4″); 47.78 (NCH_2_); 30.79 (C-2″); 29.99 (C-3″); 26.22 (C-6-c-hex); 25.14 (C-3-c-hex); 24.69 (CH_2_-pyrrol.); 22.34 (C-5-c-hex); 21.77 (C-4-c-hex) ppm. HRMS (ESI): *m*/*z* calculated for C_28_H_38_O_2_N_7_S 536.28022, found 536.27977.

#### *N*^6^-((1*r*,4*r*)-4-Aminocyclohexyl)-3-(cyclohexan-1-yl)-*N*^8^-(4-(1-pyrrolidinylsulfonyl)phenyl)imidazo[1,2-*b*]pyridazine-6,8-diamine (**30**)

Compound **29b** (100 mg, 0.18 mmol) in a mixture of EtOAc (3 mL) and MeOH (1 mL) was treated with Pd/C (10% loading, 20 mg) and stirred under a hydrogen atmosphere (15 bar) for 2 days. The mixture was filtered, evaporated, purified by RP FC (H_2_O/ACN + 0.1% formic acid), and freeze-dried from dioxane to give 70 mg of **30**. MS (ESI): *m*/*z* = 538.5 [M + H]^+^. ^1^H NMR (DMSO-*d*_6_): δ = 8.45 (s, 1H, FA); 7.75 (m, 2H, H-3′); 7.58 (m, 2H, H-2′); 7.09 (s, 1H, H-2); 6.42 (d, 1H, *J* = 6.5 Hz, NH); 6.37 (s, 1H, H-7); 3.49 (m, 1H, H-1′); 3.14 (m, 4H, NCH_2_); 2.91 (m, 2H, H-4″, H-1-c-hex); 2.15 (m, 2H, H-2″a); 2.05 (m, 2H, H-3″a); 1.96 (m, 2H, H-2a-c-hex); 1.79 (m, 2H, H-3a-c-hex); 1.73 (m, 1H, H-4a-c-hex); 1.67 (m, 4H, CH_2_-pyrrol.); 1.52 (m, 2H, H-3″b); 1.36 (m, 4H, H-2b-c-hex, H-3b-c-hex); 1.22 (m, 3H, H-4b-c-hex, H-2″b) ppm. ^13^C NMR (DMSO-*d*_6_): δ = 165.52 (FA); 153.66 (C-6); 144.84 (C-1′); 136.95 (C-8); 133.32 (C-3); 131.27 (C-9); 128.79 (C-3′); 128.74 (C-4′); 124.39 (C-7); 119.78 (C-2′); 87.62 (C-2); 49.43 (C-1″); 49.84 (C-4″); 47.77 (NCH_2_); 33.80 (C-1-c-hex); 30.67 (C-2-c-hex); 30.43 (C-3″); 30.02 (C-2″); 20.95 (C-3, C-4-c-hex); 24.88 (CH_2_-pyrrol.) ppm. HRMS (ESI): *m*/*z* calculated for C_28_H_40_O_2_N_7_S 538.29587, found 538.29535.

#### 6-Chloro-3-iodo-*N*-(4-(1-pyrrolidinylsulfonyl)phenyl)imidazo[1,2-*b*]pyridazin-8-amine (**32**)

Compound **32** was prepared according to **GP1** from 8-bromo-6-chloro-3-iodoimidazo[1,2-*b*]pyridazine^40,41^ White solid, yield 58%. MS (ESI): *m*/*z* = 503.8 [M + H]^+^. ^1^H NMR (DMSO-*d*_6_): δ = 10.27 (bs, 1H, NH); 7.83 (m, 2H, H-3′); 7.81 (s, 1H, H-2); 7.68 (m, 2H, H-2′); 6.83 (s, 1H, H-7); 3.16 (m, 4H, NCH_2_); 1.68 (m, 4H, CH_2_-pyrrol.) ppm. ^13^C NMR (DMSO-*d*_6_): δ = 148.21 (C-6); 142.80 (C-1′); 139.51 (C-8); 137.58 (C-2); 134.49 (C-9); 131.38 (C-4′); 128.92 (C-3′); 121.92 (C-2′); 95.35 (C-7); 72.66 (C-3); 47.86 (NCH_2_); 24.76 (CH_2_-pyrrol.) ppm. HRMS (ESI): *m*/*z* calculated for C_16_H_16_O_2_N_5_ClIS 503.97524, found 503.97476.

#### 6-Chloro-3-(1-cyclopentene-1-yl)-*N*-(4-(1-pyrrolidinylsulfonyl)phenyl)imidazo[1,2-*b*]pyridazine-8-amine (**33a**)

Prepared from **32** and cyclopent-1-en-1-ylboronic acid according to **GP3**. White solid, yield 57%. MS (ESI): *m*/*z* = 444.3 [M + H]^+^. ^1^H NMR (DMSO-*d*_6_): δ = 10.25 (s, 1H, NH); 7.83 (d, 2H, *J* = 8.7 Hz, H-3′); 7.69 (m, 3H, H-2′, H-2); 6.85 (t, 1H, *J* = 2.2 Hz, H-2″); 6.83 (s, 1H, H-7); 3.16 (m, 4H, NCH_2_); 2.79 (m, 2H, H-5″); 2.57 (m, 2H, H-3″); 1.95 (m, 2H, H-4″); 1.68 (m, 4H, CH_2_-pyrrol.) ppm. ^13^C NMR (DMSO-*d*_6_): δ = 147.42 (C-6); 142.92 (C-1′); 139.44 (C-8); 133.32 (C-9); 131.13 (C-4′); 130.39 (C-2); 128.90 (C-3′); 127.53 (C-2″); 121.71 (C-2′); 94.86 (C-7); 47.82 (NCH_2_); 33.51 and 33.33 (C-3″ and C-5″); 24.72 (CH_2_-pyrrol.); 22.00 (C-4″) ppm. HRMS (ESI): *m*/*z* calculated for C_21_H_23_O_2_N_5_ClS 444.12555, found 444.12518.

#### 6-Chloro-3-(propan-2-yl)-*N*-(4-(1-pyrrolidinylsulfonyl)phenyl)imidazo[1,2-*b*]pyridazine-8-amine (**33d**)

Compound **32** (500 mg, 1 mmol) and Pd(dppf)Cl_2_·DCM (81 mg, 0.1 mmol) in dry THF under an argon atmosphere were treated with 2-propylzinc bromide (0.5 M in THF, 3 mL, 1.5 mmol) at RT for 1 h, quenched with sat. NH_4_Cl, diluted with EtOAc, washed with sat. NH_4_Cl, and dried over MgSO_4_. FC (c-hexane/EtOAc + 10% MeOH) afforded a white solid (120 mg, 29%). MS (ESI): *m*/*z* = 420.2 [M + H]^+^. ^1^H NMR (DMSO-*d*_6_): δ = 10.20 (bs, 1H, NH); 7.82 (m, 2H, H-3′); 7.69 (m, 2H, H-2′); 7.50 (d, 1H, *J*(2,1″) = 0.7 Hz, H-2); 6.75 (s, 1H, H-7); 3.16 (m, 4H, NCH_2_); 3.33 (m, 1H, H-1″); 1.69 (m, 4H, CH_2_-pyrrol.); 1.35 (d, 6H, *J*(CH_3_,1″) = 6.9 Hz, CH_3_) ppm. ^13^C NMR (DMSO-*d*_6_): δ = 147.04 (C-6); 143.03 (C-1′); 139.50 (C-8); 135.67 (C-3); 132.03 (C-9); 131.01 (C-4′); 128.89 (C-3′); 127.09 (C-2); 121.66 (C-2′); 94.02 (C-7); 47.81 (NCH_2_); 24.72 (CH_2_-pyrrol.); 23.68 (C-1″); 20.65 (C-2″) ppm. HRMS (ESI): *m*/*z* calculated for C_19_H_23_O_2_N_5_ClS 420.12555, found 420.12517.

#### 6-Chloro-3-methyl-*N*-(4-(1-pyrrolidinylsulfonyl)phenyl)imidazo[1,2-*b*]pyridazine-8-amine (**33e**)

Compound **32** (600 mg, 1.2 mmol), Pd_2_(dba)_3_ (109 mg, 0.12 mmol), and XPhos (114 mg, 0.24 mmol) in THF under an argon atmosphere were treated with DABAL-Me_3_ (0.25 M in THF, 5.7 mL, 1.43 mmol); the mixture was then heated at 60 °C overnight. Another portion of DABAL-Me_3_ (5.7 mL) was added, after which the mixture was again heated at 60 °C for a further 4 h. The mixture was cooled to RT and quenched with sat. NH_4_Cl at 0 °C, diluted with EtOAc, and filtered through Celite. FC (c-hex/EtOAc + 10% MeOH, 0–60%) and RP FC (H_2_O/ACN + 0.1% of formic acid) afforded **33e** (33 mg, 7%). MS (ESI): *m*/*z* = 392.2 [M + H]^+^. ^1^H NMR (DMSO-*d*_6_): δ = 10.18 (bs, 1H, NH); 7.82 (m, 2H, H-3′); 7.68 (m, 2H, H-2′); 7.50 (d, 1H, *J*(2,CH_3_) = 1.0 Hz, H-2); 6.75 (s, 1H, H-7); 3.16 (m, 4H, NCH_2_); 2.45 (d, 3H, *J*(CH_3_,2) = 1.0 Hz, CH_3_); 1.68 (m, 4H, CH_2_-pyrrol.) ppm. ^13^C NMR (DMSO-*d*_6_): δ = 147.18 (C-6); 143.05 (C-1′); 139.37 (C-8); 131.83 (C-9); 130.97 (C-4′); 129.39 (C-2); 128.89 (C-3′); 125.89 (C-3); 121.60 (C-2′); 93.94 (C-7); 47.82 (NCH_2_); 24.73 (CH_2_-pyrrol.); 8.51 (CH_3_) ppm. HRMS (ESI): *m*/*z* calculated for C_17_H_19_O_2_N_5_ClS 392.09425, found 392.09408.

#### 6-Chloro-3-phenyl-*N*-(4-(1-pyrrolidinylsulfonyl)phenyl)imidazo[1,2-*b*]pyridazin-8-amine (**33f**)

##### Method A

Prepared from **32** and phenylboronic acid according to **GP3**. White solid, yield 29%. MS (ESI): *m*/*z* = 454.3 [M + H]^+^. ^1^H NMR (DMSO-*d*_6_): δ = 10.31 (s, 1H, NH); 8.15 (s, 1H, H-2); 8.09 (m, 2H, H-2″); 7.84 (m, 2H, H-3′); 7.72 (m, 2H, H-2′); 7.54 (t, 2H, *J* = 7.8 Hz, H-3″); 7.41 (m, 1H, H-4″); 6.86 (s, 1H, H-7); 3.17 (m, 4H, NCH_2_); 1.69 (m, 4H, CH_2_-pyrrol.) ppm. ^13^C NMR (DMSO-*d*_6_): δ = 147.45 (C-6); 142.84 (C-1′); 139.67 (C-8); 133.34 (C-9); 131.14 (C-4′); 130.34 (C-2); 128.85 (C-3′); 128.69 (C-3″); 128.06 (C-1″); 127.98 (C-4″);126.41 (C-2″); 121.77 (C-2′); 94.81 (C-7); 47.76 (NCH_2_); 24.66 (CH_2_-pyrrol.) ppm. HRMS (ESI): *m*/*z* calculated for C_22_H_21_O_2_N_5_ClS 454.10990, found 454.11032.

##### Method B

Prepared from **43** and **2** according to **GP1**, yield 80%.

#### 6-Chloro-3-(4-methoxyphenyl)-*N*-(4-(1-pyrrolidinylsulfonyl)phenyl)imidazo[1,2-*b*]pyridazin-8-amine (**33g**)

Prepared from **32** and 4-methoxyphenylboronic acid according to **GP3**. White solid, yield 30%. MS (ESI): *m*/*z* = 484.2 [M + H]^+^. ^1^H NMR (DMSO-*d*_6_): δ = 10.28 (s, 1H, NH); 8.05 (s, 1H, H-2); 8.01 (m, 2H, H-2″); 7.84 (m, 2H, H-3′); 7.71 (m, 2H, H-2′); 7.10 (m, 2H, H-3″); 6.82 (s, 1H, H-7); 3.82 (s, 3H, OCH_3_); 3.17 (m, 4H, NCH_2_); 1.69 (m, 4H, CH_2_-pyrrol.) ppm. ^13^C NMR (DMSO-*d*_6_): δ = 159.05 (C-4″); 147.34 (C-6); 142.96 (C-1′); 139.64 (C-8); 132.87 (C-9); 131.11 (C-4′); 129.52 (C-2); 128.89 (C-3′); 128.76 (C-3); 128.00 (C-2″); 121.74 (C-2′); 120.54 (C-1″); 114.20 (C-3″); 94.50 (C-7); 55.23 (OCH_3_); 47.82 (NCH_2_); 24.72 (CH_2_-pyrrol.) ppm. HRMS (ESI): *m*/*z* calculated for C_23_H_23_O_3_N_5_ClS 484.12046, found 484.12032.

#### 6-Chloro-3-(3-methoxyphenyl)-*N*-(4-(1-pyrrolidinylsulfonyl)phenyl)imidazo[1,2-*b*]pyridazin-8-amine (**33h**)

Prepared from **32** and 3-methoxyphenylboronic acid according to **GP3**. White solid, yield 28%. MS (ESI): *m*/*z* = 484.26 [M + H]^+^. ^1^H NMR (DMSO-*d*_6_): δ = 10.31 (s, 1H, NH); 8.20 (s, 1H, H-2); 7.84 (m, 2H, H-3′); 7.68–7.73 (m, 4H, H-2′, 2″, 6″); 7.45 (m, 1H, H-5″); 6.98 (ddd, 1H, *J* = 8.2, 2.6 and 0.9 Hz, H-4″); 6.86 (s, 1H, H-7); 3.84 (s, 3H, OCH_3_); 3.17 (m, 4H, NCH_2_); 1.69 (m, 4H, CH_2_-pyrrol.) ppm. ^13^C NMR (DMSO-*d*_6_): δ = 159.39 (C-3″); 147.48 (C-6); 142.91 (C-1′); 139.73 (C-8); 133.51 (C-9); 131.20 (C-4′); 130.76 (C-2); 129.88 (C-5″); 129.34 (C-1″); 128.92 (C-3′); 128.47 (C-3); 121.84 (C-2′); 118.69 (C-6″); 113.37 (C-4″); 112.05 (C-2″); 94.86 (C-7); 55.19 (OCH_3_); 47.83 (NCH_2_); 24.74 (CH_2_-pyrrol.) ppm. HRMS (ESI): *m*/*z* calculated for C_23_H_23_O_3_N_5_ClS 484.12046, found 484.12033.

#### 6-Chloro-3-(3,4-dimethoxyphenyl)-*N*-(4-(1-pyrrolidinylsulfonyl)phenyl)imidazo[1,2-*b*]pyridazin-8-amine (**33i**)

Prepared from **32** and 3,4-dimethoxyphenylboronic acid according to **GP3**. White solid, yield 19%. MS (ESI): *m*/*z* = 514.0 [M + H]^+^. ^1^H NMR (DMSO-*d*_6_): δ = 10.29 (s, 1H, NH); 8.13 (s, 1H, H-2); 7.84 (m, 2H, H-3′); 7.73–7.70 (m, 3H, H-2′, H-6″); 7.64 (d, 1H, *J* = 2.0 Hz, H-2″); 7.12 (d, 1H, *J* = 8.6 Hz, H-5″); 6.83 (s, 1H, H-7); 3.85 (s, 3H, OCH_3_); 3.82 (s, 3H, OCH_3_); 3.17 (m, 4H, NCH_2_); 1.69 (m, 4H, CH_2_-pyrrol.) ppm. ^13^C NMR (DMSO-*d*_6_): δ = 148.79 and 148.72 (C-3″, C-4″); 147.29 (C-6); 142.95 (C-1′); 139.62 (C-8); 132.94 (C-9); 131.13 (C-4′); 129.81 (C-2); 128.88 (C-3′); 128.77 (C-3); 121.72 (C-2′); 120.70 (C-1″); 119.14 (C-6″); 111.93 (C-2″); 110.44 (C-5″); 94.49 (C-7); 55.63 and 55.57 (OCH_3_); 47.81 (NCH_2_); 24.72 (CH_2_-pyrrol.) ppm. HRMS (ESI): *m*/*z* calculated for C_24_H_25_O_4_N_5_ClS 514.13103, found 514.13070.

#### 6-Chloro-3-(4-fluorophenyl)-*N*-(4-(1-pyrrolidinylsulfonyl)phenyl)imidazo[1,2-*b*]pyridazin-8-amine (**33j**)

Prepared from **32** and 4-fluorophenylboronic acid according to **GP3**. White solid, yield 46%. MS (ESI): *m*/*z* = 472.19 [M + H]^+^. ^1^H NMR (DMSO-*d*_6_): δ = 10.32 (s, 1H, NH); 8.15–8.12 (m, 3H, H-2, H-2″); 7.83 (m, 2H, H-3′); 7.71 (m, 2H, H-2′); 7.38 (m, 2H, H-3″); 6.85 (s, 1H, H-7); 3.17 (m, 4H, NCH_2_); 1.69 (m, 4H, CH_2_-pyrrol.) ppm. ^13^C NMR (DMSO-*d*_6_): δ = 161.66 (d, *J* = 245.9 Hz, C-4″); 147.53 (C-6); 142.86 (C-1′); 139.74 (C-8); 133.29 (C-9); 131.23 (C-4′); 130.34 (C-2); 128.90 (C-3′); 128.64 (d, *J* = 8.2 Hz, C-2″); 127.85 (C-3); 124.69 (d, *J* = 3.2 Hz, C-1″); 121.84 (C-2′); 115.74 (d, *J* = 21.7 Hz, C-3″); 94.83 (C-7); 47.82 (NCH_2_); 24.72 (CH_2_-pyrrol.) ppm. HRMS (ESI): *m*/*z* calculated for C_22_H_20_O_2_N_5_ClFS 472.10048, found 472.10037.

#### 6-Chloro-3-(3-fluoro-4-methoxyphenyl)-*N*-(4-(1-pyrrolidinylsulfonyl)phenyl)imidazo[1,2-*b*]pyridazin-8-amine (**33k**)

Prepared from **32** and 3-fluoro-4-methoxyphenylboronic acid according to **GP3**. White solid, yield 52%. MS (ESI): *m*/*z* = 502.2 [M + H]^+^. ^1^H NMR (DMSO-*d*_6_): δ = 10.29 (s, 1H, NH); 8.15 (s, 1H, H-2); 7.98 (dd, *J* = 13.0, 2.1 Hz, H-2″); 7.92 (m, 1H, H-6″); 7.84 (m, 2H, H-3′); 7.71 (m, 2H, H-2′); 7.33 (t, 1H, *J* = 9.0 Hz, H-5″); 6.84 (s, 1H, H-7); 3.91 (s, 3H, OCH_3_); 3.17 (m, 4H, NCH_2_); 1.69 (m, 4H, CH_2_-pyrrol.) ppm. ^13^C NMR (DMSO-*d*_6_): δ = 151.26 (d, *J* = 243.0 Hz, C-3″); 147.45 (C-6); 146.79 (d, *J* = 10.7 Hz, C-4″); 142.84 (C-1′); 139.67 (C-8); 133.17 (C-9); 131.21 (C-4′); 130.14 (C-2); 128.86 (C-3′); 127.44 (d, *J* = 2.1 Hz, C-3); 122.86 (d, *J* = 3.3 Hz, C-6″); 121.79 (C-2′); 121.00 (d, *J* = 7.7 Hz, C-1″); 114.12 (d, *J* = 2.2 Hz, C-5″); 113.81 (d, *J* = 20.3 Hz, C-2″); 94.69 (C-7); 56.09 (OCH_3_); 47.79 (NCH_2_); 24.72 (CH_2_-pyrrol.) ppm. HRMS (ESI): *m*/*z* calculated for C_23_H_22_O_3_N_5_ClFS 502.11104, found 502.11063.

#### 6-Chloro-3-(4-(trifluoromethyl)phenyl)-*N*-(4-(1-pyrrolidinylsulfonyl)phenyl)imidazo[1,2-*b*]pyridazin-8-amine (**33l**)

Prepared from **32** and 4-(trifluoromethyl)phenylboronic acid according to **GP3**. White solid, yield 40%. MS (ESI): *m*/*z* = 521.95 [M + H]^+^. ^1^H NMR (DMSO-*d*_6_): δ = 10.35 (s, 1H, NH); 8.34 (m, 2H, H-2″); 8.32 (s, 1H, H-2); 7.89 (m, 2H, H-3″); 7.85 (m, 2H, H-3′); 7.72 (m, 2H, H-2′); 7.10 (s, 1H, H-7); 3.17 (m, 4H, NCH_2_); 1.69 (m, 4H, CH_2_-pyrrol.) ppm. ^13^C NMR (DMSO-*d*_6_): δ = 147.77 (C-6); 142.75 (C-1′); 139.84 (C-8); 134.13 (C-9); 132.15 (C-1″); 131.73 (C-2); 131.39 (C-4′); 128.89 (C-2″); 127.78 (d, *J* = 31.9 Hz, C-4′); 127.21 (C-3); 126.59 (C-3′); 125.66 (d, *J* = 3.9 Hz, C-3″); 121.93 (C-2′); 95.34 (C-7); 47.81 (NCH_2_); 24.72 (CH_2_-pyrrol.) ppm. HRMS (ESI): *m*/*z* calculated for C_23_H_20_O_2_N_5_ClF_3_S 522.09728, found 522.09690.

#### 6-Chloro-3-(4-(trifluoromethoxy)phenyl)-*N*-(4-(1-pyrrolidinylsulfonyl)phenyl)imidazo[1,2-*b*]pyridazin-8-amine (**33m**)

Prepared from **32** and 4-(trifluoromethoxy)phenylboronic acid according to **GP3**. White solid, yield 87%. MS (ESI): *m*/*z* = 538.2 [M + H]^+^. ^1^H NMR (DMSO-*d*_6_): δ = 10.33 (s, 1H, NH); 8.22 (m, 2H, H-2″); 8.20 (s, 1H, H-2); 7.84 (m, 2H, H-3′); 7.72 (m, 2H, H-2′); 7.54 (m, 2H, H-3″); 6.86 (s, 1H, H-7); 3.17 (m, 4H, NCH_2_); 1.69 (m, 4H, CH_2_-pyrrol.) ppm. ^13^C NMR (DMSO-*d*_6_): δ = 147.64 (C-6); 142.80 (C-1′); 139.80 (C-8); 133.62 (C-9); 131.32 (C-4′); 130.89 (C-2); 128.88 (C-3′); 128.25 (C-2″); 127.49 (C-1″); 127.42 (C-3); 121.89 (C-2′); 121.39 (C-3″); 95.04 (C-7); 47.80 (NCH_2_); 24.71 (CH_2_-pyrrol.) ppm. HRMS (ESI): *m*/*z* calculated for C_23_H_20_O_3_N_5_ClF_3_S 538.09220, found 538.09181.

#### 6-Chloro-3-(4-cyanophenyl)-*N*-(4-(1-pyrrolidinylsulfonyl)phenyl)imidazo[1,2-*b*]pyridazin-8-amine (**33n**)

Prepared from **32** and 4-cyanophenylboronic acid according to **GP3**. White solid, yield 46%. MS (ESI): *m*/*z* = 479.02 [M + H]^+^. ^1^H NMR (DMSO-*d*_6_): δ = 10.35 (s, 1H, NH); 8.37 (s, 1H, H-2); 8.34 (m, 2H, H-2″); 7.97 (m, 2H, H-3″); 7.83 (m, 2H, H-3′); 7.71 (m, 2H, H-2′); 6.89 (s, 1H, H-7); 3.17 (m, 4H, NCH_2_); 1.69 (m, 4H, CH_2_-pyrrol.) ppm. ^13^C NMR (DMSO-*d*_6_): δ = 147.74 (C-6); 142.62 (C-1′); 139.76 (C-8); 134.36 (C-9); 132.39 (C-3″); 132.56 (C-1″); 132.22 (C-2); 131.35 (C-4′); 128.81 (C-3′); 126.81 (C-3); 126.18 (C-2″); 121.88 (C-2′); 118.76 (CN); 109.66 (C-4″); 95.41 (C-7); 47.73 (NCH_2_); 24.64 (CH_2_-pyrrol.) ppm. HRMS (ESI): *m*/*z* calculated for C_23_H_20_O_2_N_6_ClS 479.10515, found 479.10473.

#### 6-Chloro-3-(3-(*N*,*N*-dimethylcarbamoyl)phenyl)-*N*-(4-(1-pyrrolidinylsulfonyl)phenyl)imidazo[1,2-*b*]pyridazin-8-amine (**33o**)

Prepared from **32** and 3-(*N*,*N*-dimethylcarbamoyl)phenylboronic acid according to **GP3**. White solid, yield 28%. MS (ESI): *m*/*z* = 525.3 [M + H]^+^. ^1^H NMR (DMSO-*d*_6_): δ = 10.34 (s, 1H, NH); 8.24 (s, 1H, H-2); 8.16 (dt, 1H, *J* = 7.9, 1.4 Hz, H-6″); 8.13 (m, 1H, H-2″); 7.84 (m, 2H, H-3′); 7.72 (m, 2H, H-2′); 7.60 (t, 1H, *J* = 7.8 Hz, H-5″); 7.43 (dt, 1H, *J* = 7.7, 1.4 Hz, H-4″); 6.87 (s, 1H, H-7); 3.17 (m, 4H, NCH_2_-pyrrol.); 3.03 and 3.00 (2 × s, 2 × 3H, NCH_3_); 1.69 (m, 4H, CH_2_-pyrrol.) ppm. ^13^C NMR (DMSO-*d*_6_): δ = 169.73 (CO); 147.59 (C-6); 142.86 (C-1′); 139.79 (C-8); 136.91 (C-3′); 133.60 (C-9); 131.25 (C-4′); 130.87 (C-2); 128.92 (C-3′); 128.83 (C-5″); 128.13 (C-1″); 128.06 (C-3); 127.17 (C-6″); 126.47 (C-4″); 124.78 (C-2″); 121.87 (C-2′); 94.98 (C-7); 47.83 (NCH_2_-pyrrol.); 37.69 (NCH_3_); 34.83 (NCH_3_); 24.73 (CH_2_-pyrrol.) ppm. HRMS (ESI): *m*/*z* calculated for C_25_H_26_O_3_N_6_ClS 525.14701, found 525.14670.

#### 6-Chloro-3-(3-(*N*,*N*-dimethylsulfamoyl)phenyl)-*N*-(4-(1-pyrrolidinylsulfonyl)phenyl)imidazo[1,2-*b*]pyridazin-8-amine (**33p**)

Prepared from **32** and 3-(*N*,*N*-dimethylsulfamoyl)phenylboronic acid according to **GP3**. White solid, yield 27%. MS (ESI): *m*/*z* = 561.2 [M + H]^+^. ^1^H NMR (DMSO-*d*_6_): δ = 10.38 (s, 1H, NH); 8.55 (t, 1H, *J* = 1.8 Hz, H-2″); 8.36 (dt, 1H, *J* = 7.8, 1.5 Hz, H-6″); 8.33 (s, 1H, H-2); 7.85 (m, 2H, H-3′); 7.81 (t, 1H, *J* = 7.8 Hz, H-5″); 7.76 (dt, 1H, *J* = 7.9, 1.5 Hz, H-4″); 7.72 (m, 2H, H-2′); 6.90 (s, 1H, H-7); 3.17 (m, 4H, NCH_2_); 2.70 (s, 6H, NCH_3_); 1.69 (m, 4H, CH_2_-pyrrol.) ppm. ^13^C NMR (DMSO-*d*_6_): δ = 147.76 (C-6); 142.80 (C-1′); 139.90 (C-8); 135.35 (C-3″); 133.97 (C-9); 131.38 (C-2); 131.36 (C-4); 130.69 (C-6″); 129.94 (C-5″); 129.27 (C-1″); 128.95 (C-3′); 127.03 (C-3); 126.63 (C-4″); 124.71 (C-2″); 121.95 (C-2′); 95.29 (C-7); 47.85 (NCH_2_-pyrrol.); 37.71 (NCH_3_); 24.75 (CH_2_-pyrrol.) ppm. HRMS (ESI): *m*/*z* calculated for C_24_H_26_O_4_N_6_ClS_2_ 561.11400, found 561.11371.

#### 6-Chloro-3-(thiophen-3-yl)-*N*-(4-(1-pyrrolidinylsulfonyl)phenyl)imidazo[1,2-*b*]pyridazin-8-amine (**33q**)

Prepared from **32** and 3-thienylboronic acid according to **GP3**. White solid, yield 36%. MS (ESI): *m*/*z* = 460.3 [M + H]^+^. ^1^H NMR (DMSO-*d*_6_): δ = 10.30 (s, 1H, NH); 8.32 (dd, 1H, *J* = 2.9, 1.3 Hz, H-4″); 8.18 (s, 1H, H-2); 7.84 (m, 2H, H-3′); 7.81 (m, 1H, H-2″); 7.74 (m, 1H, H-5″); 7.71 (m, 2H, H-2′); 6.86 (s, 1H, H-7); 3.17 (m, 4H, NCH_2_); 1.69 (m, 4H, CH_2_-pyrrol.) ppm. ^13^C NMR (DMSO-*d*_6_): δ = 147.59 (C-6); 142.86 (C-1′); 139.61 (C-8); 132.73 (C-9); 131.20 (C-4′); 129.89 (C-2); 128.86 (C-3′); 128.15 (C-3); 126.74 (C-5″); 126.08 (C-2″); 125.63 (C-3″); 121.78 (C-2′); 121.42 (C-4″); 94.72 (C-7); 47.78 (NCH_2_); 24.69 (CH_2_-pyrrol.) ppm. HRMS (ESI): *m*/*z* calculated for C_20_H_19_O_2_N_5_ClS_2_ 460.06632, found 460.06611.

#### 6-Chloro-3-(furan-3-yl)-*N*-(4-(1-pyrrolidinylsulfonyl)phenyl)imidazo[1,2-*b*]pyridazin-8-amine (**33r**)

Prepared from **32** and 3-furanylboronic acid according to **GP3**. White solid, yield 27%. MS (ESI): *m*/*z* = 444.3 [M + H]^+^. ^1^H NMR (DMSO-*d*_6_): δ = 10.30 (s, 1H, NH); 8.40 (dd, 1H, *J* = 1.6, 0.8 Hz, H-2″); 8.07 (s, 1H, H-2); 7.86 (m, 1H, H-5″); 7.83 (m, 2H, H-3′); 7.71 (m, 2H, H-2′); 7.16 (dd, 1H, *J* = 1.8, 0.8 Hz, H-4″); 6.84 (s, 1H, H-7); 3.17 (m, 4H, NCH_2_-pyrrol.); 1.69 (m, 4H, CH_2_-pyrrol.) ppm. ^13^C NMR (DMSO-*d*_6_): δ = 147.68 (C-6); 143.84 (C-5″); 142.87 (C-1′); 139.57 (C-8); 139.15 (C-2″); 132.87 (C-9); 131.22 (C-4′); 129.30 (C-2); 128.88 (C-3′); 122.79 (C-3); 121.78 (C-2′); 113.56 (C-3″); 108.56 (C-4″); 94.59 (C-7); 47.80 (NCH_2_); 24.71 (CH_2_-pyrrol.) ppm. HRMS (ESI): *m*/*z* calculated for C_20_H_19_O_3_N_5_ClS 444.08916, found 444.08910.

#### *N*^6^-((1*r*,4*r*)-4-Aminocyclohexyl)-3-(1-cyclopenten-1-yl)-*N*^8^-(4-(1-pyrrolidinylsulfonyl)phenyl)imidazo[1,2-*b*]pyridazine-6,8-diamine (**34a**)

Prepared from **33a** according to **GP2**. White solid, yield 46%. MS (ESI): *m*/*z* = 522.5 [M + H]^+^. ^1^H NMR (DMSO-*d*_6_): δ = 9.44 (bs, 1H, NH); 7.98 (d, 2H, *J* = 5.0 Hz, NH_2_); 7.76 (m, 2H, H-3′); 7.59 (m, 2H, H-2′); 7.39 (s, 1H, H-2); 6.86 (m, 1H, c-pent-2); 6.67 (d, 1H, *J*(NH,1″) = 6.8 Hz, NH); 6.49 (s, 1H, H-7); 3.53 (m, 1H, H-1″); 3.14 (m, 4H, NCH_2_); 3.07 (m, 1H, H-4″); 2.75 (m, 2H, c-pent-5); 2.56 (m, 2H, c-pent-3); 2.18 (m, 2H, H-2″a); 1.91–2.03 (m, 4H, H-3″a, c-pent-4); 1.67 (m, 4H, CH_2_-pyrrol.); 1.46 (m, 2H, H-3″b); 1.26 (m, 2H, H-2″b) ppm. ^13^C NMR (DMSO-*d*_6_): δ = 154.34 (C-6); 144.62 (C-1′); 136.80 (C-8); 132.50 (C-9); 130.11 (c-pent-1); 129.06 (C-4′); 128.81 (C-3′); 127.50 (C-2); 125.71 (C-3); 125.02 (c-pent-2); 119.96 (C-2′); 88.37 (C-7); 49.21 (C-4″); 48.76 (C-1″); 47.81 (NCH_2_); 33.46 (c-pent-5); 33.31 (c-pent-3); 29.64 (C-2″); 29.18 (C-3″); 24.72 (CH_2_-pyrrol.); 22.15 (c-pent-4) ppm. HRMS (ESI): *m*/*z* calculated for C_27_H_36_O_2_N_7_S 522.26457, found 522.26499.

#### *N*^6^-((1*r*,4*r*)-4-Aminocyclohexyl)-3-(cyclopentan-1-yl)-*N*^8^-(4-(1-pyrrolidinylsulfonyl)phenyl)imidazo[1,2-*b*]pyridazine-6,8-diamine (**34b**)

Compound **34a** (60 mg) in MeOH (5 mL) was treated with Pd/C (10% loading, 15 mg); the mixture was then stirred under a hydrogen atmosphere (10 bar) for 2 days. The mixture was filtered through Celite and then evaporated and freeze-dried from dioxane to give **34b** as an off-white solid (45 mg, 75%). MS (ESI): *m*/*z* = 524.5 [M + H]^+^. ^1^H NMR (DMSO-*d*_6_): δ = 9.55 (bs, 1H, NH); 8.01 (d, 2H, *J* = 4.8 Hz, NH_2_); 7.78 (m, 2H, H-3′); 7.55 (m, 2H, H-2′); 7.48 (s, 1H, H-2); 6.82 (bs, 1H, NH); 6.58 (s, 1H, H-7); 3.53 (m, 1H, H-1″); 3.35 (m, 1H, c-pent-1); 3.15 (m, 4H, NCH_2_); 3.06 (m, 1H, H-4″); 1.98–2.16 (m, 6H, H-2″a, c-pent-2, c-pent-5); 1.75–1.82 (m, 6H, H-3″a, c-pent-4, c-pent-3); 1.67 (m, 4H, CH_2_-pyrrol.); 1.44 (m, 2H, H-3″b); 1.25 (m, 2H, H-2″b) ppm. ^13^C NMR (DMSO-*d*_6_): δ = 154.57 (C-6); 144.19 (C-1′); 135.77 (C-8); 132.50 (C-9); 132.88 (C-3); 130.07 (C-9); 129.58 (C-4′); 128.98 (C-3′); 121.50 (C-2); 120.04 (C-2′); 90.05 (C-7); 49.05 (C-4″); 48.70 (C-1″); 47.82 (NCH_2_); 34.60 (c-pent-1); 30.25 (c-pent-2, c-pent-5); 29.54 (C-2″); 29.08 (C-3″); 25.11 (c-pent-5, c-pent-4); 24.73 (CH_2_-pyrrol.) ppm. HRMS (ESI): *m*/*z* calculated for C_27_H_38_O_2_N_7_S 524.28022, found 524.28043.

#### *N*^6^-((1*r*,4*r*)-4-Aminocyclohexyl)-3-(2-methylpropan-1-yl)-*N*^8^-(4-(1-pyrrolidinylsulfonyl)phenyl)imidazo[1,2-*b*]pyridazine-6,8-diamine (**34c**)

Compound **32** (500 mg, 1 mmol) and Pd(dppf)Cl_2_·DCM (81 mg, 0.1 mmol) in freshly distilled THF (5 mL) were treated with isobutylzinc bromide (0.5 M solution in THF, 4 mL, 2 mmol) under an argon atmosphere; the mixture was then heated at 45 °C overnight. The mixture was diluted with EtOAc, washed with sat. NH_4_Cl, and dried over MgSO_4_. FC (c-hex/EtOAc + 10% MeOH) afforded an inseparable mixture of **33c** and a dialkylated side product (310 mg, ratio approximately 3:1 of **33c** to side product). MS (ESI): *m*/*z* = 434.1 [M + H]^+^. The mixture was treated with *trans*-1,4-diaminocyclohexane according to **GP2** and freeze-dried from dioxane. White foam, 85 mg of **34c**. MS (ESI): *m*/*z* = 512.5 [M + H]^+^. ^1^H NMR (DMSO-*d*_6_): δ = 8.42 (s, HCOOH); 7.75 (d, 2H, *J* = 8.8 Hz, H-3′); 7.58 (d, 2H, *J* = 8.8 Hz, H-2′); 7.14 (s, 1H, H-2); 6.44 (d, 1H, *J* = 6.7 Hz, NH); 6.37 (s, 1H, H-7); 3.50 (m, 1H, H-1″); 3.14 (m, 4H, NCH_2_); 3.01 (m, 1H, H-4″); 2.67 (d, 2H, *J* = 6.7 Hz, CH_2_-isobutyl); 2.14 (m, 2H, H-2″a); 2.07 (m, 1H, CH-isobutyl); 1.98 (m, 2H, H-3″a); 1.67 (m, 4H, CH_2_-pyrrol.); 1.41 (m, 2H, H-3″b); 1.22 (m, 2H, H-2″b); 0.92 (d, 6H, *J* = 6.7 Hz, CH_3_-isobutyl) ppm. ^13^C NMR (DMSO-*d*_6_): δ = 165.46 (HCOOH); 153.81 (C-6); 144.83 (C-1′); 136.95 (C-8); 131.16 (C-9); 128.82 (C-4′); 128.78 (C-3′); 127.69 (C-3); 126.95 (C-2); 119.83 (C-2′); 87.54 (C-7); 49.06 (C-1″); 48.83 (C-4″); 47.79 (NCH_2_); 32.23 (CH_2_-isobutyl); 29.81 (C-2″); 29.61 (C-3″); 26.74 (CH-isobutyl); 24.70 (CH_2_-pyrrol.); 22.38 (CH_3_-isobutyl) ppm. HRMS (ESI): *m*/*z* calculated for C_26_H_38_O_2_N_7_S 512.28022, found 512.27985.

#### *N*^6^-((1*r*,4*r*)-4-Aminocyclohexyl)-3-(propan-2-yl)-*N*^8^-(4-(1-pyrrolidinylsulfonyl)phenyl)imidazo[1,2-*b*]pyridazine-6,8-diamine (**34d**)

Prepared from **33d** according to **GP2**, isolated as a salt with formic acid, and freeze-dried from water. White foam, yield 46%. MS (ESI): *m*/*z* = 498.46 [M + H]^+^. ^1^H NMR (DMSO-*d*_6_): δ = 8.47 (bs, 1H, FA); 7.75 (m, 2H, H-3′); 7.88 (m, 2H, H-2′); 7.11 (d, 1H, *J*(2,1‴) = 0.7 Hz, H-2); 6.42 (d, 1H, *J*(NH,1″) = 7.0 Hz, NH); 6.39 (s, 1H, H-7); 3.52 (dtt, *J* = 7.5, 3.9 Hz, H-1″); 3.21 (m, 1H, CH-ipr.); 3.14 (m, 4H, NCH_2_); 2.89 (tt, 1H, *J* = 11.1, 4.0 Hz, H-4″); 2.12 (m, 2H, H-2″a); 1.94 (m, 2H, H-3″a); 1.67 (m, 4H, CH_2_-pyrrol.); 1.34 (d, 6H, *J*(CH_3_,CH) = 6.9 Hz, CH_3_-ipr.); 1.17–1.31 (m, 4H, H-3″b, H-2″b) ppm. ^13^C NMR (DMSO-*d*_6_): δ = 165.98 (FA); 153.80 (C-6); 144.85 (C-1′); 136.92 (C-8); 134.25 (C-3); 131.49 (C-9); 128.78 (C-3′); 128.12 (C-4′); 124.23 (C-2); 119.80 (C-2′); 87.69 (C-7); 49.14 (C-4″); 48.96 (C-1″); 47.80 (NCH_2_); 30.83 and 30.10 (C-2″, C-3″); 24.72 (CH_2_-pyrrol.); 24.12 (CH-ipr.); 20.53 (CH_3_) ppm. HRMS (ESI): *m*/*z* calculated for C_25_H_36_O_2_N_7_S 498.26457, found 498.26412.

#### *N*^6^-((1*r*,4*r*)-4-Aminocyclohexyl)-3-methyl-*N*^8^-(4-(1-pyrrolidinylsulfonyl)phenyl)imidazo[1,2-*b*]pyridazine-6,8-diamine (**34e**)

Prepared from **33e** according to **GP2**, isolated as a salt with formic acid, and freeze-dried from water. Off-white foam, yield 87%. MS (ESI): *m*/*z* = 470.44 [M + H]^+^. ^1^H NMR (DMSO-*d*_6_): δ = 8.43 (bs, 1H, NH); 7.75 (m, 2H, H-3′); 7.58 (m, 2H, H-2′); 7.15 (s, 1H, H-2); 6.43 (d, 1H, *J*(NH,1″) = 7.1 Hz, NH); 6.39 (s, 1H, H-7); 3.55 (m, 1H, H-1″); 3.15 (m, 4H, NCH_2_); 2.97 (m, 1H, H-4″); 2.35 (s, 3H, CH_3_); 2.13 (m, 2H, H-2″a); 1.99 (m, 2H, H-3″a); 1.67 (m, 4H, CH_2_-pyrrol.); 1.41 (m, 2H, H-3″b); 1.23 (m, 2H, H-2″b) ppm. ^13^C NMR (DMSO-*d*_6_): δ = 154.10 (C-6); 144.87 (C-1′); 136.84 (C-8); 131.21 (C-9); 128.79 (C-3′); 128.76 (C-4′); 126.54 (C-2); 124.38 (C-3); 119.74 (C-2′); 87.60 (C-7); 48.71 and 48.68 (C-4″, C-1″); 47.81 (NCH_2_); 30.02 (C-2″); 29.74 (C-3″); 24.72 (CH_2_-pyrrol.); 8.57 (CH_3_) ppm. HRMS (ESI): *m*/*z* calculated for C_23_H_32_O_2_N_7_S 470.23327, found 470.23288.

#### *N*^6^-((1*r*,4*r*)-4-Aminocyclohexyl)-3-phenyl-*N*^8^-(4-(1-pyrrolidinylsulfonyl)phenyl)imidazo[1,2-*b*]pyridazine-6,8-diamine (**34f**)

Prepared from **33f** according to **GP2** and freeze-dried from dioxane. White foam, yield 52%. MS (ESI): *m*/*z* = 532.5 [M + H]^+^. ^1^H NMR (DMSO-*d*_6_): δ = 9.50 (s, 1H, NH); 8.21 (m, 2H, Ph-2); 7.90 (s, 1H, H-2); 7.88 (bd, 2H, *J* = 4.5 Hz, NH_2_); 7.78 (m, 2H, H-3′); 7.61 (m, 2H, H-2′); 7.45 (dd, 2H, *J* = 8.4, 7.1 Hz, Ph-3); 7.35 (m, 1H, Ph-4); 6.72 (bs, 1H, NH); 6.51 (s, 1H, H-7); 3.56 (m, 1H, H-1″); 3.15 (m, 4H, NCH_2_); 3.08 (m, 1H, H-4″); 2.19 (m, 2H, H-2″a); 2.02 (m, 2H, H-3″a); 1.68 (m, 4H, CH_2_-pyrrol.); 1.48 (m, 2H, H-3″b); 1.28 (m, 2H, H-2″b) ppm. ^13^C NMR (DMSO-*d*_6_): δ = 154.23 (C-6); 144.54 (C-1′); 137.05 (C-8); 132.51 (C-9); 129.48 (Ph-1); 129.22 (C-4′); 128.83 (C-3′); 128.37 (Ph-3); 127.54 (C-3); 127.32 (Ph-4); 127.05 (C-2); 125.86 (Ph-2); 120.12 (C-2′); 88.45 (C-7); 49.40 (C-4″); 48.77 (C-1″); 47.81 (NCH_2_); 29.63 (C-2″); 29.21 (C-3″); 24.72 (CH_2_-pyrrol.) ppm. HRMS (ESI): *m*/*z* calculated for C_28_H_34_O_2_N_7_S 532.24892, found 532.24922.

#### *N*^6^-((1*r*,4*r*)-4-Aminocyclohexyl)-3-(4-methoxyphenyl)-*N*^8^-(4-(1-pyrrolidinylsulfonyl)phenyl)imidazo[1,2-*b*]pyridazine-6,8-diamine (**34g**)

Prepared from **33g** according to **GP2**. Off-white solid, yield 62%. MS (ESI): *m*/*z* = 562.4 [M + H]^+^. ^1^H NMR (DMSO-*d*_6_): δ = 9.45 (bs, 1H, NH); 8.39 (bs, 1H, FA); 8.14 (m, 2H, H-2″); 7.78–7.75 (m, 3H, H-2, H-3′); 7.61 (m, 2H, H-2’); 7.02 (m, 2H, H-3″); 6.63 (bd, 1H, *J* = 6.5 Hz, NH); 6.45 (s, 1H, H-7); 3.832 (s, 3H, OCH_3_); 3.55 (m, 1H, c-hex-1); 3.15 (m, 4H, NCH_2_); 3.03 (m, 1H, c-hex-4); 2.17 (m, 2H, c-hex-2a); 2.02 (m, 2H, c-hex-3a); 1.68 (m, 4H, CH_2_); 1.49 (m, 2H, c-hex-3b); 1.26 (m, 2H, c-hex-2b) ppm. ^13^C NMR (DMSO-*d*_6_): δ = 158.23 (C-4″); 154.03 (C-6); 144.70 (C-1′); 137.12 (-8); 132.25 (C-9); 129.02 (C-4′); 128.77 (C-3′); 127.47 (C-3); 127.22 (C-2″); 126.83 (C-2); 122.25 (C-1″); 119.98 (C-2′); 113.79 (C-3″); 87.82 (C-7); 55.16 (OCH_3_); 49.46 (c-hex-4); 48.65 (c-hex-1); 47.79 (NCH_2_); 29.81 and 29.64 (c-hex-2, 3); 24.70 (CH_2_) ppm. HRMS (ESI): *m*/*z* calculated for C_29_H_36_O_3_N_7_S 562.25949, found 562.25957.

#### *N*^6^-((1*r*,4*r*)-4-Aminocyclohexyl)-3-(3-methoxyphenyl)-*N*^8^-(4-(1-pyrrolidinylsulfonyl)phenyl)imidazo[1,2-*b*]pyridazine-6,8-diamine (**34h**)

Prepared from **33h** according to **GP2**. Off-white solid, yield 58%. MS (ESI): *m*/*z* = 562.5 [M + H]^+^. ^1^H NMR (DMSO-*d*_6_): δ = 8.41 (bs, 1H, FA); 7.90 (dd, 1H, *J* = 2.6, 1.6 Hz, H-2″); 7.88 (s, 1H, H-2); 7.77 (m, 2H, H-3′); 7.73 (dt, 1H, *J* = 8.0, 1.1 Hz, H-6″); 7.62 (m, 2H, H-2′); 7.35 (t, 1H, *J* = 8.0 Hz, H-5″); 6.89 (ddd, 1H, *J* = 8.3, 2.6, 0.9 Hz, H-4″); 6.63 (d, 1H, *J* = 6.7 Hz, NH); 6.49 (s, 1H, H-7); 3.85 (s, 3H, OCH_3_); 3.57 (m, 1H, c-hex-1); 3.15 (m, 4H, NCH_2_-pyrrol.); 3.01 (m, 1H, c-hex-4); 2.18 (m, 2H, c-hex-2a); 2.00 (m, 2H, c-hex-3a); 1.69 (m, 4H, CH_2_-pyrrol.); 1.43 (m, 4H, c-hex-3b); 1.27 (m, 4H, c-hex-2b) ppm. ^13^C NMR (DMSO-*d*_6_): δ = 165.28 (FA); 159.36 (C-3″); 154.19 (C-6); 144.63 (C-1′); 137.13 (C-8); 132.91 (C-9); 130.88 (C-1″); 129.38 (C-5″); 129.12 (C-4′); 128.78 (C-3′); 128.23 (C-2); 127.34 (C-3); 120.07 (C-2′); 118.06 (C-6″); 112.56 (C-4″); 111.16 (C-2″); 88.17 (C-7); 55.22 (OCH_3_); 49.19 (c-hex-1); 48.67 (c-hex-4); 47.79 (NCH_2_-pyrrol.); 29.91 and 29.73 (c-hex-2, 3); 24.71 (CH_2_-pyrrol.) ppm. HRMS (ESI): *m*/*z* calculated for C_29_H_36_O_3_N_7_S 562.25949, found 562.25907.

#### *N*^6^-((1*r*,4*r*)-4-Aminocyclohexyl)-3-(3,4-dimethoxyphenyl)-*N*^8^-(4-(1-pyrrolidinylsulfonyl)phenyl)imidazo[1,2-*b*]pyridazine-6,8-diamine (**34i**)

Prepared from **33i** according to **GP2**. Off-white solid, yield 35%. MS (ESI): *m*/*z* = 592.50 [M + H]^+^. ^1^H NMR (DMSO-*d*_6_): δ = 9.44 (bs, 1H, NH); 8.37 (bs, 2H, NH_2_); 7.82 (d, 1H, *J* = 2.1 Hz, H-2″); 7.79–7.75 (m, 3H, H-2, H-3′); 7.70 (dd, 1H, *J* = 8.4, 2.0 Hz, H-6″); 7.62 (m, 2H, H-2′); 7.03 (d, 1H, *J* = 8.6 Hz, H-5″); 6.57 (d, 1H, *J* = 7.1 Hz, NH); 6.46 (s, 1H, H-7); 3.87 and 3.81 (s, 3H, OCH_3_); 3.61 (m, 1H, c-hex-1); 3.15 (m, 4H, NCH_2_); 3.02 (m, 1H, c-hex-4); 2.15 (m, 2H, c-hex-2a); 1.99 (m, 2H, c-hex-3a); 1.68 (m, 4H, CH_2_-pyrrol.); 1.41 (m, 2H, c-hex-3b); 1.26 (m, 2H, c-hex-2b) ppm. ^13^C NMR (DMSO-*d*_6_): δ = 154.15 (C-6); 148.56 and 148.03 (C-3″, C-4″); 144.69 (C-1′); 137.07 (C-8); 132.42 (C-9); 129.03 (C-4′); 128.78 (C-3′); 127.71 (C-3); 127.28 (C-2); 122.50 (C-1″); 119.98 (C-2′); 118.58 (C-6″); 111.81 (C-5″); 109.98 (C-2″); 87.97 (C-7); 55.75 and 55.56 (2 × OCH_3_); 48.77 and 48.61 (c-hex-1 and 4); 47.79 (NCH_2_); 29.95 and 29.55 (c-hex-2 and 3); 24.70 (CH_2_-pyrrol.) ppm. HRMS (ESI): *m*/*z* calculated for C_30_H_38_O_4_N_7_S 592.27005, found 592.26955.

#### *N*^6^-((1*r*,4*r*)-4-Aminocyclohexyl)-3-(4-fluorophenyl)-*N*^8^-(4-(1-pyrrolidinylsulfonyl)phenyl)imidazo[1,2-*b*]pyridazine-6,8-diamine (**34j**)

Prepared from **33j** according to **GP2**. Off-white solid, yield 69%. MS (ESI): *m*/*z* = 550.37 [M + H]^+^. ^1^H NMR (DMSO-*d*_6_): δ = 8.42 (bs, 1H, NH); 8.26 (dd, 2H, *J* = 8.9, 5.6 Hz, H-2″); 7.84 (s, 1H, H-2); 7.77 (m, 2H, H-3′); 7.62 (m, 2H, H-2′); 7.29 (t, 2H, *J* = 8.9 Hz, H-3″); 6.68 (d, 1H, *J* = 6.7 Hz, NH); 6.48 (s, 1H, H-7); 3.54 (m, 1H, c-hex-1); 3.15 (m, 4H, NCH_2_); 3.02 (m, 1H, c-hex-4); 2.15 (m, 2H, c-hex-2a); 2.01 (m, 2H, c-hex-3a); 1.68 (m, 4H, CH2); 1.48 (m, 2H, c-hex-3b); 1.26 (m, 2H, c-hex-2b) ppm. ^13^C NMR (DMSO-*d*_6_): δ = 160.97 (d, *J* = 244.3 Hz, C-4″); 154.17 (C-6); 144.61 (C-1′); 137.21 (C-8); 132.63 (C-9); 129.15 (C-4′); 128.77 (C-3′); 127.81 (d, *J* = 7.8 Hz, C-2″); 127.72 (C-2); 126.60 (C-3); 126.24 (d, *J* = 3.2 Hz, C-1″); 120.10 (C-2′); 115.21 (d, *J* = 21.2 Hz, C-3″); 88.07 (C-7); 49.51 (c-hex-1); 48.66 (c-hex-4); 47.79 (NCH_2_); 29.89 (c-hex-2, 3); 24.70 (CH_2_) ppm. HRMS (ESI): *m*/*z* calculated for C_28_H_33_O_2_N_7_FS 550.23950, found 550.23942.

#### *N*^6^-((1*r*,4*r*)-4-Aminocyclohexyl)-3-(3-fluoro-4-hydroxyphenyl)-*N*^8^-(4-(1-pyrrolidinylsulfonyl)phenyl)imidazo[1,2-*b*]pyridazine-6,8-diamine (**34k**)

Prepared from **33k** according to **GP2**. Heating of the reaction mixture at high temperatures in basic media led to the cleavage of a methoxy group and hydroxy derivative **34k**, which was isolated as a major product. Off-white solid, yield 38%. MS (ESI): *m*/*z* = 566.5 [M + H]^+^. ^1^H NMR (DMSO-*d*_6_): δ = 8.42 (bs, 1H, FA); 8.13 (dd, 1H, *J* = 13.6, 2.1 Hz, H-2″); 7.78–7.72 (m, 4H, H-2, H-3′, H-6″); 7.61 (m, 2H, H-2′); 7.05 (m, 1H, H-5″); 6.64 (d, 1H, *J* = 6.4 Hz, NH); 6.45 (s, 1H, H-7); 3.52 (m, 1H, c-hex-1); 3.15 (m, 4H, NCH_2_-pyrrol.); 3.03 (m, 1H, c-hex-4); 2.21 (m, 2H, c-hex-2a); 2.02 (m, 2H, c-hex-3a); 1.68 (m, 4H, CH_2_-pyrrol.); 1.46 (m, 2H, c-hex-3b); 1.28 (m, 2H, c-hex-2b) ppm. ^13^C NMR (DMSO-*d*_6_): δ = 154.10 (C-6); 150.80 (d, *J* = 239.3 Hz, C-3″); 144.66 (C-1′); 144.32 (d, *J* = 12.0 Hz, C-4″); 137.12 (C-8); 132.31 (C-9); 129.06 (C-4′); 128.77 (C-3′); 127.00 (C-2); 126.76 (d, *J* = 2.0 Hz, C-3); 122.18 (d, *J* = 2.9 Hz, H-6″); 120.93 (d, *J* = 7.4 Hz, C-1″); 120.02 (C-2′); 117.84 (d, *J* = 3.4 Hz, C-5″); 113.41 (d, *J* = 21.5 Hz, C-2″); 87.84 (C-7); 49.54 (c-hex-1); 48.74 (c-hex-4); 47.79 (NCH_2_-pyrrol.); 29.82 (c-hex-2); 29.65 (c-hex-3); 24.70 (CH_2_-pyrrol.) ppm. HRMS (ESI): *m*/*z* calculated for C_28_H_33_O_3_N_7_FS 566.23441, found 566.23436.

#### *N*^6^-((1*r*,4*r*)-4-Aminocyclohexyl)-3-(4-(trifluoromethyl)phenyl)-*N*^8^-(4-(1-pyrrolidinylsulfonyl)phenyl)imidazo[1,2-*b*]pyridazine-6,8-diamine (**34l**)

Prepared from **33l** according to **GP2**. Off-white solid, yield 71%. MS (ESI): *m*/*z* = 600.37 [M + H]^+^. ^1^H NMR (DMSO-*d*_6_): δ = 8.50 (d, 2H, *J* = 8.2 Hz, H-2″); 8.47 (s, 1H, FA); 8.05 (s, 1H, H-2); 7.78 (m, 4H, H-3′, 3″); 7.62 (m, 2H, H-2′); 6.77 (d, 1H, *J* = 6.8 Hz, NH); 6.52 (s, 1H, H-7); 3.58 (m, 1H, c-hex-1); 3.15 (m, 4H, NCH_2_); 2.97 (m, 1H, c-hex-4); 2.17 (m, 2H, c-hex-2a); 2.01 (m, 2H, c-hex-3a); 1.68 (m, 4H, CH_2_); 1.50 (m, 2H, c-hex-3b); 1.25 (m, 2H, c-hex-2b) ppm. ^13^C NMR (DMSO-*d*_6_): δ = 165.71 (FA); 154.41 (C-6); 144.54 (C-1′); 137.29 (C-8); 133.73 (C-1″); 133.55 (C-9); 129.43 (C-2); 129.28 (C-4′); 128.84 (C-3′); 126.64 (d, *J* = 32.0 Hz, C-4″); 125.96 (C-3); 125.66 (C-2″); 125.25 (d, *J* = 4.4 Hz, C-3″); 120.27 (C-2′); 88.48 (C-7); 49.61 (c-hex-1); 48.77 (c-hex-4); 47.83 (NCH_2_); 30.19 and 29.95 (c-hex-2 and 3); 24.74 (CH_2_) ppm. HRMS (ESI): *m*/*z* calculated for C_29_H_33_O_2_N_7_F_3_S 600.23631, found 600.23582.

#### *N*^6^-((1*r*,4*r*)-4-Aminocyclohexyl)-3-(4-(trifluoromethoxy)phenyl)-*N*^8^-(4-(1-pyrrolidinylsulfonyl)phenyl)imidazo[1,2-*b*]pyridazine-6,8-diamine (**34m**)

Prepared from **33m** according to **GP2**. Off-white solid, yield 61%. MS (ESI): *m*/*z* = 616.4 [M + H]^+^. ^1^H NMR (DMSO-*d*_6_): δ = 8.48 (bs, 1H, FA); 8.37 (d, 2H, *J* = 9.0 Hz, H-2″); 7.92 (s, 1H, H-2); 7.78 (m, 2H, H-3′); 7.62 (m, 2H, H-2′); 7.44 (m, 2H, H-3″); 6.68 (d, 1H, *J* = 6.7 Hz, NH); 6.50 (s, 1H, H-7); 3.56 (m, 1H, c-hex-1); 3.15 (m, 4H, NCH_2_-pyrrol.); 2.90 (m, 1H, c-hex-4); 2.15 (m, 2H, c-hex-2a); 1.97 (m, 2H, c-hex-3a); 1.68 (m, 4H, CH_2_-pyrrol.); 1.43 (m, 2H, c-hex-3b); 1.25 (m, 2H, c-hex-2b) ppm. ^13^C NMR (DMSO-*d*_6_): δ = 154.31 (C-6); 146.79 (d, *J* = 2.0 Hz, C-4″); 144.57 (C-1′); 137.20 (C-8); 133.01 (C-9); 129.21 (C-4′); 129.11 (C-3); 128.77 (C-3′); 128.40 (C-2); 127.25 (C-2″); 126.11 (C-1″); 120.98 (C-3″); 120.16 (C-2′); 88.28 (C-7); 49.64 (c-hex-1); 48.96 (c-hex-4); 47.79 (NCH_2_-pyrrol.); 30.95 (c-hex-3); 30.08 (c-hex-2); 24.70 (CH_2_-pyrrol.) ppm. HRMS (ESI): *m*/*z* calculated for C_29_H_33_O_3_N_7_F_3_S 616.23122, found 616.23077.

#### *N*^6^-((1*r*,4*r*)-4-Aminocyclohexyl)-3-(4-cyanophenyl)-*N*^8^-(4-(1-pyrrolidinylsulfonyl)phenyl)imidazo[1,2-*b*]pyridazine-6,8-diamine (**34n**)

Prepared from **33n** according to **GP2**. Off-white solid, yield 28%. MS (ESI): *m*/*z* = 557.49 [M + H]^+^. ^1^H NMR (DMSO-*d*_6_): δ = 8.48 (d, 2H, *J* = 8.7 Hz, H-2″); 8.44 (s, 1H, FA); 8.09 (s, 1H, H-2); 7.88 (m, 2H, H-3″); 7.78 (m, 2H, H-3′); 7.62 (m, 2H, H-2′); 6.78 (d, 1H, *J* = 6.7 Hz, NH); 6.52 (s, 1H, H-7); 3.57 (m, 1H, c-hex-1); 3.15 (m, 4H, NCH_2_); 3.02 (m, 1H, c-hex-4); 2.15 (m, 2H, c-hex-2a); 2.02 (m, 2H, c-hex-3a); 1.68 (m, 4H, CH_2_); 1.52 (m, 2H, c-hex-3b); 1.27 (m, 2H, c-hex-2b) ppm. ^13^C NMR (DMSO-*d*_6_): δ = 165.41 (FA); 154.39 (C-6); 144.44 (C-1′); 137.30 (C-8); 134.18 (C-1″); 133.77 (C-9); 132.27 (C-3′); 130.00 (C-2); 129.38 (C-4′); 128.78 (C-3′); 125.75 (C-3); 125.62 (C-2″); 120.30 (C-2′); 119.02 (CN); 108.50 (C-4″); 88.56 (C-7); 49.61 (c-hex-4); 48.66 (c-hex-1); 47.79 (NCH_2_); 29.84 and 29.79 (c-hex-2 and 3); 24.71 (CH_2_) ppm. HRMS (ESI): *m*/*z* calculated for C_29_H_33_O_2_N_8_S 557.24417, found 557.24377. Purity was 93.7% due to complications with purification.

#### *N*^6^-((1*r*,4*r*)-4-Aminocyclohexyl)-3-(3-(*N*,*N*-dimethylcarbamoyl)phenyl)-*N*^8^-(4-(1-pyrrolidinylsulfonyl)phenyl)imidazo[1,2-*b*]pyridazine-6,8-diamine (**34o**)

Prepared from **33o** according to **GP2**. Off-white solid, yield 54%. MS (ESI): *m*/*z* = 603.5 [M + H]^+^. ^1^H NMR (DMSO-*d*_6_): δ = 8.43 (s, 1H, FA); 8.26 (m, 1H, H-2″); 8.24 (m, 1H, H-6″); 7.94 (s, 1H, H-2); 7.78 (m, 2H, H-3′); 7.62 (m, 2H, H-2′); 7.50 (td, 1H, *J* = 7.6, 0.9 Hz, H-5″); 7.32 (dt, 1H, *J* = 7.6, 1.4 Hz, H-4″); 6.64 (d, 1H, *J* = 6.5 Hz, NH); 6.50 (s, 1H, H-7); 3.48 (m, 1H, c-hex-1); 3.15 (m, 4H, NCH_2_-pyrrol.); 3.03 (s, 3H, NCH_3_); 2.97–2.92 (m, 4H, NCH_3_, c-hex-4); 2.18 (m, 2H, c-hex-2a); 1.97 (m, 2H, c-hex-3a); 1.68 (m, 4H, CH_2_-pyrrol.); 1.38 (m, 2H, c-hex-3b); 1.25 (m, 2H, c-hex-2b) ppm. ^13^C NMR (DMSO-*d*_6_): δ = 170.05 (CO); 154.32 (C-6); 144.59 (C-1′); 137.13 (C-3″); 136.98 (C-8); 133.02 (C-9); 129.71 (C-1″); 129.30 (C-2); 129.17 (C-4′); 128.78 (C-3′); 128.36 (C-5″); 126.83 (C-3); 126.17 (C-6″); 125.07 (C-4″); 123.77 (C-2″); 120.11 (C-2′); 88.28 (C-7); 49.46 (c-hex-1); 48.88 (c-hex-4); 47.79 (NCH_2_-pyrrol.); 30.28 (c-hex-3); 30.05 (c-hex-2); 24.70 (CH_2_-pyrrol.) ppm. HRMS (ESI): *m*/*z* calculated for C_31_H_39_O_3_N_8_S 603.28603, found 603.28597.

#### *N*^6^-((1*r*,4*r*)-4-Aminocyclohexyl)-3-(3-(*N*,*N*-dimethylsulfamoyl)phenyl)-*N*^8^-(4-(1-pyrrolidinylsulfonyl)phenyl)imidazo[1,2-*b*]pyridazine-6,8-diamine (**34p**)

Prepared from **33p** according to **GP2**. Off-white solid, yield 58%. MS (ESI): *m*/*z* = 639.48 [M + H]^+^. ^1^H NMR (DMSO-*d*_6_): δ = 8.73 (t, 1H, *J* = 1.8 Hz, H-2″); 8.47 (dt, 1H, *J* = 7.7, 1.6 Hz, H-6″); 8.39 (bs, 1H, FA); 8.04 (s, 1H, H-2); 7.78 (m, 2H, H-3′); 7.72 (t, 1H, *J* = 7.8 Hz, H-5″); 7.67 (dt, 1H, *J* = 7.8, 1.5 Hz, H-4″); 7.62 (m, 2H, H-2′); 6.64 (d, 1H, *J* = 7.5 Hz, NH); 6.53 (s, 1H, H-7); 3.66 (m, 1H, c-hex-1); 3.15 (m, 4H, NCH_2_-pyrrol.); 2.97 (m, 1H, c-hex-4); 2.67 (s, 6H, NCH_3_); 2.15 (m, 2H, c-hex-2a); 1.97 (m, 2H, c-hex-3a); 1.68 (m, 4H, CH_2_-pyrrol.); 1.51 (m, 2H, c-hex-3b); 1.26 (m, 2H, c-hex-2b) ppm. ^13^C NMR (DMSO-*d*_6_): δ = 154.58 (C-6); 144.50 (C-1′); 137.16 (C-8); 135.14 (C-3″); 133.49 (C-9); 130.76 (C-1″); 129.59 (C-6″); 129.48 (C-5″); 129.28 (C-4′); 129.10 (C-2); 128.79 (C-3′); 125.93 (C-3); 125.49 (C-4″); 123.81 (C-2″); 120.20 (C-2′); 88.64 (C-7); 48.81 (c-hex-1); 48.46 (c-hex-4); 47.80 (NCH_2_-pyrrol.); 37.70 (NCH_3_); 30.23 (c-hex-2); 29.59 (c-hex-3); 24.71 (CH_2_-pyrrol.) ppm. HRMS (ESI): *m*/*z* calculated for C_30_H_39_O_4_N_8_S_2_ 639.25302, found 639.25276.

#### *N*^6^-((1*r*,4*r*)-4-Aminocyclohexyl)-3-(thiophen-3-yl)-*N*^8^-(4-(1-pyrrolidinylsulfonyl)phenyl)imidazo[1,2-*b*]pyridazine-6,8-diamine (**34q**)

Prepared from **33q** according to **GP2**. Off-white solid, yield 32%. MS (ESI): *m*/*z* = 538.2 [M + H]^+^. ^1^H NMR (DMSO-*d*_6_): δ = 8.45 (bs, 1H, FA); 8.34 (dd, 1H, *J* = 3.0, 1.2 Hz, H-4″); 7.86 (s, 1H, H-2); 7.76–7.80 (m, 3H, H-3′, H-2″); 7.68 (m, 1H, H-2″); 7.62 (m, 2H, H-2′); 6.69 (d, 1H, *J* = 6.8 Hz, NH); 6.49 (s, 1H, H-7); 3.63 (m, 1H, c-hex-1); 3.16 (m, 4H, NCH_2_); 2.99 (m, 1H, c-hex-4); 2.20 (m, 2H, c-hex-2a); 2.02 (m, 2H, c-hex-3a); 1.69 (m, 4H, CH_2_-pyrrol.); 1.49 (m, 2H, c-hex-3b); 1.27 (m, 2H, c-hex-2b) ppm. ^13^C NMR (DMSO-*d*_6_): δ = 154.47 (C-6); 144.68 (C-1′); 137.12 (C-8); 132.13 (C-9); 129.88 (C-4′); 129.14 (C-3); 128.80 (C-3′); 128.01 (C-2); 127.32 (C-5″); 126.06 (C-2″); 124.85 (C-3″); 120.06 (C-2′); 118.89 (C-4″); 88.01 (C-7); 49.50 (c-hex-1); 48.80 (c-hex-4); 47.80 (NCH_2_); 30.16 and 29.95 (c-hex-2, 3); 224.72 (CH_2_-pyrrol.) ppm. HRMS (ESI): *m*/*z* calculated for C_26_H_32_O_2_N_7_S_2_ 538.20534, found 538.20500.

#### *N*^6^-((1*r*,4*r*)-4-Aminocyclohexyl)-3-(furan-3-yl)-*N*^8^-(4-(1-pyrrolidinylsulfonyl)phenyl)imidazo[1,2-*b*]pyridazine-6,8-diamine (**34r**)

Prepared from **33r** according to **GP2**. Off-white solid, yield 57%. MS (ESI): *m*/*z* = 522.4 [M + H]^+^. ^1^H NMR (DMSO-*d*_6_): δ = 8.41 (s, 1H, FA); 8.37 (dd, 1H, *J* = 1.7, 0.7 Hz, H-2″); 7.80 (t, 1H, *J* = 1.7 Hz, H-5″); 7.77 (m, 2H, H-3′); 7.73 (s, 1H, H-2); 7.61 (m, 2H, H-2′); 7.10 (dd, 1H, *J* = 1.9, 0.7 Hz, H-4″); 6.68 (d, 1H, *J* = 6.8 Hz, NH); 6.46 (s, 1H, H-7); 3.60 (m, 1H, c-hex-1); 3.15 (m, 4H, NCH_2_-pyrrol.); 3.02 (m, 1H, c-hex-4); 2.19 (m, 2H, c-hex-2a); 2.03 (m, 2H, c-hex-3a); 1.68 (m, 4H, CH_2_-pyrrol.); 1.49 (m, 2H, c-hex-3b); 1.27 (m, 2H, c-hex-2b) ppm. ^13^C NMR (DMSO-*d*_6_): δ = 165.35 (FA); 154.51 (C-6); 144.64 (C-1′); 137.06 (C-8); 132.25 (C-9); 129.10 (C-4′); 128.78 (C-3′); 126.56 (C-2); 121.84 (C-3); 120.03 (C-2′); 114.89 (C-3″); 108.44 (C-4″); 87.79 (C-7); 49.35 (c-hex-1); 48.64 (c-hex-4); 47.80 (NCH_2_-pyrrol.); 29.85 and 29.79 (c-hex-2 and 3); 24.71 (CH_2_-pyrrol.) ppm. HRMS (ESI): *m*/*z* calculated for C_26_H_32_O_3_N_7_S 522.22819, found 522.22793.

#### 6-Chloro-3-nitro-*N*-(4-(1-pyrrolidinylsulfonyl)phenyl)imidazo[1,2-*b*]pyridazine-8-amine (**37**)

In concentrated sulfuric acid (10 mL), 8-bromo-3-chloroimidazo[1,2-*b*]pyridazine (1 g, 4.3 mmol) was treated with HNO_3_ (1.5 mL) at 0 °C. The resulting mixture was stirred at RT for 5 h, poured over ice, extracted with EtOAc, washed with sat. NaHCO_3_ and water, and dried over MgSO_4_. MS (ESI): *m*/*z* = 277.0 [M + H]^+^. The crude product was converted to **37** according to **GP1**. Yellow solid, yield 72%. MS (ESI): *m*/*z* = 423.2 [M + H]^+^. ^1^H NMR (DMSO-*d*_6_): δ = 10.60 (s, 1H, NH); 8.76 (s, 1H, H-2); 7.86 (m, 2H, H-3′); 7.71 (m, 2H, H-2′); 7.11 (s, 1H, H-7); 3.17 (m, 4H, NCH_2_); 1.69 (m, 4H, CH_2_-pyrrol.) ppm. ^13^C NMR (DMSO-*d*_6_): δ = 150.22 (C-6); 142.08 (C-1′); 140.20 (C-8); 134.94 (C-9); 134.89 (C-3); 134.43 (C-2); 132.08 (C-4′); 128.95 (C-3′); 122.41 (C-2′); 98.91 (C-7); 47.84 (NCH_2_); 24.75 (CH_2_-pyrrol.) ppm. HRMS (ESI): *m*/*z* calculated for C_16_H_16_O_4_N_6_ClS 423.06368, found 423.06343.

#### *N*^6^-((1*r*,4*r*)-4-Aminocyclohexyl)-3-amino-*N*^8^-(4-(1-pyrrolidinylsulfonyl)phenyl)imidazo[1,2-*b*]pyridazine-6,8-diamine (**39**)

Compound **37** (200 mg, 0.47 mmol) was converted to amine **38** according to **GP2**. Off-white solid, yield 100 mg (42%). MS (ESI): *m*/*z* = 501.4 [M + H]^+^. Crude **38** (80 mg, 0.16 mmol) in EtOH (13 mL) was treated with SnCl_2_ (121 mg, 0.64 mmol) and heated at 75 °C for 2 h. The mixture was diluted with EtOAc and extracted with NaOH. RP FC (H_2_O/ACN + 0.1% formic acid) afforded **39** (40 mg, 53%) as a salt with formic acid. MS (ESI): *m*/*z* = 471.4 [M + H]^+^. ^1^H NMR (DMSO-*d*_6_): δ = 9.22 (bs, 1H, NH); 8.40 (s, 1H, HCOOH); 7.72 (m, 2H, H-3′); 7.55 (m, 2H, H-2′); 6.59 (s, 1H, H-2); 6.29 (d, 1H, *J*(NH,1″) = 7.2 Hz, NH); 6.21 (s, 1H, H-7); 3.61 (m, 1H, H-1″); 3.13 (m, 4H, NCH_2_); 2.96 (m, 1H, H-4″); 2.13 (m, 2H, H-2″a); 1.98 (m, 2H, H-3″a); 1.66 (m, 4H, CH_2_-pyrrol.); 1.44 (m, 2H, H-3″b); 1.20 (m, 2H, H-2″b) ppm. ^13^C NMR (DMSO-*d*_6_): δ = 165.40 (HCOOH); 153.69 (C-6); 145.15 (C-1′); 136.63 (C-8); 134.71 (C-3); 128.76 (C-3′); 128.29 (C-4′); 125.87 (C-9); 119.38 (C-2′); 110.49 (C-2); 86.01 (C-7); 48.73 (C-4″); 48.39 (C-1″); 47.99 (NCH_2_); 30.17 (C-2″); 29.65 (C-3″); 24.70 (CH_2_-pyrrol.) ppm. HRMS (ESI): *m*/*z* calculated for C_22_H_31_O_2_N_8_S 471.22852, found 471.22859. Purity was 90.55% due to complications of purification.

#### 6-Chloro-3-(1*H*-pyrazole-4-yl)-*N*-(4-(1-pyrrolidinylsulfonyl)phenyl)imidazo[1,2-*b*]pyridazine-8-amine (**41a**) and 6-Chloro-*N*-(4-(1-pyrrolidinylsulfonyl)phenyl)imidazo[1,2-*b*]pyridazine-8-amine (**41b**)

Compounds **32** (600 mg, 1.2 mmol) and **40** (667 mg, 2.4 mmol), Pd(dppf)Cl_2_·DCM (97.3 mg, 0.12 mmol), and Na_2_CO_3_ (379 mg, 3.57 mmol) in a dioxane/water mixture (9:1, 20 mL) under an argon atmosphere were heated at 100 °C overnight. Compound **40** (200 mg) and Pd(dppf)Cl_2_·DCM (48 mg) were added; the mixture was heated for a further 24 h. The mixture was then diluted with EtOAc, washed with sat. NH_4_Cl, and dried over MgSO_4_. FC (c-hex/EtOAc + 10% MeOH) and repurification by RP FC (H_2_O/ACN) afforded compounds **41a** (white solid, 160 mg, 30%) and **41b** (white solid, 70 mg, 16%).

**41a.** MS (ESI): *m*/*z* = 444.2 [M + H]^+^. ^1^H NMR (DMSO-*d*_6_): δ = 13.19 (bs, 1H, NH-1″); 10.27 (s, 1H, NH-8); 8.41 and 8.17 (bs, 1H, H-3″, H-5″); 8.00 (s, 1H, H-2); 7.83 (m, 2H, H-3′); 7.71 (m, 2H, H-2′); 6.80 (s, 1H, H-7); 3.17 (m, 4H, NCH_2_); 1.69 (m, 4H, CH_2_-pyrrol.) ppm. ^13^C NMR (DMSO-*d*_6_): δ = 147.49 (C-6); 143.00 (C-1′); 139.50 (C-8); 136.69 and 125.68 (C-3″, C-5″); 132.05 (C-9); 131.05 (C-4′); 128.90 (C-3′); 128.00 (C-2); 123.61 (C-3); 121.68 (C-2′); 108.44 (C-4″); 94.15 (C-7); 47.82 (NCH_2_); 24.73 (CH_2_-pyrrol.) ppm. HRMS (ESI): *m*/*z* calculated for C_19_H_19_O_2_N_7_ClS 444.10040, found 444.10013.

**41b.** MS (ESI): *m*/*z* = 378.2 [M + H]^+^. ^1^H NMR (DMSO-*d*_6_): δ = 10.26 (s, 1H, NH); 8.19 (d, 1H, *J*(3,2) = 1.2 Hz, H-3); 7.83 (m, 2H, H-3′); 7.70 (d, 1H, *J*(2,3) = 1.3 Hz, H-2); 7.69 (m, 2H, H-2′); 6.77 (s, 1H, H-7); 3.16 (m, 4H, NCH_2_); 1.69 (m, 4H, CH_2_-pyrrol.) ppm. ^13^C NMR (DMSO-*d*_6_): δ = 147.43 (C-6); 142.90 (C-1′); 139.50 (C-8); 132.26 (C-9); 131.46 (C-2); 131.17 (C-4′); 128.89 (C-3′); 121.82 (C-2′); 118.25 (C-3); 94.65 (C-7); 47.81 (NCH_2_); 24.72 (CH_2_-pyrrol.) ppm. HRMS (ESI): *m*/*z* calculated for C_16_H_17_O_2_N_5_ClS 378.07860, found 378.07837.

#### *N*^6^-((1*r*,4*r*)-4-Aminocyclohexyl)-3-(1*H*-pyrazole-4-yl)-*N*^8^-(4-(1-pyrrolidinylsulfonyl)phenyl)imidazo[1,2-*b*]pyridazine-6,8-diamine (**42a**)

Prepared from **41a** according to **GP2** and isolated as a salt with formic acid. White solid, yield 60%. MS (ESI): *m*/*z* = 522.4 [M + H]^+^. ^1^H NMR (DMSO-*d*_6_): δ = 9.44 (bs, 1H NH); 8.46 (s, 1H, HCOOH); 8.27 (s, 2H, NH_2_); 7.77 (m, 2H, H-3′); 7.66 (s, 1H, H-2); 7.61 (m, 2H, H-2′); 6.61 (d, 1H, *J* = 6.8 Hz, NH); 6.44 (s, 1H, H-7); 6.61 (d, 1H, *J*(NH,CH) = 6.8 Hz, NH); 6.44 (s, 1H, H-7); 3.62 (m, 1H, H-1″); 3.15 (m, 4H, NCH_2_); 3.02 (m, 1H, H-4″); 2.20 (m, 2H, H-2″a); 2.03 (m, 2H, H-3″a); 1.68 (m, 4H, CH_2_-pyrrol.); 1.51 (m, 2H, H-3″b); 1.27 (m, 2H, H-2″b) ppm. ^13^C NMR (DMSO-*d*_6_): δ = 165.67 (HCOOH); 154.33 (C-6); 144.79 (C-1′); 137.01 (C-8); 131.42 (C-9); 128.91 (C-4′); 128.81 (C-4′); 125.18 (C-2); 122.62 (C-3); 119.91 (C-2′); 110.02 (C-1‴); 87.52 (C-7); 49.36 (C-4″); 48.73 (C-1″); 47.82 (NCH_2_); 29.88 (C-2″); 29.80 (C-3″); 24.73 (CH_2_-pyrrol.) ppm. HRMS (ESI): *m*/*z* calculated for C_25_H_32_O_2_N_9_S 522.23942, found 522.23932.

#### *N*^6^-((1*r*,4*r*)-4-Aminocyclohexyl)-*N*^8^-(4-(1-pyrrolidinylsulfonyl)phenyl)imidazo[1,2-*b*]pyridazine-6,8-diamine (**42b**)

Prepared from **41b** according to **GP2**. White solid, yield 46%. MS (ESI): *m*/*z* = 456.37 [M + H]^+^. ^1^H NMR (DMSO-*d*_6_): δ = 8.46 (s, 1H, FA); 7.79 (d, 1H, *J*(3,2) = 1.1 Hz, H-3); 7.75 (m, 2H, H-3′); 7.59 (m, 2H, H-2′); 7.32 (d, *J*(2,3) = 1.1 Hz, H-2); 6.45 (d, 1H, *J*(NH,1″) = 7.4 Hz, NH); 3.51 (dtt, 1H, *J* = 7.4, 3.6, 11.0 Hz, H-1″); 3.14 (m, 4H, NCH_2_); 2.91 (tt, 1H, *J* = 11.4, 4.0 Hz, H-4″); 2.06 (dd, 2H, *J* = 13.0, 4.0 Hz, H-2″a); 1.95 (m, 2H, H-3″a); 1.67 (m, 4H, CH_2_-pyrrol.); 1.39 (m, 2H, H-3″b); 1.21 (m, 2H, H-2″b) ppm. ^13^C NMR (DMSO-*d*_6_): δ = 165.83 (FA); 154.42 (C-6); 144.72 (C-1′); 136.88 (C-8); 131.64 (C-9); 128.98 (C-4′); 128.79 (C-3′); 128.58 (C-2); 120.00 (C-2′); 117.23 (C-3); 88.43 (C-7); 48.69 (C-4′); 48.45 (C-1′); 47.81 (NCH_2_); 30.23 and 30.19 (C-2″, C-3″); 24.72 (CH_2_-pyrrol.) ppm. HRMS (ESI): *m*/*z* calculated for C_22_H_30_O_2_N_7_S 456.21762, found 456.21756.

#### 8-Bromo-6-chloro-3-phenylimidazo[1,2-*b*]pyridazine (**43**)

Phenylacetaldehyde (4 g, 33.28 mmol) in 1,4-dioxane (10 mL) was slowly treated with bromine (1.79 mL, 35 mmol) at 0 °C and left to warm to RT. The mixture was then diluted with DCM, washed with sat. Na_2_S_2_O_3_ and water, dried over MgSO_4_, filtered, and evaporated to half its volume. The dark brown residue was treated with 3-amino-4-bromo-6-chloropyridazine (5 g, 24 mmol), after which the mixture was refluxed overnight, evaporated, and purified by FC (c-hex/EtOAc/MeOH). Crystallization from the EtOAc and c-hex mixture afforded a yellow crystalline product (2.3 g, 49%). MS (ESI): *m*/*z* = 308.03 [M + H]^+^. ^1^H NMR (DMSO-*d*_6_): δ = 8.33 (s, 1H, H-2); 8.06 (m, 2H, H-3′); 7.97 (s, 1H, H-7); 7.55 (m, 2H, H-2′); 7.45 (m, 1H, H-4′) ppm. ^13^C NMR (DMSO-*d*_6_): δ = 145.27 (C-6); 137.63 (C-9); 133.68 (C-2); 129.53 (C-3); 128.82 (C-3′); 128.58 (C-4′); 127.44 (C-1′); 126.65 (C-2′); 124.20 (C-8); 120.81 (C-7) ppm. HRMS (ESI): *m*/*z* calculated for C_12_H_8_N_3_BrCl 307.95846, found 307.95865.

### Kinase-Inhibitory Assays

FLT3-ITD and FLT3-D835Y were purchased from ProQinase, FLT3-ITD-F691L was purchased from Signalchem, and CDK2/Cyclin E was produced in Sf9 insect cells in-house. Activities of compounds against recombinant kinases were analyzed as described previously.^12,42^ Briefly, the kinase reactions were assayed with the peptide substrate (1 mg/mL AGLT (poly(Ala, Glu, Lys, Tyr) 6:2:5:1 hydrobromide)) for FLT3-ITD and FLT3-D835Y, with 1 mg/mL myelin basic protein for FLT3-ITD-F691L or with 1 mg/mL histone H1 for CDK2 in the presence of 1/1/12.5/15 μM ATP (for FLT3-ITD/D835Y/ITD-F691L/CDK2), 0.05 μCi [γ-^33^P]ATP, and the test compound in a final volume of 10 μL in a reaction buffer for FLT3-ITD, FLT3-D835Y, and CDK2 (60 mM HEPES–NaOH, pH 7.5, 3 mM MgCl_2_, 3 mM MnCl_2_, 3 μM Na-orthovanadate, 1.2 mM DTT, 2.5 μg/50 μL PEG_20.000_) or for FLT3-ITD-F691L (40 mM Tris-HCl, pH 7.4, 20 mM MgCl_2_, 0.1 mg/mL BSA, 50 μM DTT).

The protein kinase selectivity of compound **34f** at a single concentration (100 nM) against 48 enzymes, as well as the determination of IC_50_ values for the relevant off-targets, was determined at Eurofins Discovery. The kinome tree was generated using KinMap.^43^ Illustration reproduced courtesy of Cell Signaling Technology, Inc. (www.cellsignal.com).

### Cell Culture and Proliferation Assay

Human cell lines were obtained from the German Collection of Microorganisms and Cell Cultures (MOLM-13, NOMO-1, ML-2), the Cell Lines Service (MV4-11), and the European Collection of Authenticated Cell Cultures (CEM, K562), and were cultivated according to the providers’ instructions. Briefly, MV4-11, MOLM-13, CEM, NOMO-1, and ML-2 were maintained in RPMI-1640 medium, SEM in IMDM, and K562 in DMEM. The cell culture medium was supplemented with 10% fetal bovine serum, penicillin (100 U/mL), and streptomycin (100 μg/mL); cells were cultivated at 37 °C in 5% CO_2_. A MOLM-13-resistant cell line (provided by Prof. Julhash Uddin Kazi) was obtained by long-term exposure to increasing concentrations of sorafenib (up to 1 μM); NGS revealed an FLT3-ITD-D835Y mutation (data not shown).

For antiproliferative assays, cells were seeded into 96-well plates in appropriate densities and subsequently treated with test compounds. After the incubation period, resazurin (Merck) solution was added for 4 h. Fluorescence of resorufin corresponding to live cells was measured at 544/590 nm (excitation/emission) using a Fluoroskan Ascent microplate reader (Labsystems).

### Cell Cycle Analysis

Leukemia cells were seeded and, after a preincubation period, treated with tested compounds for 24 h. After staining with propidium iodide, DNA content was analyzed by flow cytometry using a 488 nm laser (BD FACSVerse with BD FACSuite software, version 1.0.6). Cell cycle distribution was analyzed using ModFit LT (Verity Software House).

### Immunoblotting

After the preparation of cell lysates, proteins were separated on sodium dodecyl sulfate (SDS)-polyacrylamide gels and electroblotted onto nitrocellulose membranes. After blocking, proteins were incubated first with specific primary antibodies and subsequently with peroxidase-conjugated secondary antibodies. Peroxidase activity was detected using SuperSignal West Pico reagents (Thermo Fisher Scientific) and the LAS-4000 CCD camera system (Fujifilm). The following specific antibodies were purchased from Cell Signaling Technology: anti-FLT3 (8F2), anti-phospho-FLT3 Y589/591 (30D4), anti-STAT5 (D2O6Y), anti-phospho-STAT5 Y694, anti-ERK1/2, anti-phospho-ERK1/2 (T202/Y204), anti-c-KIT (D13A2), anti-phospho-c-KIT Y703 (D12E12), and anti-phospho-c-KIT Y719. Anti-PCNA (clone PC-10) was generously gifted by Dr. Bořivoj Vojtěšek.

### RNA Isolation and qPCR

Total RNA was isolated using the RNeasy Plus Mini Kit (QIAGEN) according to the manufacturer’s instructions. RNA concentration and purity were measured using the DS-11 Series Spectrophotometer (DeNovix). RNA was transcribed into first-strand cDNA using the SensiFAST cDNA Synthesis Kit (Bioline). Quantitative RT-PCR was carried out on the CFX96 Touch Real-Time PCR Detection System (Bio-Rad) and the SensiFAST SYBR No-ROX Kit (Bioline). Suitable primers were designed using Primer-BLAST^44^ and synthesized by Generi Biotech. Primary data were analyzed using CFX Maestro Software 2.2 (Bio-Rad). Relative gene expressions were determined using the delta–delta Ct method.^45^ Expression of the MYC gene was normalized per the GAPDH and RPL13 genes, which were determined to be the most stable according to CFX Maestro Software 2.2 (Bio-Rad).

Primers used: GAPDH (F: TCCAAAATCAAGTGGGGCGA; R: TGGTTCACACCCATGACGAA), MYC (F: TACAACACCCGAGCAAGGAC; R: AGCTAACGTTGAGGGGCATC), RPL13A (F: CGACAAGAAAAAGCGGATGG; R: TTCTCTTTCCTCTTCTCCTCC).

### Plasma Stability Assay

To determine plasma stability, 5 μM of the given compound was incubated with human pooled plasma from 50 donors (Biowest) for 20, 60, and 120 min at 37 °C. The reactions were terminated by adding four volumes of ice-cold methanol. The samples were then mixed vigorously and left at −20 °C for 30 min before being centrifuged. The supernatants were diluted with four volumes of 30% methanol in water and then analyzed using the Echo MS system (SCIEX). Zero time points were prepared by adding ice-cold methanol to the compound prior to the addition of plasma.

### Microsomal Stability Assay

A microsomal stability assay was performed using 0.5 mg/mL of pooled human liver microsomes (Thermo Fisher Scientific) and 5 μM compounds in 90 mM TRIS-Cl buffer (pH 7.4) containing 2 mM NADPH and 2 mM MgCl_2_ for 10, 30, and 45 min at 37 °C. The reactions were terminated by the addition of four volumes of ice-cold methanol, mixed vigorously, and left at −20 °C for 30 min; the samples were then centrifuged. The supernatants were diluted with four volumes of 30% methanol in water and then analyzed using the Echo MS system (SCIEX). Zero time points were prepared by adding ice-cold methanol to the mixture of compounds and cofactors prior to the addition of microsomes.

### In Vivo Efficacy

The experimental design was approved by the Institutional Animal Care and Use Committee (Charles University, MSMT-37334/2020-4). Immunodeficient adult female NOD.Cg-PrkdcscidIl2rgtm1Wjl/SzJ mice (referred to as NSG mice) purchased from the Jackson Laboratory were preserved in a pathogen-free environment in individually ventilated cages and provided with sterilized food and water. NSG mice were subcutaneously inoculated with MV4-11 cells. After all mice developed palpable tumors, they were stratified into cohorts (6 mice per group) with comparable calculated tumor volumes. Therapy involving **34f** (5 and 10 mg/kg dissolved in 5% DMSO in saline, intraperitoneal administration, final volume 500 μL per mouse) was then initiated. Three perpendicular dimensions (in millimeters) were measured with a digital caliper. Tumor volumes were calculated using the following formula: π/6 × length × width × height. The experiment was terminated when the tumors reached a maximum diameter of 20 mm. For the purpose of immunoblotting analysis, mice that had developed tumors were treated with a 10 mg/kg intraperitoneal dose of **34f** for 6 or 24 h. The mice were then euthanized, and the tumors were processed for further analysis.

## Supporting Information Available

Activities of purine derivatives and intermediates; kinase-inhibitory and antiproliferative activities with standard deviations; additional in vitro data with quizartinib, gilteritinib, and **34f**; further kinase selectivity profiling; microsomal clearance; pharmacokinetic study; NMR spectra; and HPLC traces (PDF)
**29a** docked to FLT3 (PDB)
**34f** docked to FLT3 (PDB)
Molecular formula strings (CSV)

## Supplementary Material

jm3c00575_si_001.pdf

jm3c00575_si_002.pdb

jm3c00575_si_003.pdb

## Abbreviations Used

ACN: acetonitrile
AML: acute myeloid leukemia
CDK: cyclin-dependent kinase
EGFR: epidermal growth factor receptor
FA: formic acid
FC: flash column chromatography
FLT3: FMS-like tyrosine kinase 3
ITD: internal tandem duplication
JAK: Janus kinase
MAPK: mitogen-activated protein kinase
PARP-1: poly(ADP-ribose) polymerase 1
PDGFR: platelet-derived growth factor receptor
PI3K: phosphatidylinositol 3-kinase
RP FC: reversed-phase flash column chromatography
STAT: signal transducer and activator of transcription
TRK: tropomyosin receptor kinase
UPLC: ultra-performance liquid chromatography
