# Supplementary material for: Inhibition of FLT3-ITD Kinase in Acute Myeloid Leukemia by New Imidazo[1,2-b]pyridazine Derivatives Identified by Scaffold Hopping
Source: J Med Chem. 2023 Aug 3;66(16):11133–57. doi: 10.1021/acs.jmedchem.3c00575 (PMC10461230; doi:10.1021/acs.jmedchem.3c00575)
Supplement: Supplementary file 1 — jm3c00575_si_001.pdf [file jm3c00575_si_001.pdf]

Supporting Information

Inhibition of FLT3-ITD Kinase in Acute Myeloid Leukemia by New Imidazo[1,2-*b*]pyridazine Derivatives Identified by Scaffold Hopping

Petra Břehová,[a,#] Eva Řezníčková,[b,#] Kryštof Škach,[a] Radek Jorda,[b] Milan Dejmek,[a] Veronika Vojáčková,[b] Michal Šála,[a] Markéta Kovalová,[b] Martin Dračínský,[a] Alexandra Dolníková,[c] Timotej Strmeň,[a] Monika Kinnertová,[b] Karel Chalupský,[a] Alexandra Dvořáková,[a] Tomáš Gucký,[b] Helena Mertlíková Kaiserová,[a] Pavel Klener,[c] Radim Nencka,[a,*] Vladimír Kryštof[b,d,*]

[a] Institute of Organic Chemistry and Biochemistry of the Czech Academy of Sciences, Flemingovo nám. 2, 16000 Prague, Czech Republic

[b] Department of Experimental Biology, Faculty of Science, Palacký University Olomouc, Šlechtitelů 27, 78371 Olomouc, Czech Republic

[c] Institute of Pathological Physiology, First Faculty of Medicine, Charles University, 12108 Prague, Czech Republic

[d] Institute of Molecular and Translational Medicine, Faculty of Medicine and Dentistry, Palacký University Olomouc, Hněvotínská 5, 77900 Olomouc, Czech Republic

[#] Both authors contributed equally.

Corresponding Author

*E-mail: vladimir.krystof@upol.cz. Phone: +420 585 634 854

*E-mail: radim.nencka@uochb.cas.cz. Phone: +420 220 183 265

Table of Contents

Table S1. Previously Described CDK2 Inhibitors with FLT3-Inhibitory Potency S3

Table S2: Docking scores of the most successful binders S4

Table S3. Kinase-Inhibitory and Antiproliferative Activities of Novel Compounds................... S5

Table S4. Kinase-Inhibitory and Antiproliferative Activities of Derivatives with an Imidazo[1,2-*b*]pyridazine Core and 6-Chloro Substitution.. S6

Figure S1 and S2. Cell cycle analysis of MV4-11 and NOMO-1 cells treated with quizartinib and gilteritinib S7

Figure S3 and S4. Immunoblotting analysis of FLT3 and its downstream signaling pathways in MV4-11 treated with quizartinib and gilteritinib. Relative normalized expression of the MYC gene in MV4-11 and NOMO-1 cells treated with quizartinib and gilteritinib S8

Table S5: Kinase selectivity profiling of **34f** (IC_{50} values for selected off-targets) S9

Figure S5. Immunoblotting analysis of KIT and its downstream signaling pathway in Kasumi-1 treated with **34f**... S9

Table S6: Microsomal clearance CL_{int} S9

Figure S6: Concentration profile of **34f** in mouse plasma.. S10

Table S7: Pharmacokinetic parameters of **34f**.. S10

Pharmacokinetic study S10

NMR spectra and LC MS traces S12-S59

Table S1. Previously Described CDK2 Inhibitors with FLT3-Inhibitory Potency

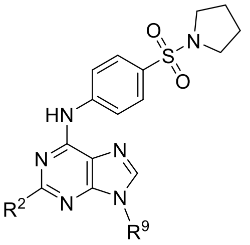

CDK2 inhibitors
(see Table S1)

Code	R2	R9	FLT3 WT IC 50 (nM)	CDK2 IC50 (μM)
MS760	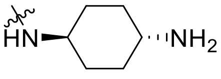	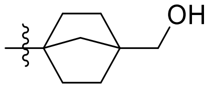	44.4 ±41.4	0.22
MD610	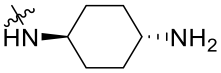	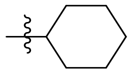	78.9 ±69.5	0.43
MD637-1	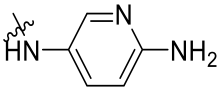	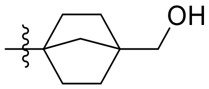	87.4 ±22.2	2.79
MD636-1	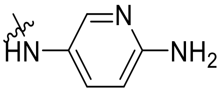	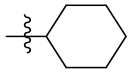	56.4 ±31.0	14.84

Table S2: Docking scores of the most successful binders

	R	Docking score
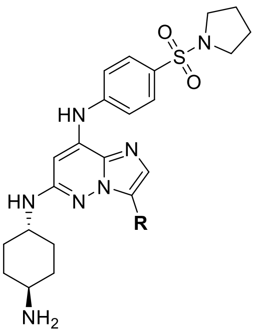	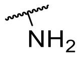	-6.713
	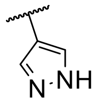	-6.962
	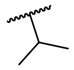	-7.961
	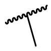	-8.147
	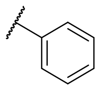	-8.369
	Br	-8.624
	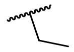	-8.916

Table S3. Kinase-Inhibitory and Antiproliferative Activities of Novel Compounds

	IC_{50} ± SD (μM)			GI_{50} ± SD (μM)						
	**FLT3-ITD**	**FLT3-D835Y**	**CDK2**	**MV4-11**	**MOLM-13**	**SEM**	**CEM**	**NOMO-1**	**ML-2**	**K562**
**4a**	>20	NT	>20	1.765 ± 1.082	3.260 ± 2.206	>6.25	>6.25	>10	>10	9.890 ± 0.028
**4b**	>20	NT	>20	2.570 ± 0.820	6.010 ± 2.333	4.400 ± 0.283	>6.25	8.945 ± 0.785	6.775 ± 0.728	8.533 ± 0.605
**5a**	5.098 ± 2.072	NT	17.204 ± 2.804	1.665 ± 0.191	2.180 ± 0.283	1.467 ± 0.426	7.490 ± 0.790	8.030 ± 0.509	4.243 ± 1.390	6.595 ± 0.573
**5b**	>20	NT	>20	2.705 ± 0.021	6.075 ± 2.143	2.595 ± 0.191	>10	>10	7.335 ± 3.769	>10
**9**	>20	NT	>20	7.360 ± 2.659	3.285 ± 0.615	1.575 ± 0.049	3.595 ± 0.431	>10	>10	>10
**10a**	10.612 ± 0.278	NT	>20	>10	>10	>10	>10	>10	>10	>10
**10b**	2.507 ± 1.283	NT	>20	5.737 ± 1.455	3.280 ± 0.919	9.840 ± 1.188	>10	>10	>10	>10
**11**	0.540 ± 0.181	0.109 ± 0.077	0.774 ± 0.122	1.817 ± 0.485	1.755 ± 0.078	5.510 ± 0.014	>10	>10	>10	>10
**14**	0.623 ± 0.178	0.272 ± 0.117	0.100 ± 0.018	0.690 ± 0.075	1.020 ± 0.042	7.905 ± 0.530	>10	>10	>10	>10
**17**	2.453 ± 0.738	NT	>20	5.520 ± 0.424	3.075 ± 0.431	>10	>10	>10	9.530 ± 0.665	>10
**18a**	0.430 ± 0.254	0.479 ± 0.220	>20	1.877 ± 0.606	1.540 ± 0.668	2.380 ± 0.099	>10	>10	>10	>10
**18b**	0.134 ± 0.029	0.392 ± 0.028	>20	0.735 ± 0.007	0.335 ± 0.035	1.565 ± 0.049	>10	8.565 ± 1.585	5.835 ± 0.785	8.180 ± 0.127
**23**	>20	NT	>20	3.900 ± 0.283	1.675 ± 0.191	1.340 ± 0.170	3.365 ± 0.332	7.510 ± 1.117	8.295 ± 0.686	8.710 ± 0.028
**24**	>20	NT	>20	>10	>10	>10	>10	>10	>10	>10
**25**	1.907 ± 0.274	NT	>20	6.560 ± 1.896	4.520 ± 0.113	7.350 ± 1.428	>10	>10	>10	9.530 ± 0.665
**29a**	0.002 ± 0.000	0.002 ± 0.002	0.003 ± 0.000	0.0001 ± 0.000	0.004 ± 0.003	0.008 ± 0.001	0.623 ± 0.182	0.240 ± 0.028	0.118 ± 0.006	0.320 ± 0.042
**29b**	0.005 ± 0.004	0.004 ± 0.003	0.037 ± 0.010	0.001 ± 0.000	0.024 ± 0.013	0.225 ± 0.035	1.073 ± 0.263	1.380 ± 0.184	0.596 ± 0.037	0.722 ± 0.124
**30**	0.006 ± 0.006	0.012 ± 0.007	0.211 ± 0.049	0.279 ± 0.132	0.070 ± 0.014	0.655 ± 0.007	1.910 ± 0.014	4.875 ± 1.319	3.100 ± 1.062	1.363 ± 0.188
**34a**	0.007 ± 0.008	0.004 ± 0.001	0.196 ± 0.033	0.005 ± 0.002	0.007 ± 0.007	0.372 ± 0.092	3.800 ± 0.386	8.430 ± 2.475	5.780 ± 0.566	6.870 ± 1.429
**34b**	0.005 ± 0.002	0.002 ± 0.001	0.005 ± 0.001	0.008 ± 0.001	0.007 ± 0.001	0.235 ± 0.028	0.917 ± 0.076	0.620 ± 0.000	0.480 ± 0.124	0.885 ± 0.007
**34c**	0.008 ± 0.004	0.002 ± 0.001	0.083 ± 0.011	0.028 ± 0.011	0.030 ± 0.007	0.453 ± 0.091	3.727 ± 0.546	2.435 ± 0.092	1.078 ± 0.330	1.725 ± 0.064
**34d**	0.004 ± 0.000	0.001 ± 0.001	0.011 ± 0.001	0.005 ± 0.003	0.009 ± 0.002	0.210 ± 0.026	0.810 ± 0.144	0.848 ± 0.302	0.490 ± 0.071	0.635 ± 0.120
**34e**	0.009 ± 0.002	0.002 ± 0.000	0.093 ± 0.008	0.005 ± 0.003	0.007 ± 0.001	0.132 ± 0.035	0.683 ± 0.099	0.700 ± 0.099	0.455 ± 0.035	1.310 ± 0.170
**34f**	0.004 ± 0.002	0.001 ± 0.000	0.493 ± 0.091	0.007 ± 0.004	0.009 ± 0.006	0.140 ± 0.051	1.768 ± 0.261	4.275 ± 0.818	3.030 ± 0.311	1.525 ± 0.078
**34g**	0.001 ± 0.001	0.002 ± 0.000	2.119 ±1.328	0.045 ± 0.019	0.040 ± 0.010	0.283 ± 0.088	2.645 ± 0.247	5.223 ± 2.979	3.505 ± 0.870	1.640 ± 0.211
**34h**	0.002 ± 0.000	0.002 ± 0.001	0.961 ± 0.325	0.073 ± 0.027	0.176 ± 0.082	1.060 ± 0.311	6.170 ± 2.650	>10	6.865 ± 0.035	1.010 ± 0.594
**34i**	0.002 ± 0.000	0.006 ± 0.000	1.435 ± 0.419	0.060 ± 0.017	0.027 ± 0.003	0.877 ± 0.231	6.380 ± 1.540	5.085 ± 3.302	4.220 ± 0.113	2.255 ± 0.247
**34j**	0.001 ± 0.000	0.001 ± 0.001	0.234 ± 0.001	0.023 ± 0.013	0.030 ± 0.010	0.150 ± 0.028	1.690 ± 0.269	5.030 ± 1.103	2.050 ± 0.269	1.063 ± 0.398
**34k**	0.001 ± 0.000	0.002 ± 0.000	0.037 ± 0.017	0.017 ± 0.009	0.019 ± 0.003	0.590 ± 0.113	1.940 ± 0.390	0.865 ± 0.007	0.325 ± 0.035	2.120 ± 0.184
**34l**	0.004 ± 0.002	0.011 ± 0.001	1.443 ± 0.506	0.156 ± 0.023	0.200 ± 0.059	1.031 ± 0.098	1.570 ± 1.270	5.025 ± 1.867	3.923 ± 0.537	1.505 ± 0.064
**34m**	0.013 ± 0.000	0.025 ± 0.003	6.587 ± 0.725	0.790 ± 0.028	0.410 ± 0.056	1.335 ± 0.120	4.750 ± 1.770	4.260 ± 1.613	3.310 ± 0.637	1.550 ± 0.042
**34n**	0.001 ± 0.000	0.002 ± 0.000	0.366 ± 0.028	0.042 ± 0.013	0.025 ± 0.005	0.168 ± 0.053	0.760 ± 0.028	0.393 ± 0.011	0.245 ± 0.035	1.655 ± 0.134
**34o**	0.231 ± 0.029	0.569 ± 0.136	3.428 ± 0.400	0.710 ± 0.140	0.935 ± 0.021	2.525 ± 0.148	8.990 ± 0.660	9.035 ± 0.092	4.475 ± 3.128	>10
**34p**	0.142 ± 0.028	0.834 ± 0.302	2.435 ± 0.177	1.183 ± 0.223	1.160 ± 0.310	1.500 ± 0.608	8.400 ± 0.099	2.385 ± 0.191	2.000 ± 0.127	4.128 ± 1.784
**34q**	0.001 ± 0.000	0.003 ± 0.002	0.082 ± 0.022	0.002 ± 0.002	0.004 ± 0.000	0.381 ± 0.003	1.400 ± 0.240	1.455 ± 0.007	0.440 ± 0.014	1.470 ± 0.007
**34r**	0.001 ± 0.000	0.002 ± 0.001	0.031 ± 0.004	0.001 ± 0.001	0.001 ± 0.000	0.399 ± 0.045	1.030 ± 0.410	0.755 ± 0.064	0.270 ± 0.057	0.570 ± 0.085
**39**	0.333 ± 0.089	0.268 ± 0.038	1.116 ± 0.292	1.557 ± 0.156	2.213 ± 0.530	>10	>10	>10	>10	>10
**42a**	0.002 ± 0.000	0.001 ± 0.000	0.031 ± 0.017	0.006 ± 0.002	0.011 ± 0.005	0.823 ± 0.301	7.363 ± 1.523	1.745 ± 0.092	0.370 ± 0.042	9.850 ± 0.212
**42b**	0.106 ± 0.064	0.014 ± 0.002	0.178 ± 0.018	0.039 ± 0.007	0.097 ± 0.029	0.523 ± 0.093	1.423 ± 0.307	4.733 ± 2.586	3.650 ± 0.782	5.545 ± 0.669

NT = not tested.

Table S4. Kinase-Inhibitory and Antiproliferative Activities of Derivatives with an Imidazo[1,2-*b*]pyridazine Core and 6-Chloro Substitution

	IC_{50} ± SD (μM)			GI_{50} ± SD (μM)			
	**FLT3-ITD**	**FLT3-D835Y**	**CDK2**	**MV4-11**	**MOLM-13**	**SEM**	**CEM**
**33a**	5.335 ± 0.204	NT	18.635 ± 1.874	5.330 ± 3.017	6.827 ± 1.557	>25	>25
**33e**	4.218 ± 0.263	NT	0.166 ± 0.058	3.840 ± 1.152	3.383 ± 0.833	3.780 ± 0.789	5.563 ± 0.706
**33d**	2.189 ± 0.308	NT	0.192 ± 0.024	4.200 ± 0.941	3.457 ± 1.119	7.597 ± 2.332	8.333 ± 3.608
**33f**	0.475 ± 0.325	0.878 ± 0.313	>20	2.168 ± 2.141	2.163 ± 1.810	>6.25	>6.25
**33g**	0.081 ± 0.061	NT	>20	2.355 ± 1.371	1.400 ± 0.505	>10	>10
**33h**	0.865 ± 0.185	NT	1.597 ± 0.997	6.008 ± 3.707	6.333 ± 3.178	>10	>10
**33i**	0.150 ± 0.007	NT	0.217 ± 0.100	2.060 ± 0.765	1.830 ± 0.753	>10	>10
**33j**	0.295 ± 0.129	NT	0.364 ± 0.169	2.560 ± 1.025	2.893 ± 2.241	>10	>10
**33k**	0.419 ± 0.452	NT	3.013 ± 2.406	1.718 ± 0.569	1.140 ± 0.690	>10	>10
**33l**	1.317 ± 0.021	NT	1.131 ± 0.457	7.833 ± 2.909	6.590 ± 3.090	>10	>10
**33m**	0.685 ± 0.254	NT	>20	>10	8.953 ± 1.514	>10	>10
**33n**	0.644 ± 0.210	NT	2.037 ± 0.986	7.008 ± 3.495	4.423 ± 3.781	>10	>10
**33o**	0.685 ± 0.073	NT	0.646 ± 0.307	2.950 ± 0.401	3.838 ± 3.142	6.550 ± 0.382	9.330 ± 0.948
**33p**	0.529 ± 0.298	NT	0.541 ± 0.284	9.323 ± 1.355	4.980 ± 3.660	>10	>10
**33q**	0.085 ± 0.002	NT	0.192 ± 0.083	2.493 ± 1.260	2.653 ± 1.924	>10	>10
**33r**	0.076 ± 0.015	NT	0.440 ± 0.167	1.675 ± 0.137	1.308 ± 0.552	>10	>10
**41a**	0.542 ± 0.238	0.399 ± 0.188	0.937 ± 0.130	1.377 ± 0.263	0.970 ± 0.305	2.917 ± 0.231	>10
**41b**	12.546 ± 4.405	NT	1.345 ± 0.269	>10	9.017 ± 2.567	>10	>10

NT = not tested.

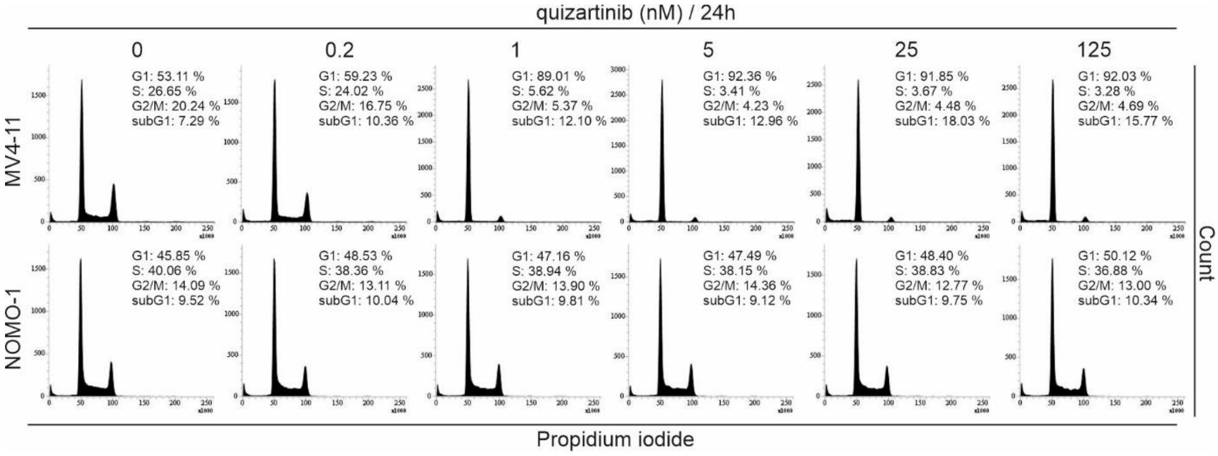

Figure S1. Cell cycle analysis of MV4-11 and NOMO-1 cells treated with quizartinib for 24 h.

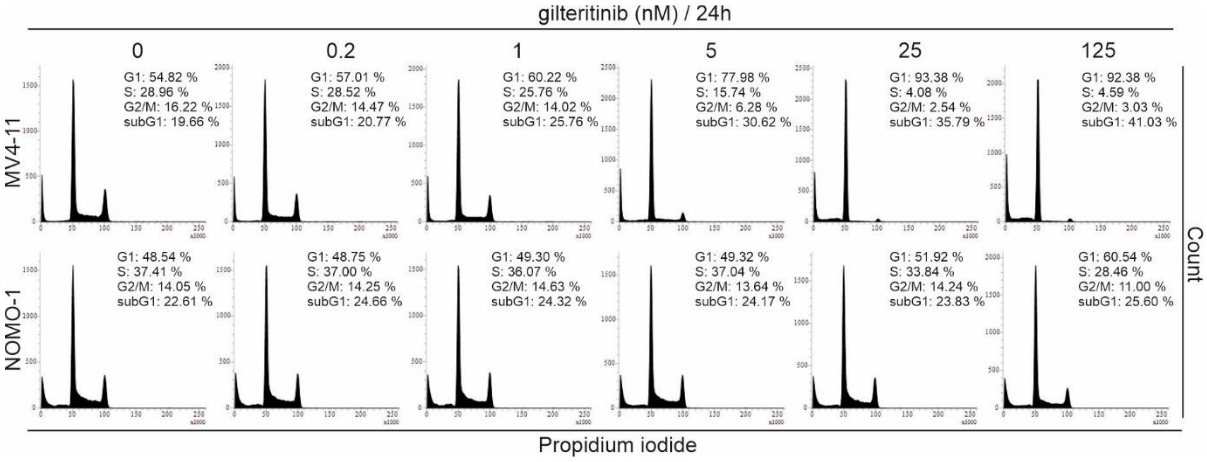

Figure S2. Cell cycle analysis of MV4-11 and NOMO-1 cells treated with gilteritinib for 24 h.

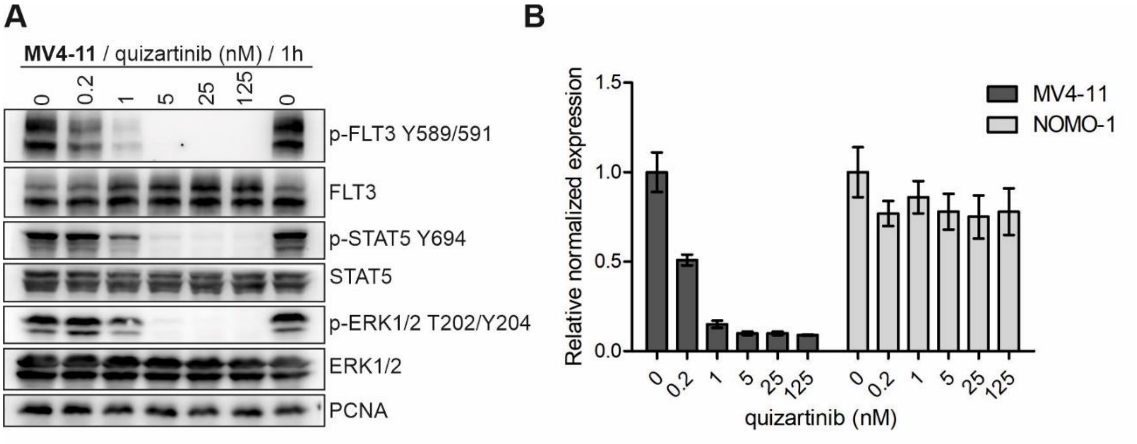

Figure S3. (A) Immunoblotting analysis of FLT3 and its downstream signaling pathways in MV4-11 treated with quizartinib for 1 h. (B) Relative normalized expression of the MYC gene in MV4-11 and NOMO-1 cells treated with quizartinib for 4 h.

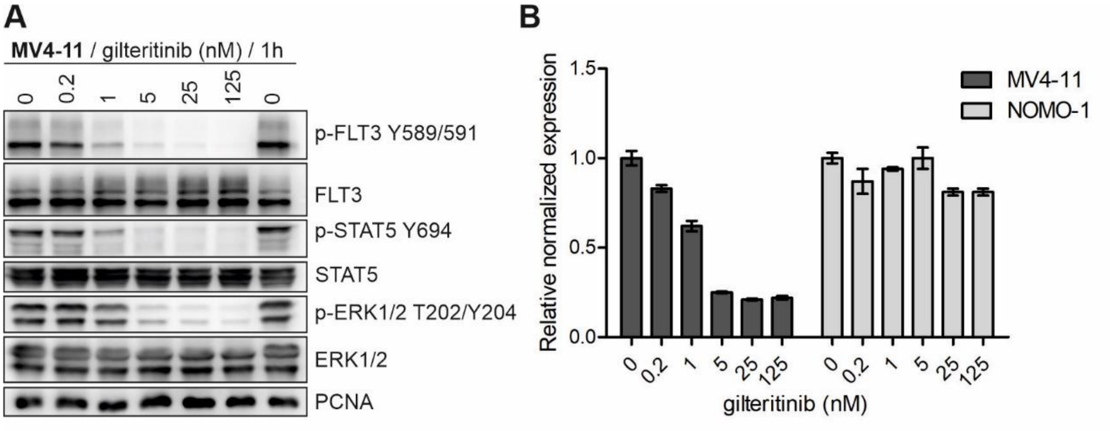

Figure S4. (A) Immunoblotting analysis of FLT3 and its downstream signaling pathways in MV4-11 treated with gilteritinib for 1 h. (B) Relative normalized expression of the MYC gene in MV4-11 and NOMO-1 cells treated with gilteritinib for 4 h.

Table S5: Kinase selectivity profiling of **34f** (IC_{50} values for selected off-targets).

**kinase**	**IC_{50} (nM)**
CaMKIIδ	4
KIT	680
Mer	17
SIK	8
Src(1-530	12
Yes	5

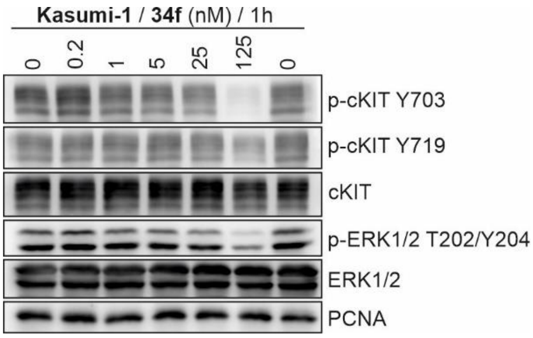

Figure S5. Immunoblotting analysis of KIT and its downstream signaling pathway in Kasumi-1 treated with **34f** for 1 h.

Table S6: Microsomal clearance CL_{int}.

	**Microsomal clearance CL_{int} (µL/min/mg)**	
	**human**	**mouse**
**34f**	18	13
verapamil	57	100

Figure S6: Concentration profile of **34f** in mouse plasma after intraperitoneal administration of 10 mg/kg.

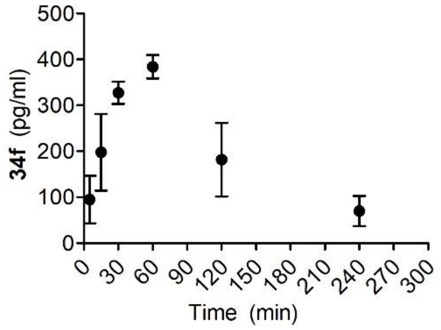

Table S7: Pharmacokinetic parameters of **34f** after intraperitoneal administration.

Parameter	Unit	Value
t1/2ka	min	24,20531
t1/2k10	min	47,2681
V/F	(mg/kg)/(pg/ml)	0,013597
CL/F	(mg/kg)/(pg/ml)/min	0,000199
Tmax	min	48,66447
Cmax	pg/ml	364,3407
AUC 0-t	pg/ml*min	47164,23
AUC 0-inf	pg/ml*min	50154,15
AUMC	pg/ml*min^2	5171612
MRT	min	103,1143

Pharmacokinetic study.

NOD.Cg-Prkdc^{scid} Il2rg^{tm1Wjl}/SzJ female mice were housed on a 12 h light and 12 h dark cycle with room temperature maintained at 22 ± 3 °C and relative humidity at 50 ± 20%. Animals were fasted 4 h before dosing. Water was provided ad libitum throughout the study. The animals (n = 3) were dosed with 10 mg/kg of **34f** formulated in 5% DMSO and saline by intraperitoneal injection. Blood samples were taken at 5, 15, 30, 60, 120 and 240 min after dosing and plasma was prepared for analysis. All samples were stored at −70 °C until analysis for side-by-side comparison. Three volumes of 80% acetonitrile with 150nM of caffeine as internal standard were added to one volume of plasma to precipitate proteins. Samples were centrifuged (20,000 g for 10 min), and supernatants were subjected to the analysis by LC−MS/MS. Calibration standards were made by preparation of a 1 mg/ml stock solution in 80% acetonitrile. LC separation was done on Synergi 4µm Fusion 50 × 2 mm column (Phenomenex) using a water/acetonitrile gradient with 0.1% formic acid starting from 5 to 90% for 8 min. MS/MS analysis

was performed on QTRAP 5500+ (Sciex) utilizing multiple reaction monitoring 532.3 to 515.3 for **34f** and 195.1 to 138.1 for caffeine detection. The area under the plasma concentration vs time curve was calculated using the linear trapezoidal method. Figure and analysis were generated in GraphPad Prism and PK solver.[1]

[1] Zhang, Y.; Huo, M.; Zhou, J.; Xie, S. PKSolver: An add-in program for pharmacokinetic and pharmacodynamic data analysis in Microsoft Excel. *Comput. Methods Programs Biomed*. **2010**, *99* (3), 306 – 314.

NMR spectra and LC MS traces

N[6]-((1r,4r)-4-aminocyclohexyl)-3-(1-cyclopenten-1-yl)-*N*[8]-(4-(1-pyrrolidinylsulfonyl)phenyl)imidazo[1,2-*b*]pyridazine-6,8-diamine (34a)

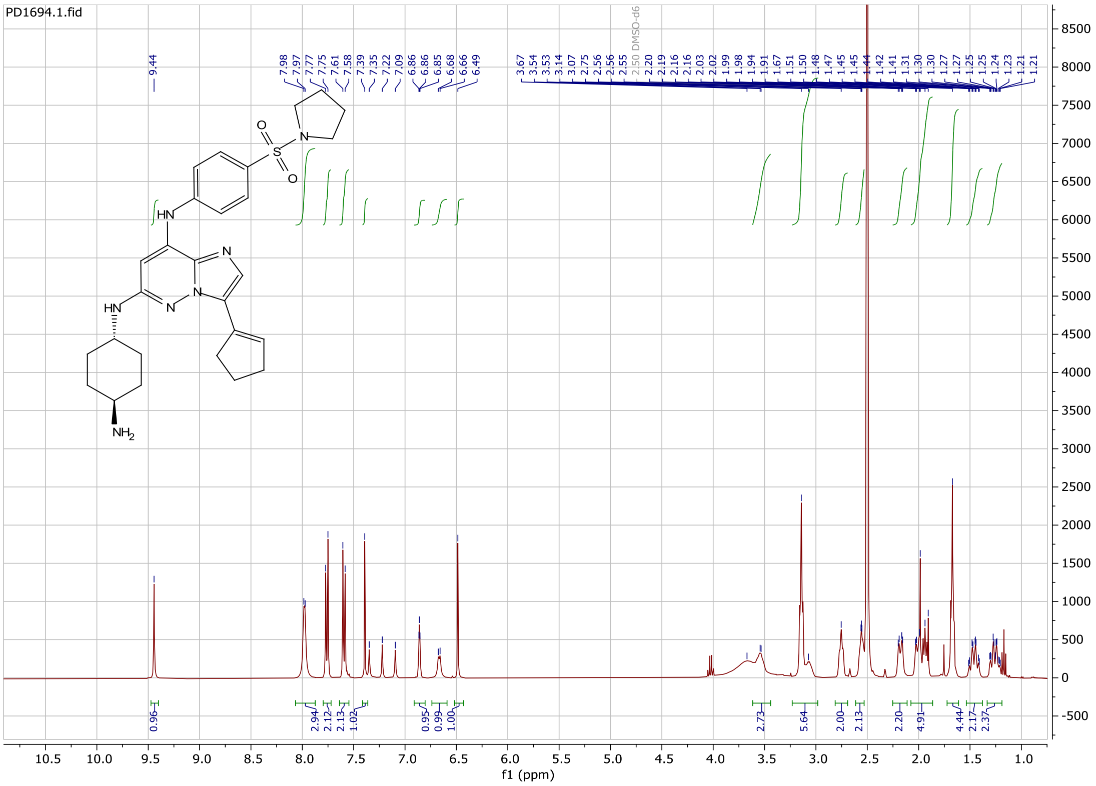

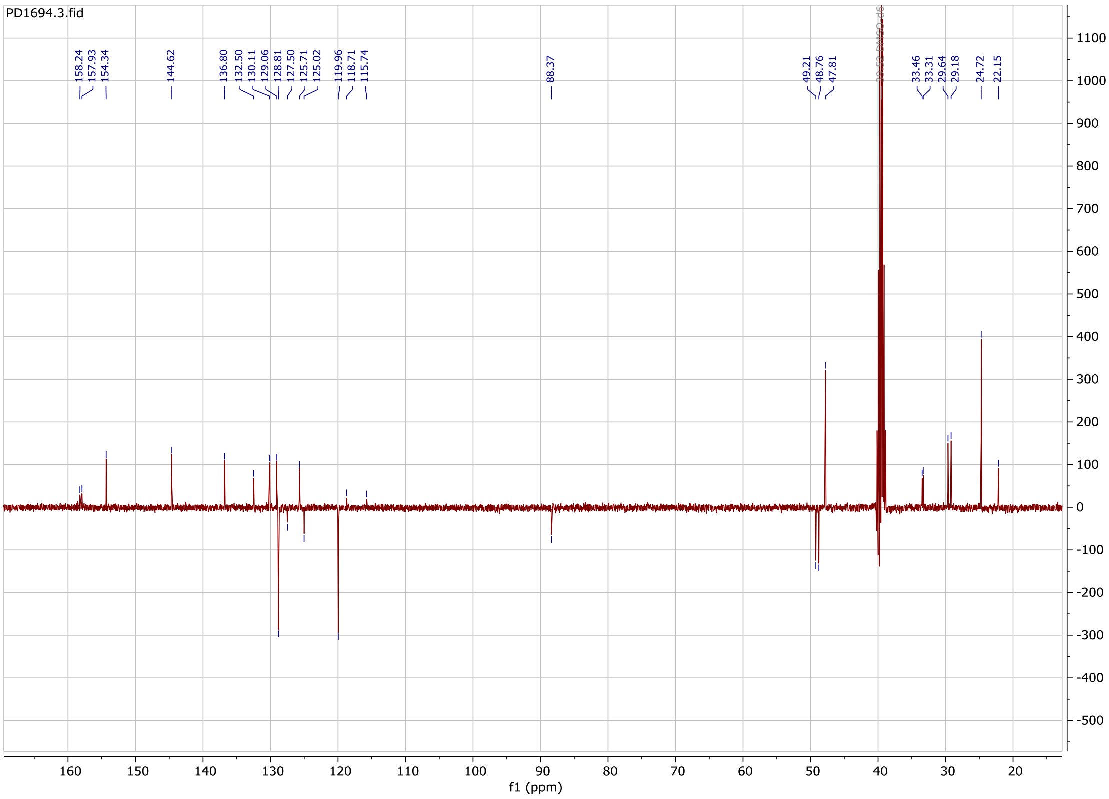

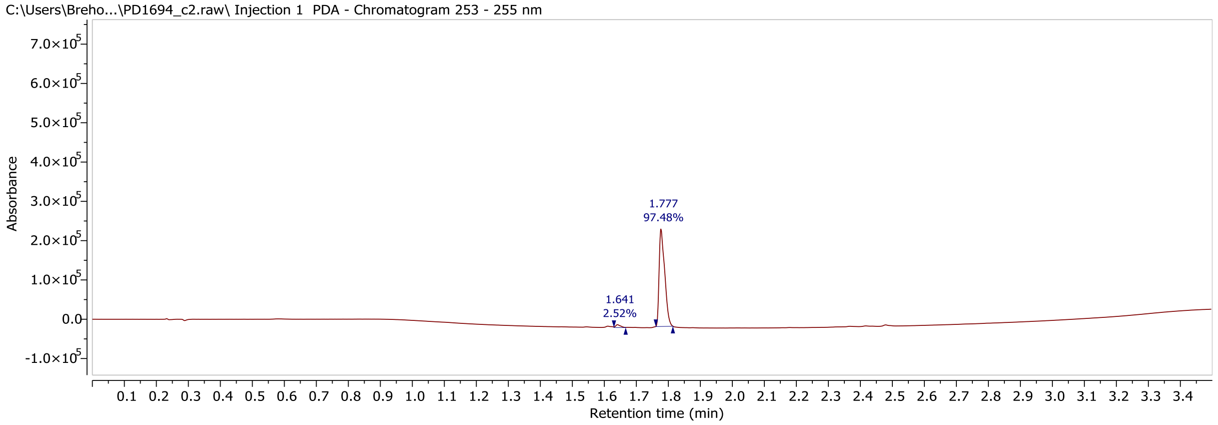

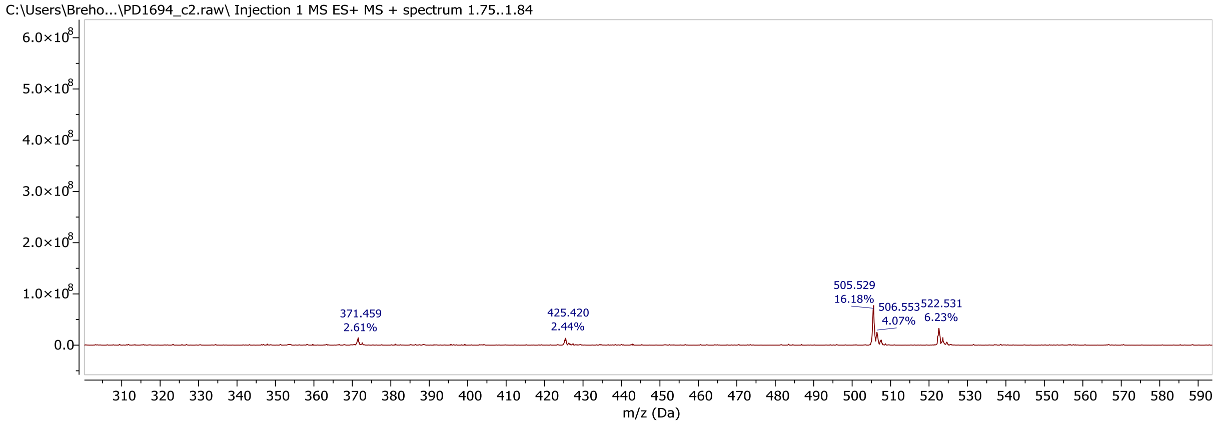

***N*^6^-((1r,4r)-4-aminocyclohexyl)-3-(cyclopentan-1-yl)-*N*^8^-(4-(1-pyrrolidinylsulfonyl)phenyl)imidazo[1,2-*b*]pyridazine-6,8-diamine (34b)**

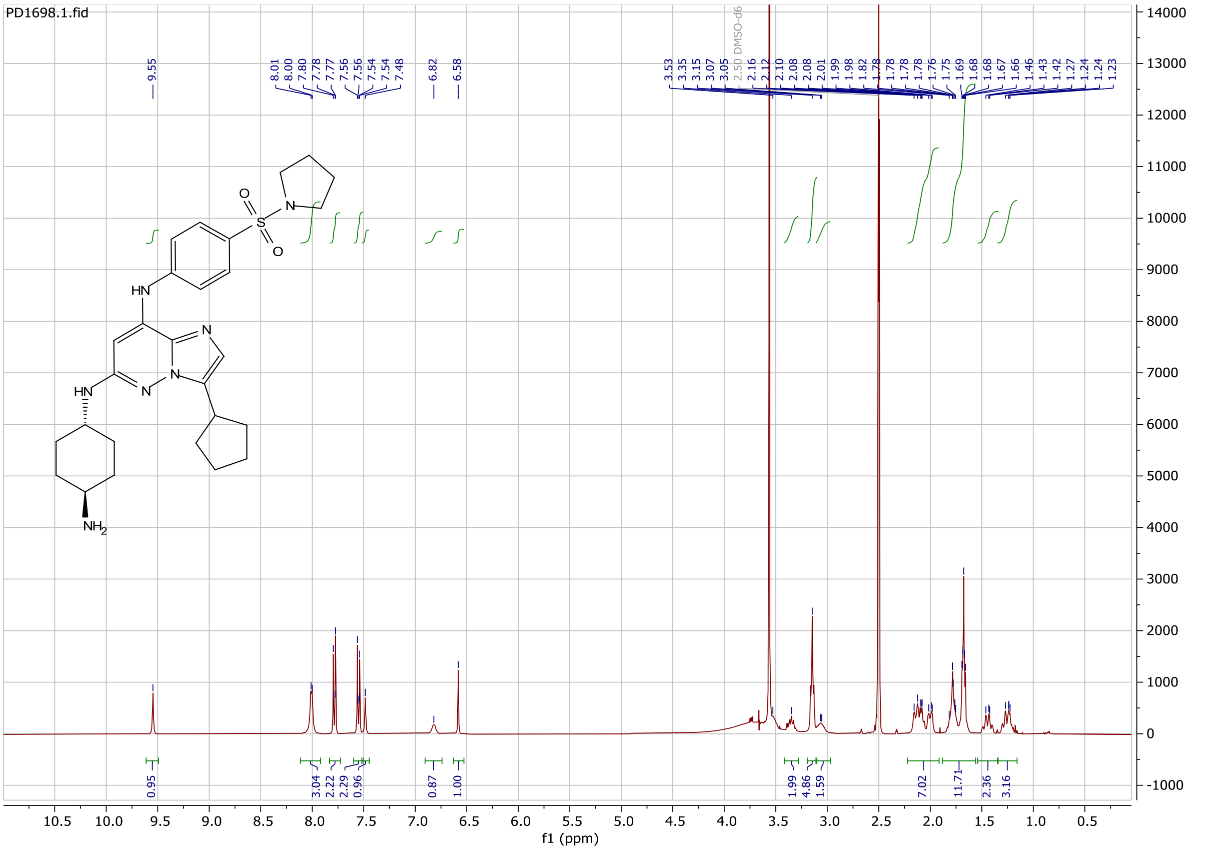

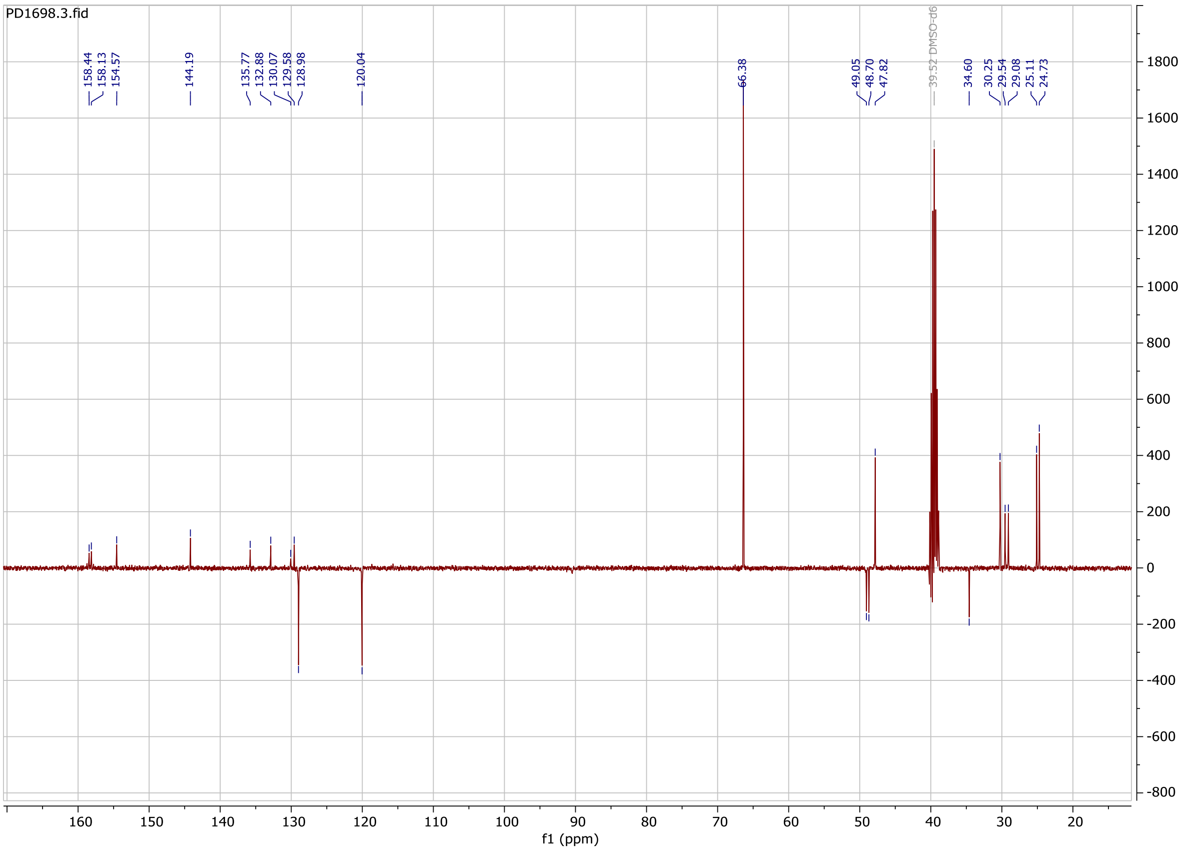
PD1698.3.fid
158.44
158.13
154.57
144.19
135.77
132.88
130.07
129.58
128.98
120.04
66.38
49.05
48.70
47.82
39.52 DMSO-d6
34.60
30.25
29.54
29.08
25.11
24.73
f1 (ppm)

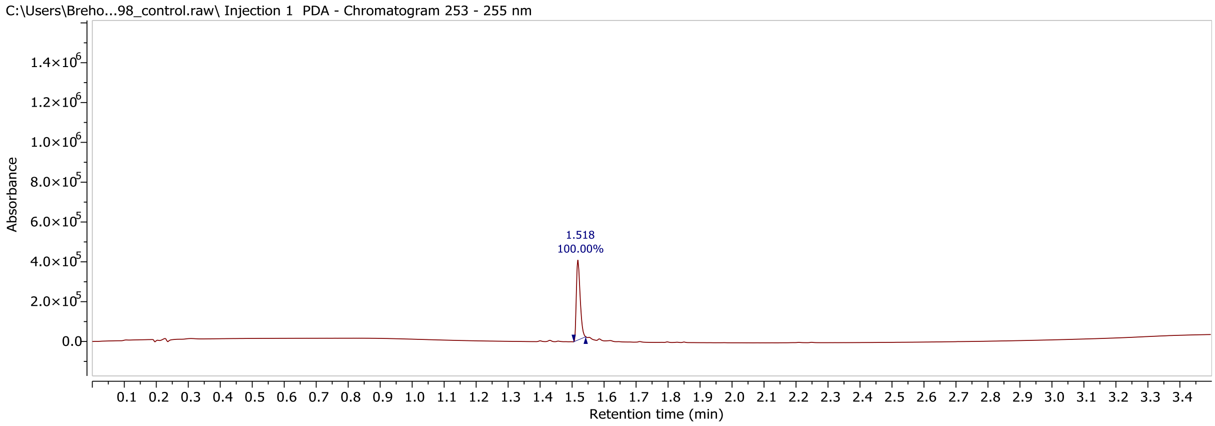
C:\Users\Breho...98_control.raw\ Injection 1 PDA - Chromatogram 253 - 255 nm
Absorbance
1.518
100.00%
Retention time (min)

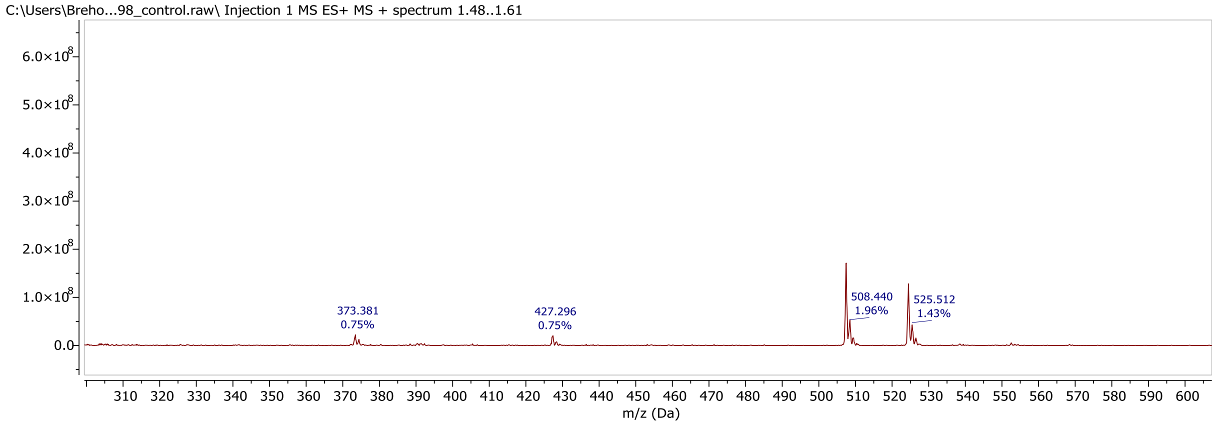
C:\Users\Breho...98_control.raw\ Injection 1 MS ES+ MS + spectrum 1.48..1.61
373.381
0.75%
427.296
0.75%
508.440
1.96%
525.512
1.43%
m/z (Da)

N^6-((1r,4r)-4-aminocyclohexyl)-3-(2-methylpropan-1-yl)-N^8-(4-(1-pyrrolidinylsulfonyl)phenyl)imidazo[1,2-*b*]pyridazine-6,8-diamine (34c).

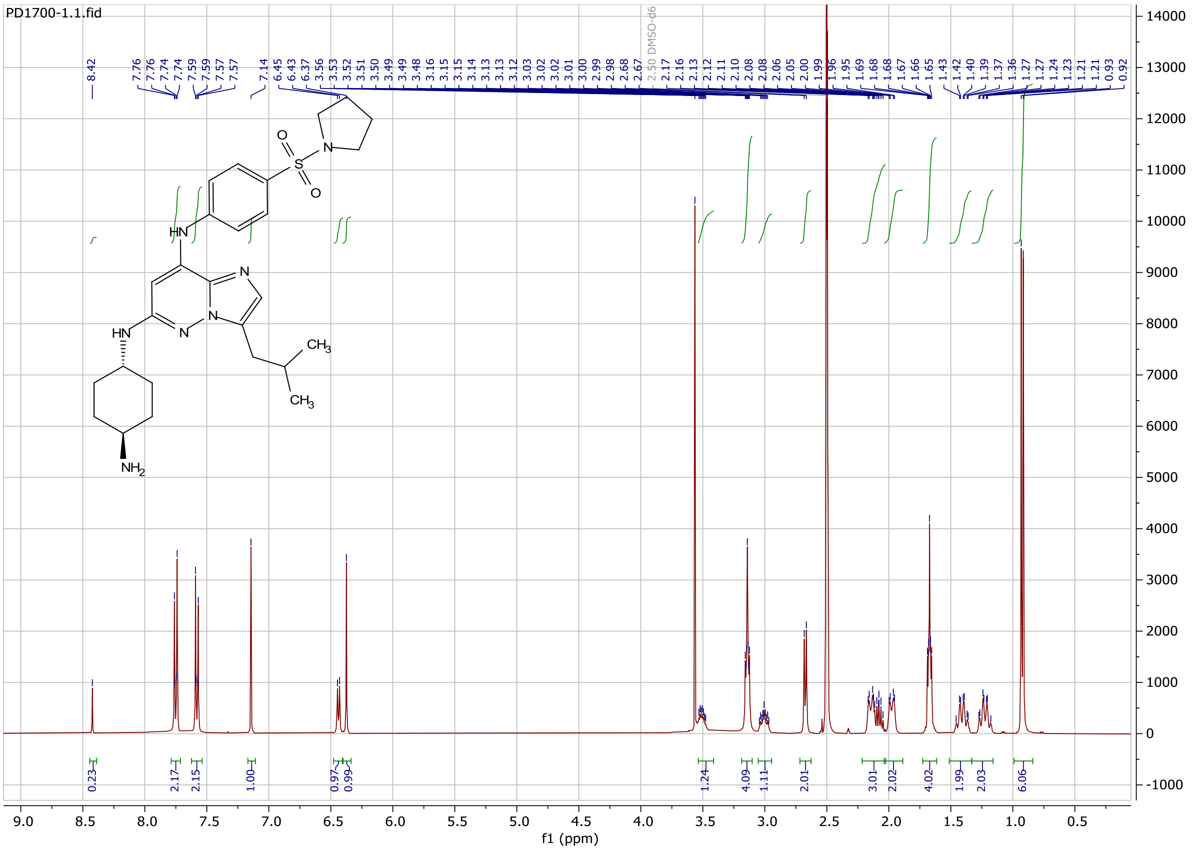

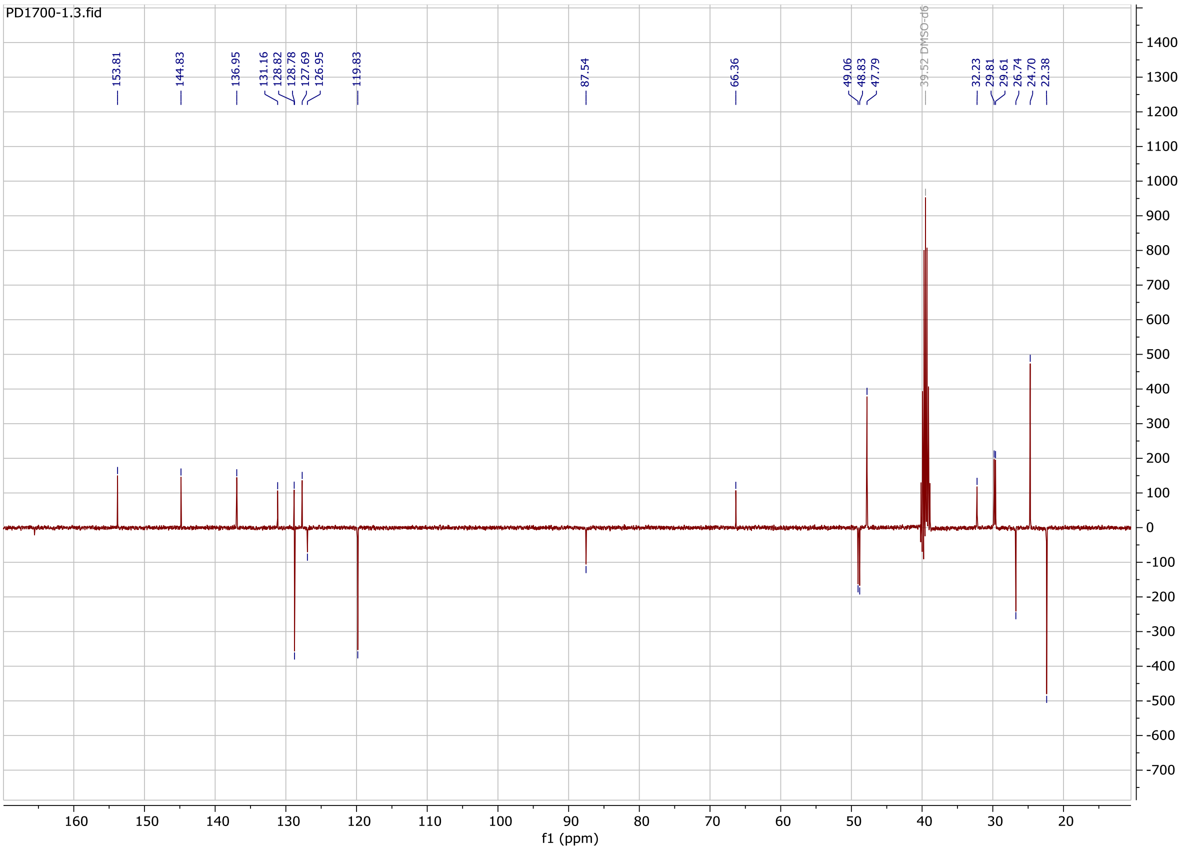

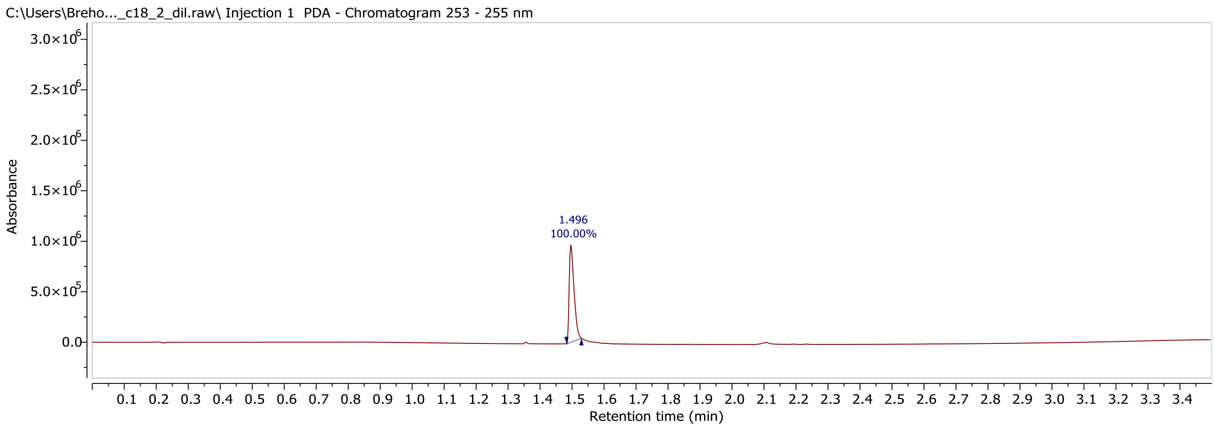

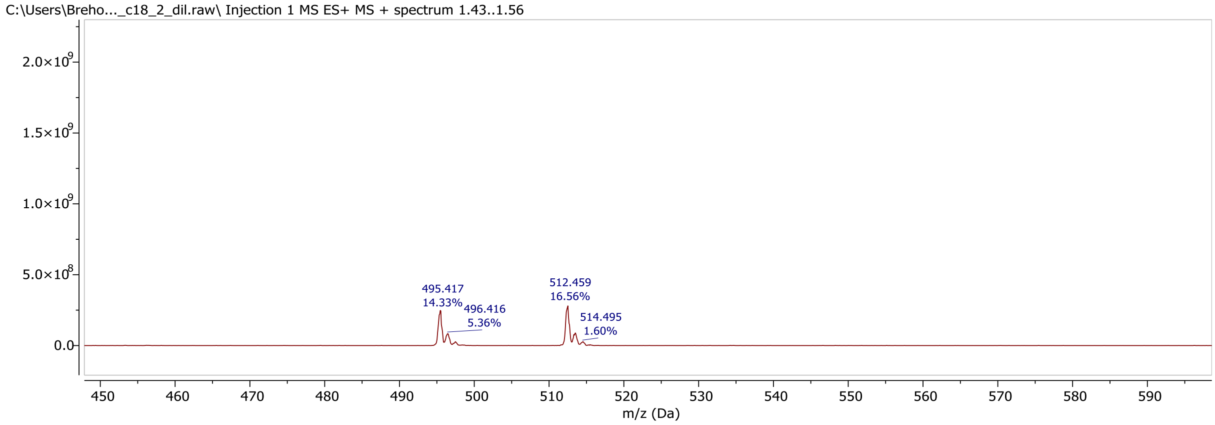

N[6]-((1r,4r)-4-aminocyclohexyl)-3-(propan-2-yl)-*N*[8]-(4-(1-pyrrolidinylsulfonyl)phenyl)imidazo[1,2-*b*]pyridazine-6,8-diamine (34d)

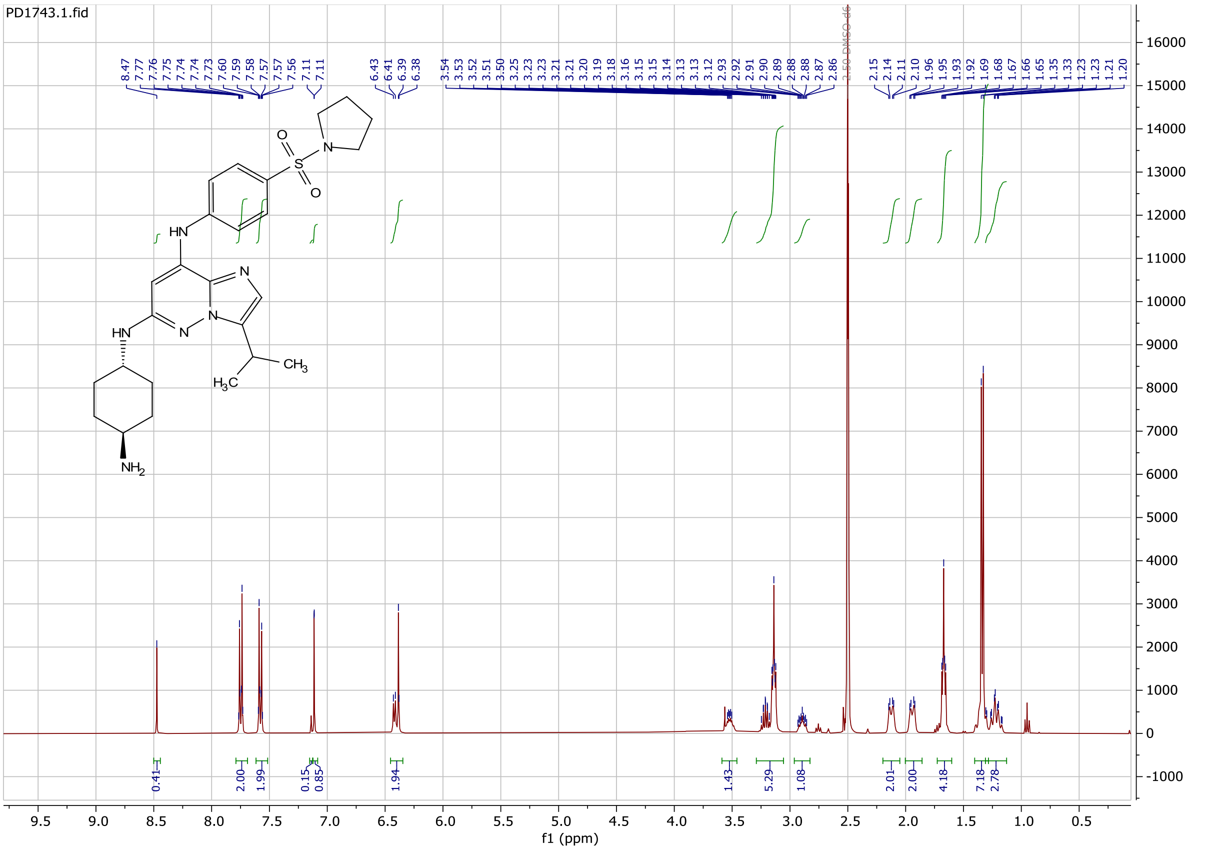

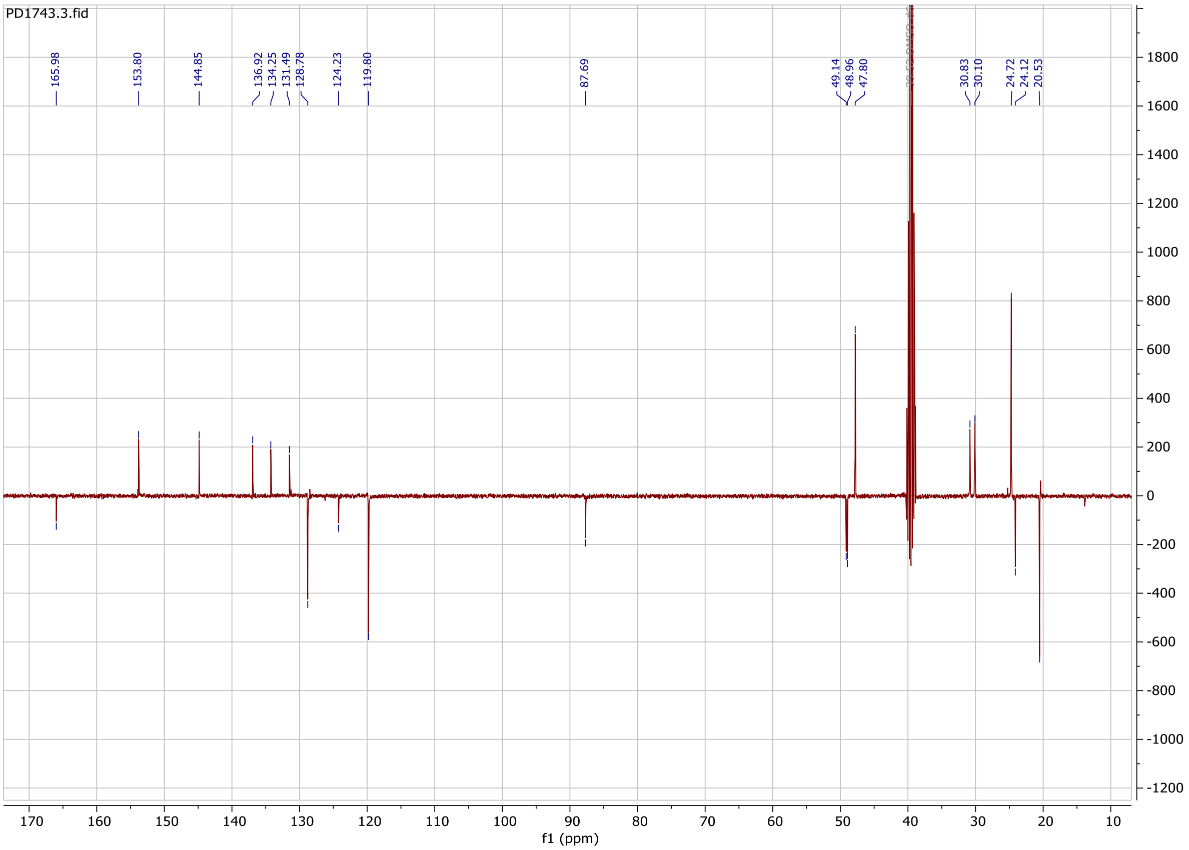

C:\Users\Breho...\PD1743_c2.raw\ Injection 1 PDA - Chromatogram 253 - 255 nm

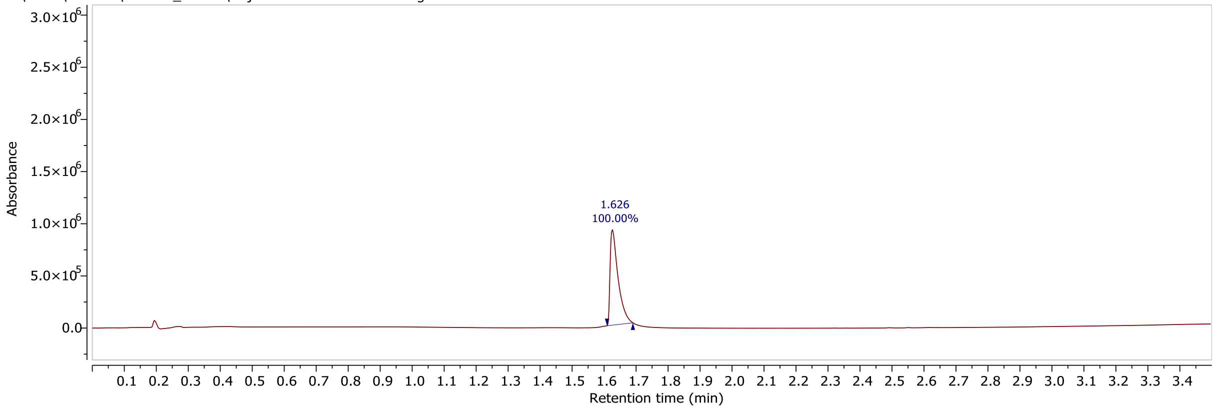

C:\Users\Breho...\PD1743_c2.raw\ Injection 1 MS ES+ MS + spectrum 1.57..1.72

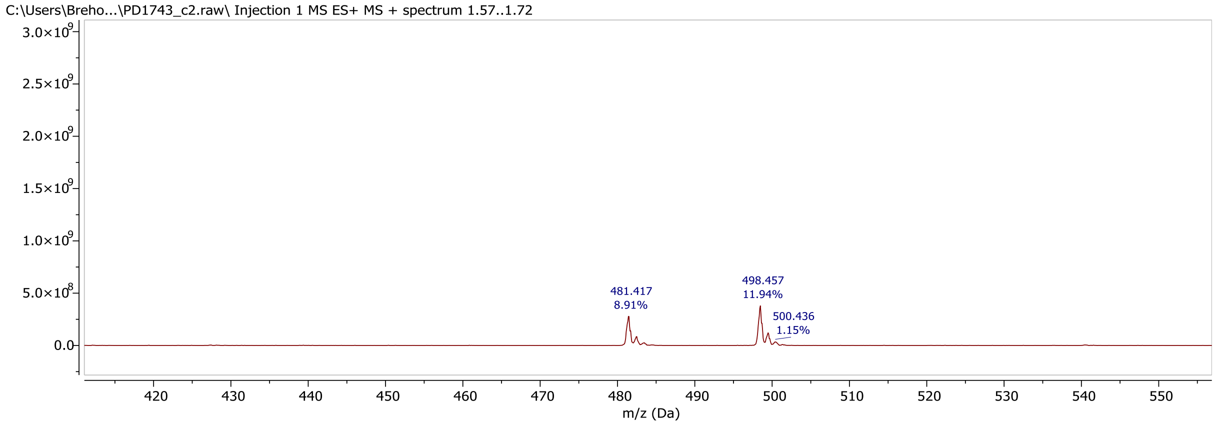

N^6-((1r,4r)-4-aminocyclohexyl)-3-methyl-N^8-(4-(1-pyrrolidinylsulfonyl)phenyl)imidazo[1,2-*b*]pyridazine-6,8-diamine (34e)

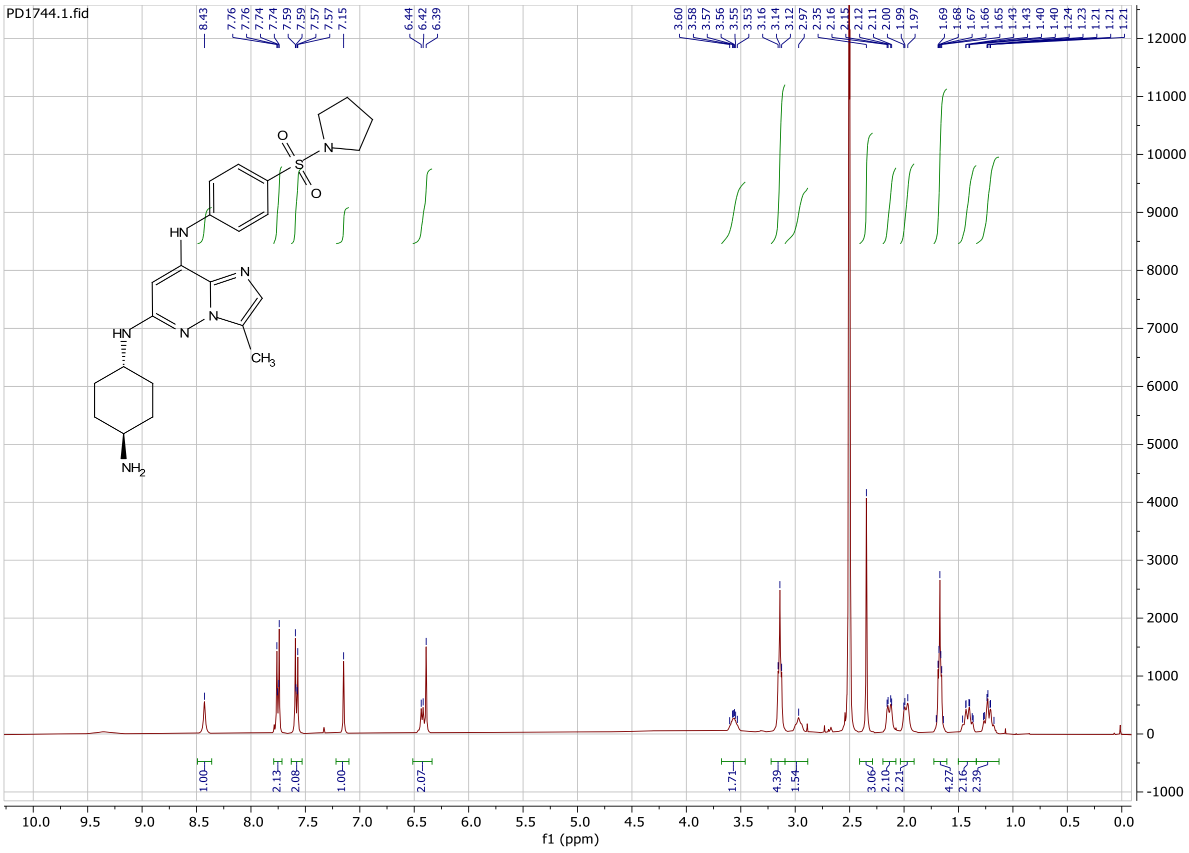

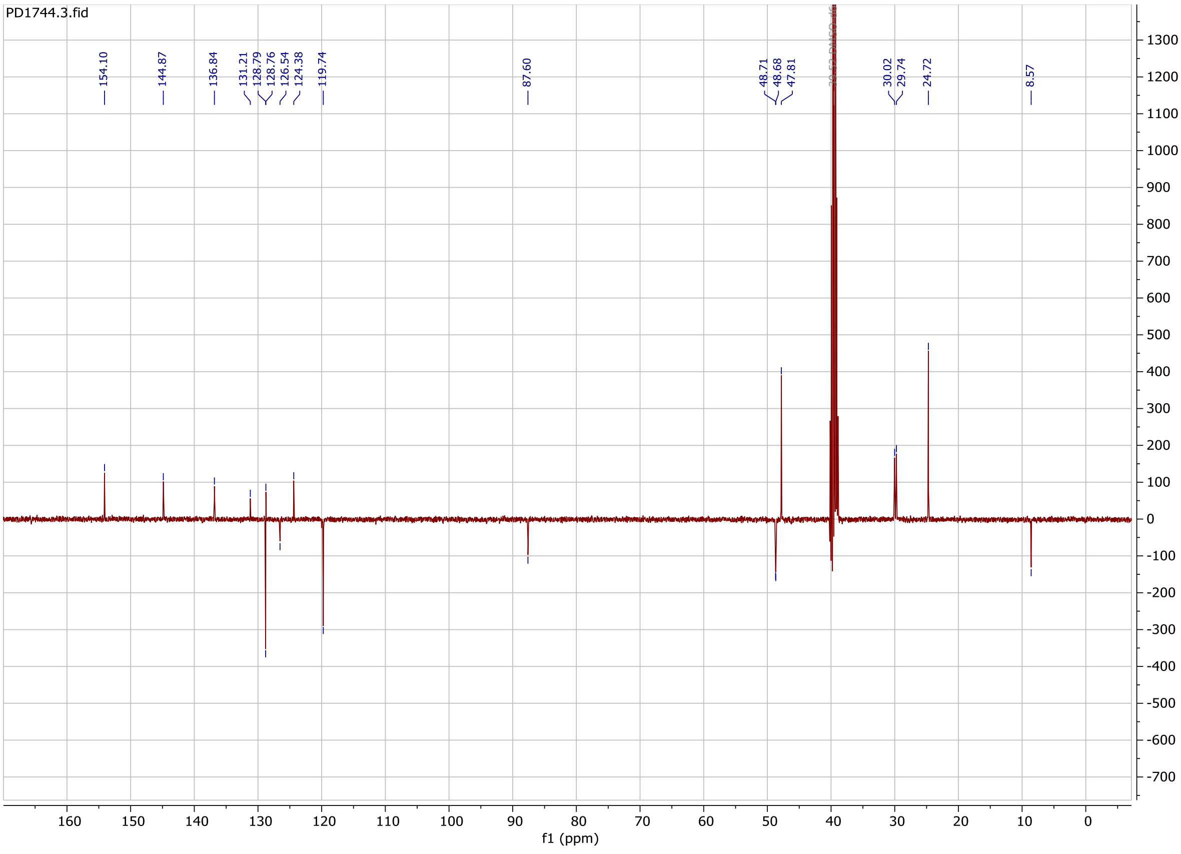

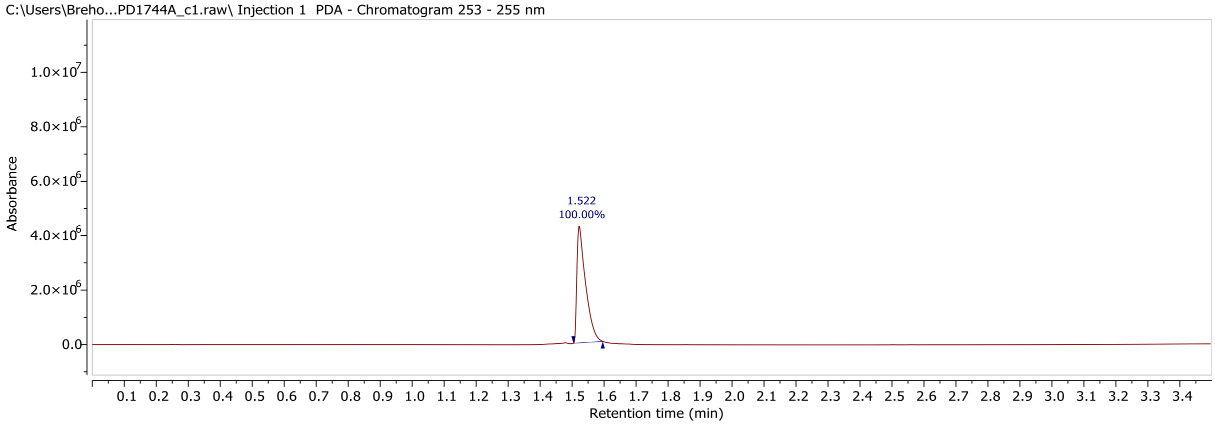

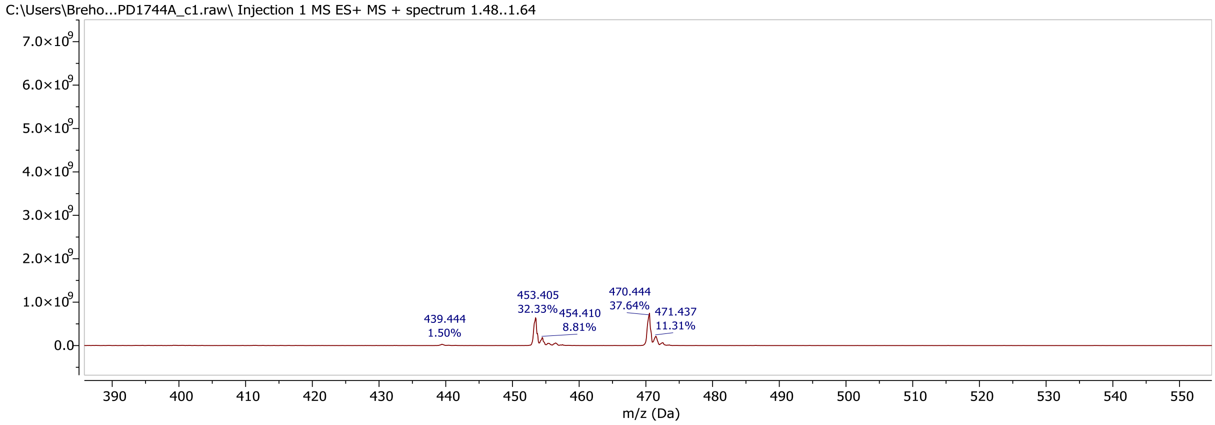

N^6-((1r,4r)-4-aminocyclohexyl)-3-phenyl-N^8-(4-(1-pyrrolidinylsulfonyl)phenyl)imidazo[1,2-*b*]pyridazine-6,8-diamine (34f)

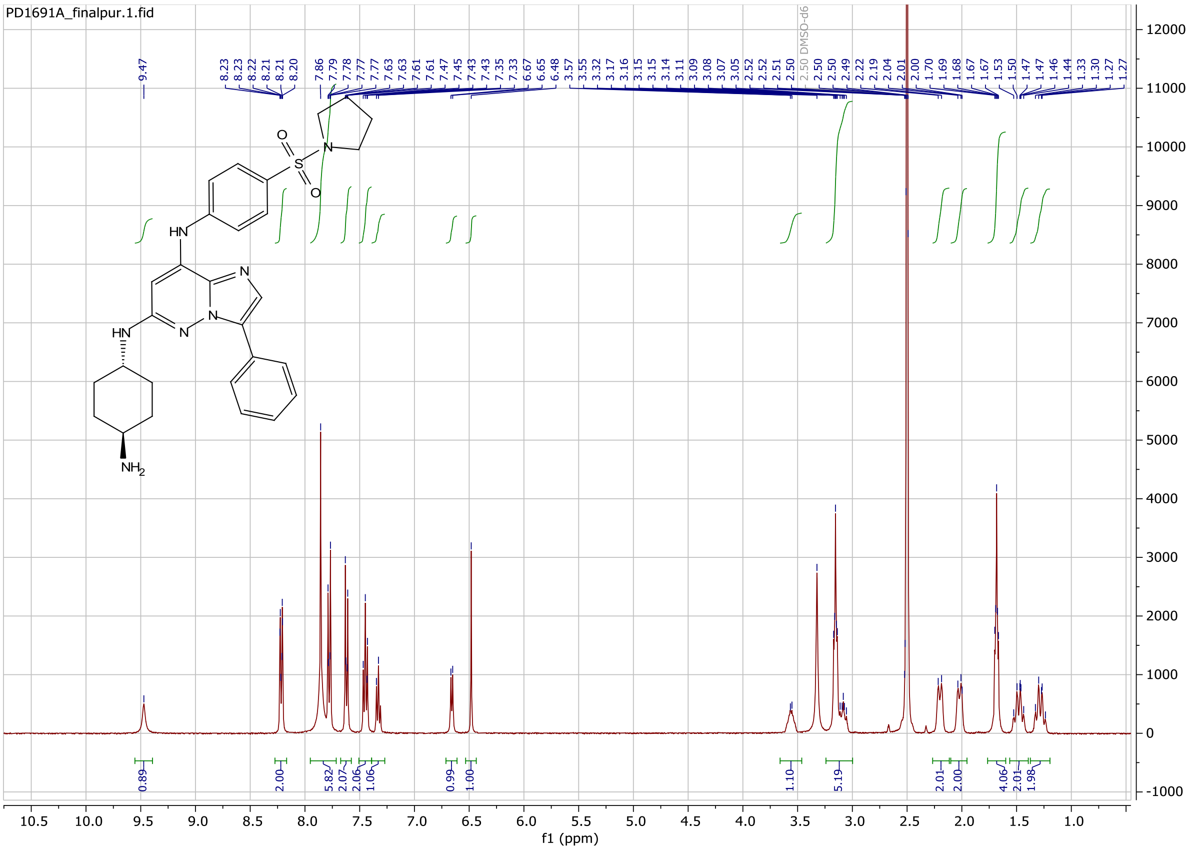

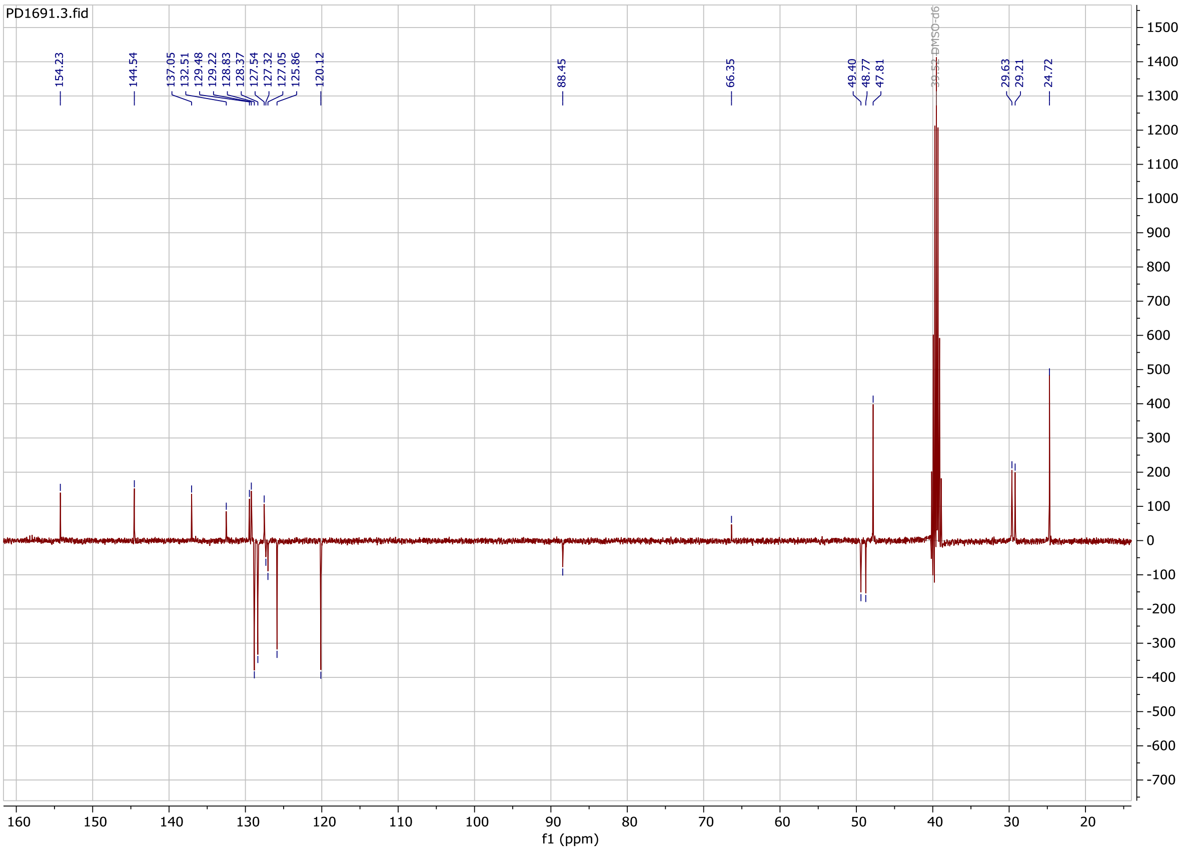
PD1691.3.fid
154.23
144.54
137.05
132.51
129.48
129.22
128.83
128.37
127.54
127.32
127.05
125.86
120.12
88.45
66.35
49.40
48.77
47.81
39.52 DMSO-d6
29.63
29.21
24.72
f1 (ppm)

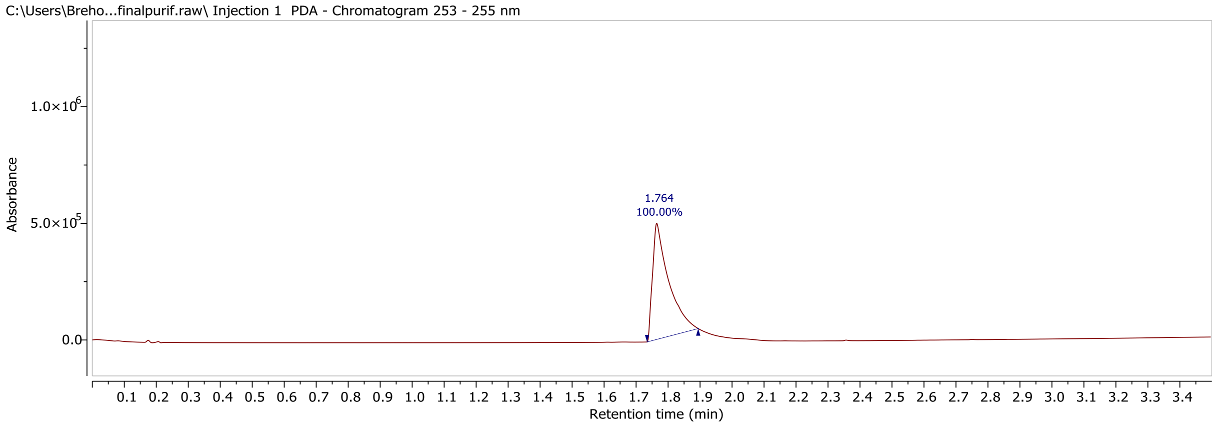
C:\Users\Breho...finalpurif.raw\ Injection 1 PDA - Chromatogram 253 - 255 nm
Absorbance
1.764
100.00%
Retention time (min)

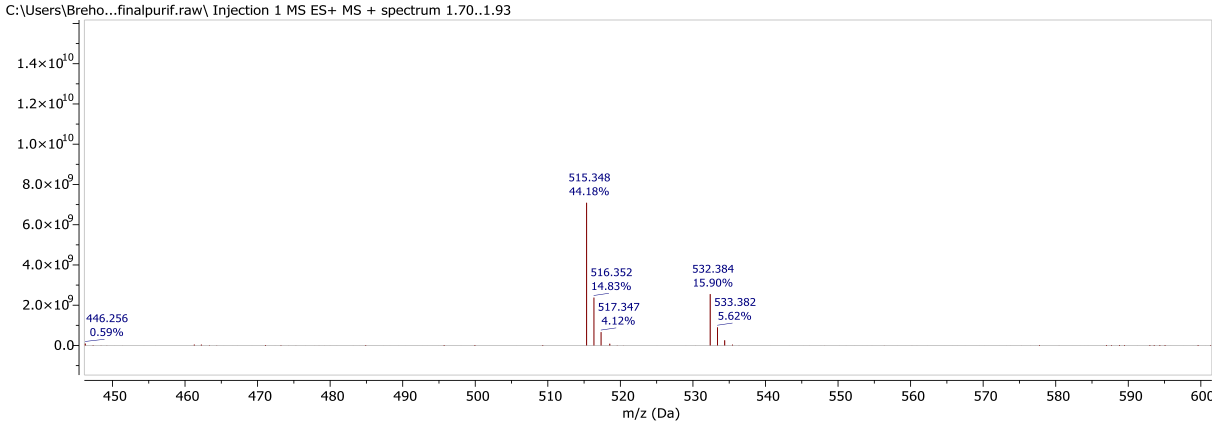
C:\Users\Breho...finalpurif.raw\ Injection 1 MS ES+ MS + spectrum 1.70..1.93
515.348
44.18%
516.352
14.83%
517.347
4.12%
532.384
15.90%
533.382
5.62%
446.256
0.59%
m/z (Da)

N[6]-((1r,4r)-4-aminocyclohexyl)-3-(4-methoxyphenyl)-*N*[8]-(4-(1-pyrrolidinylsulfonyl)phenyl)imidazo[1,2-*b*]pyridazine-6,8-diamine (34g)

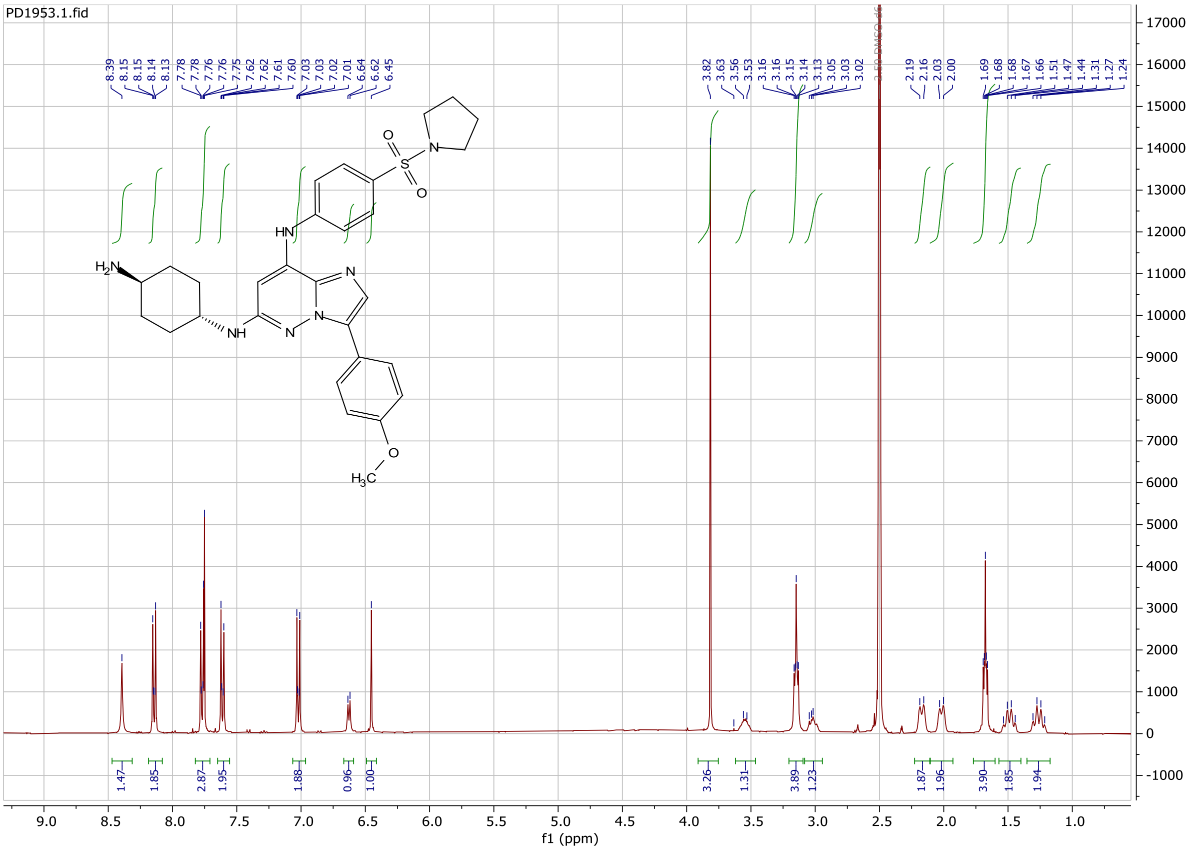

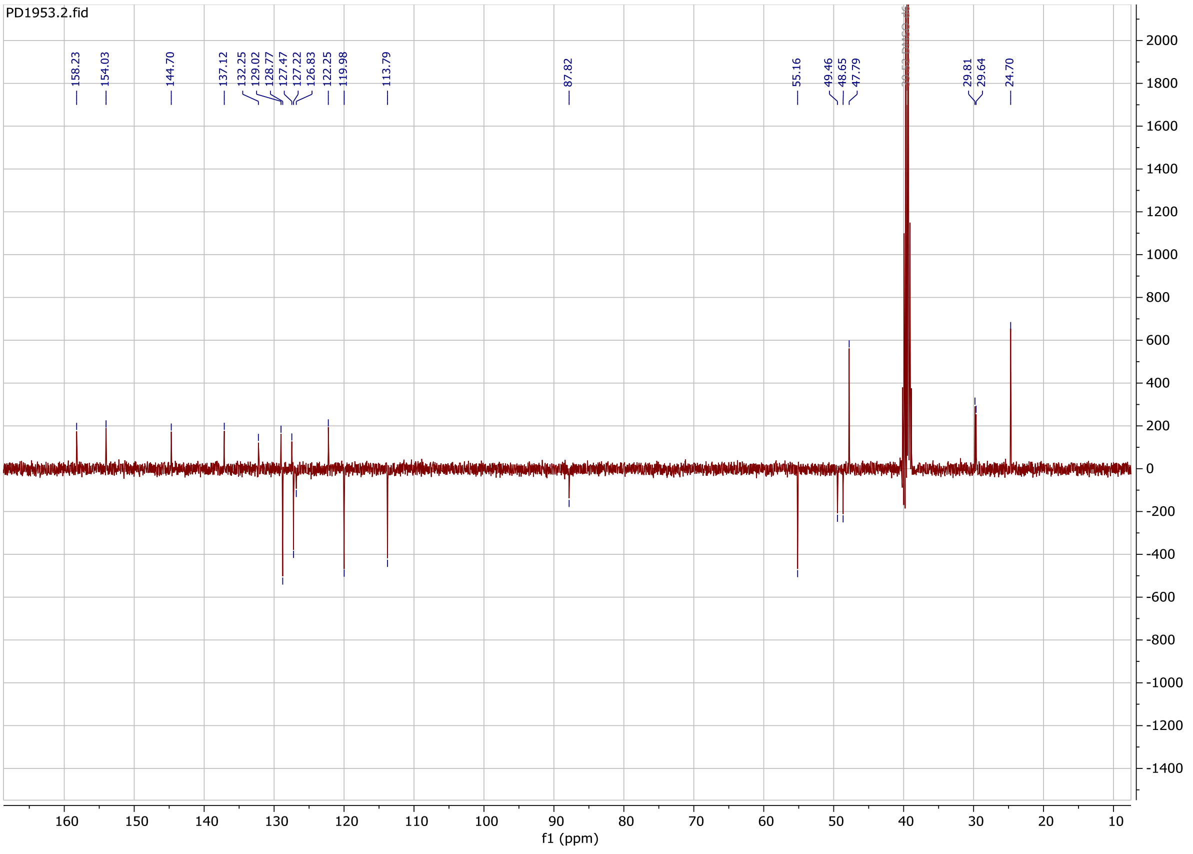

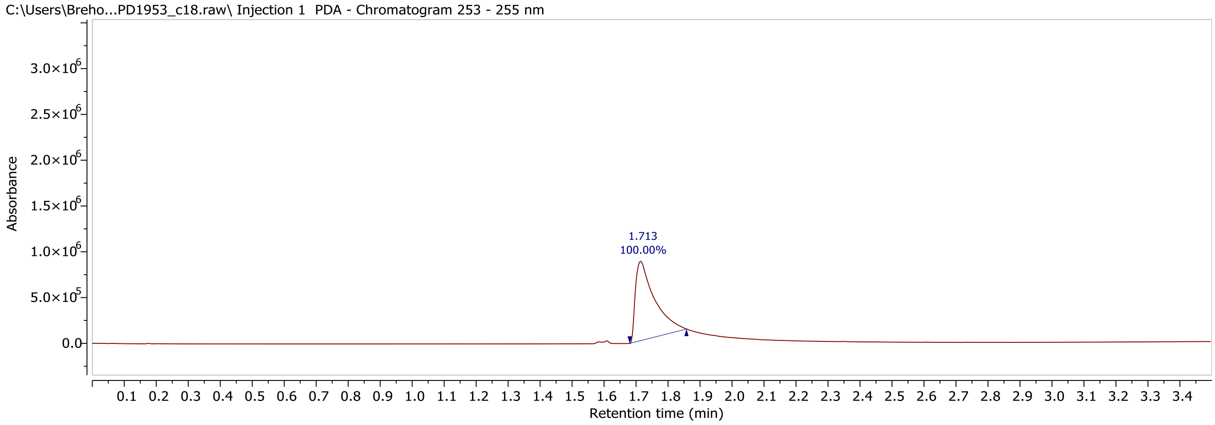

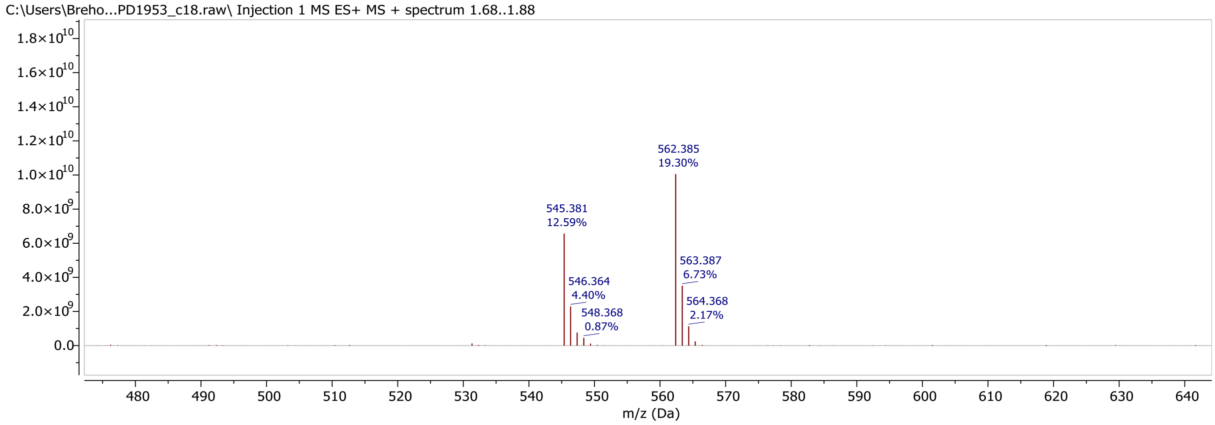

N^6-((1r,4r)-4-aminocyclohexyl)-3-(3-methoxyphenyl)-N^8-(4-(1-pyrrolidinylsulfonyl)phenyl)imidazo[1,2-*b*]pyridazine-6,8-diamine (34h)

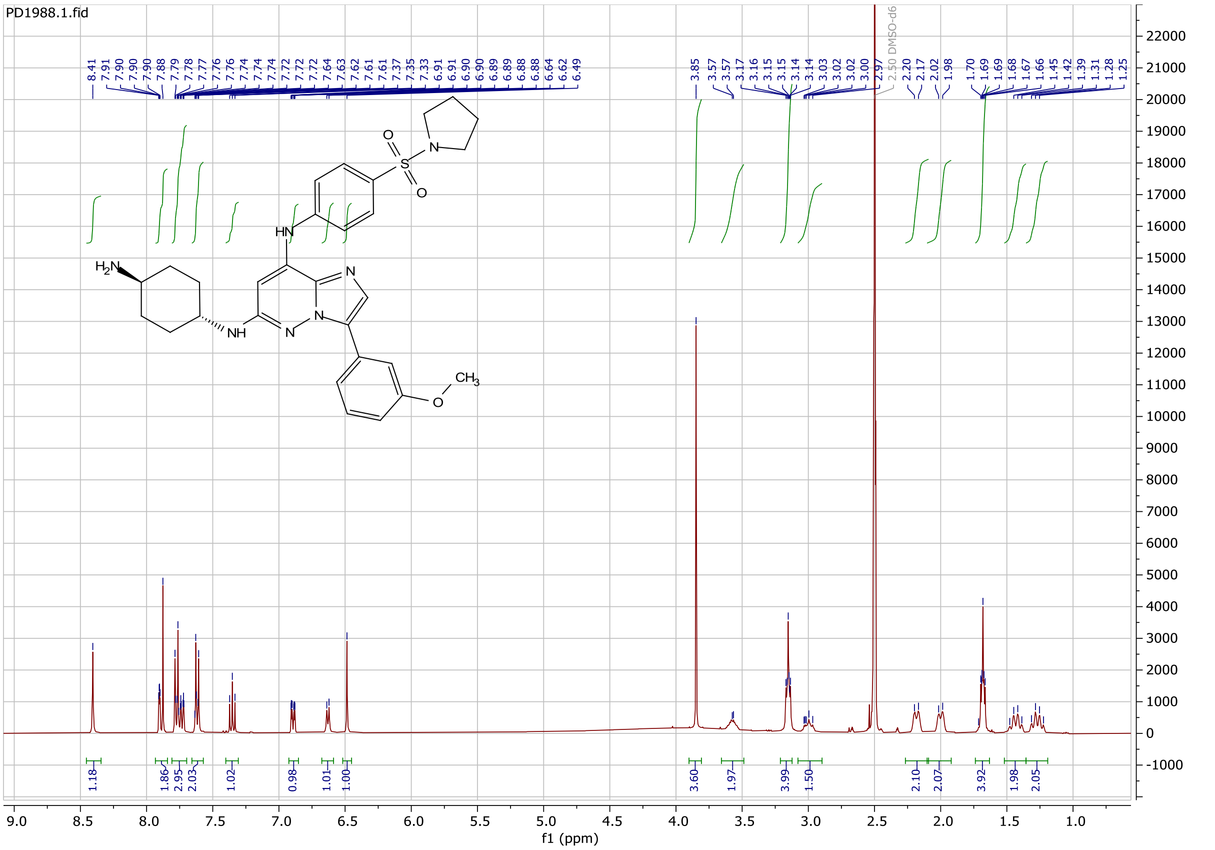

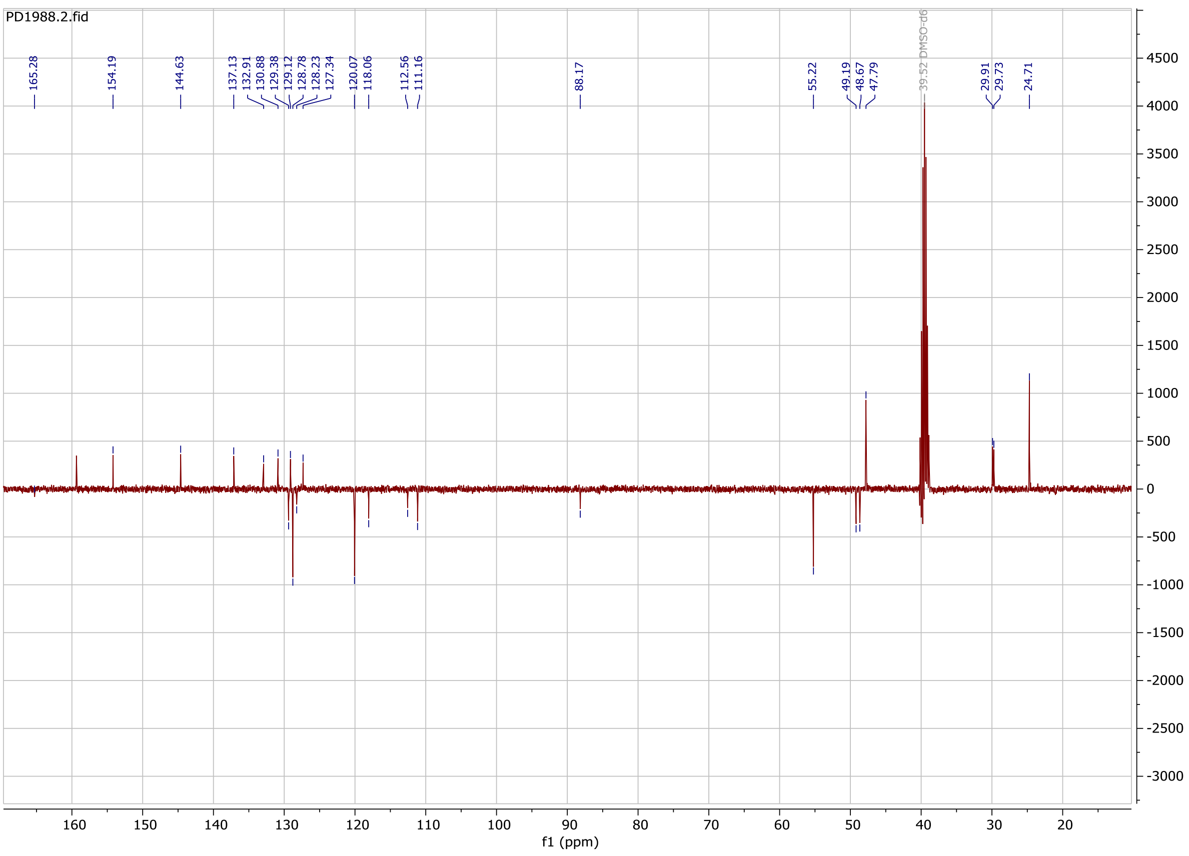
PD1988.2.fid
165.28
154.19
144.63
137.13
132.91
130.88
129.38
129.12
128.78
128.23
127.34
120.07
118.06
112.56
111.16
88.17
55.22
49.19
48.67
47.79
39.52 DMSO-d6
29.91
29.73
24.71
f1 (ppm)

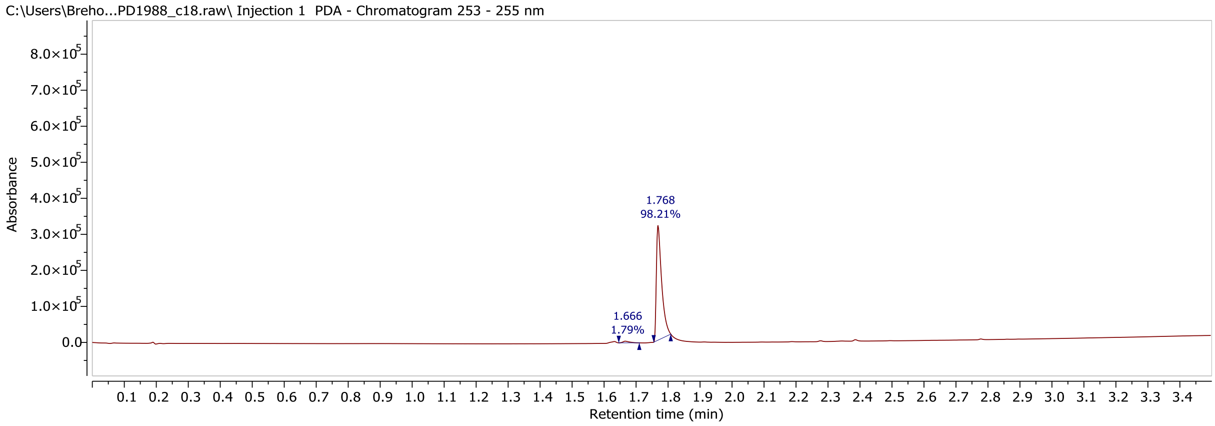
C:\Users\Breho...PD1988_c18.raw\ Injection 1 PDA - Chromatogram 253 - 255 nm
Absorbance
1.768
98.21%
1.666
1.79%
Retention time (min)

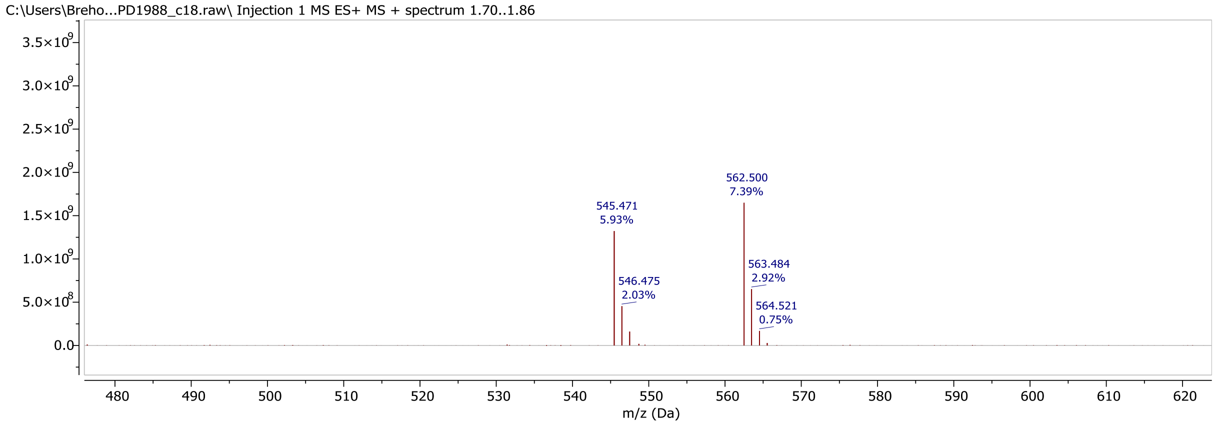
C:\Users\Breho...PD1988_c18.raw\ Injection 1 MS ES+ MS + spectrum 1.70..1.86
545.471
5.93%
546.475
2.03%
562.500
7.39%
563.484
2.92%
564.521
0.75%
m/z (Da)

N^6-((1r,4r)-4-aminocyclohexyl)-3-(3,4-dimethoxyphenyl)-N^8-(4-(1-pyrrolidinylsulfonyl)phenyl)imidazo[1,2-*b*]pyridazine-6,8-diamine (34i)

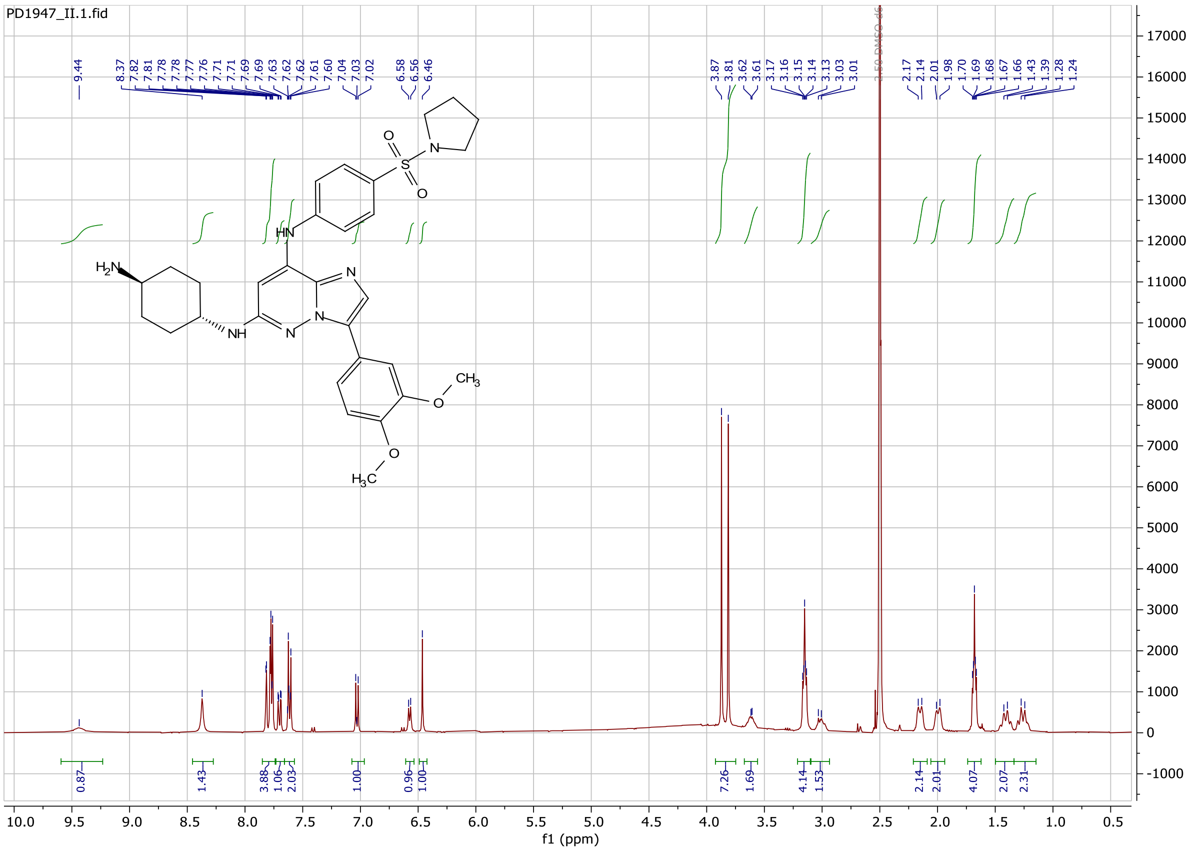

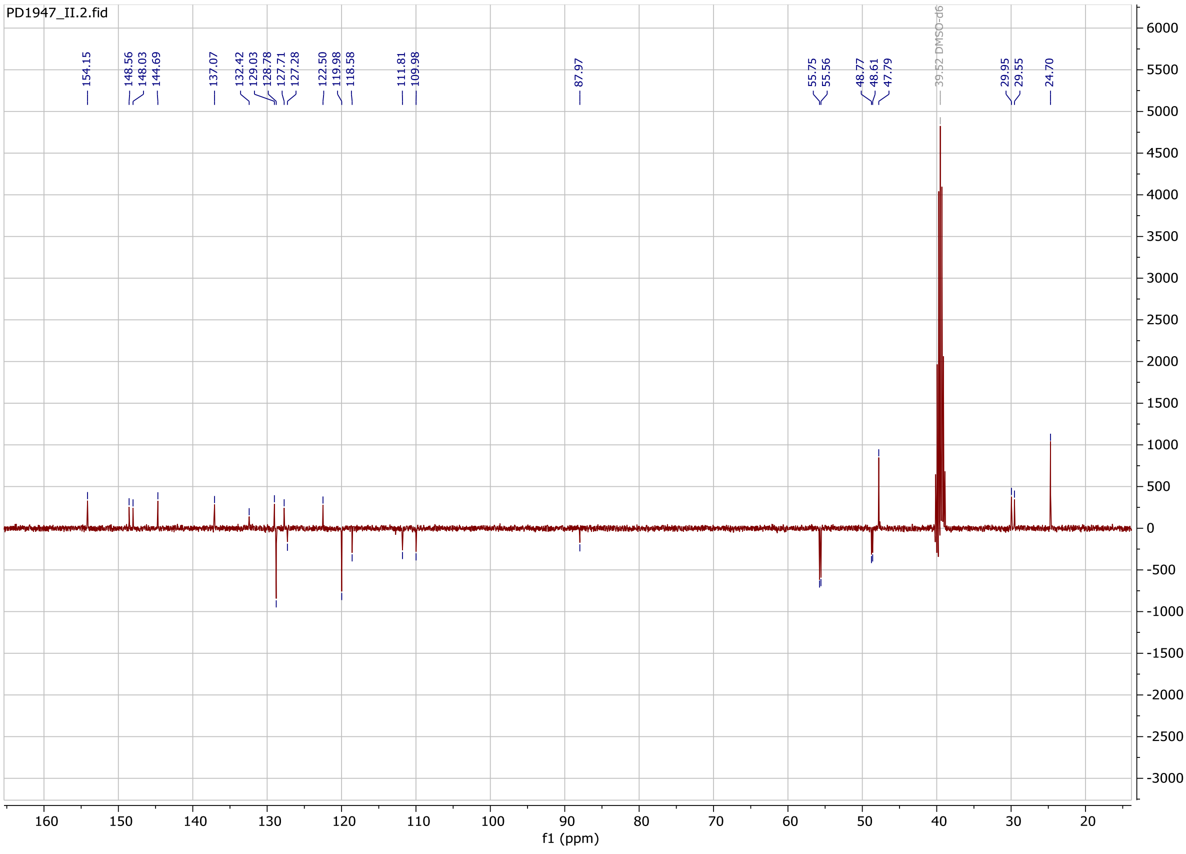

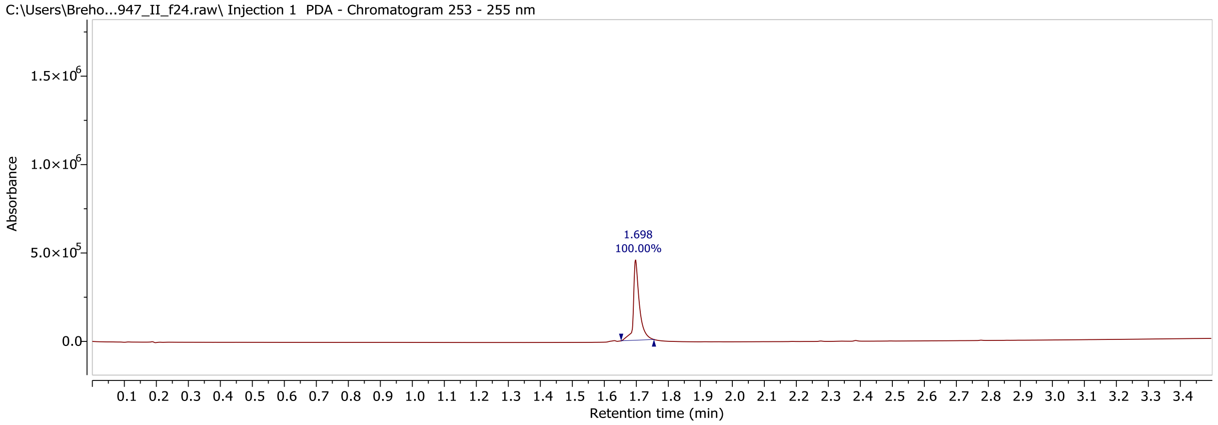

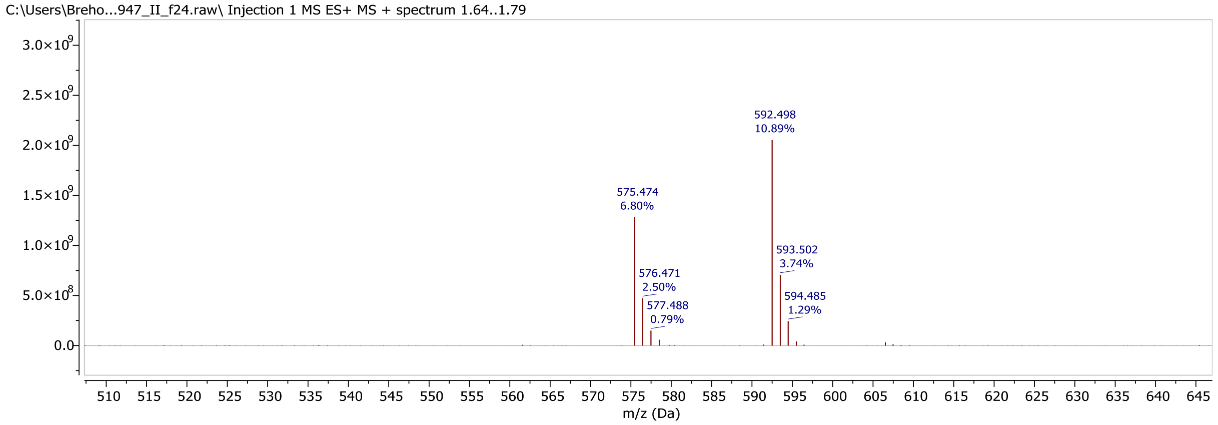

N^{6}-((1r,4r)-4-aminocyclohexyl)-3-(4-fluorophenyl)-*N*^{8}-(4-(1-pyrrolidinylsulfonyl)phenyl)imidazo[1,2-*b*]pyridazine-6,8-diamine (34j)

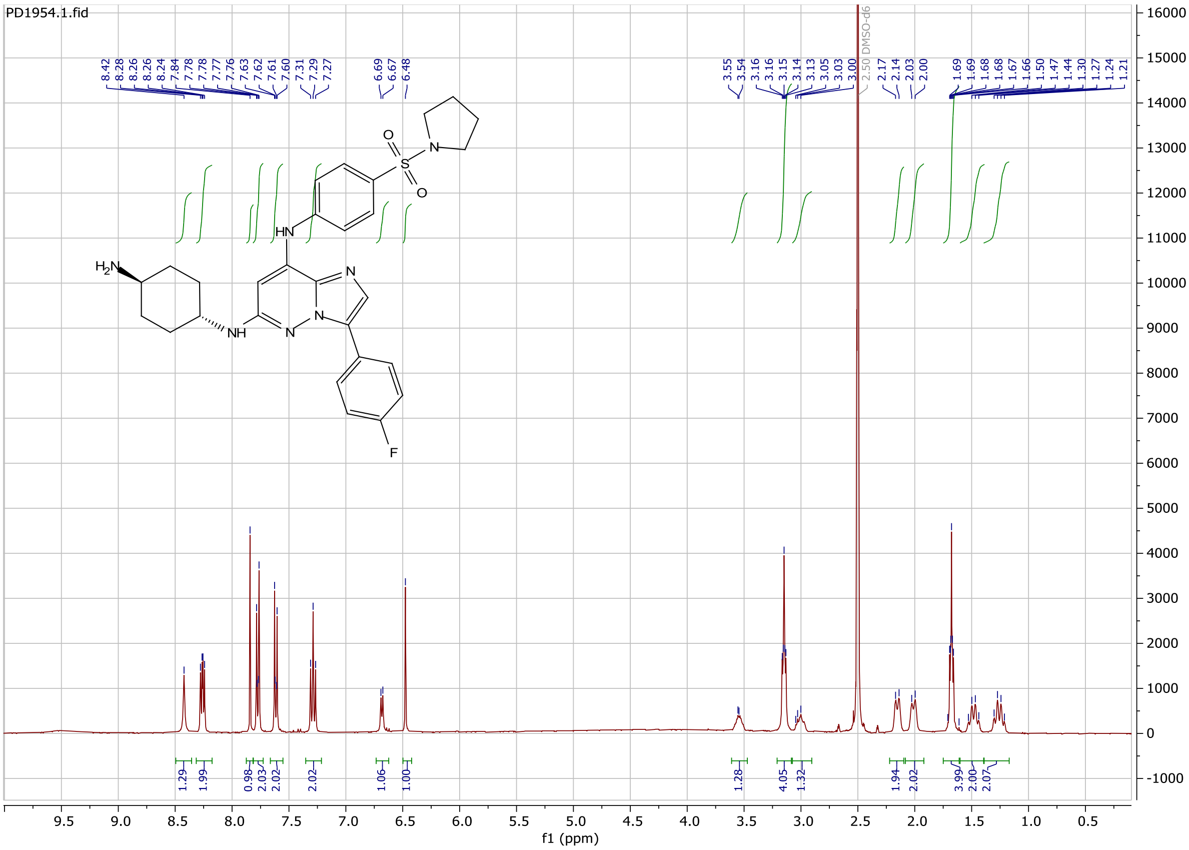

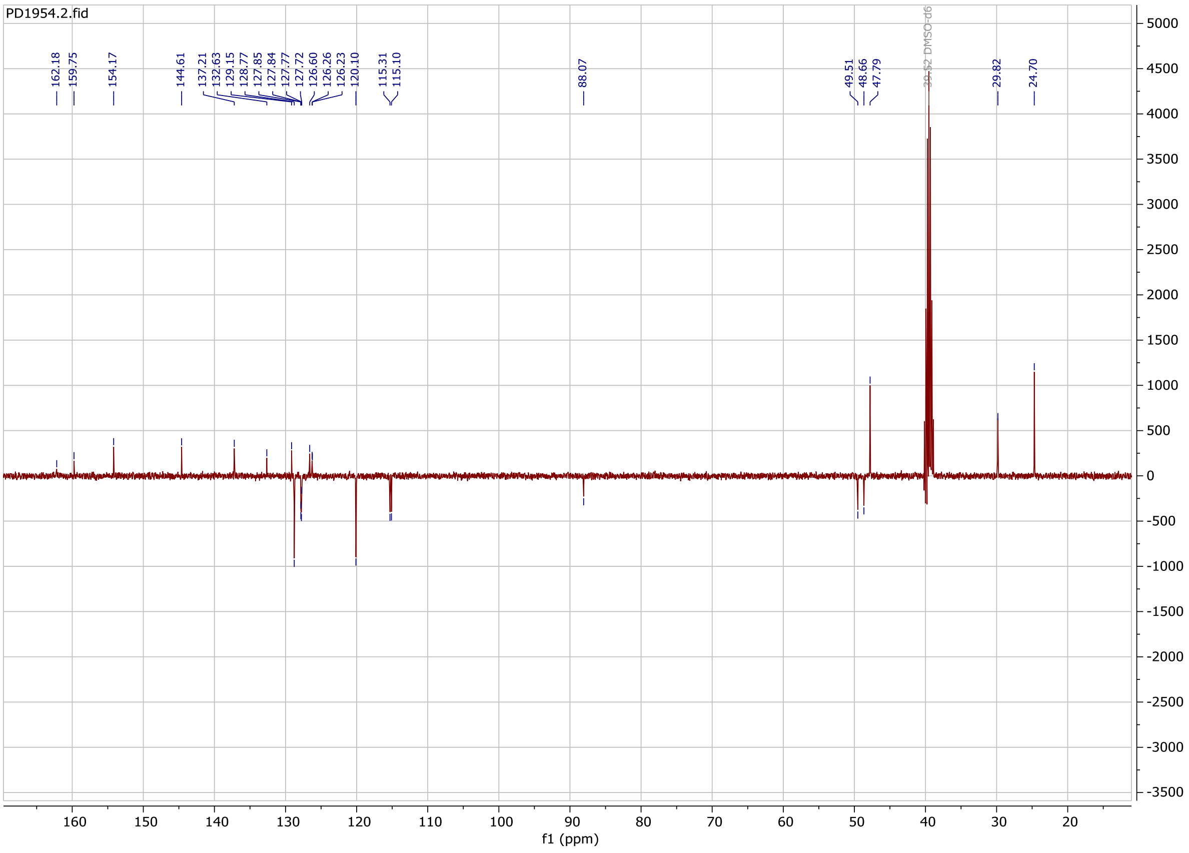
PD1954.2.fid
162.18
159.75
154.17
144.61
137.21
132.63
129.15
128.77
127.85
127.84
127.77
127.72
126.60
126.26
126.23
120.10
115.31
115.10
88.07
49.51
48.66
47.79
39.52 DMSO-d6
29.82
24.70
f1 (ppm)

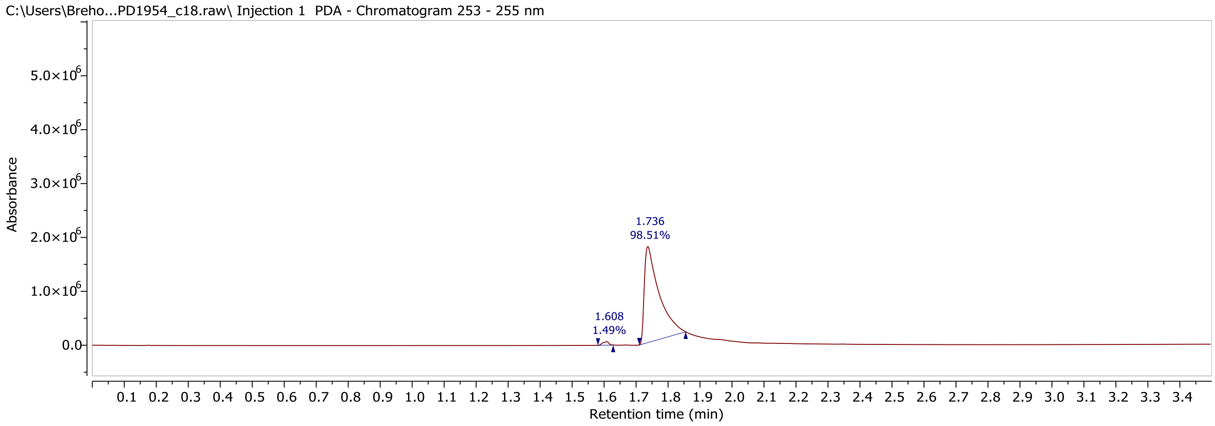
C:\Users\Breho...PD1954_c18.raw\ Injection 1 PDA - Chromatogram 253 - 255 nm
Absorbance
1.736
98.51%
1.608
1.49%
Retention time (min)

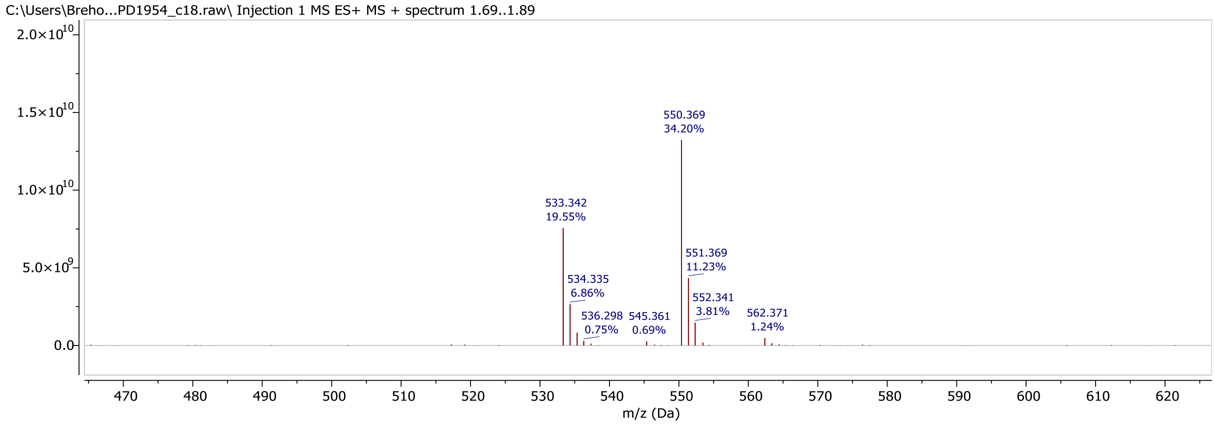
C:\Users\Breho...PD1954_c18.raw\ Injection 1 MS ES+ MS + spectrum 1.69..1.89
550.369
34.20%
533.342
19.55%
551.369
11.23%
534.335
6.86%
552.341
3.81%
536.298
0.75%
545.361
0.69%
562.371
1.24%
m/z (Da)

N[6]-((1r,4r)-4-aminocyclohexyl)-3-(3-fluoro-4-hydroxyphenyl)-*N*[8]-(4-(1-pyrrolidinylsulfonyl)phenyl)imidazo[1,2-*b*]pyridazine-6,8-diamine (34k)

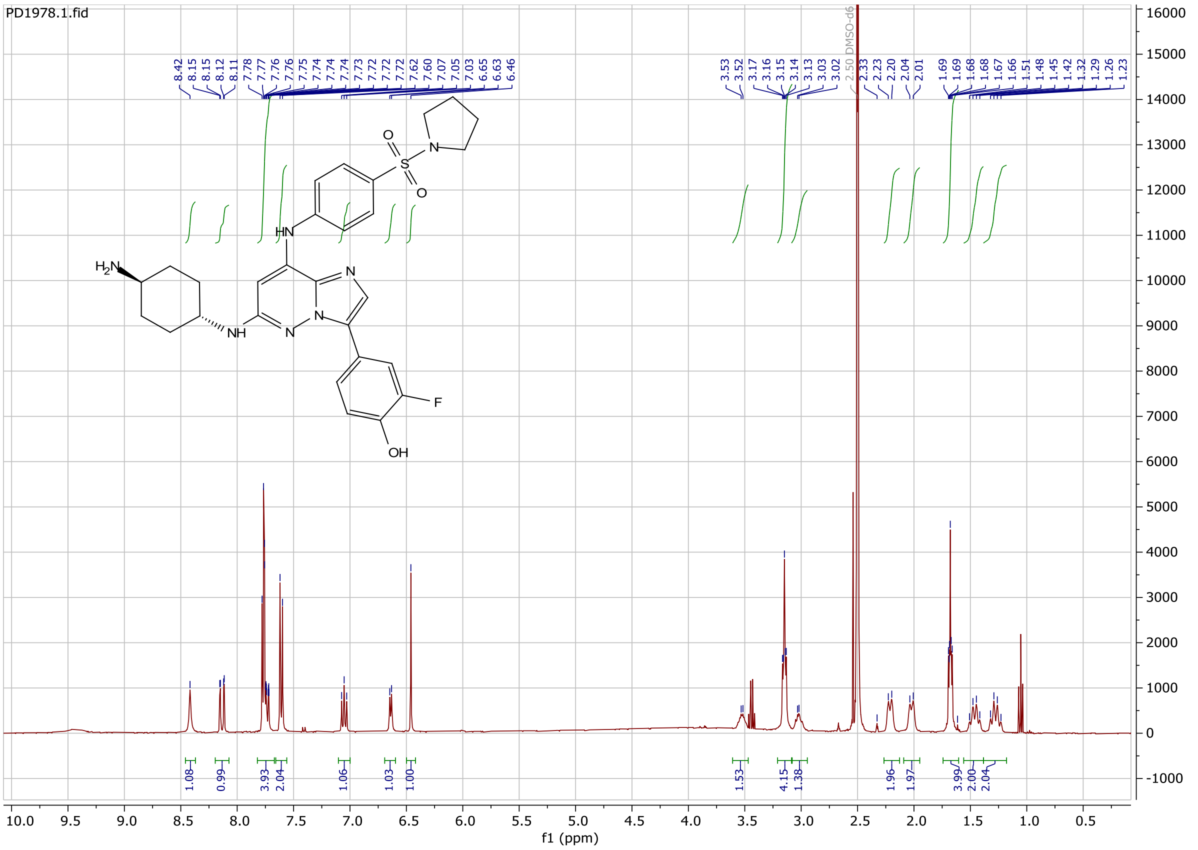

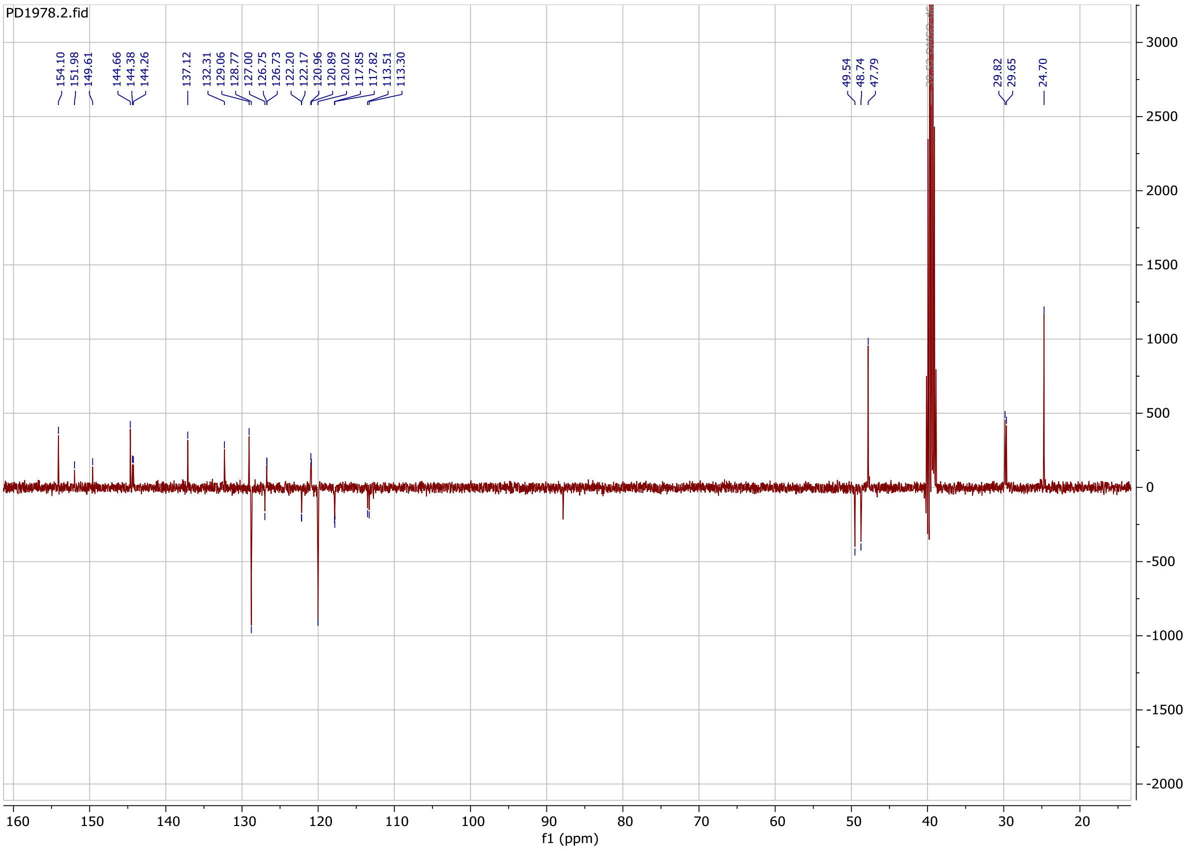

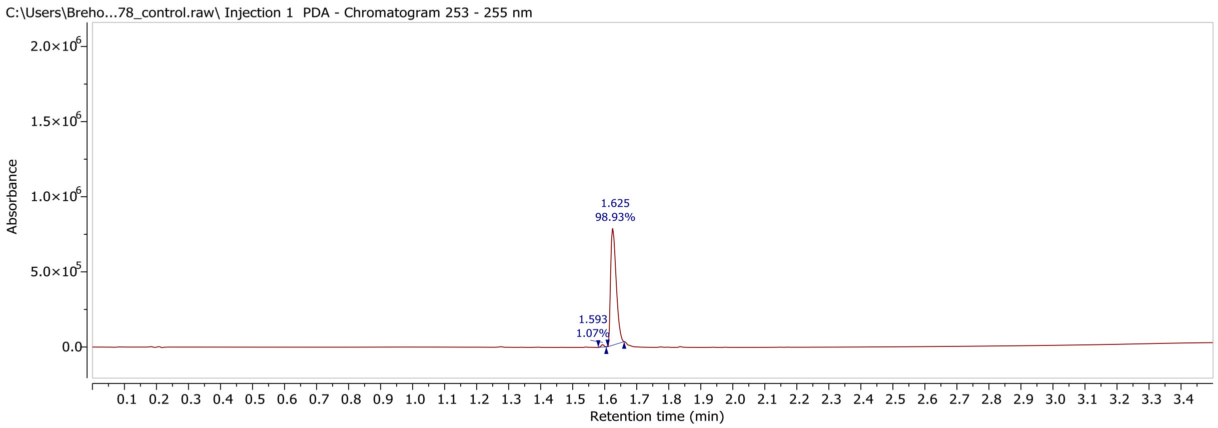

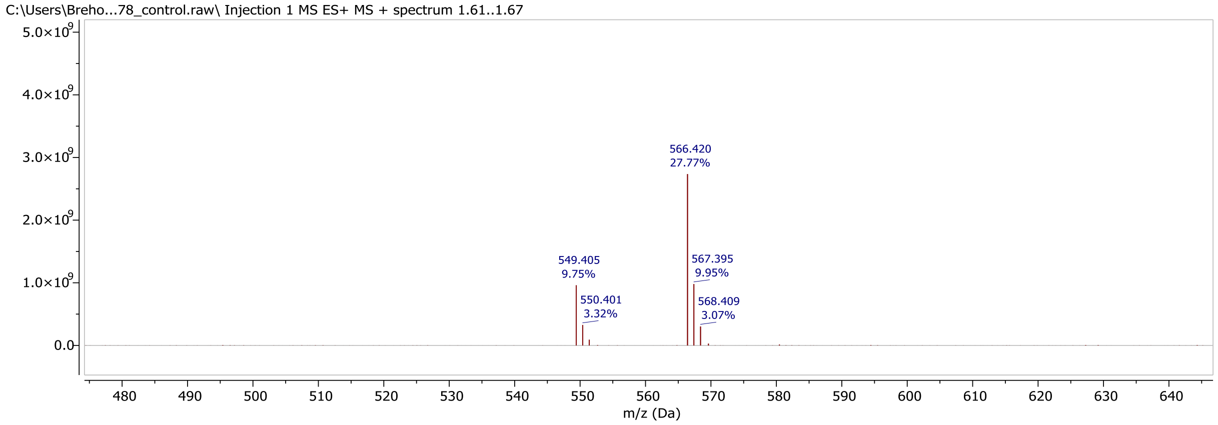

N[6]-((1r,4r)-4-aminocyclohexyl)-3-(4-(trifluoromethyl)phenyl)-*N*[8]-(4-(1-pyrrolidinylsulfonyl)phenyl)imidazo[1,2-*b*]pyridazine-6,8-diamine (34l)

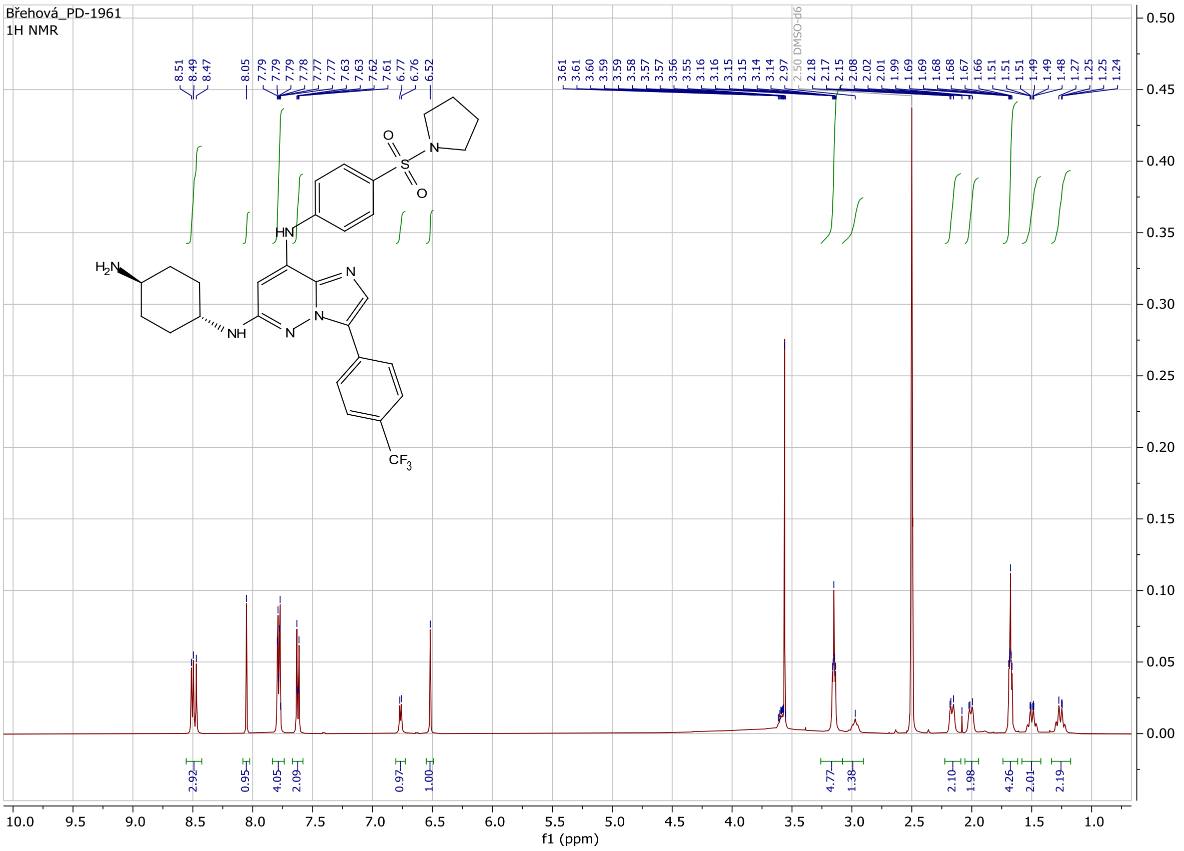

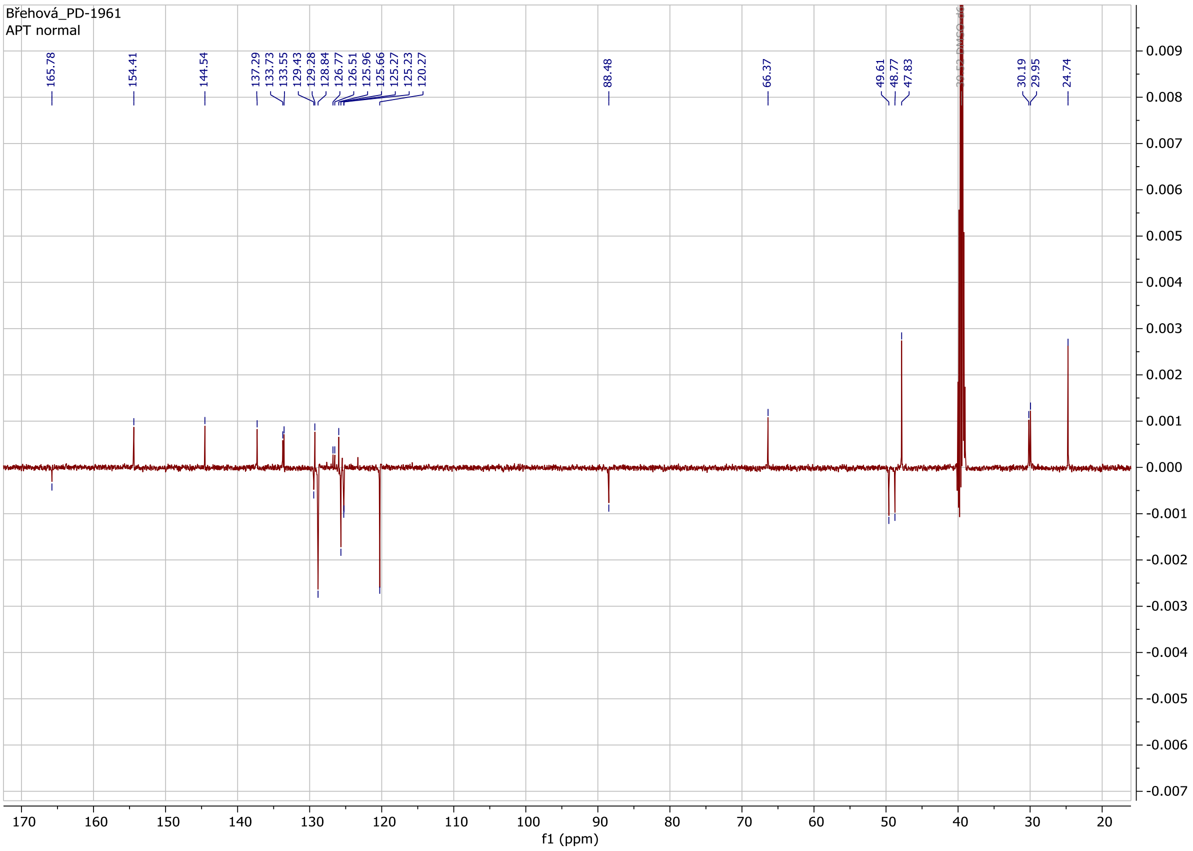

Břehová_PD-1961
APT normal
165.78
154.41
144.54
137.29
133.73
133.55
129.43
129.28
128.84
126.77
126.51
125.96
125.66
125.27
125.23
120.27
88.48
66.37
49.61
48.77
47.83
30.19
29.95
24.74
f1 (ppm)

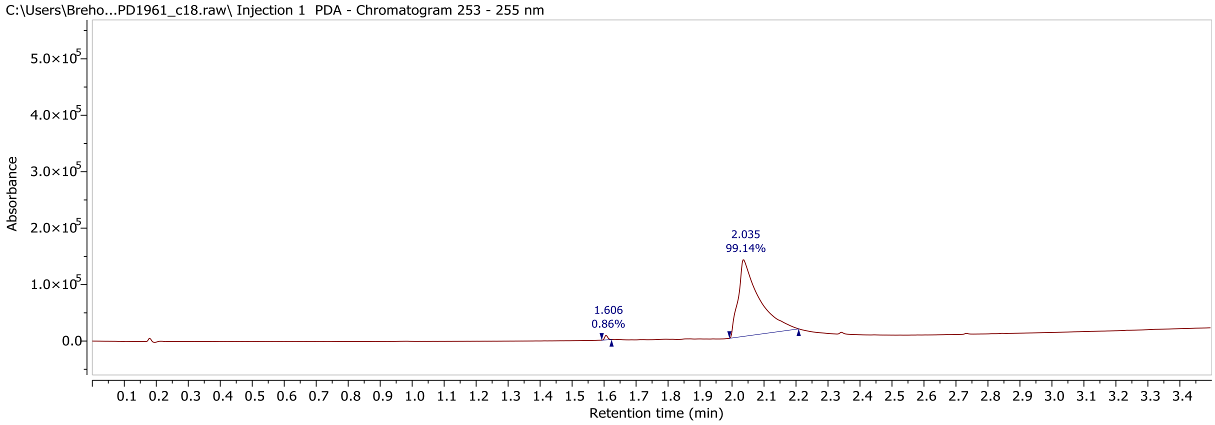

C:\Users\Breho...PD1961_c18.raw\ Injection 1 PDA - Chromatogram 253 - 255 nm
Absorbance
1.606
0.86%
2.035
99.14%
Retention time (min)

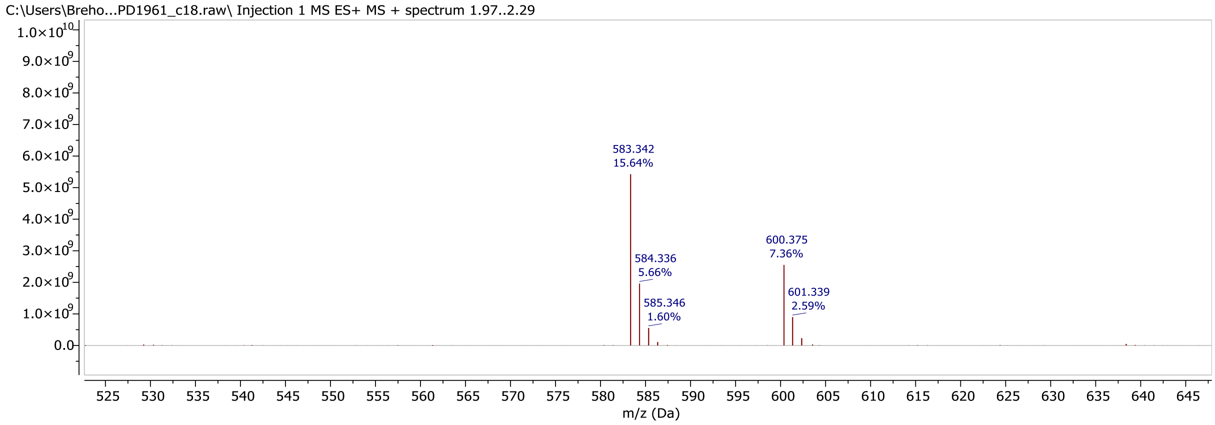

C:\Users\Breho...PD1961_c18.raw\ Injection 1 MS ES+ MS + spectrum 1.97..2.29
583.342
15.64%
584.336
5.66%
585.346
1.60%
600.375
7.36%
601.339
2.59%
m/z (Da)

N^6-((1r,4r)-4-aminocyclohexyl)-3-(4-(trifluoromethoxy)phenyl)-N^8-(4-(1-pyrrolidinylsulfonyl)phenyl)imidazo[1,2-*b*]pyridazine-6,8-diamine (34m)

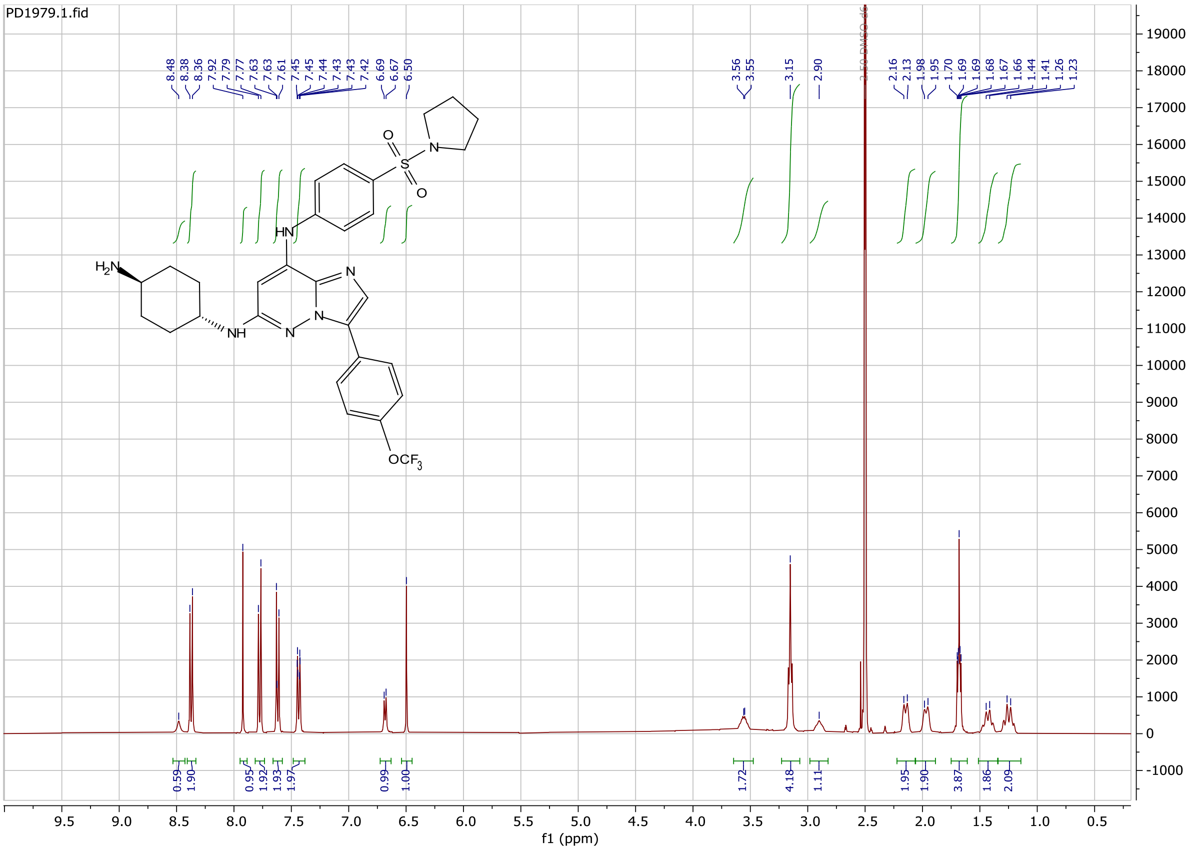

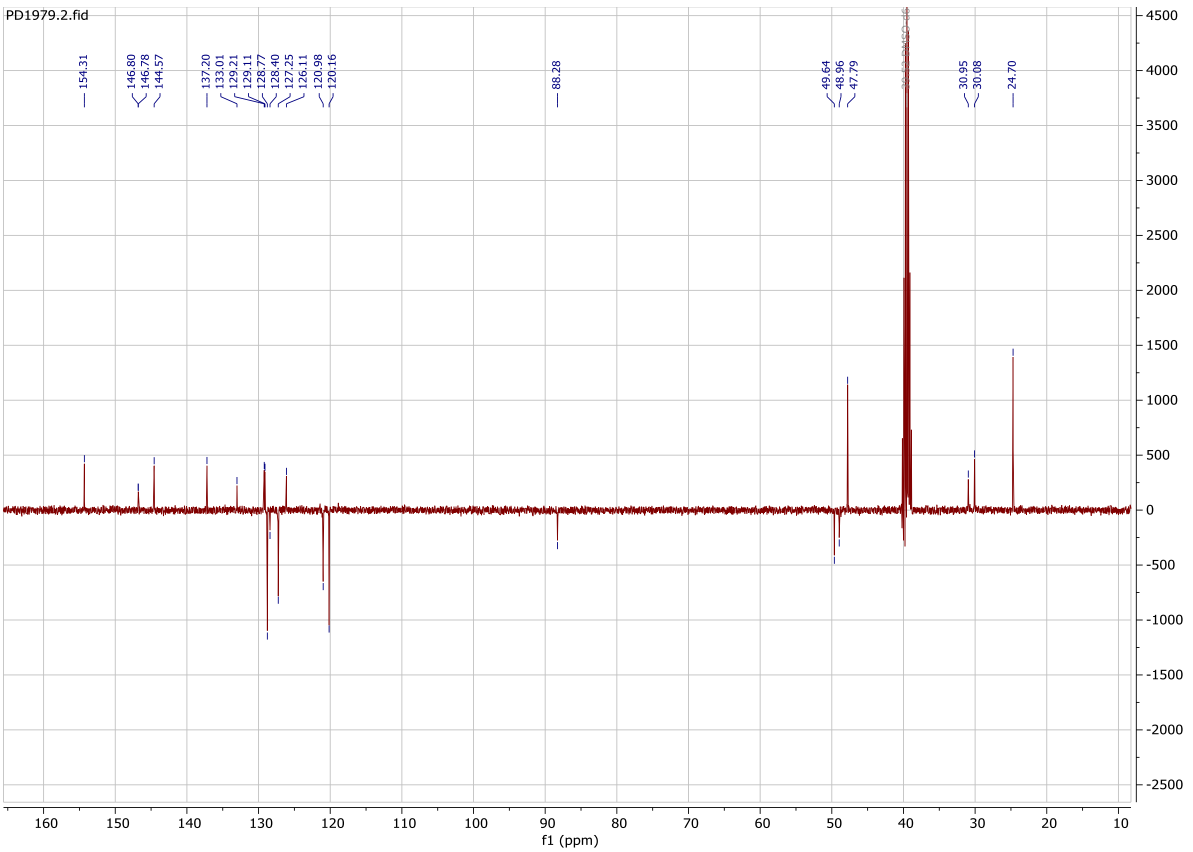

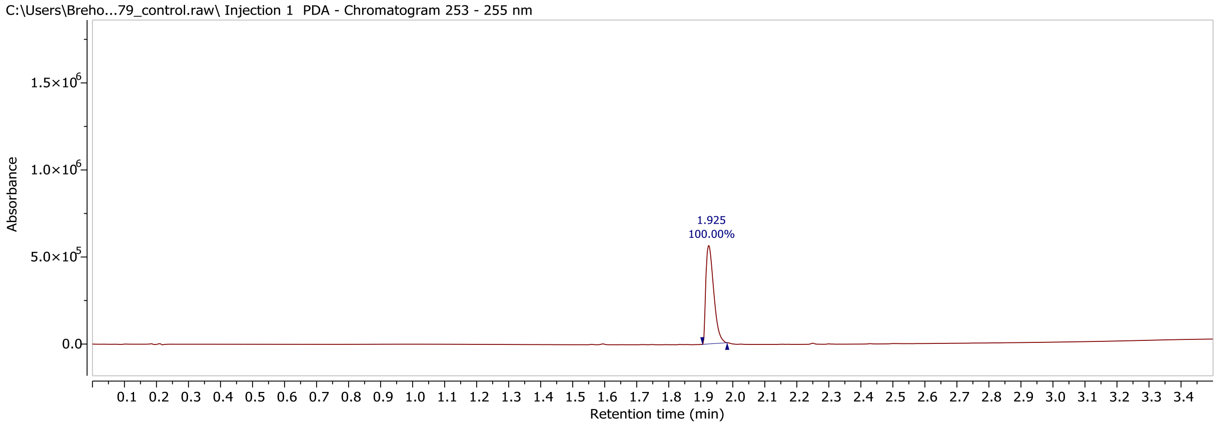

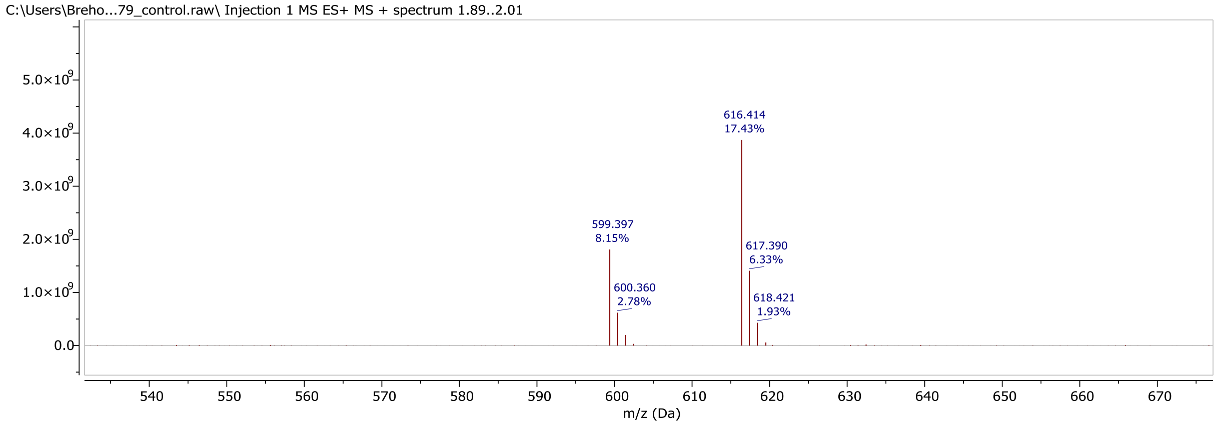

N^6-((1r,4r)-4-aminocyclohexyl)-3-(4-cyanophenyl)-N^8-(4-(1-pyrrolidinylsulfonyl)phenyl)imidazo[1,2-*b*]pyridazine-6,8-diamine (34n)

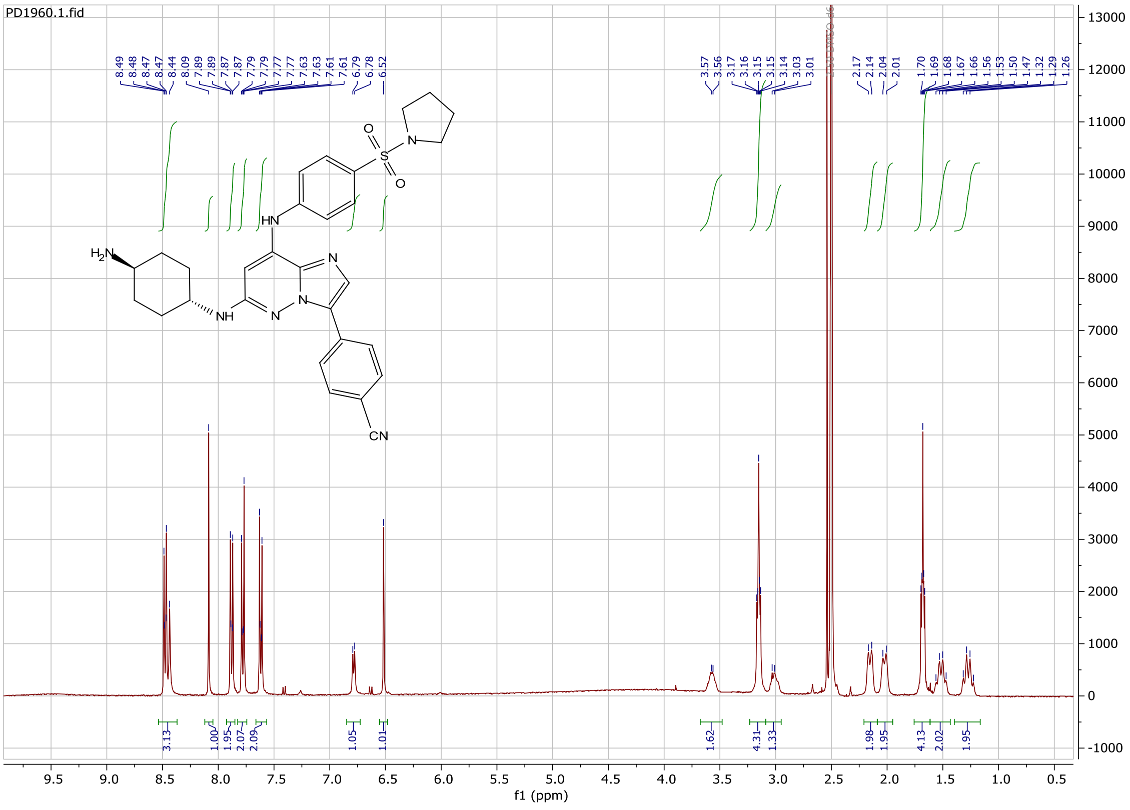

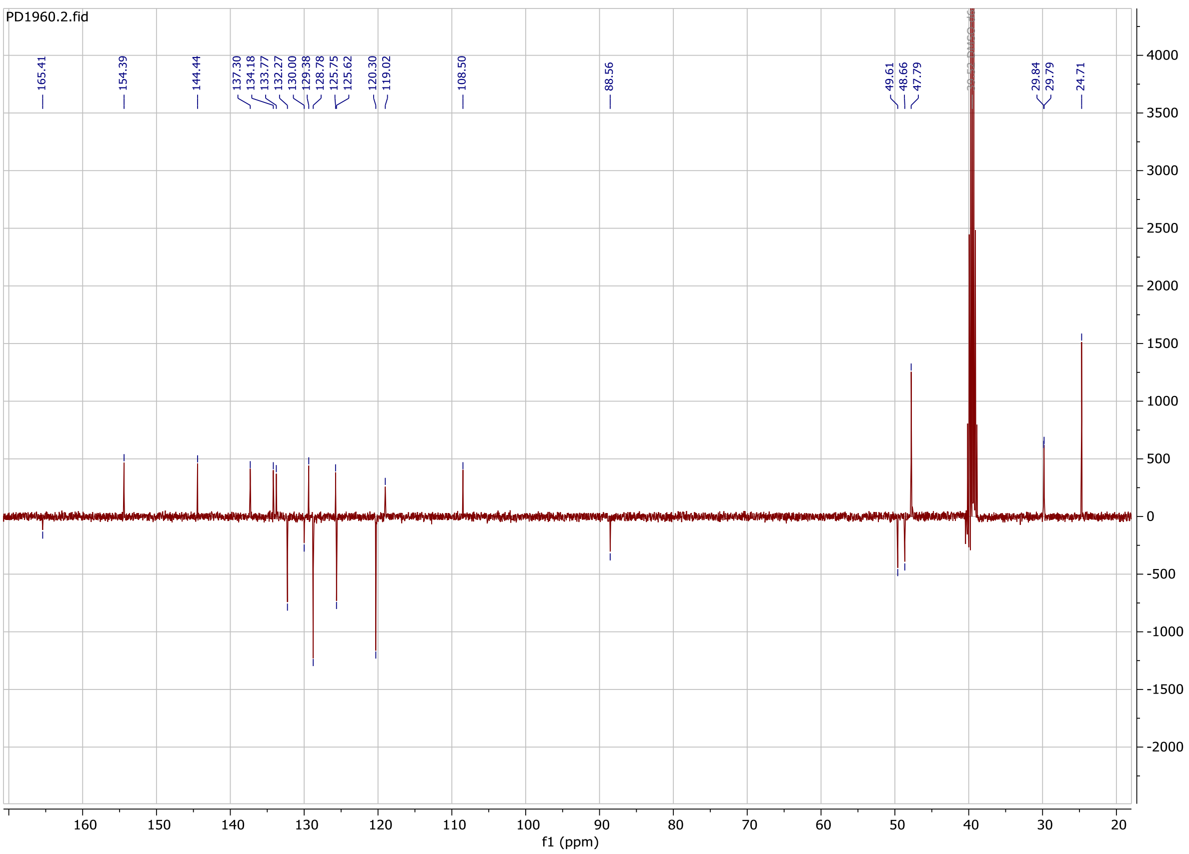
PD1960.2.fid
165.41
154.39
144.44
137.30
134.18
133.77
132.27
130.00
129.38
128.78
125.75
125.62
120.30
119.02
108.50
88.56
49.61
48.66
47.79
29.84
29.79
24.71
f1 (ppm)

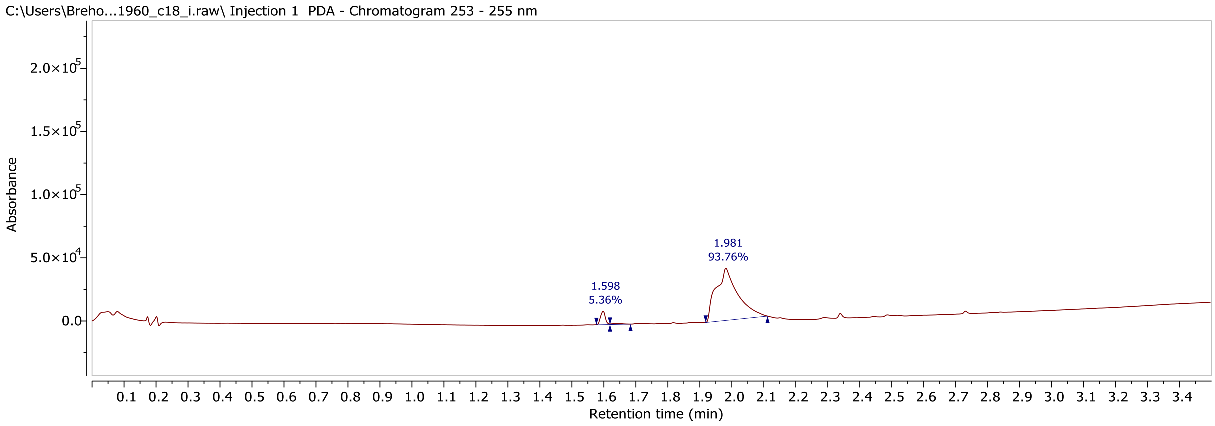
C:\Users\Breho...1960_c18_i.raw\ Injection 1 PDA - Chromatogram 253 - 255 nm
Absorbance
1.598
5.36%
1.981
93.76%
Retention time (min)

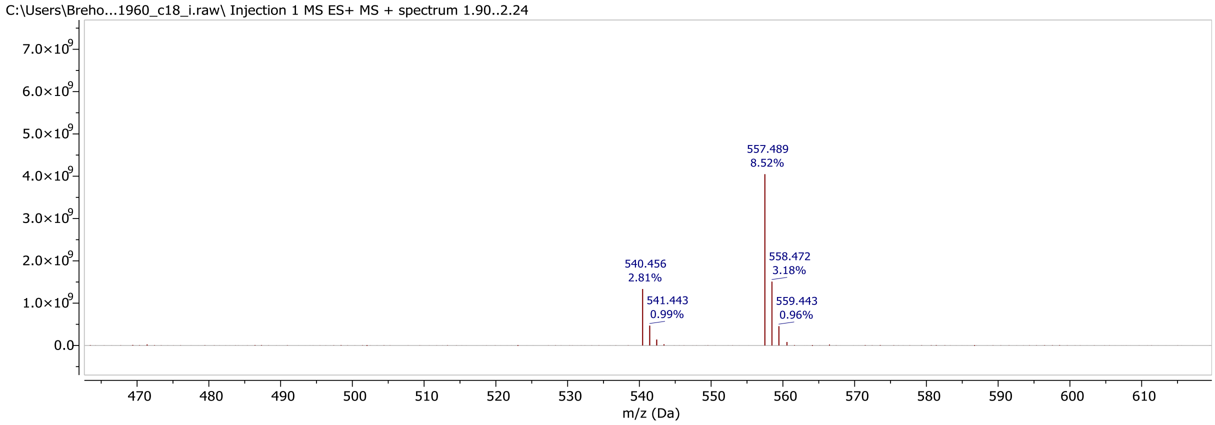
C:\Users\Breho...1960_c18_i.raw\ Injection 1 MS ES+ MS + spectrum 1.90..2.24
557.489
8.52%
540.456
2.81%
558.472
3.18%
541.443
0.99%
559.443
0.96%
m/z (Da)

N^6-((1r,4r)-4-aminocyclohexyl)-3-(3-(*N,N*-dimethylcarbamoyl)phenyl)-N^8-(4-(1-pyrrolidinylsulfonyl)phenyl)imidazo[1,2-*b*]pyridazine-6,8-diamine (34o)

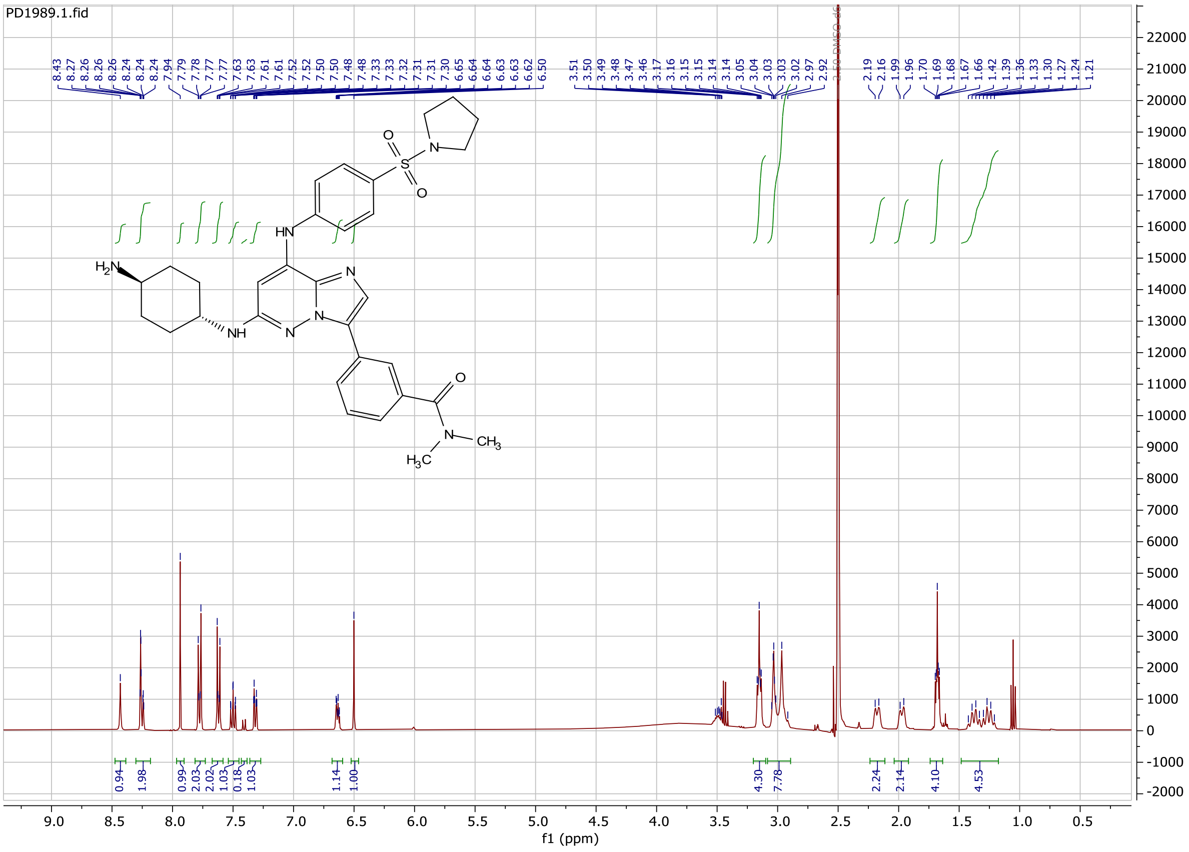

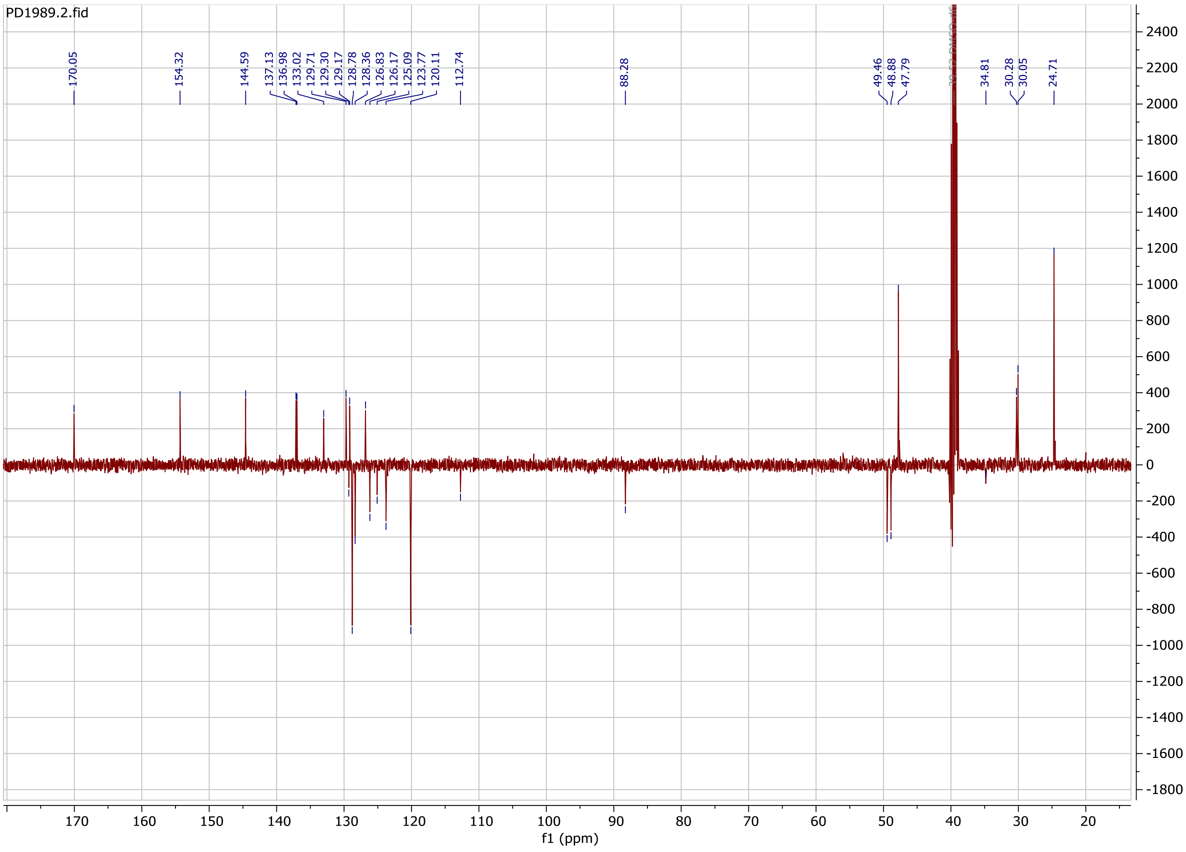

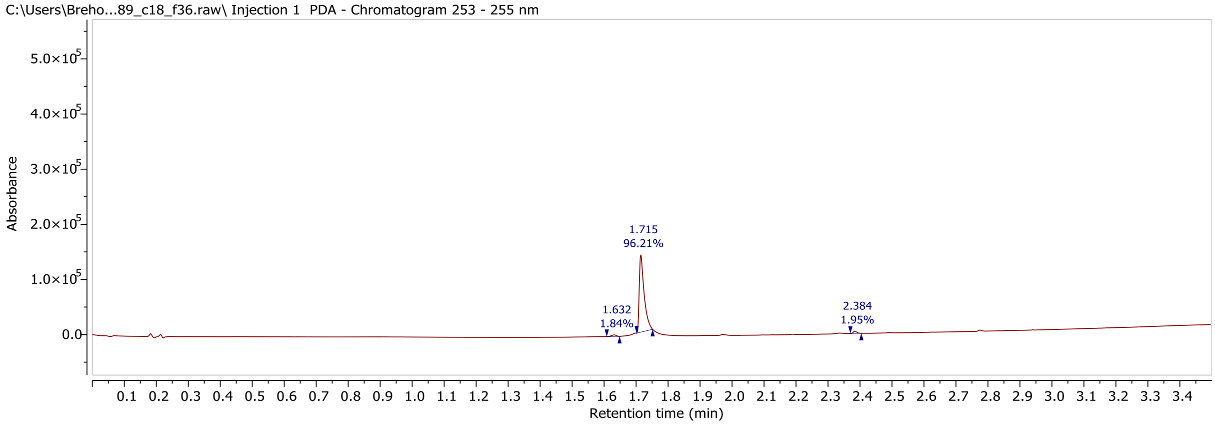

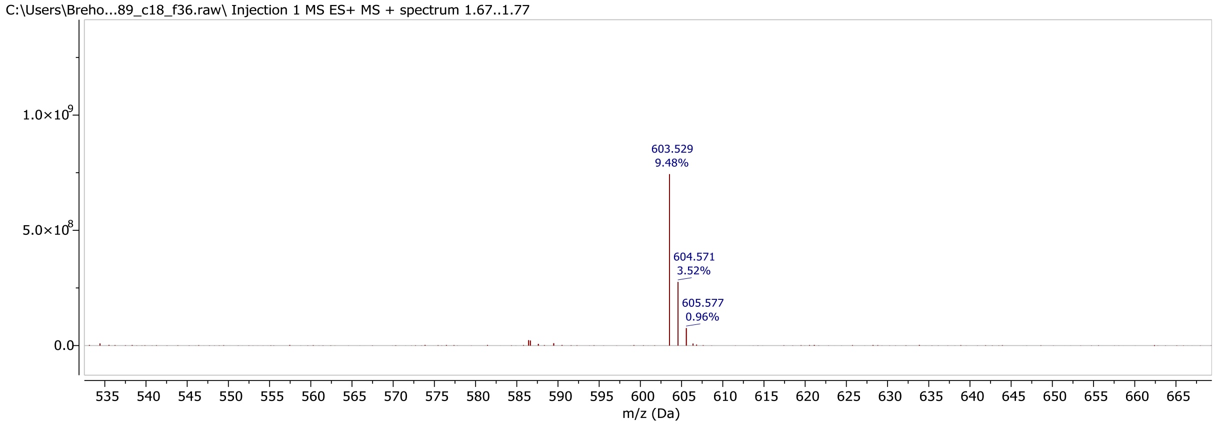

N^6-((1r,4r)-4-aminocyclohexyl)-3-(3-(*N*,*N*-dimethylsulfamoyl)phenyl)-N^8-(4-(1-pyrrolidinylsulfonyl)phenyl)imidazo[1,2-*b*]pyridazine-6,8-diamine (34p)

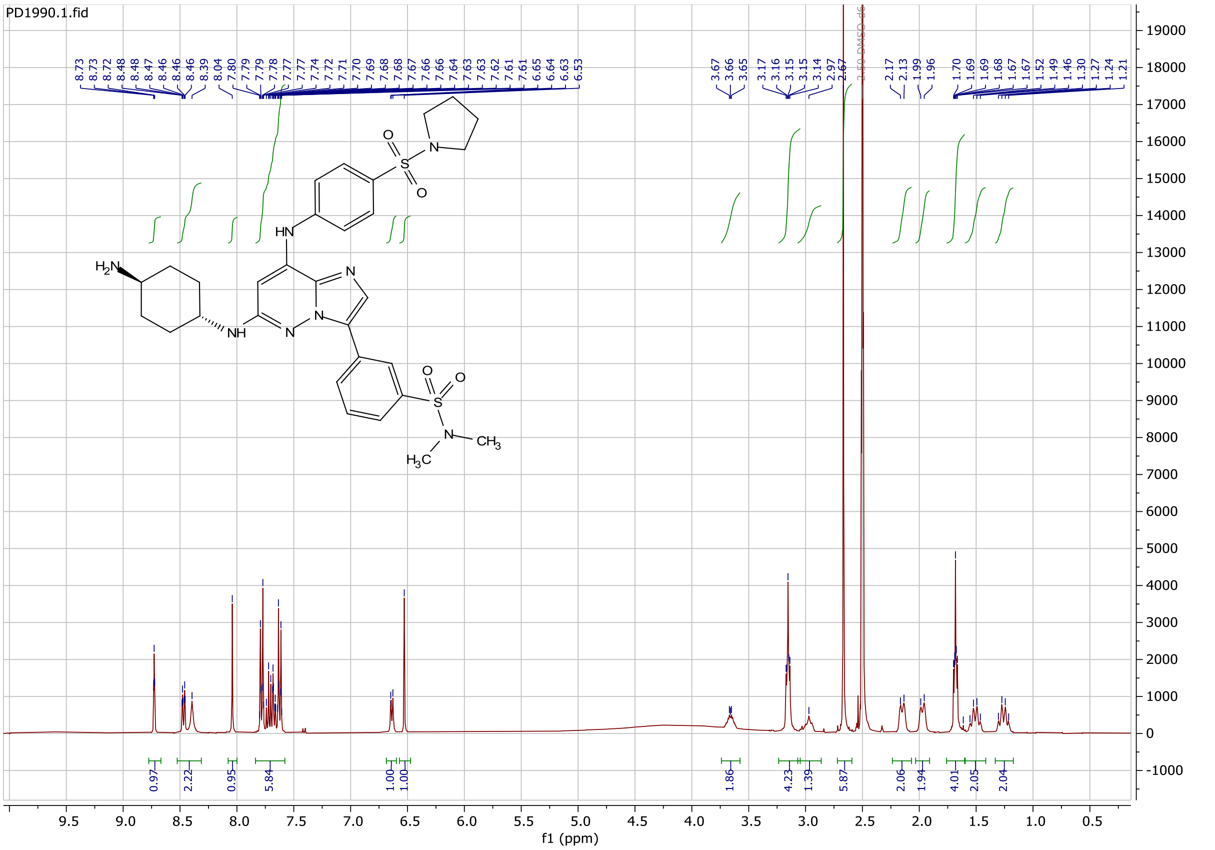

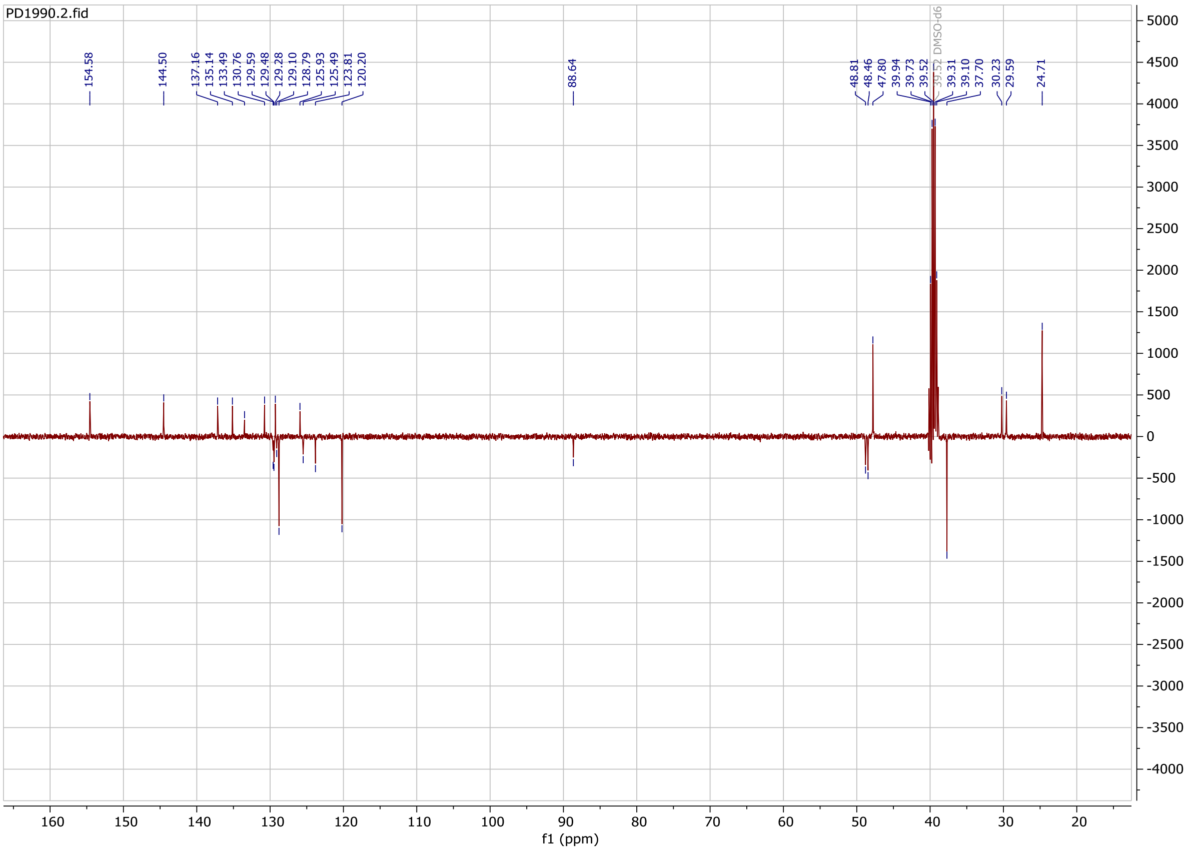
PD1990.2.fid
154.58
144.50
137.16
135.14
133.49
130.76
129.59
129.48
129.28
129.10
128.79
125.93
125.49
123.81
120.20
88.64
48.81
48.46
47.80
39.94
39.73
39.52 DMSO-d6
39.31
39.10
37.70
30.23
29.59
24.71
f1 (ppm)

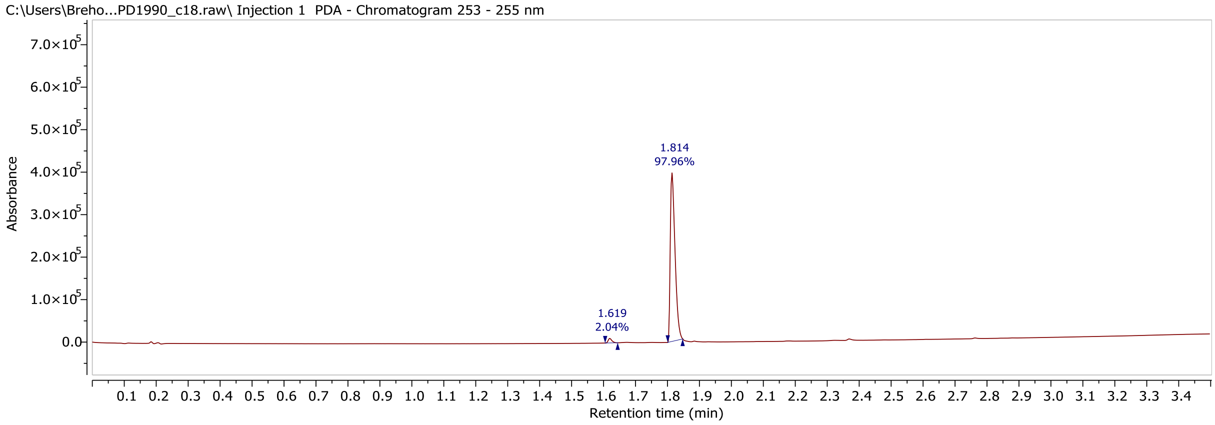
C:\Users\Breho...PD1990_c18.raw\ Injection 1 PDA - Chromatogram 253 - 255 nm
Absorbance
1.814
97.96%
1.619
2.04%
Retention time (min)

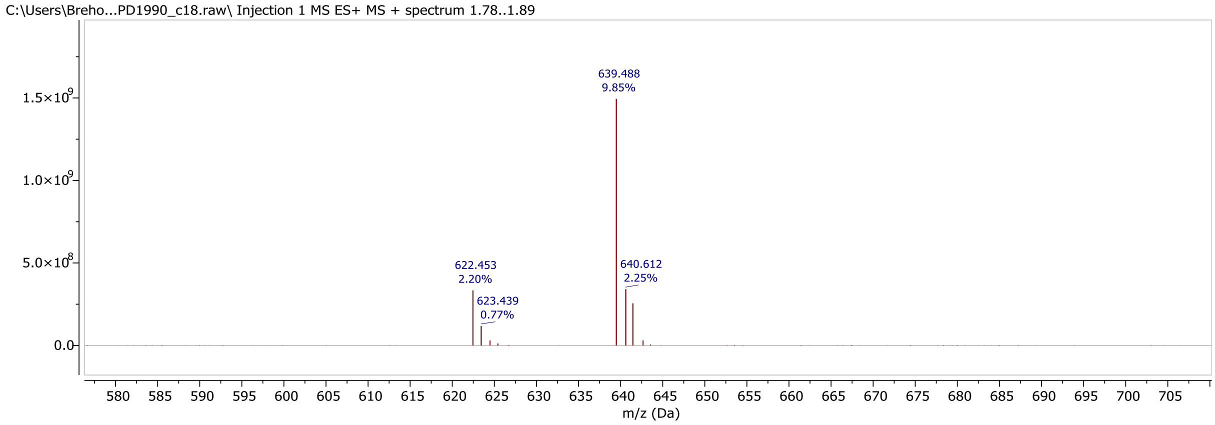
C:\Users\Breho...PD1990_c18.raw\ Injection 1 MS ES+ MS + spectrum 1.78..1.89
639.488
9.85%
622.453
2.20%
623.439
0.77%
640.612
2.25%
m/z (Da)

N^6-((1r,4r)-4-aminocyclohexyl)-3-(thiophen-3-yl)-N^8-(4-(1-pyrrolidinylsulfonyl)phenyl)imidazo[1,2-*b*]pyridazine-6,8-diamine (34q)

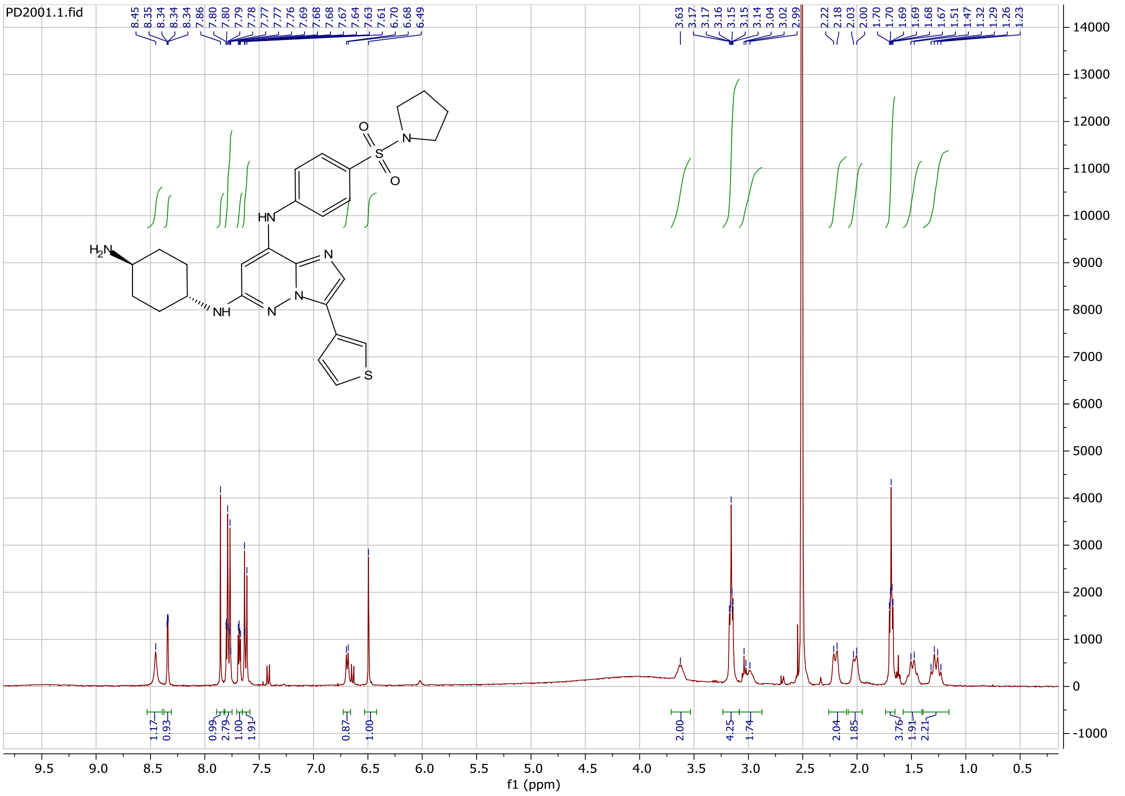

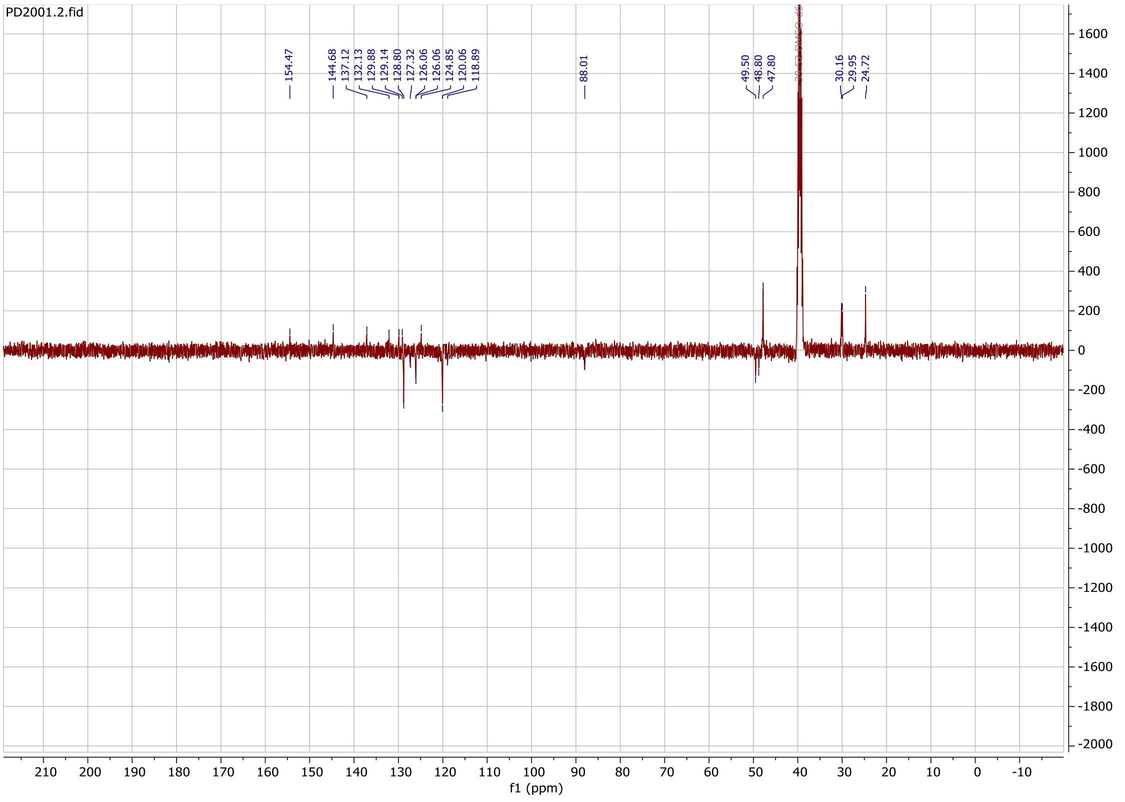

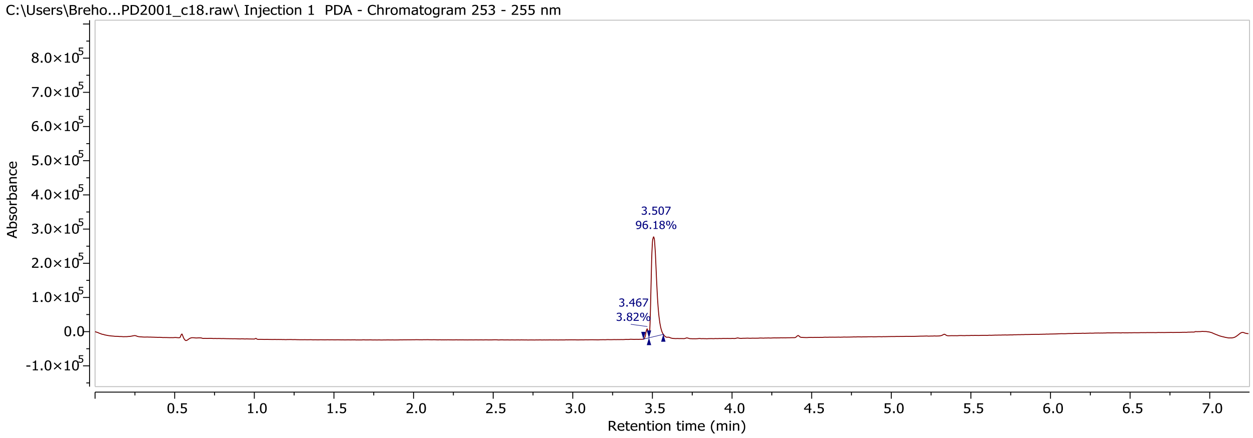

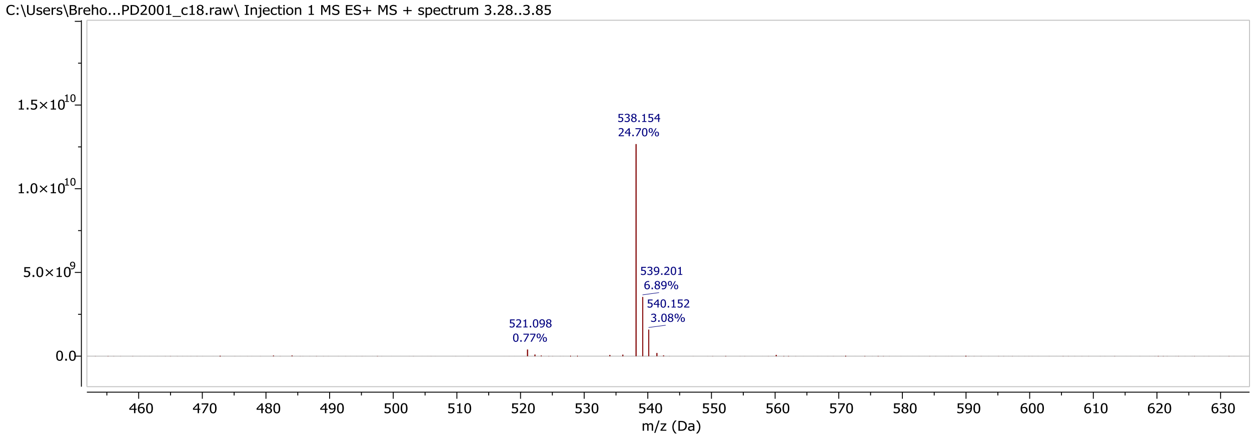

N[6]-((1r,4r)-4-aminocyclohexyl)-3-(furan-3-yl)-*N*[8]-(4-(1-pyrrolidinylsulfonyl)phenyl)imidazo[1,2-*b*]pyridazine-6,8-diamine (34r)

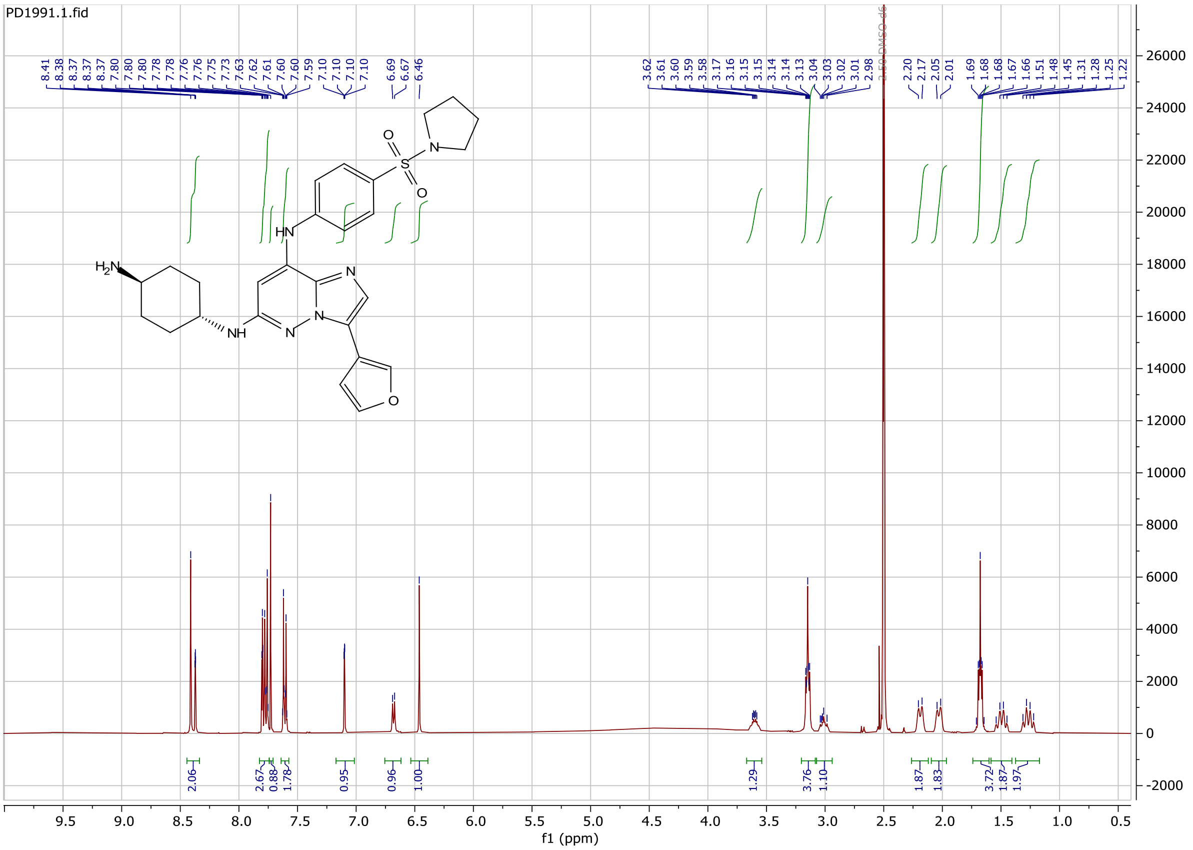

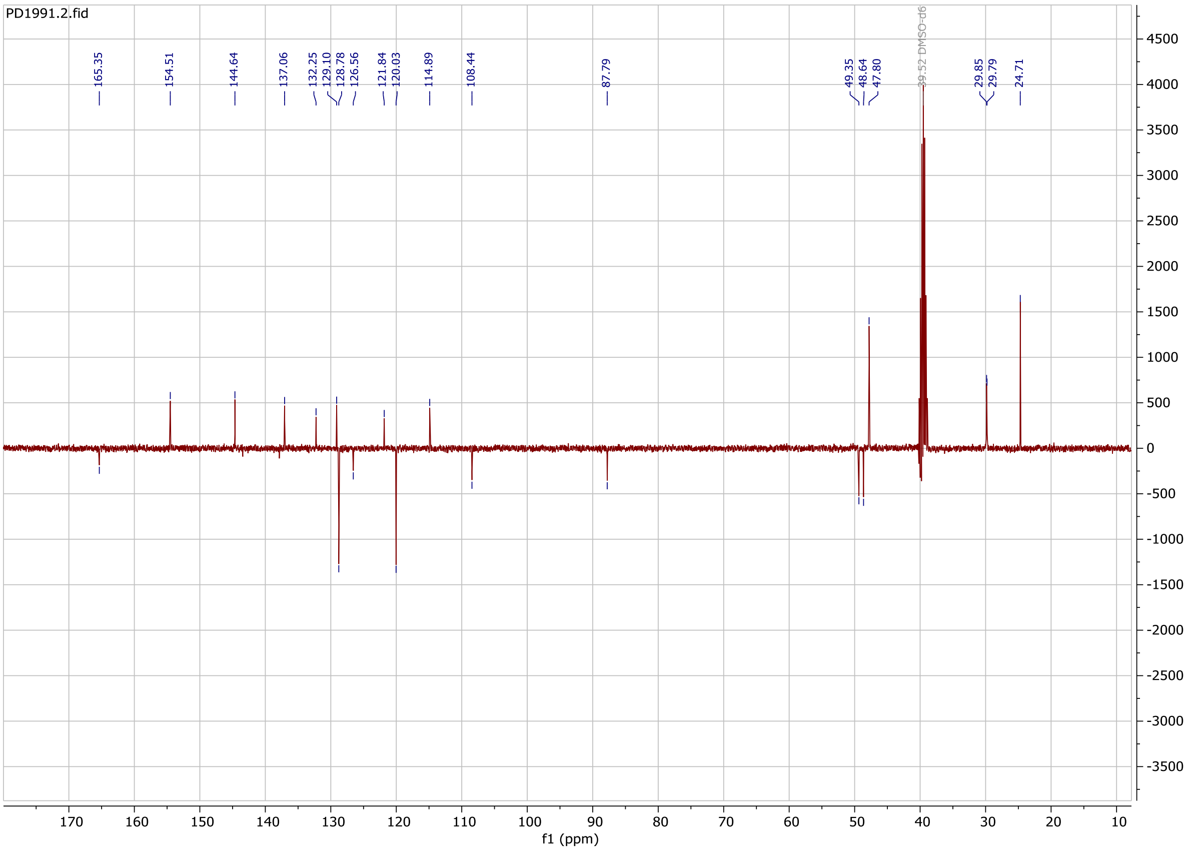
PD1991.2.fid
165.35
154.51
144.64
137.06
132.25
129.10
128.78
126.56
121.84
120.03
114.89
108.44
87.79
49.35
48.64
47.80
39.52 DMSO-d6
29.85
29.79
24.71
f1 (ppm)

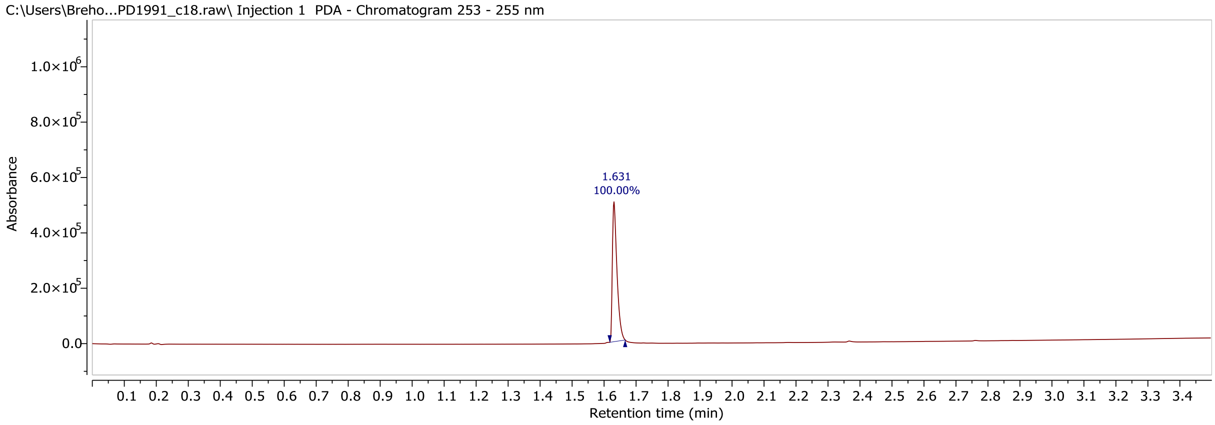
C:\Users\Breho...PD1991_c18.raw\ Injection 1 PDA - Chromatogram 253 - 255 nm
Absorbance
1.631
100.00%
Retention time (min)

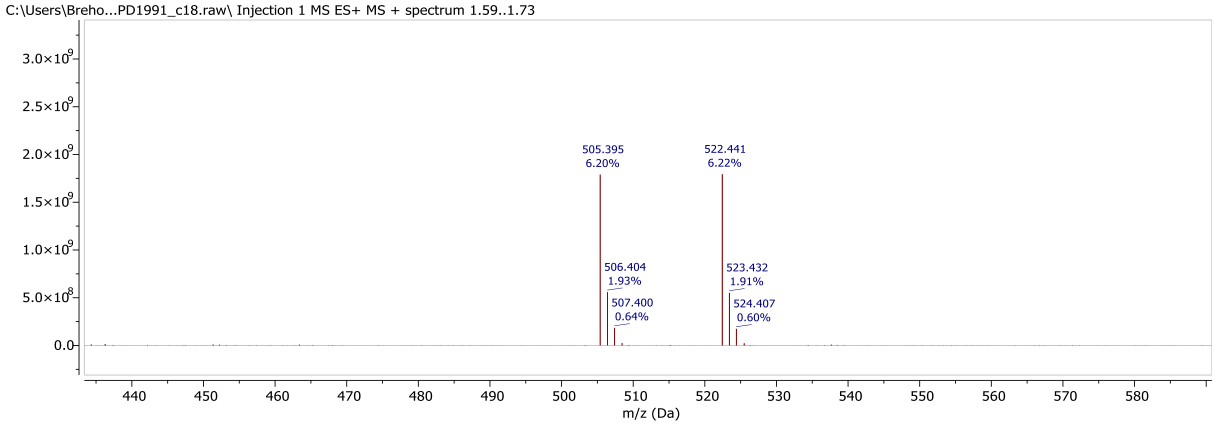
C:\Users\Breho...PD1991_c18.raw\ Injection 1 MS ES+ MS + spectrum 1.59..1.73
505.395
6.20%
506.404
1.93%
507.400
0.64%
522.441
6.22%
523.432
1.91%
524.407
0.60%
m/z (Da)

N^6-((1r,4r)-4-aminocyclohexyl)-3-amino-N^8-(4-(1-pyrrolidinylsulfonyl)phenyl)imidazo[1,2-*b*]pyridazine-6,8-diamine (39)

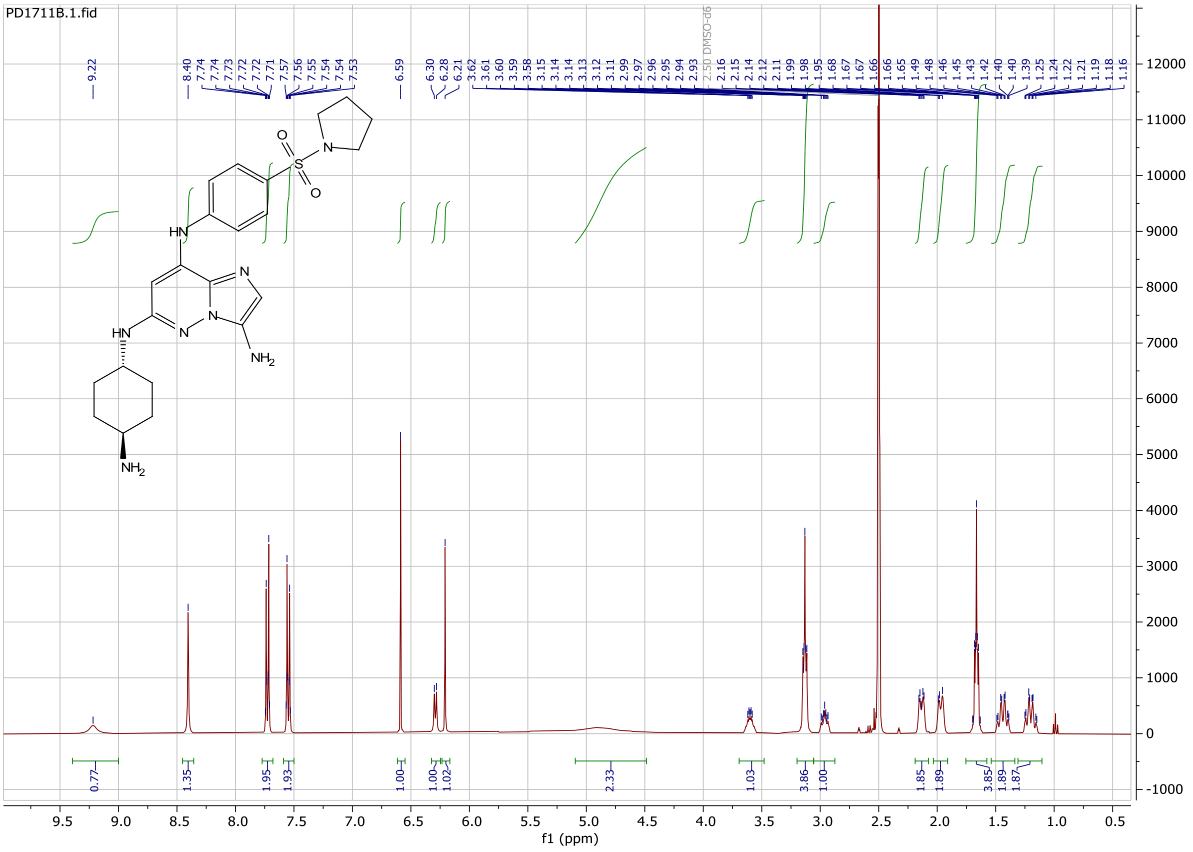

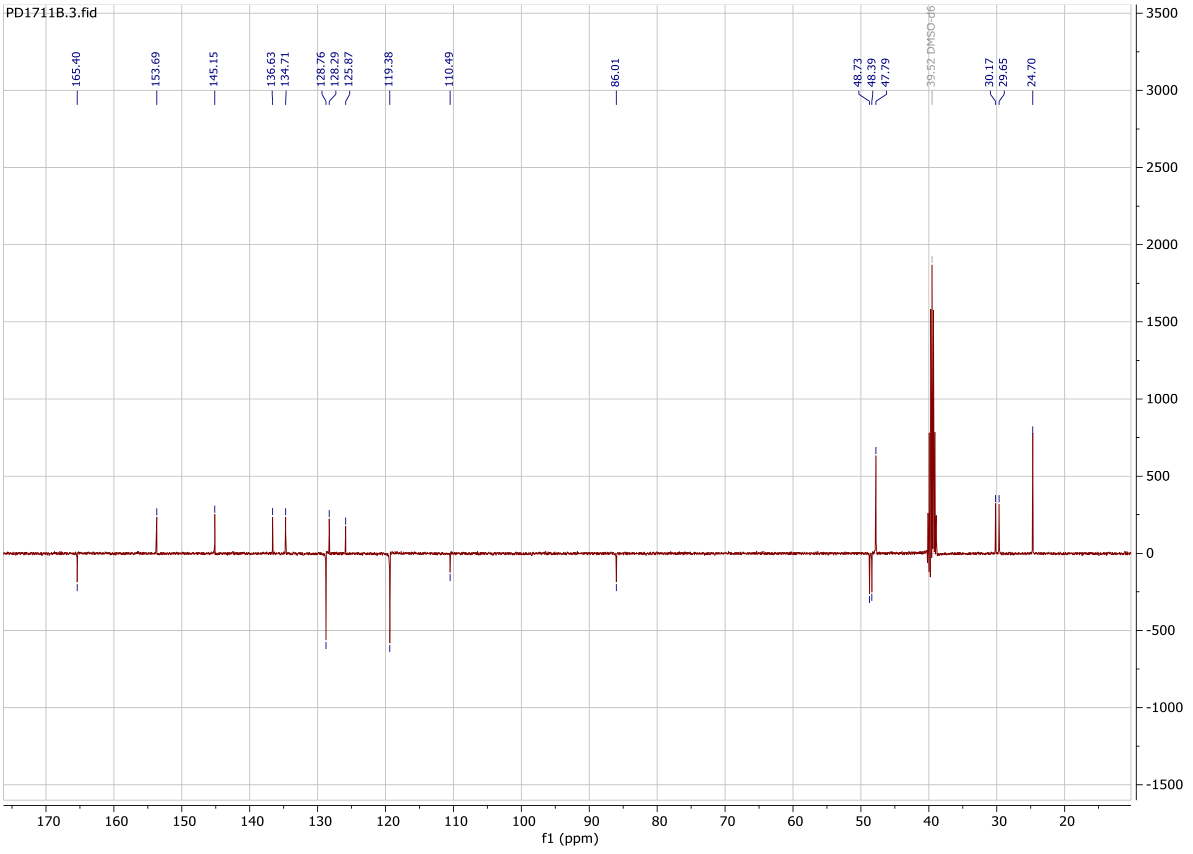

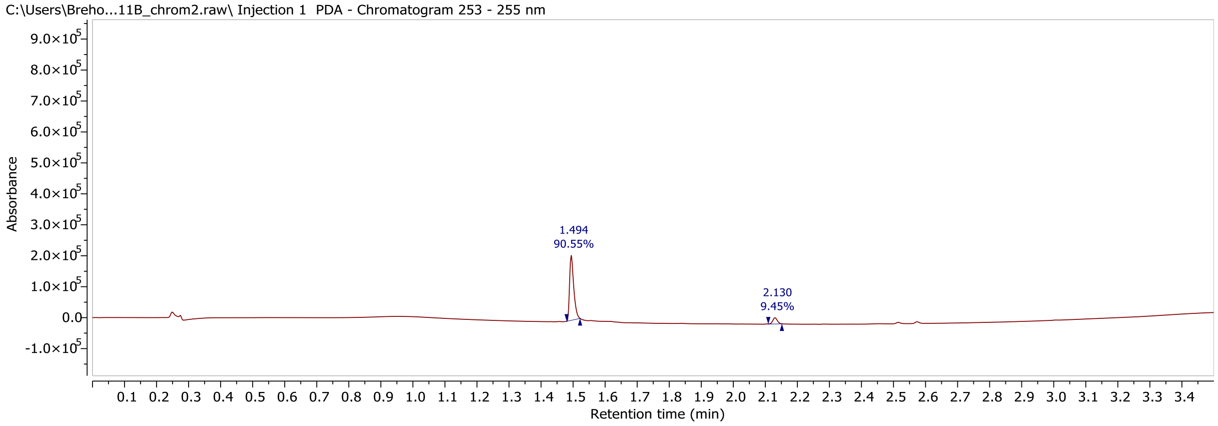

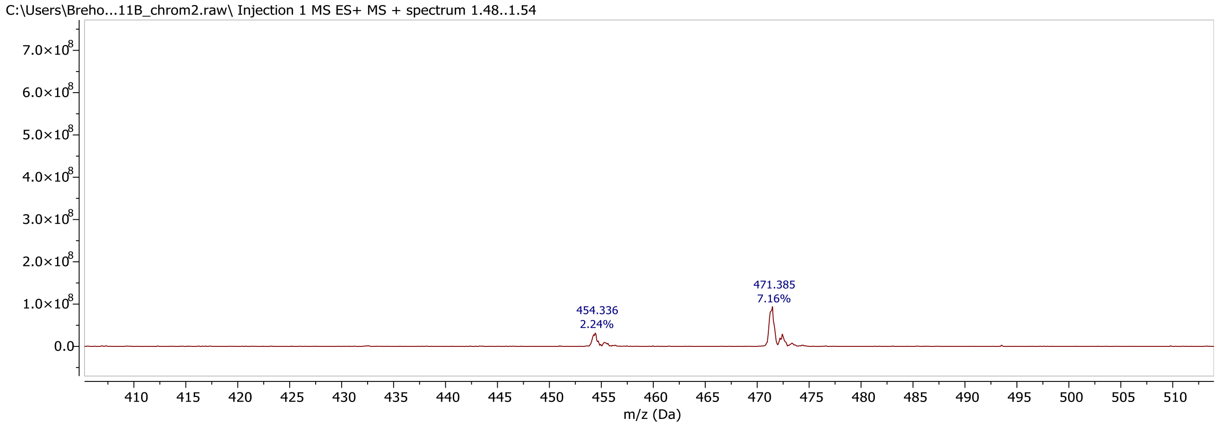

N[6]-((1r,4r)-4-aminocyclohexyl)-3-(1*H*-pyrazole-4-yl)-*N*[8]-(4-(1-pyrrolidinylsulfonyl)phenyl)imidazo[1,2-*b*]pyridazine-6,8-diamine (42a)

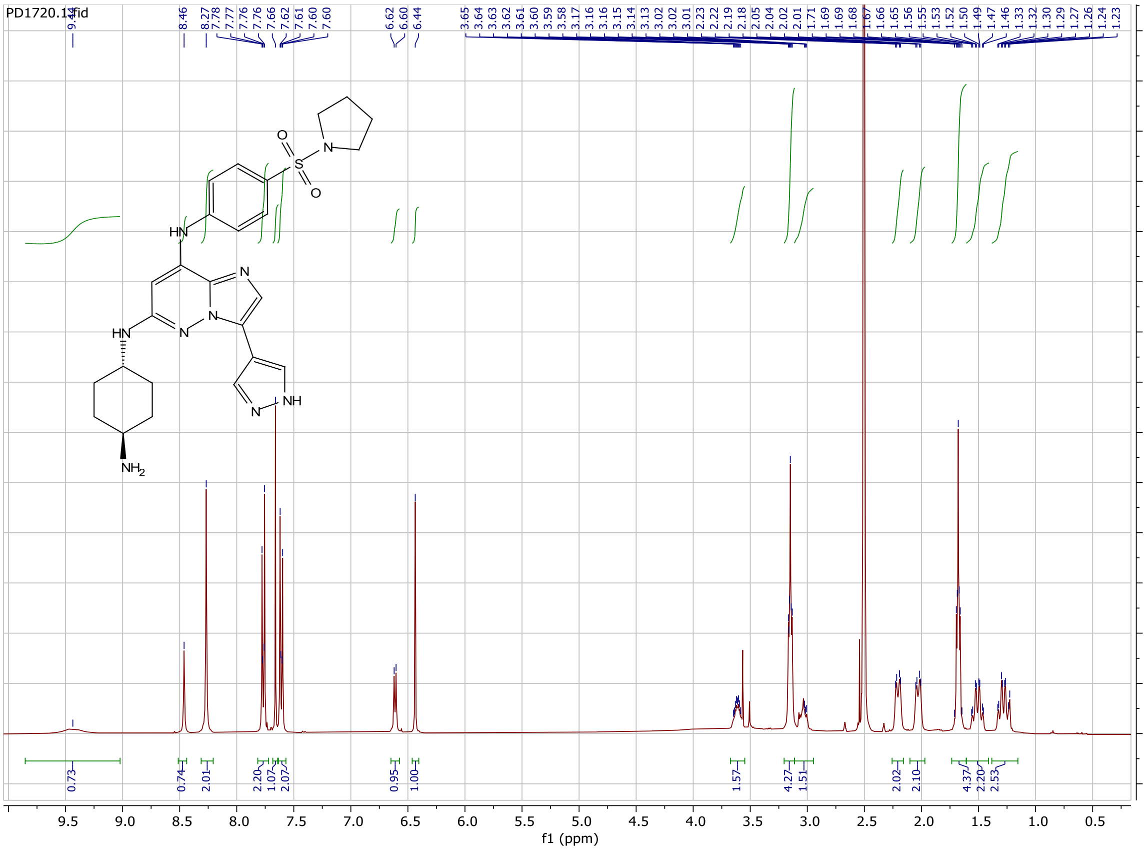

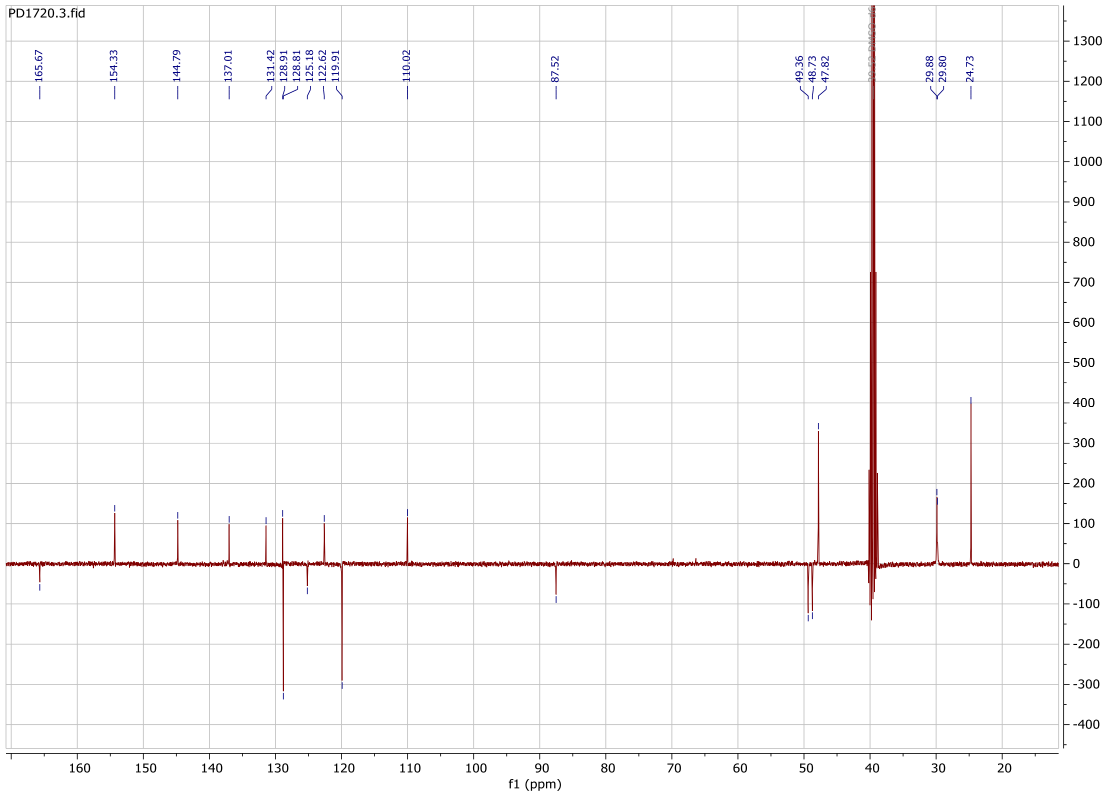

C:\Users\Breho...\PD1720_c2.raw\ Injection 1 PDA - Chromatogram 253 - 255 nm

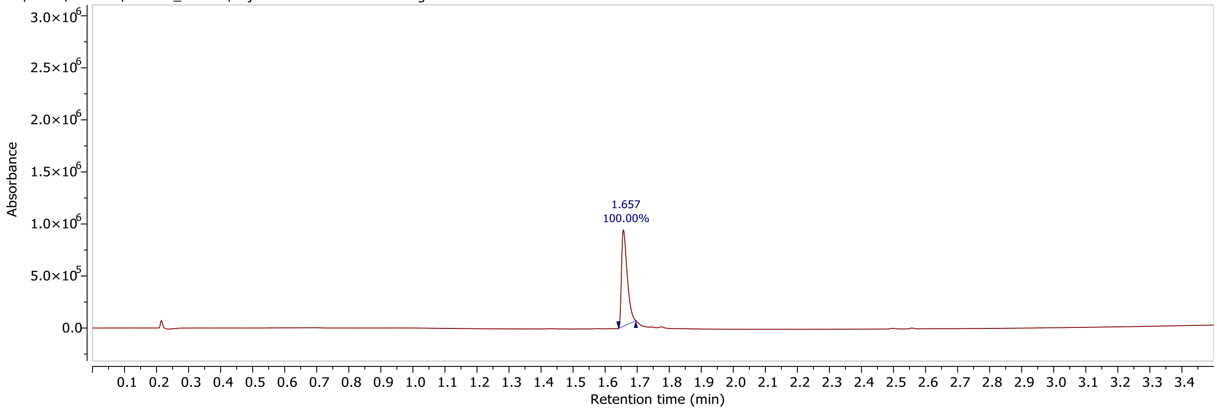

C:\Users\Breho...\PD1720_c2.raw\ Injection 1 MS ES+ MS + spectrum 1.58..1.75

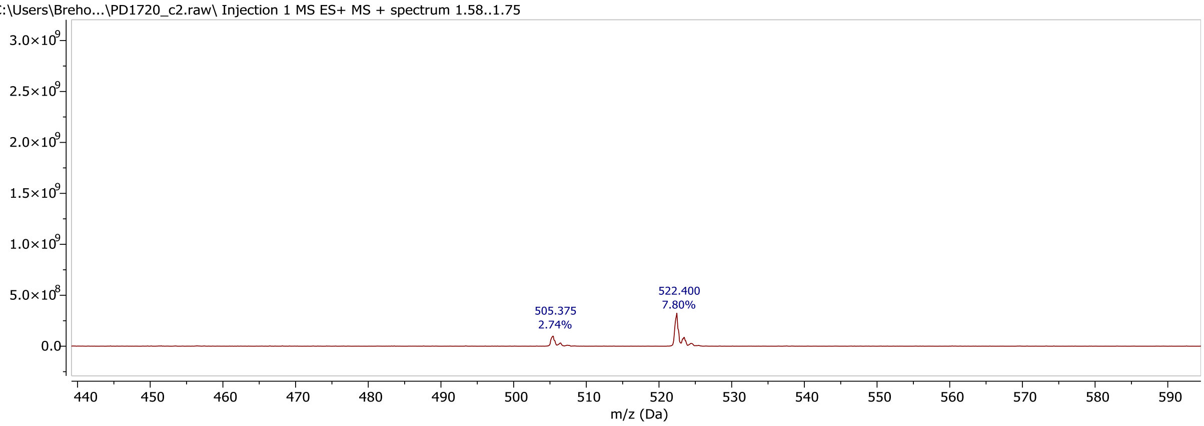

N^6-((1r,4r)-4-aminocyclohexyl)-N^8-(4-(1-pyrrolidinylsulfonyl)phenyl)imidazo[1,2-*b*]pyridazine-6,8-diamine (42b)

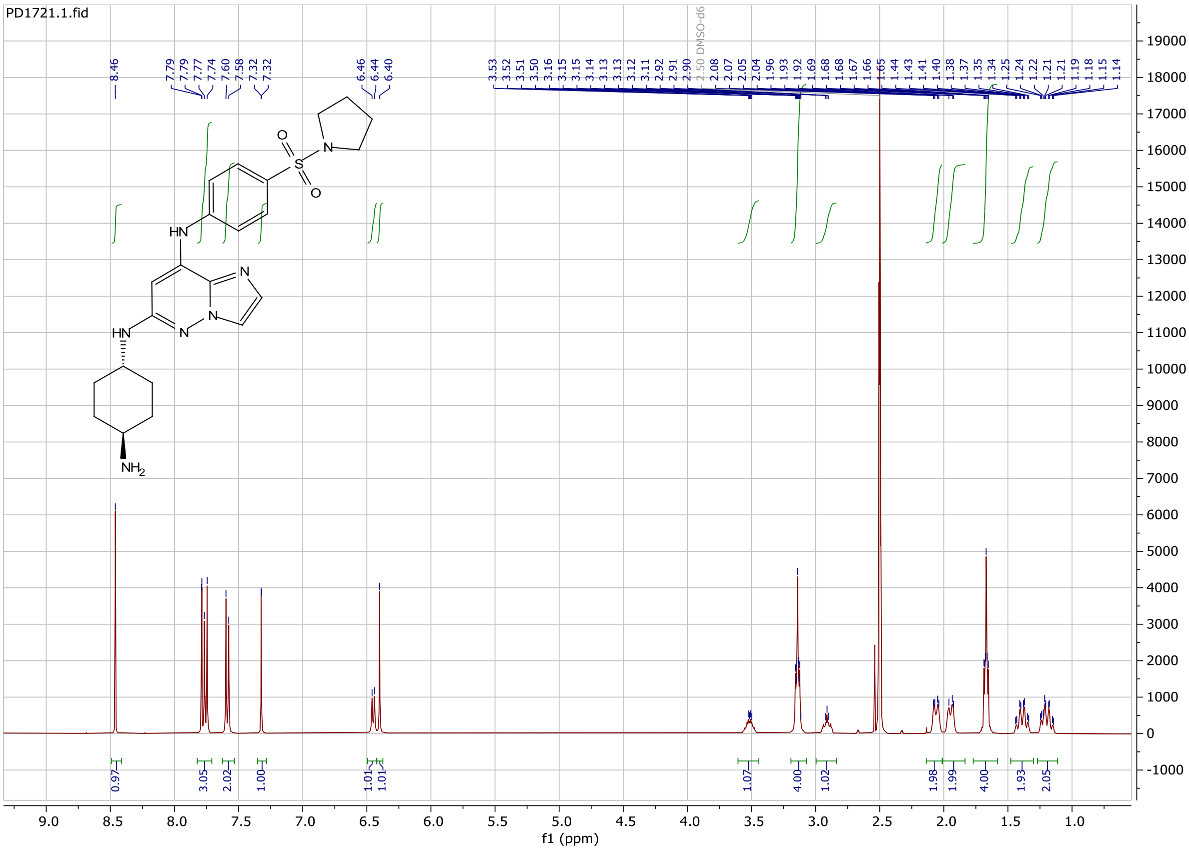

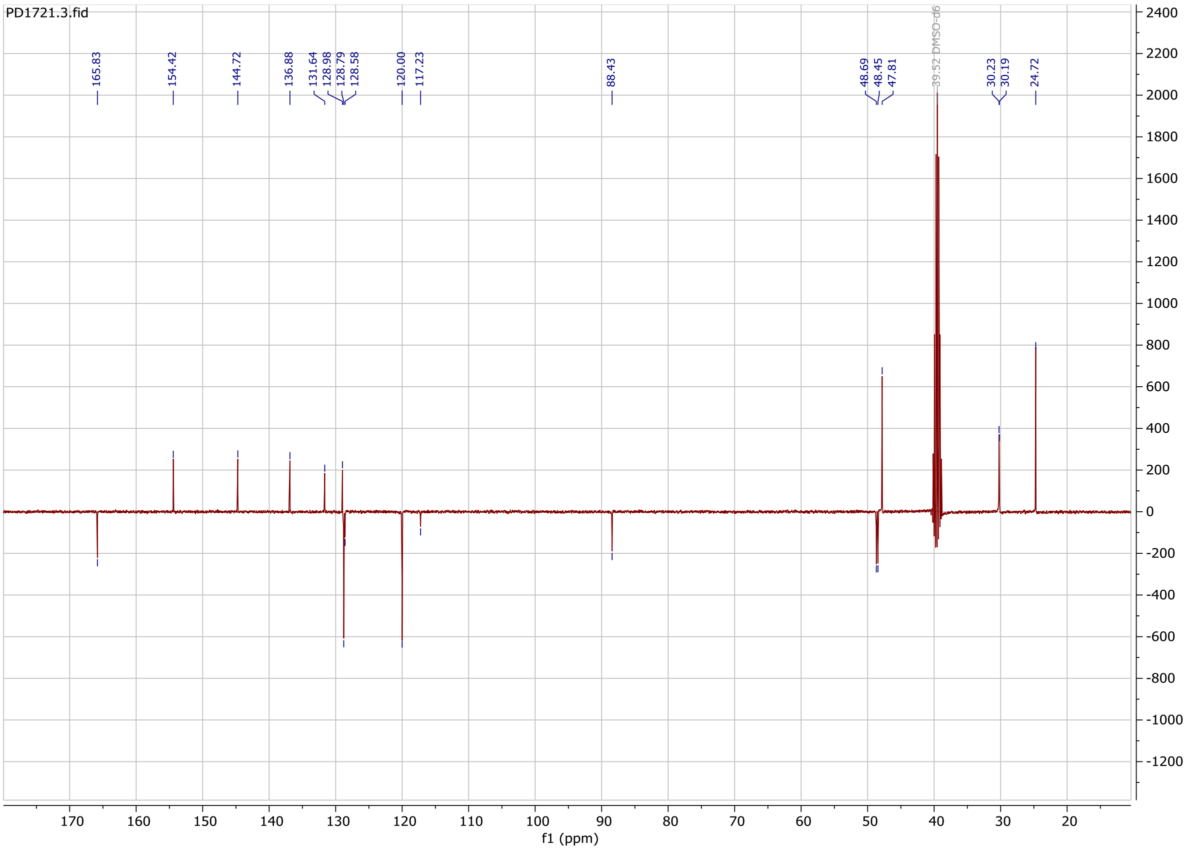

C:\Users\Breho...21_control.raw\ Injection 1 PDA - Chromatogram 253 - 255 nm

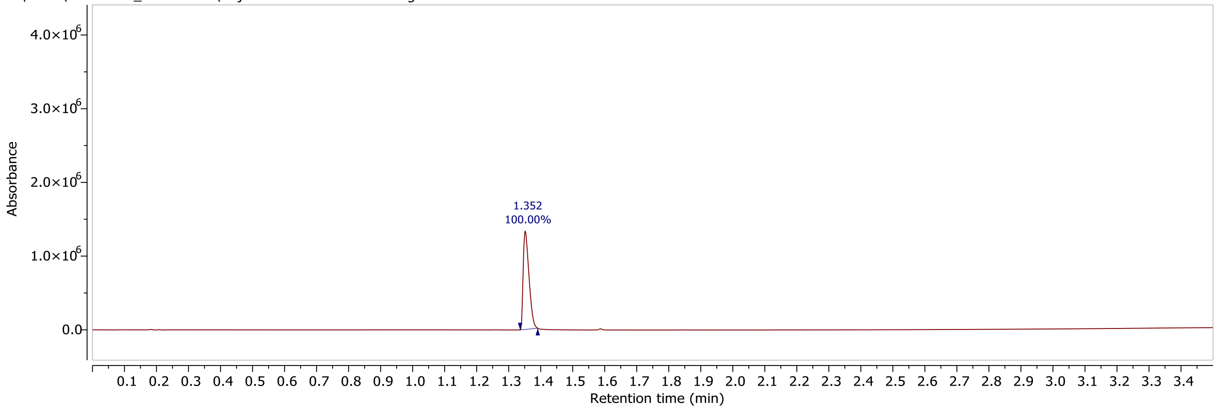

C:\Users\Breho...21_control.raw\ Injection 1 MS ES+ MS + spectrum 1.30..1.43

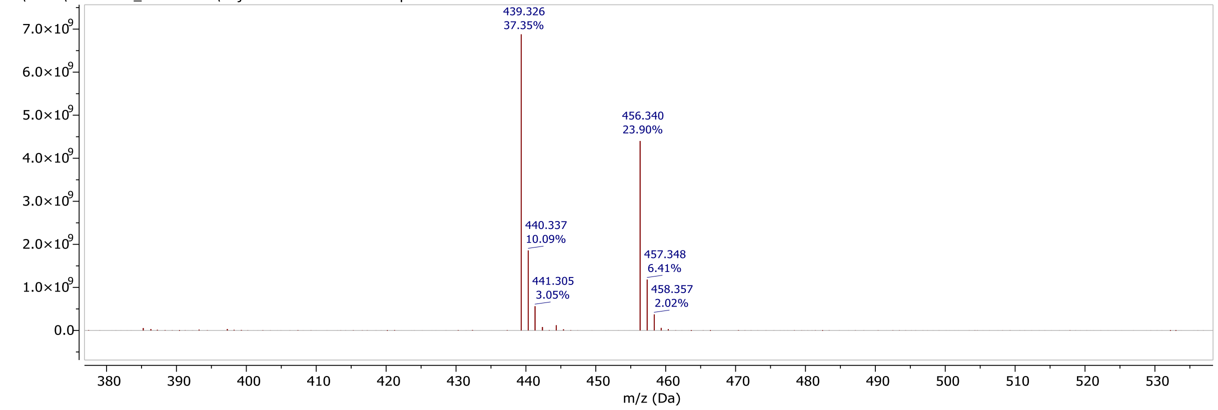

N^2-(6-aminopyridin-3-yl)-7-(cyclohex-1-en-1-yl)-N^4-[4-(1-pyrrolidinylsulfonyl)phenyl]thieno[3,2-*d*]pyrimidin-2,4-diamine (5a)

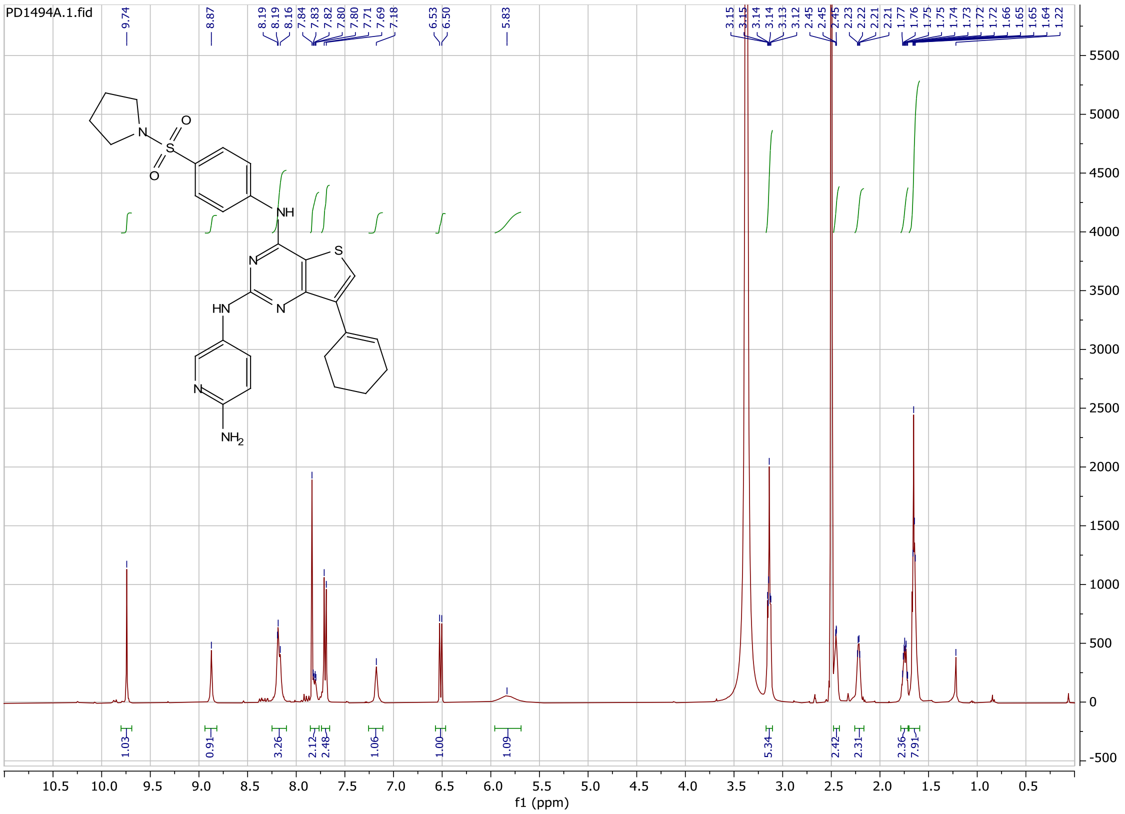

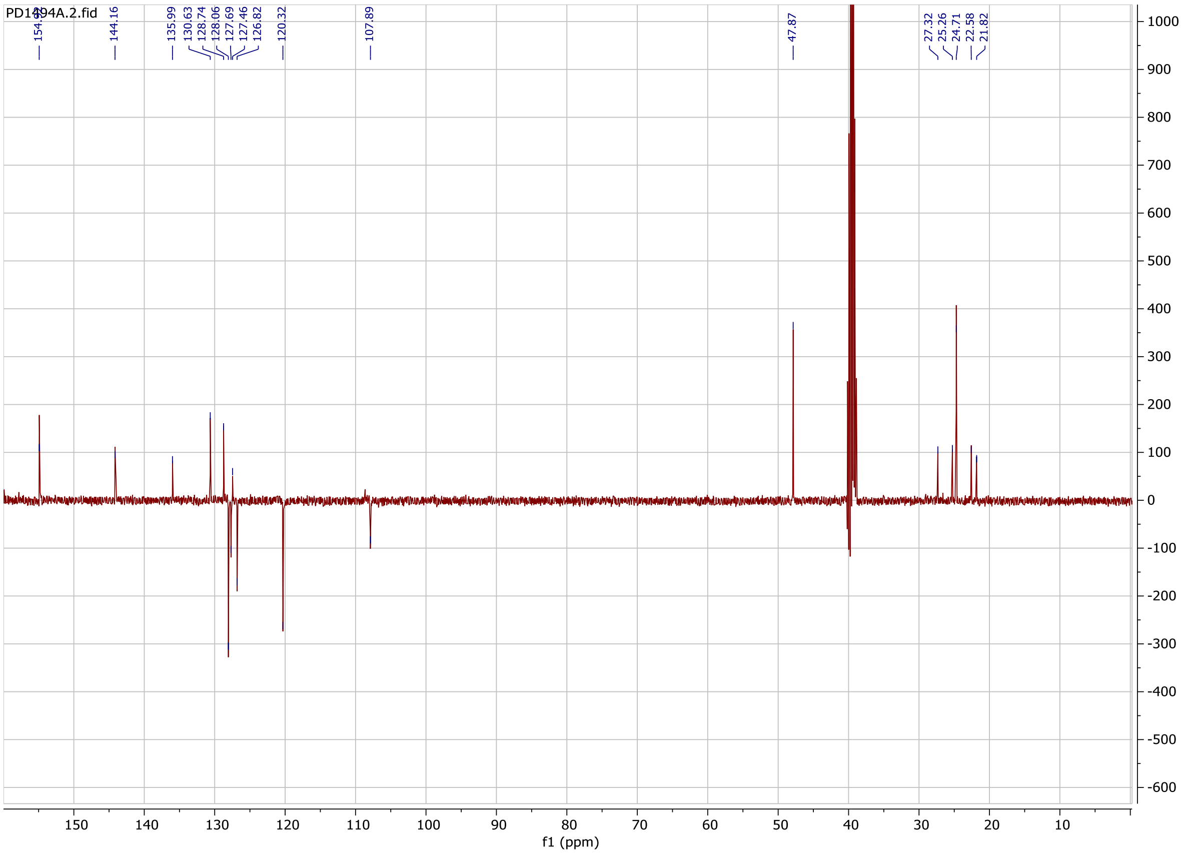
PD1494A.2.fid
154.[illegible]
144.16
135.99
130.63
128.74
128.06
127.69
127.46
126.82
120.32
107.89
47.87
27.32
25.26
24.71
22.58
21.82
f1 (ppm)

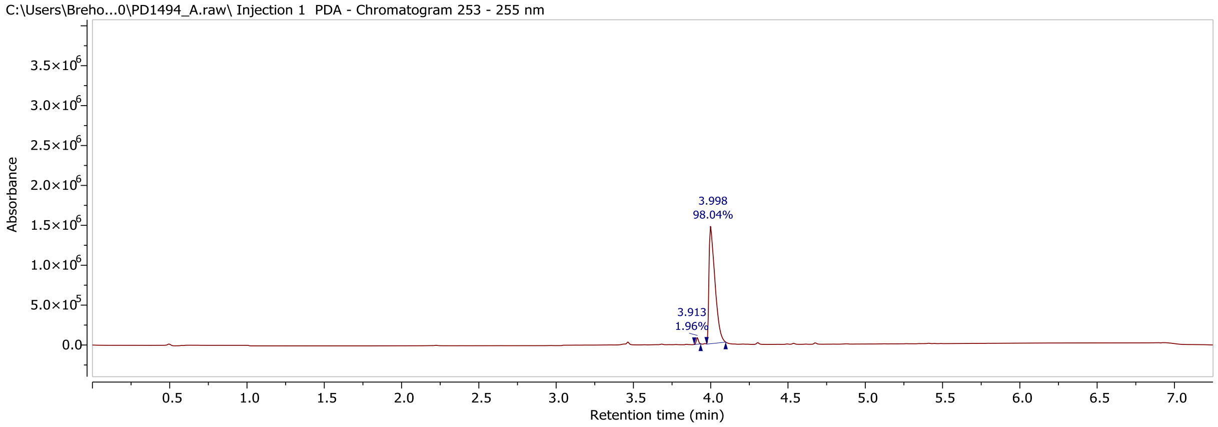
C:\Users\Breho...0\PD1494_A.raw\ Injection 1 PDA - Chromatogram 253 - 255 nm
Absorbance
3.998
98.04%
3.913
1.96%
Retention time (min)

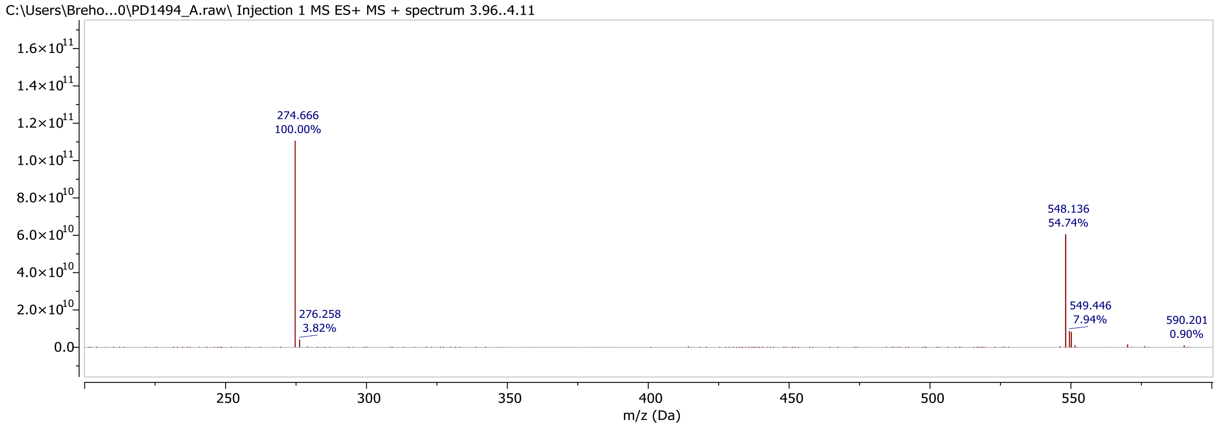
C:\Users\Breho...0\PD1494_A.raw\ Injection 1 MS ES+ MS + spectrum 3.96..4.11
274.666
100.00%
276.258
3.82%
548.136
54.74%
549.446
7.94%
590.201
0.90%
m/z (Da)

N^2-(5-aminopyridin-2-yl)-7-(cyclohex-1-en-1-yl)-N^4-[4-(1-pyrrolidinylsulfonyl)phenyl]thieno[3,2-d]pyrimidin-2,4-diamine (5b)

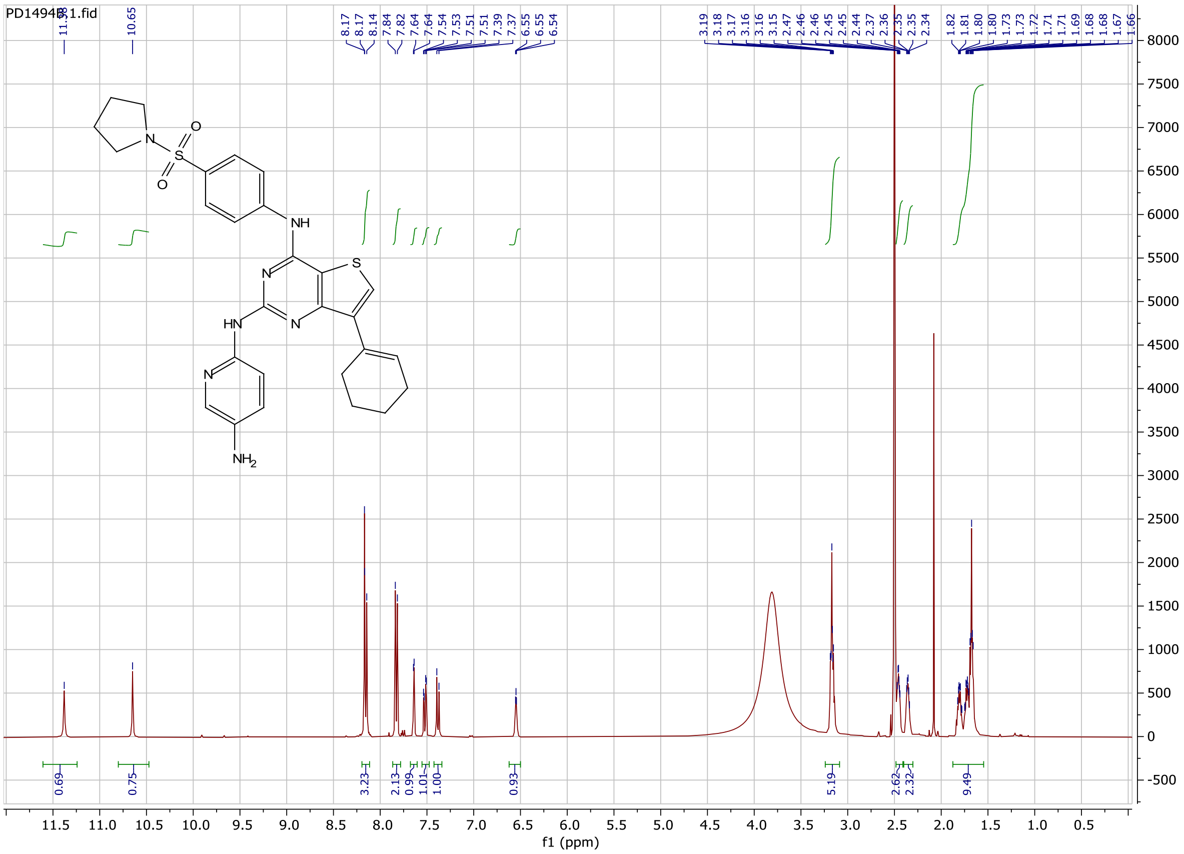

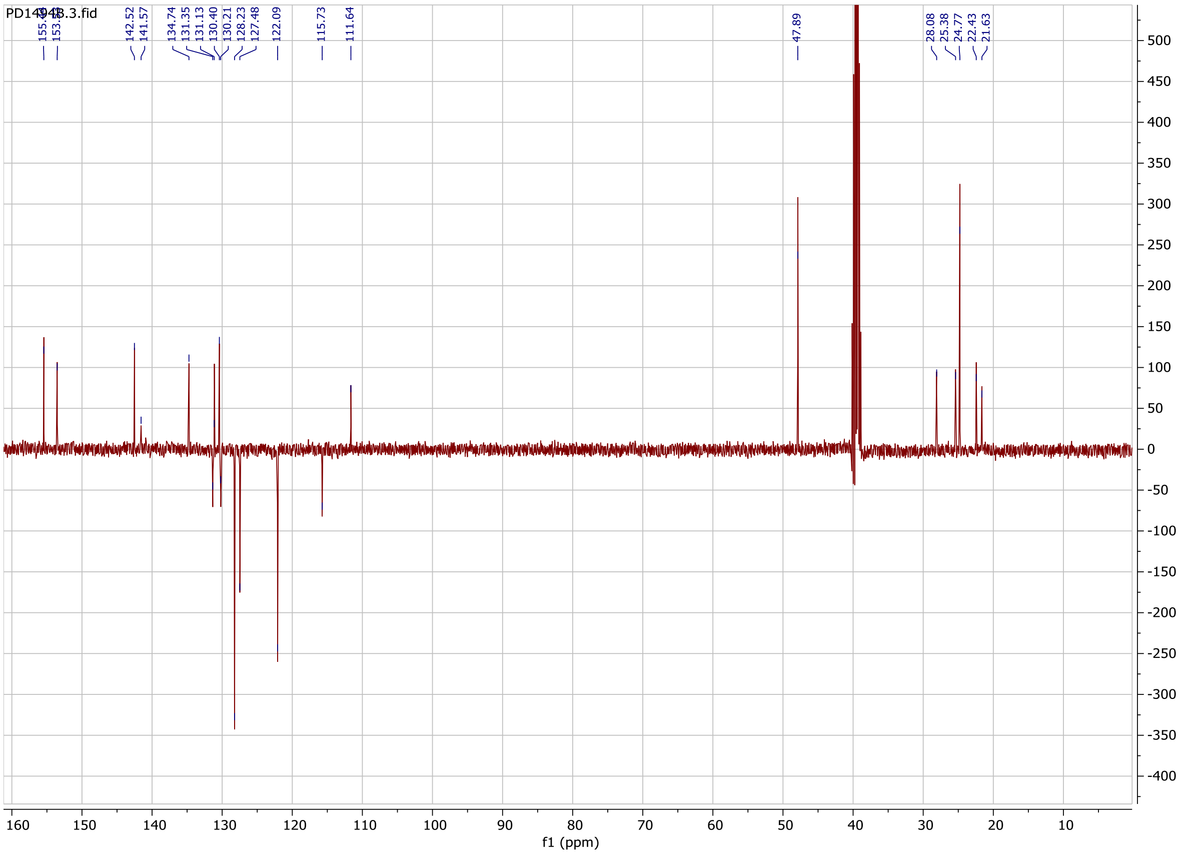

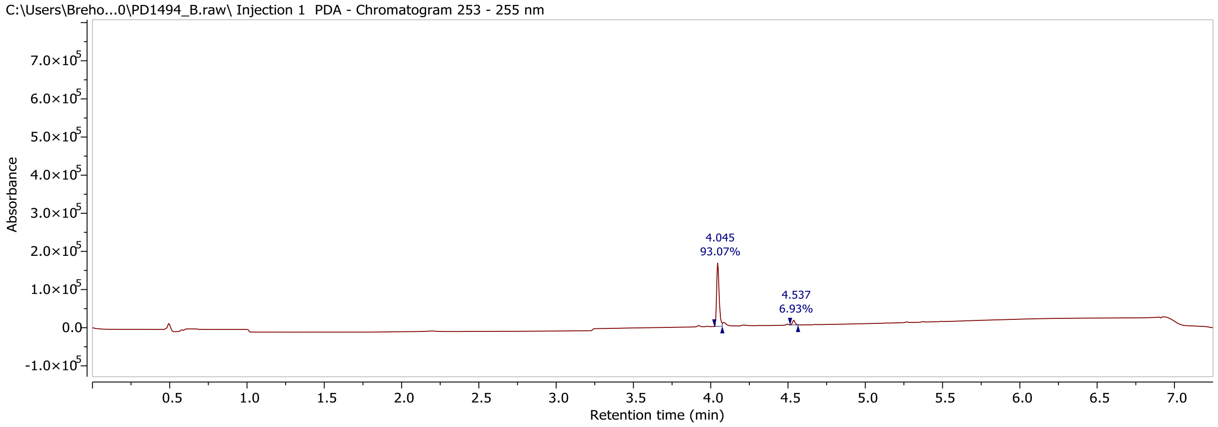

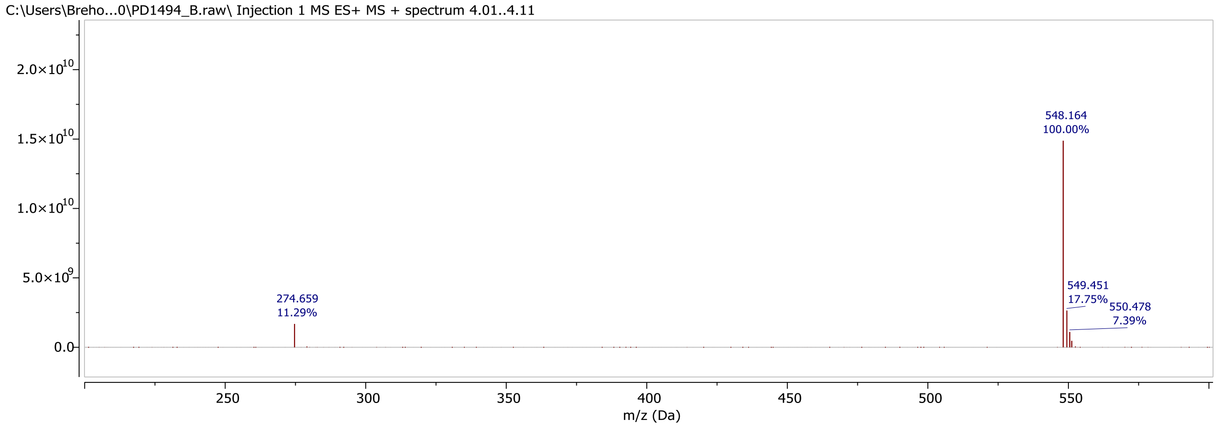

N^5-(5-aminopyridin-2-yl)-3-(cyclohex-1-en-1-yl)-N^7-(4-(1-pyrrolidinylsulfonyl)phenyl)pyrazolo[1,5-*a*]pyrimidine-5,7-diamine (10a)

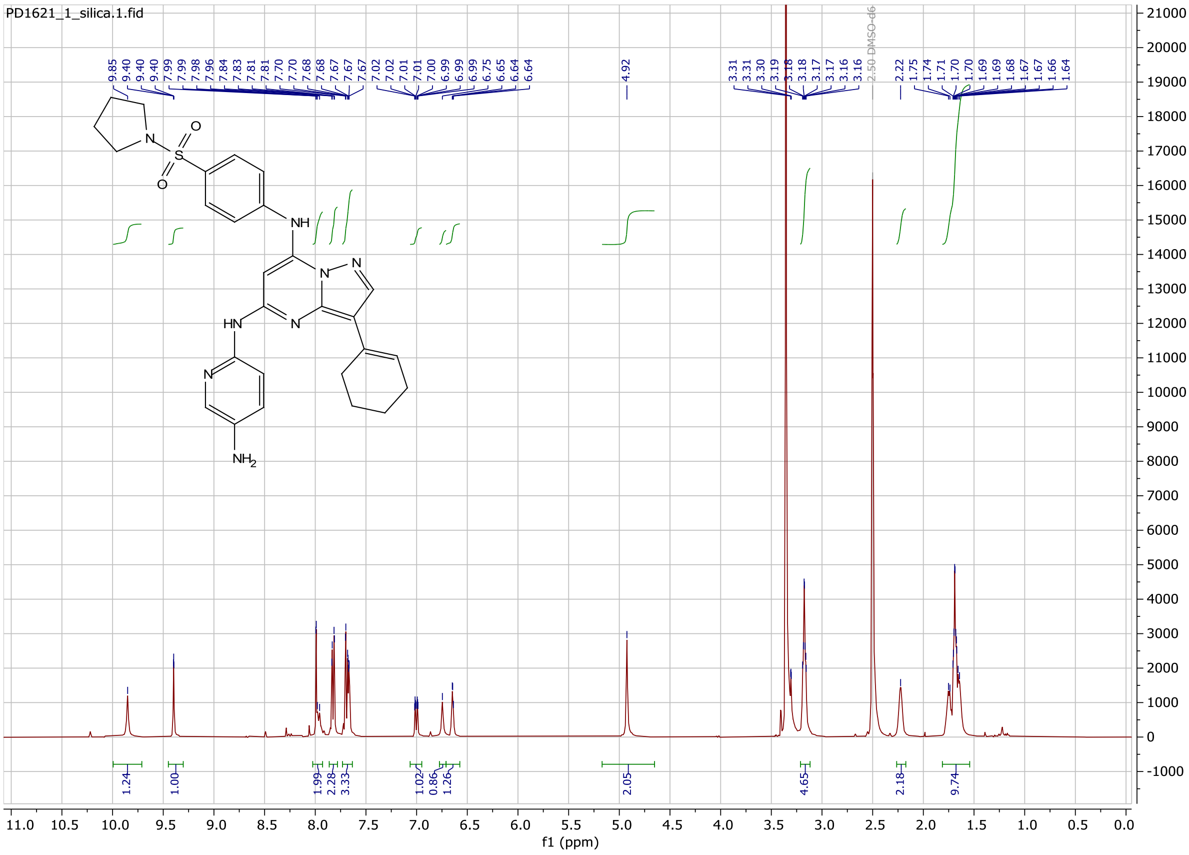

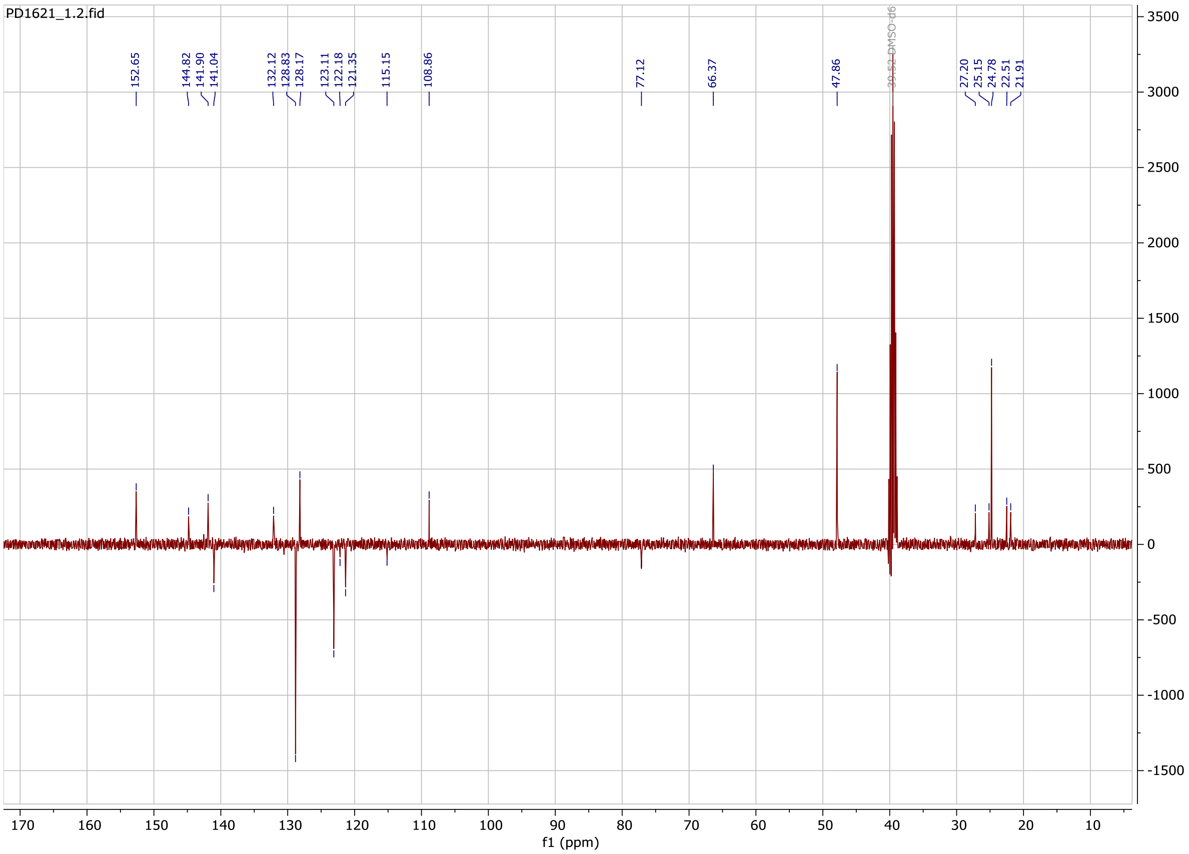
PD1621_1.2.fid
152.65
144.82
141.90
141.04
132.12
128.83
128.17
123.11
122.18
121.35
115.15
108.86
77.12
66.37
47.86
39.52 DMSO-d6
27.20
25.15
24.78
22.51
21.91
f1 (ppm)

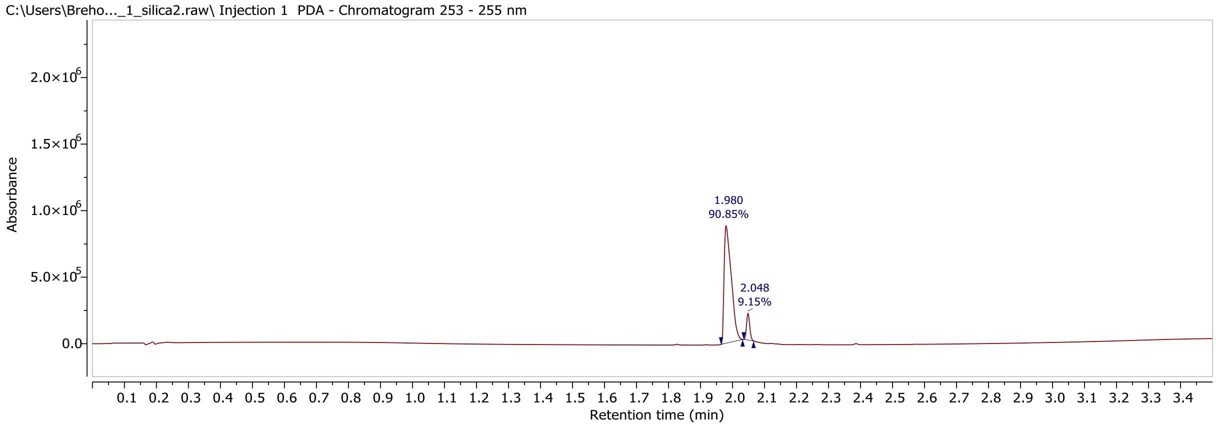
C:\Users\Breho..._1_silica2.raw\ Injection 1 PDA - Chromatogram 253 - 255 nm
Absorbance
1.980
90.85%
2.048
9.15%
Retention time (min)

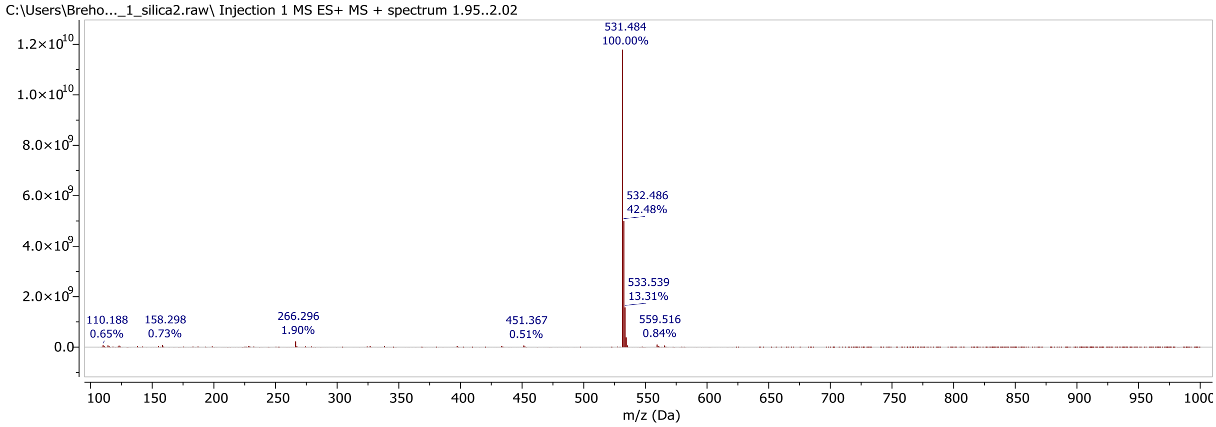
C:\Users\Breho..._1_silica2.raw\ Injection 1 MS ES+ MS + spectrum 1.95..2.02
531.484
100.00%
532.486
42.48%
533.539
13.31%
110.188
0.65%
158.298
0.73%
266.296
1.90%
451.367
0.51%
559.516
0.84%
m/z (Da)

N^5-(6-aminopyridin-3-yl)-3-(cyclohex-1-en-1-yl)-N^7-(4-(1-pyrrolidinylsulfonyl)phenyl)pyrazolo[1,5-*a*]pyrimidine-5,7-diamine (10b)

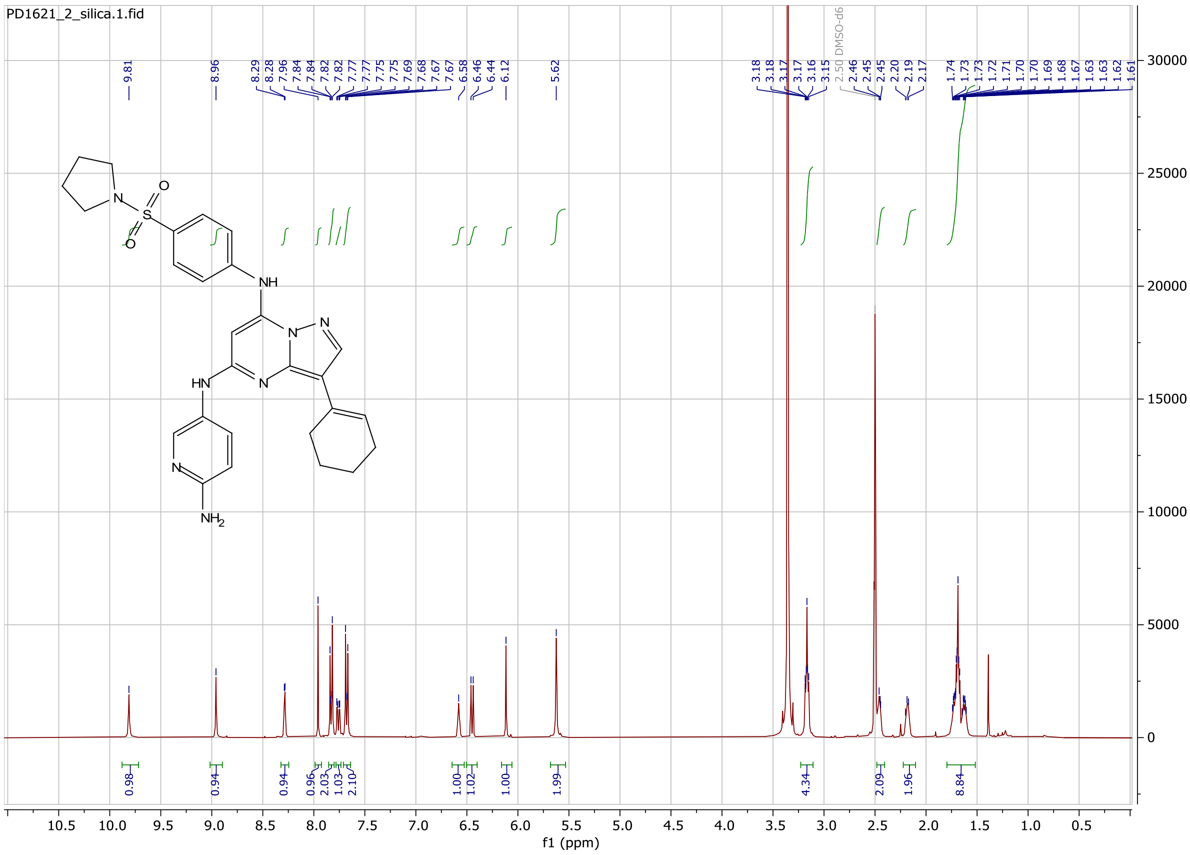

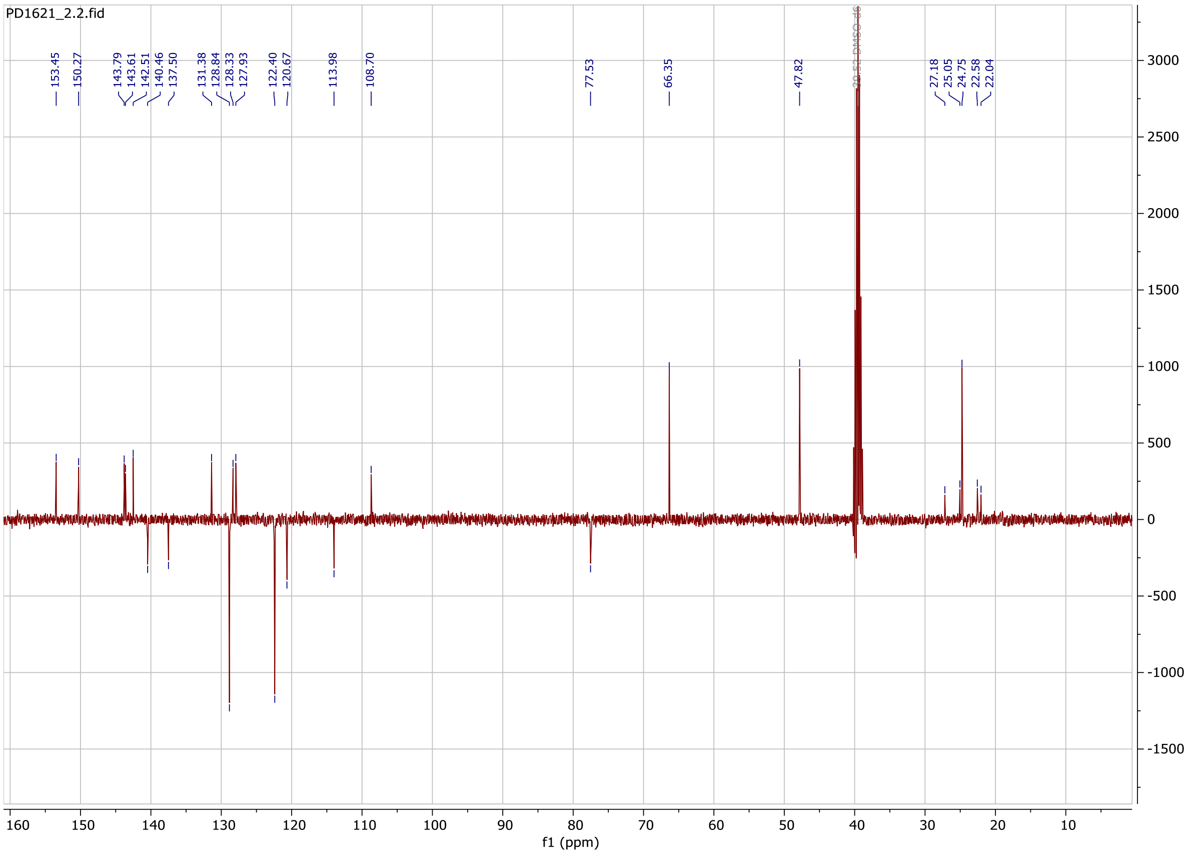

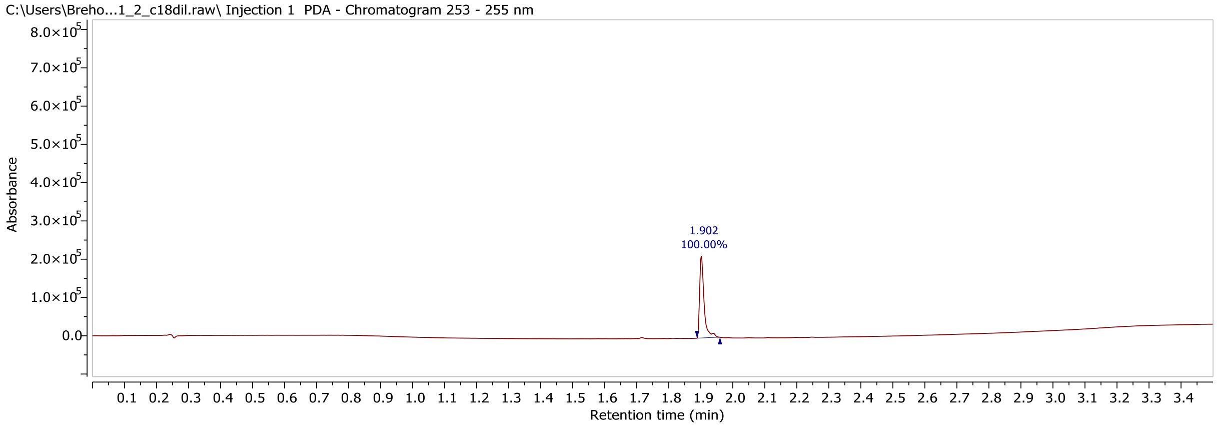

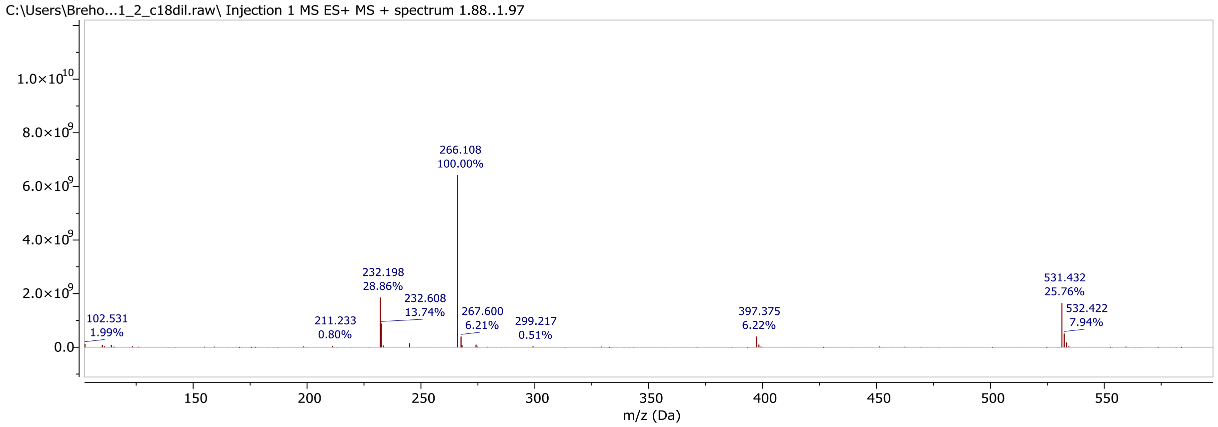

N^5-((1r,4r)-4-aminocyclohexyl)-N^7-(4-(1-pyrrolidinylsulfonyl)phenyl)pyrazolo[1,5-*a*]pyrimidine-5,7-diamine (14)

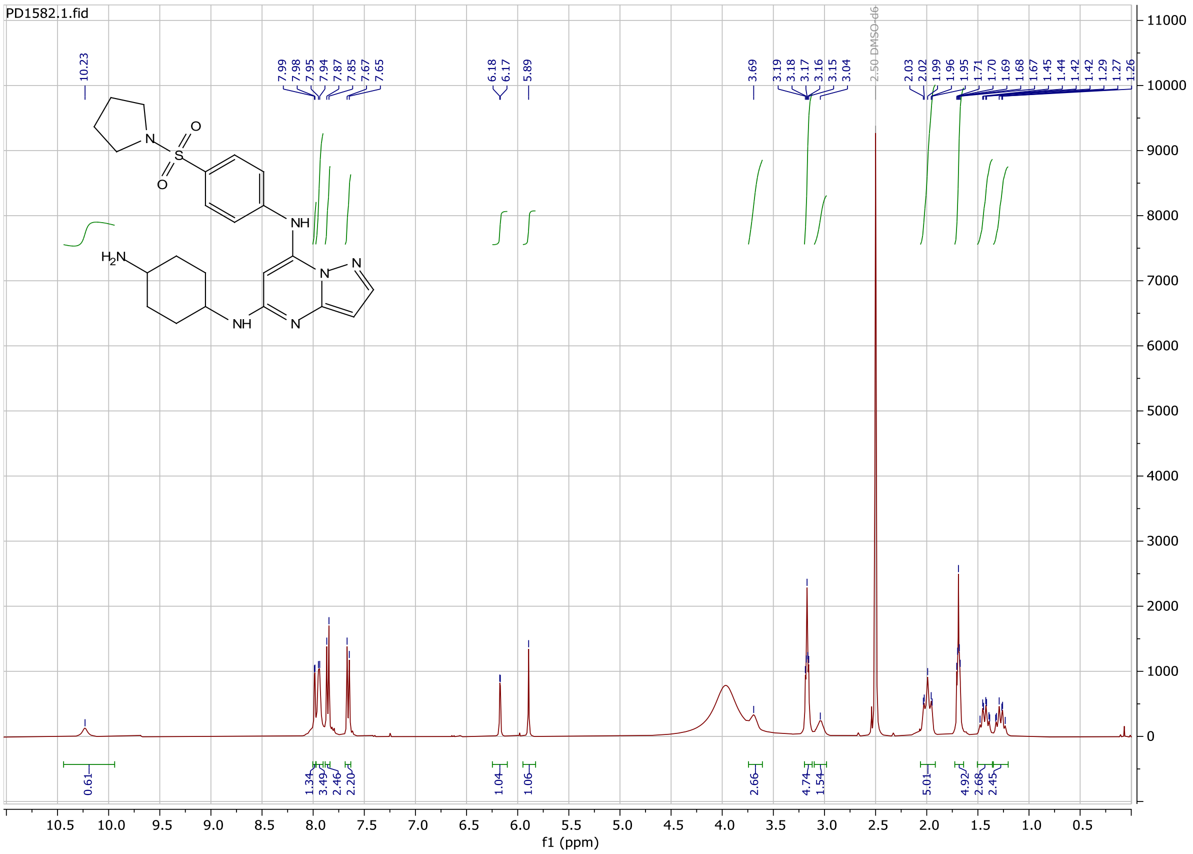

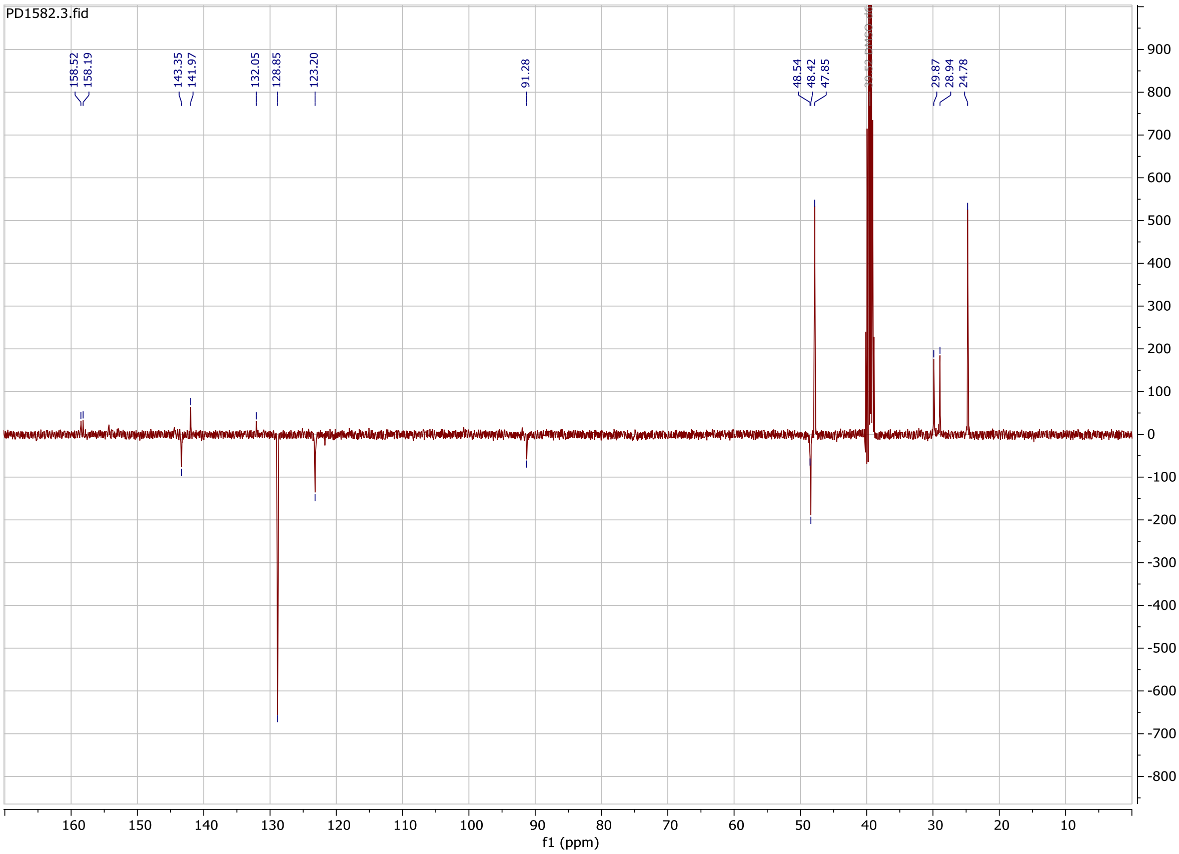

C:\Users\Breho...PD1582_dil.raw\ Injection 1 PDA - Chromatogram 253 - 255 nm

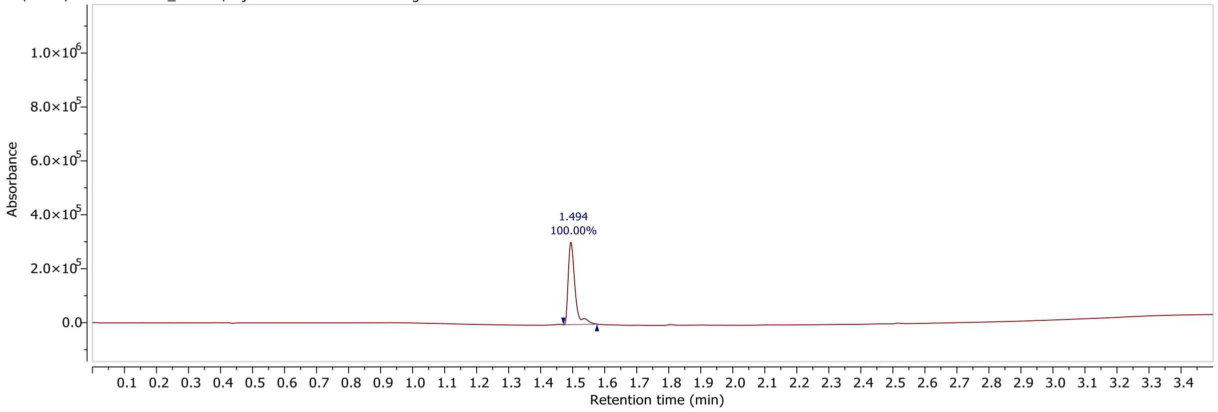

C:\Users\Breho...PD1582_dil.raw\ Injection 1 MS ES+ MS + spectrum 1.49..1.62

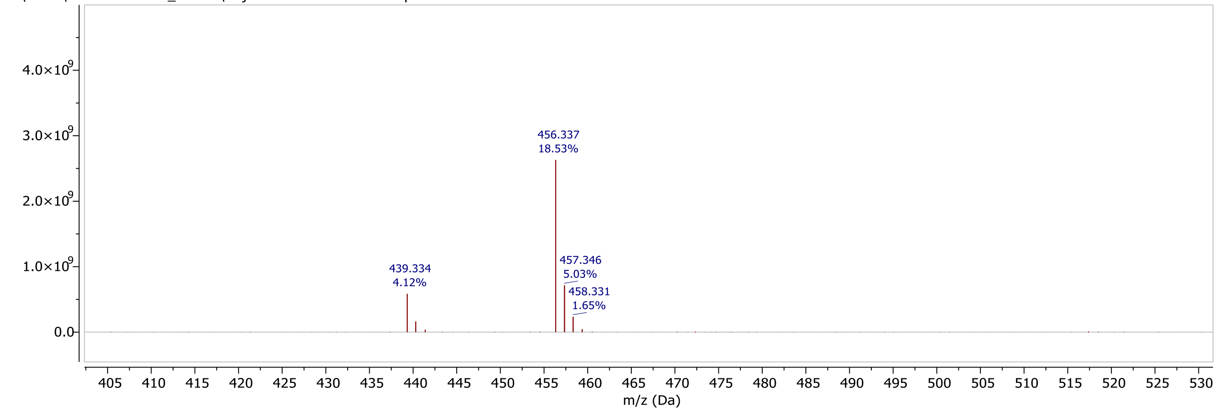

N[5]-(5-aminopyridin-2-yl)-3-cyclohexyl-*N*[7]-(4-(pyrrolidin-1-ylsulfonyl)phenyl)-3*H*-imidazo[4,5-*b*]pyridine-5,7-diamine (18a)

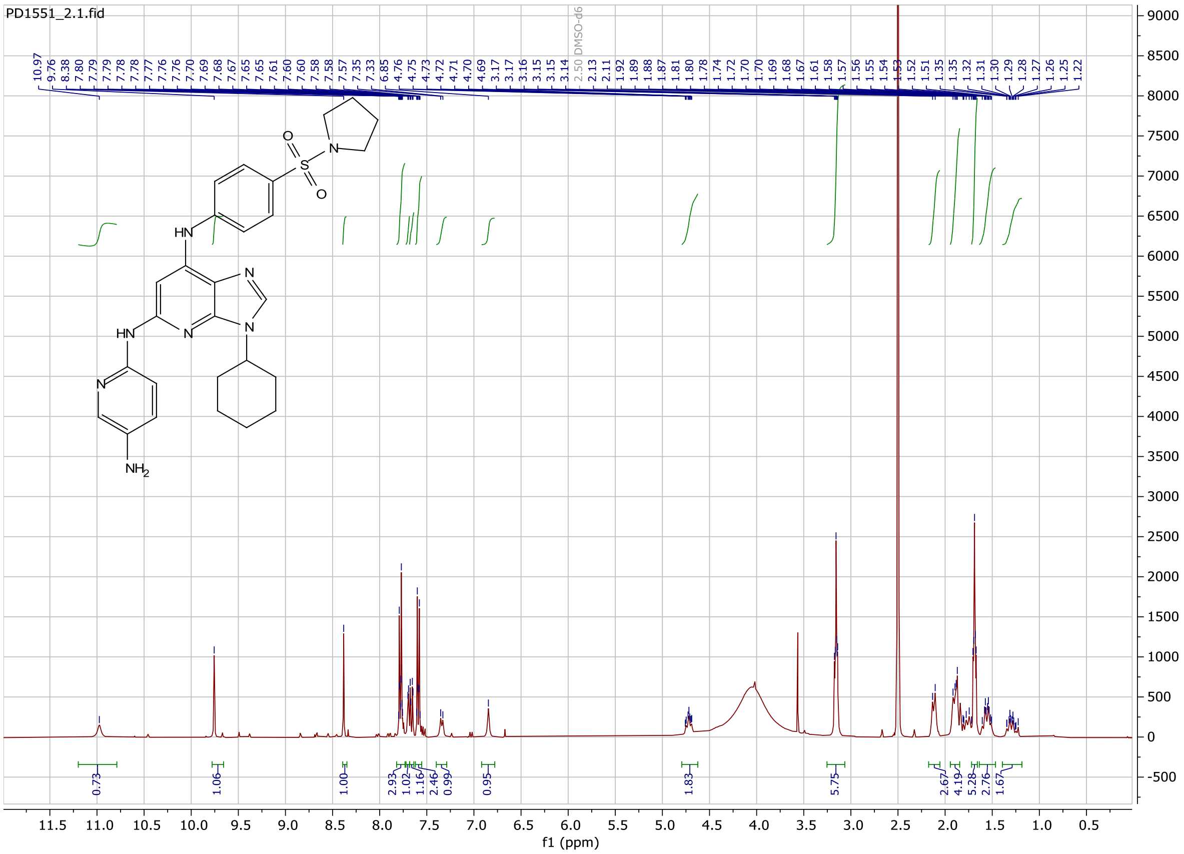

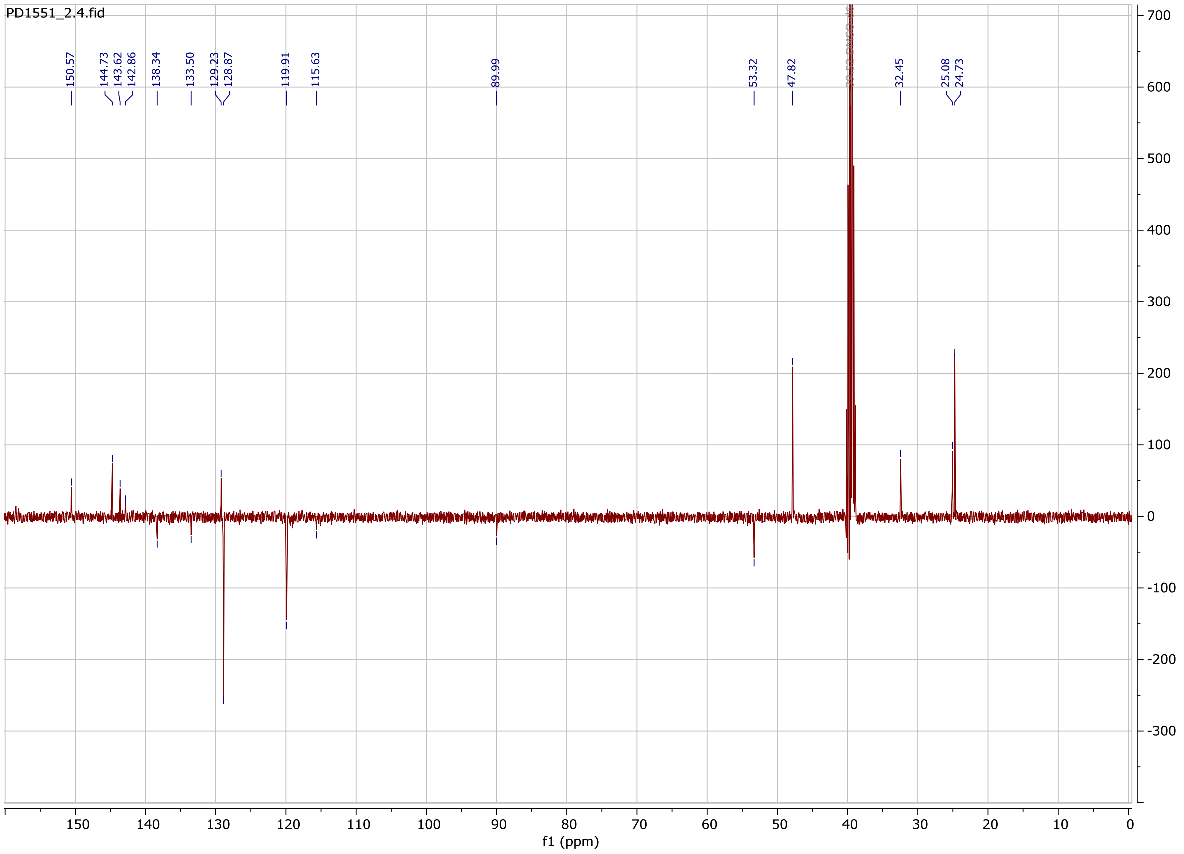

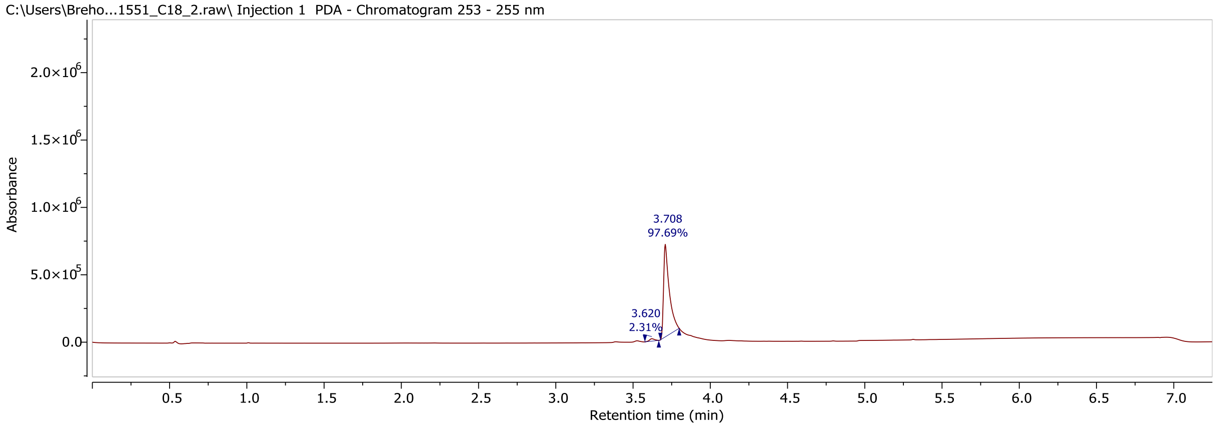

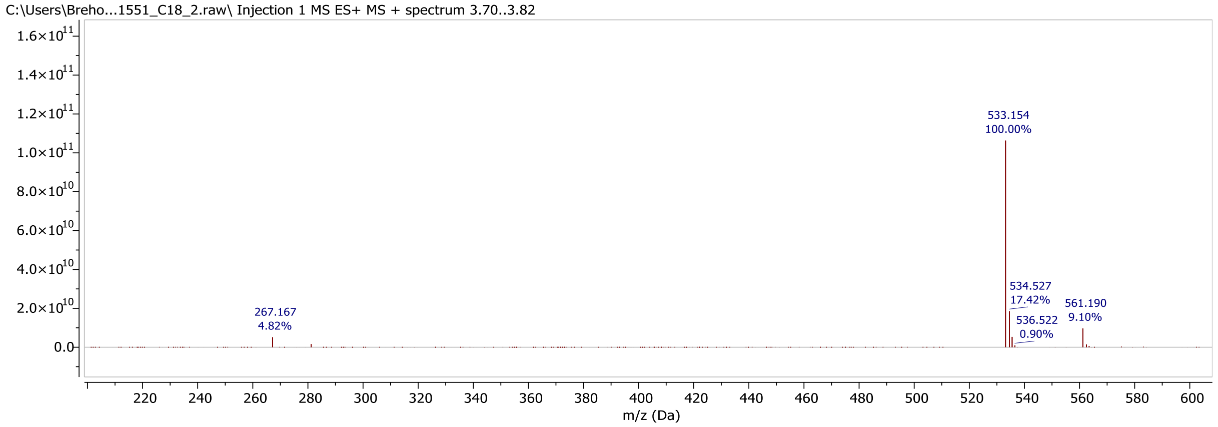

N^5-(6-aminopyridin-3-yl)-3-cyclohexyl-N^7-(4-(pyrrolidin-1-ylsulfonyl)phenyl)-3*H*-imidazo[4,5-*b*]pyridine-5,7-diamine (18b)

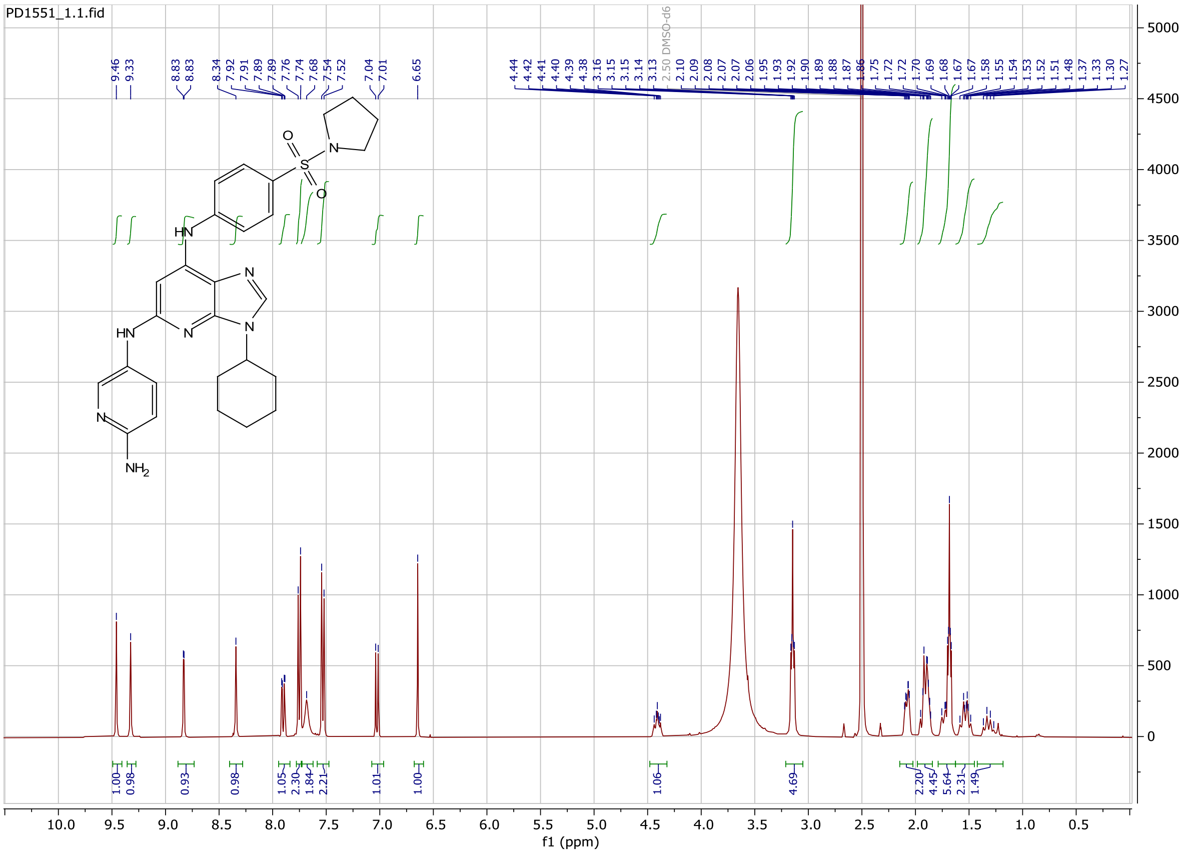

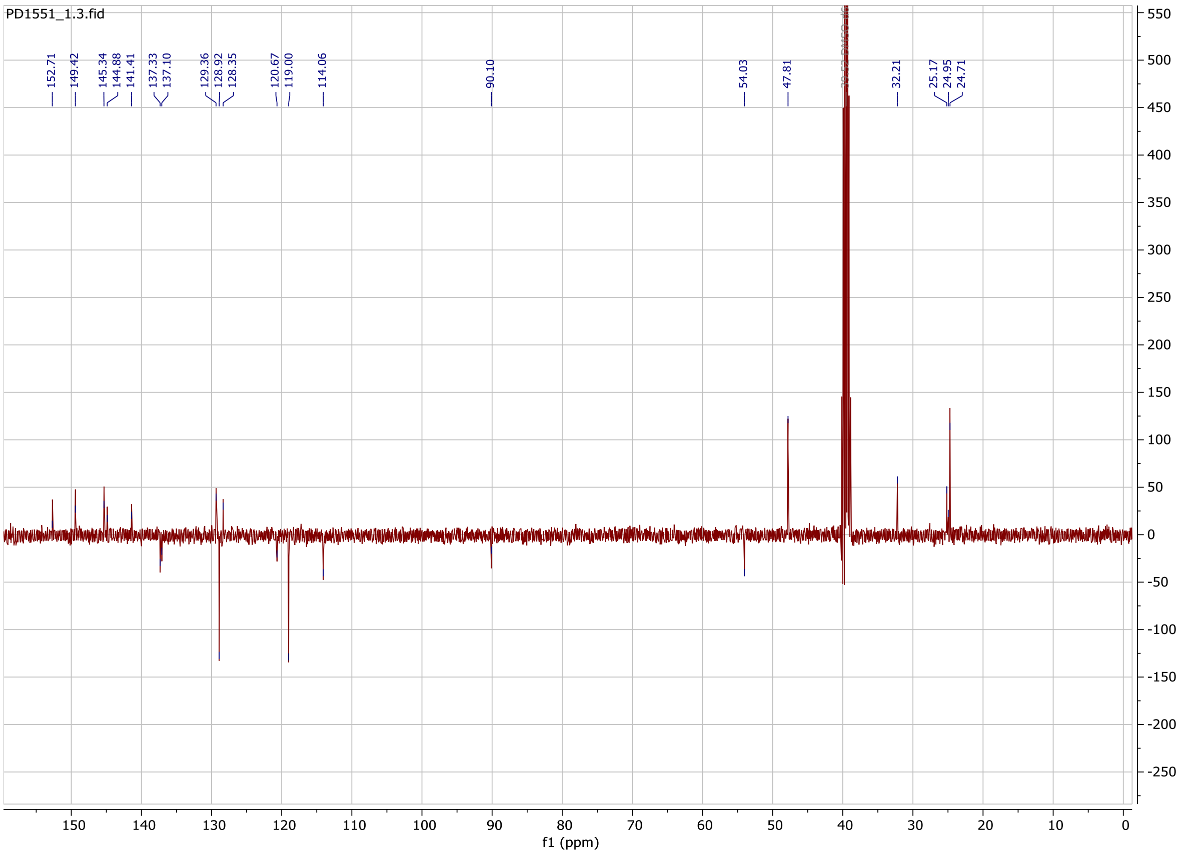

C:\Users\Breho...1551_C18_1.raw\ Injection 1 PDA - Chromatogram 253 - 255 nm

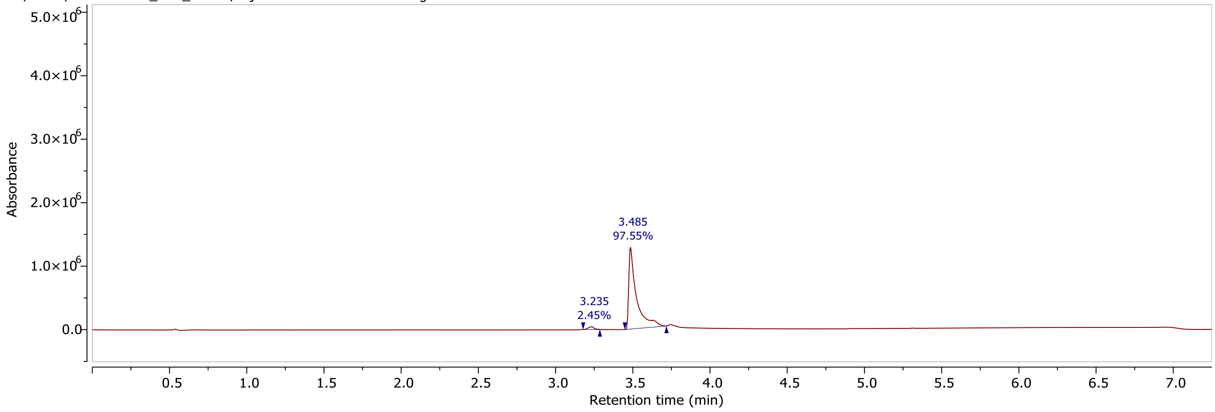

C:\Users\Breho...1551_C18_1.raw\ Injection 1 MS ES+ MS + spectrum 3.45..3.72

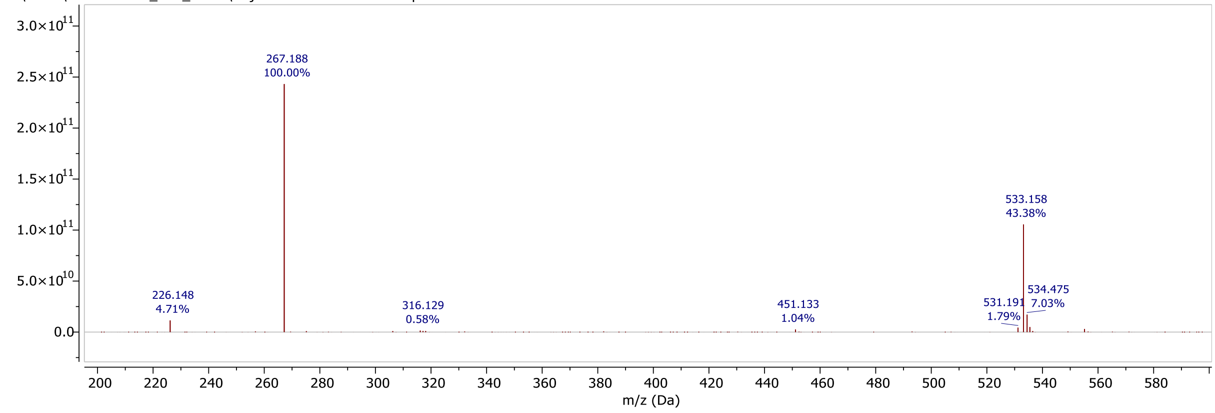

8-(1-Cyclohexen-1-yl)-N^2-(4-aminophenyl)-N^4-(4-(1-pyrrolidinylsulfonyl)phenyl)pyrido[4,3-*d*]pyrimidine-2,4-diamine (25)

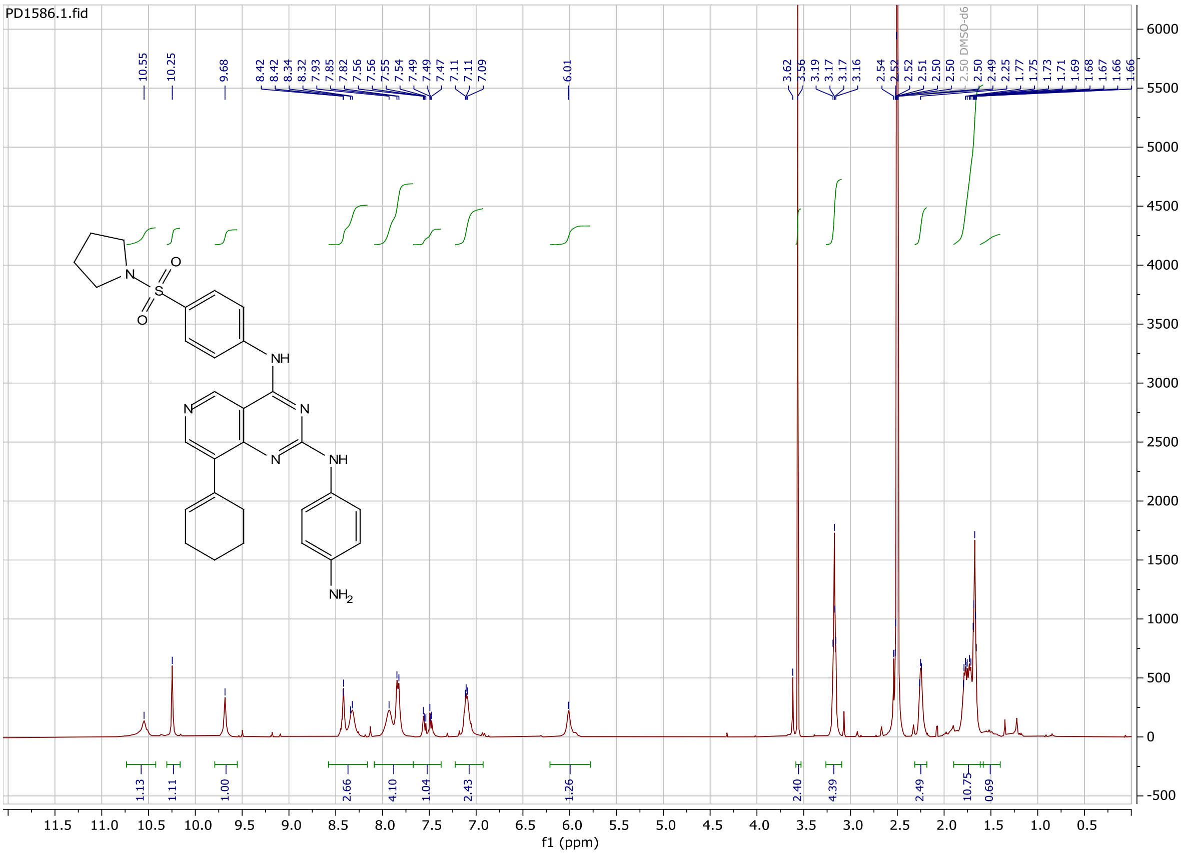

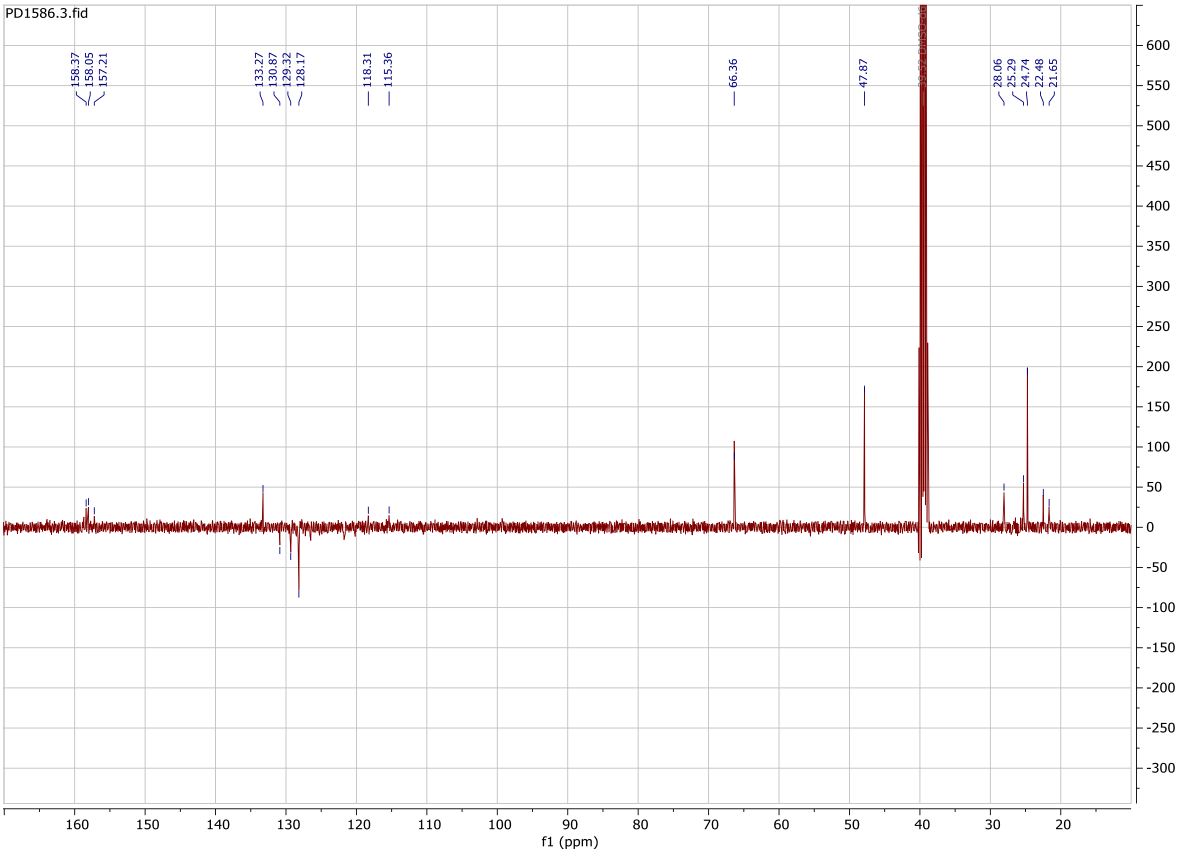

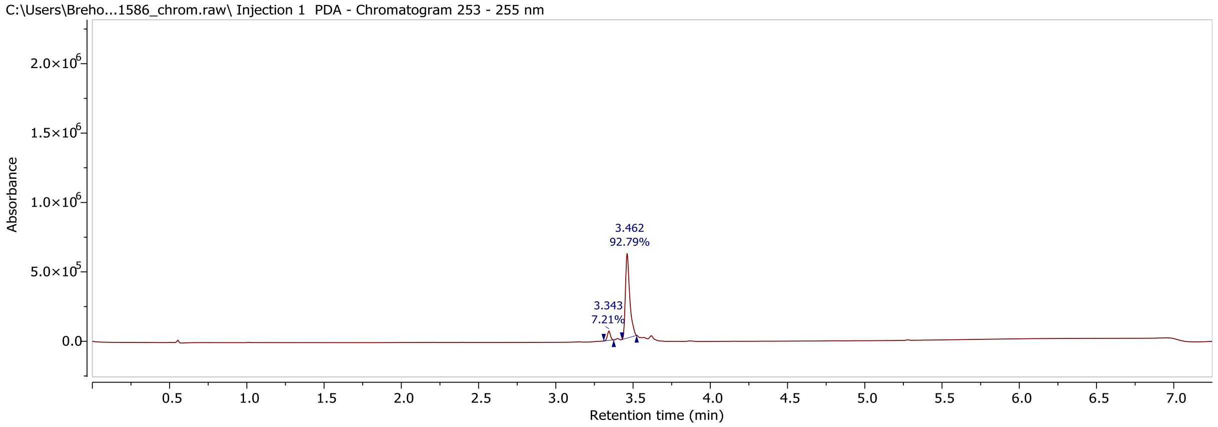

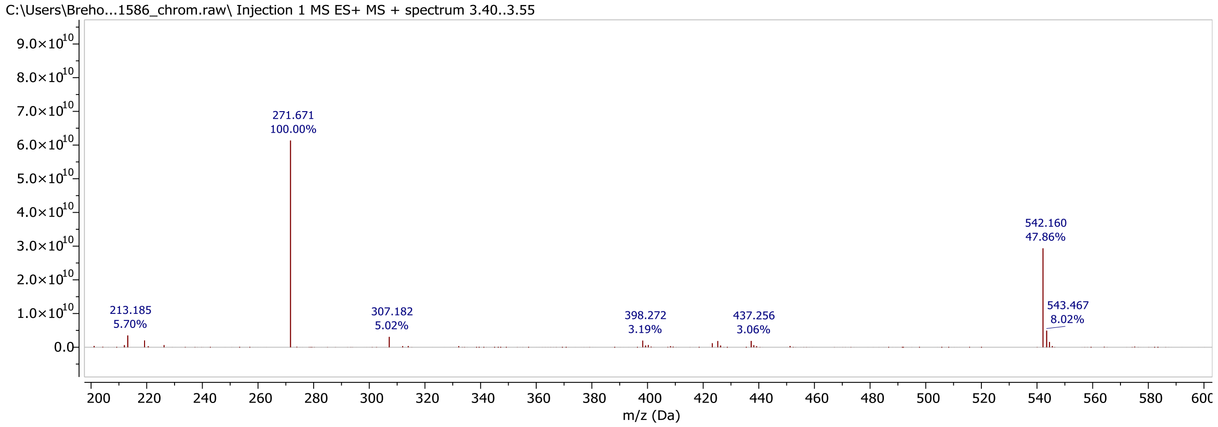

N^6-((1r,4r)-4-aminocyclohexyl)-3-bromo-N^8-(4-(1-pyrrolidinylsulfonyl)phenyl)imidazo[1,2-*b*]pyridazine-6,8-diamine (29a)

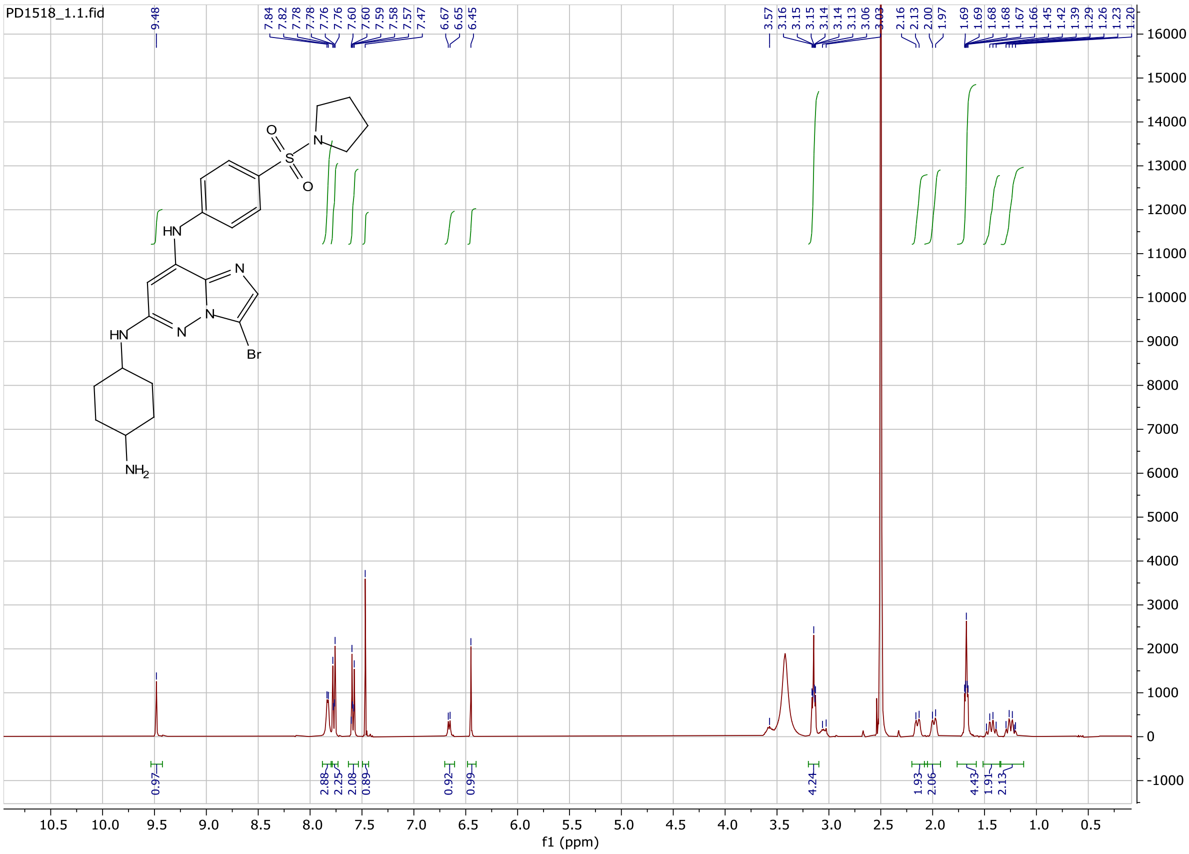

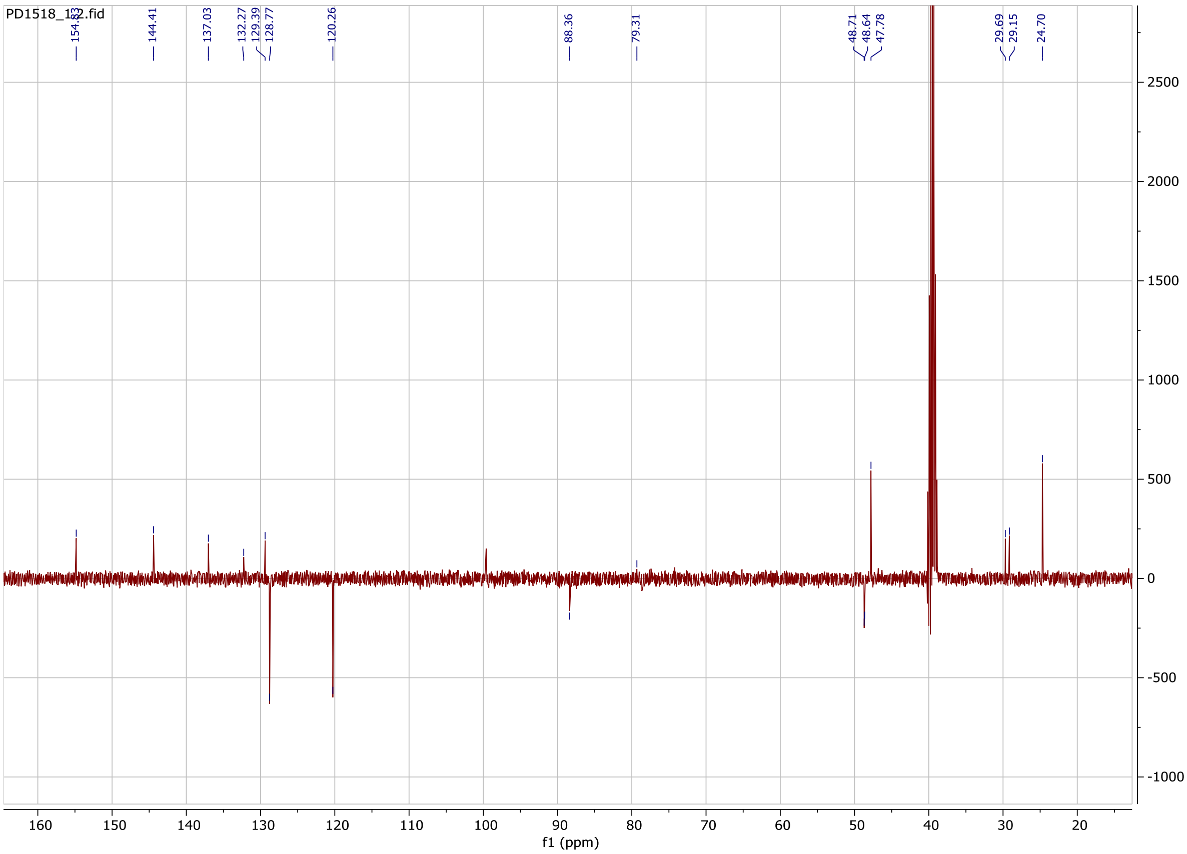
PD1518_1.2.fid
154.83
144.41
137.03
132.27
129.39
128.77
120.26
88.36
79.31
48.71
48.64
47.78
29.69
29.15
24.70
f1 (ppm)

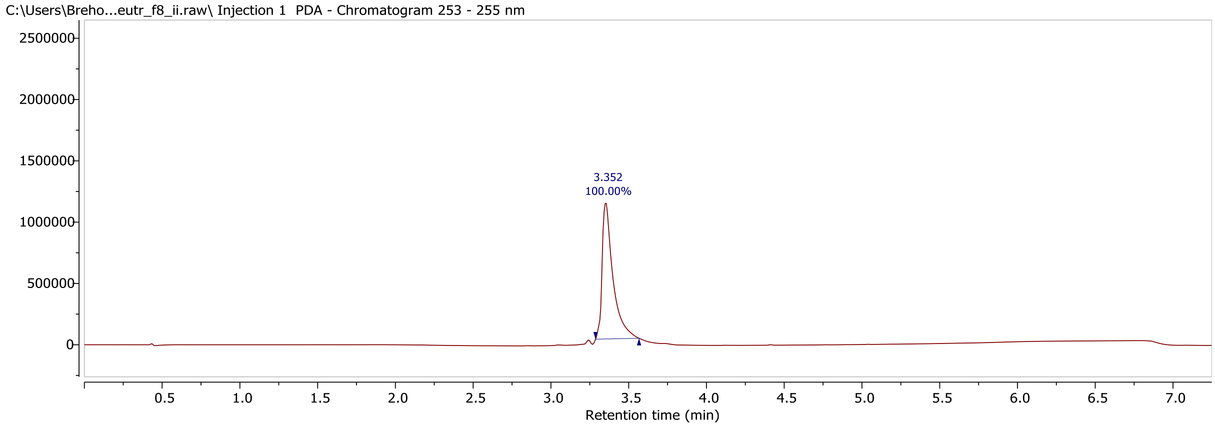
C:\Users\Breho...eutr_f8_ii.raw\ Injection 1 PDA - Chromatogram 253 - 255 nm
3.352
100.00%
Retention time (min)

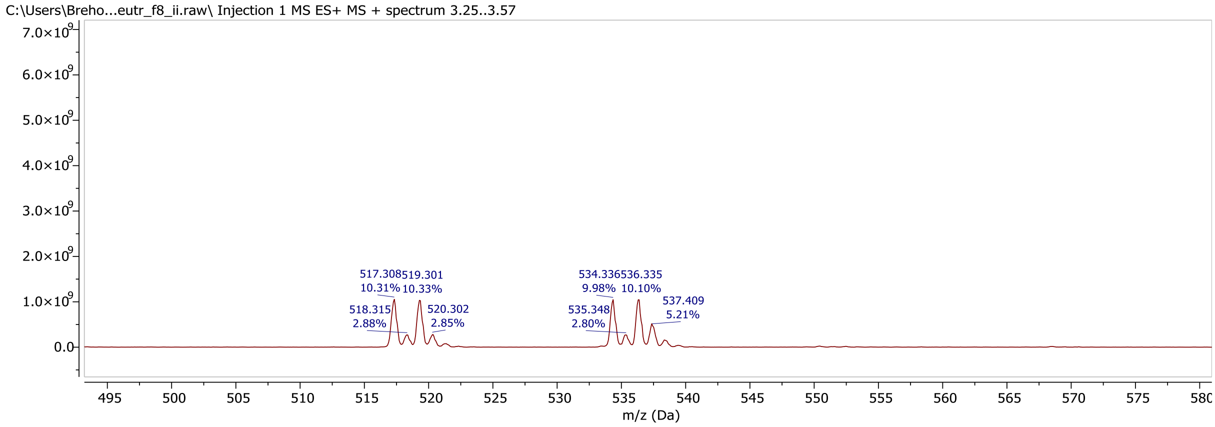
C:\Users\Breho...eutr_f8_ii.raw\ Injection 1 MS ES+ MS + spectrum 3.25..3.57
517.308
10.31%
519.301
10.33%
518.315
2.88%
520.302
2.85%
534.336
9.98%
536.335
10.10%
535.348
2.80%
537.409
5.21%
m/z (Da)

***N*[6]-((1r,4r)-4-aminocyclohexyl)-3-(1-cyclohexene-1-yl)-*N*[8]-(4-(1-pyrrolidinylsulfonyl)phenyl)imidazo[1,2-*b*]pyridazine-6,8-diamine (29b)**

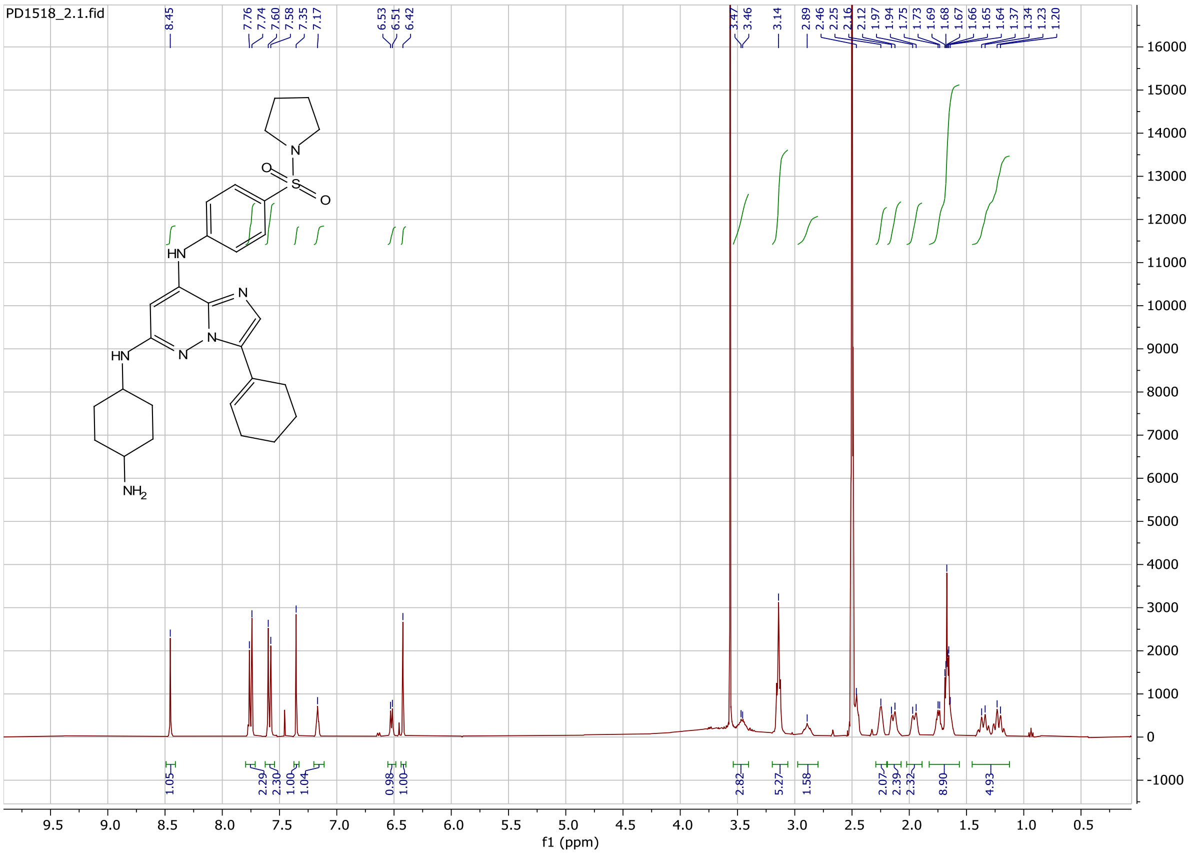

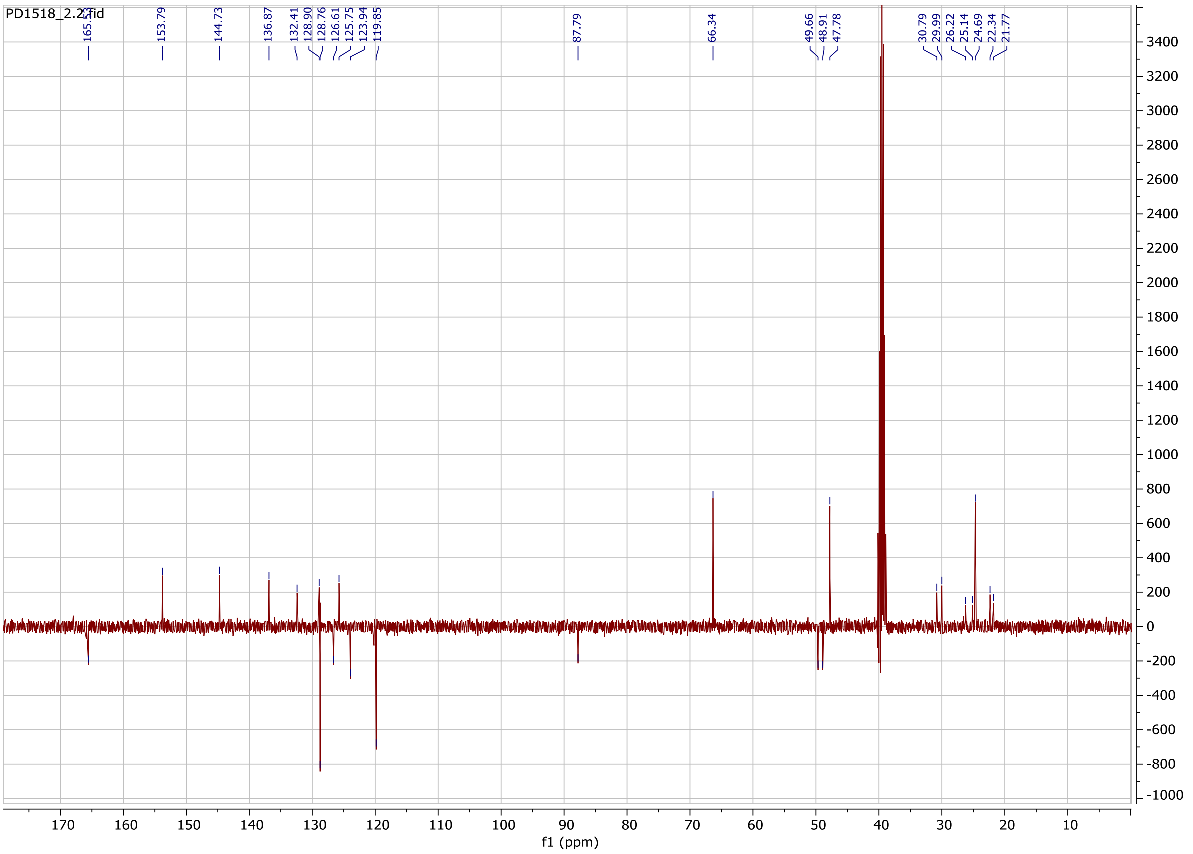

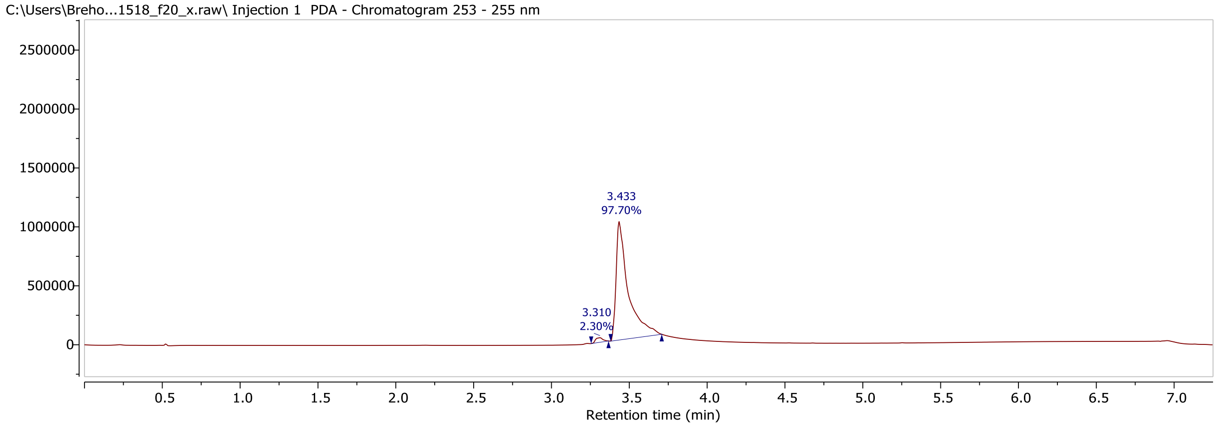

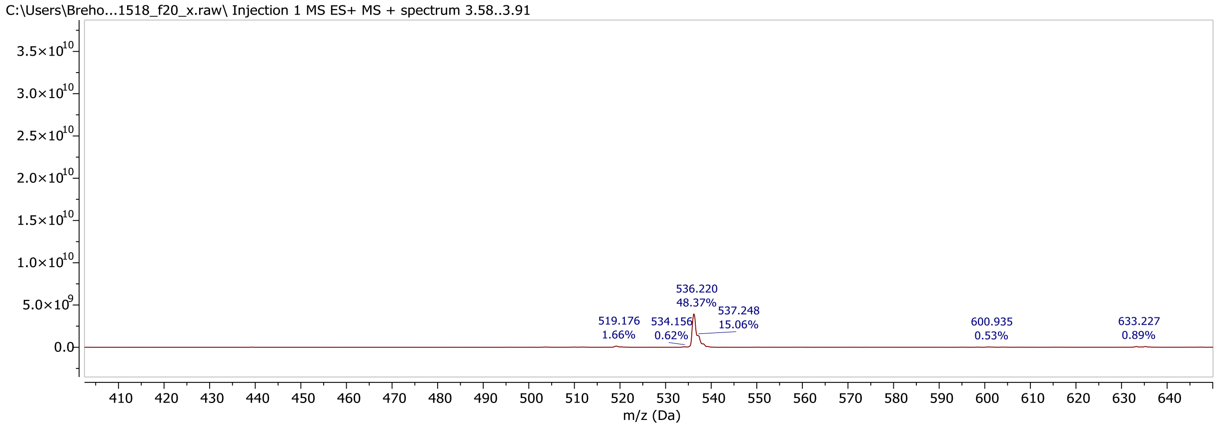

***N*^{6}-((1r,4r)-4-aminocyclohexyl)-3-(cyclohexan-1-yl)-*N*^{8}-(4-(1-pyrrolidinylsulfonyl)phenyl)imidazo[1,2-*b*]pyridazine-6,8-diamine (30)**

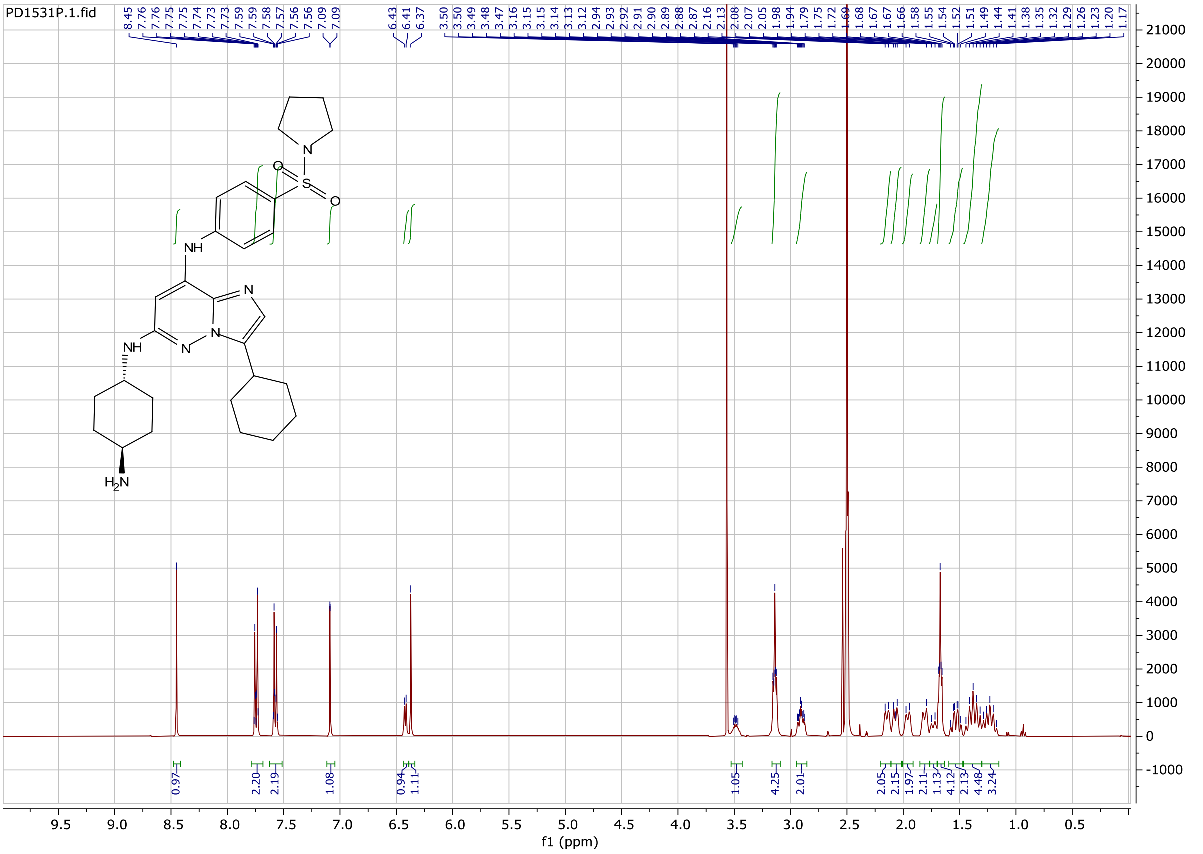

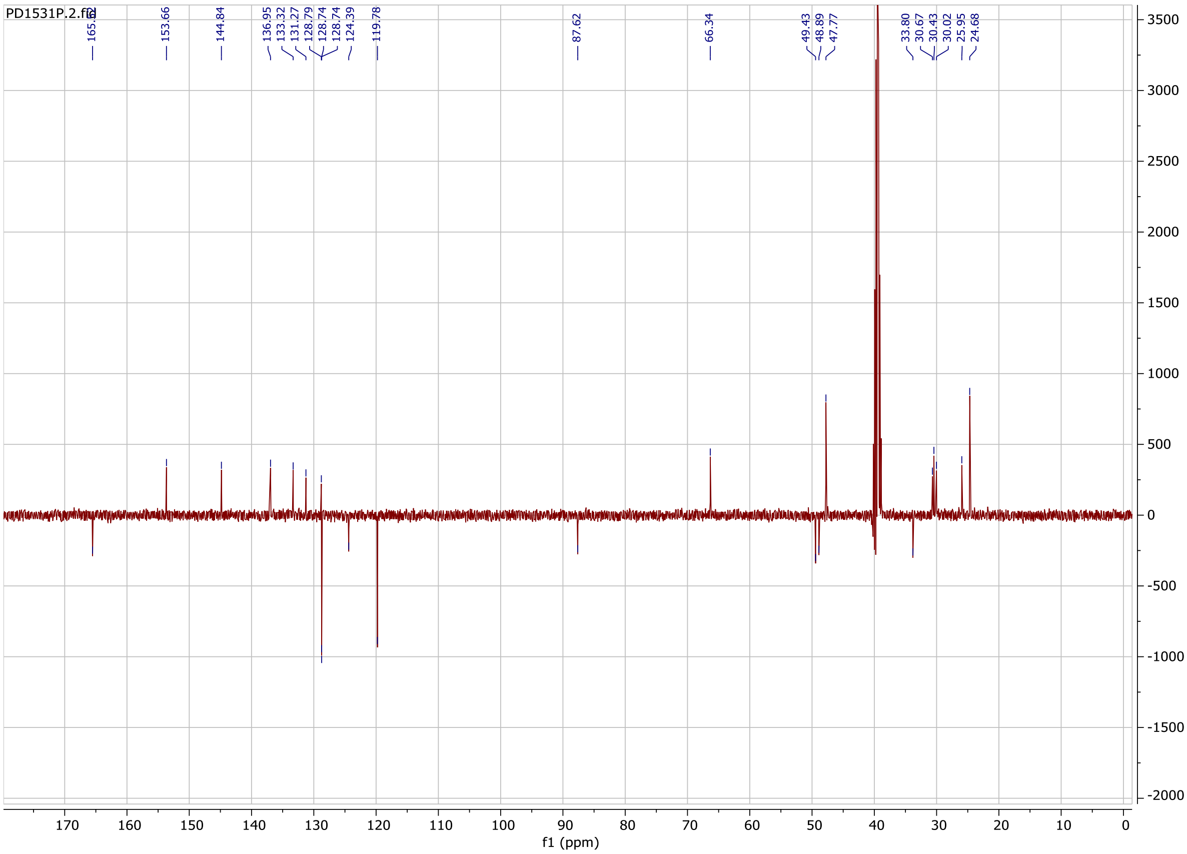
PD1531P.2.fid
165.0
153.66
144.84
136.95
133.32
131.27
128.79
128.74
128.74
124.39
119.78
87.62
66.34
49.43
48.89
47.77
33.80
30.67
30.43
30.02
25.95
24.68
f1 (ppm)

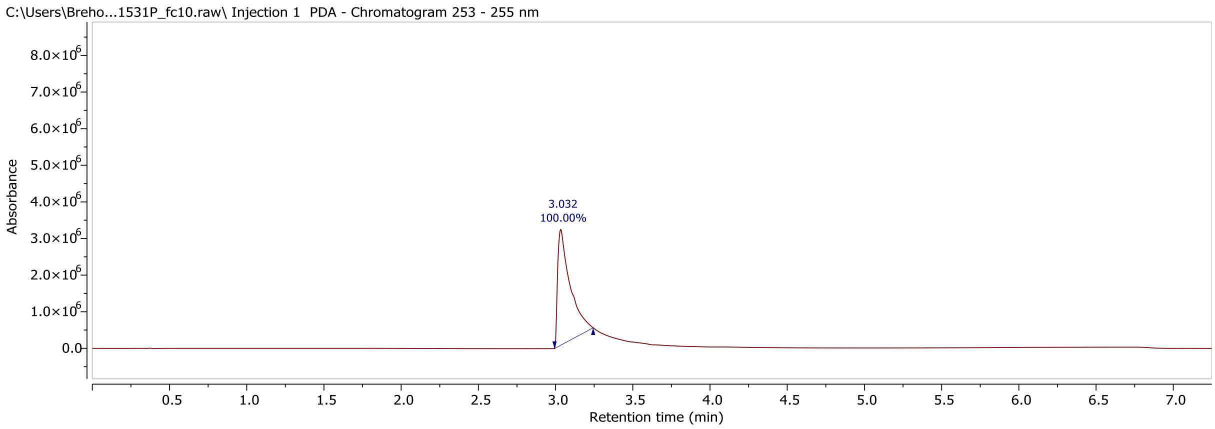
C:\Users\Breho...1531P_fc10.raw\ Injection 1 PDA - Chromatogram 253 - 255 nm
Absorbance
3.032
100.00%
Retention time (min)

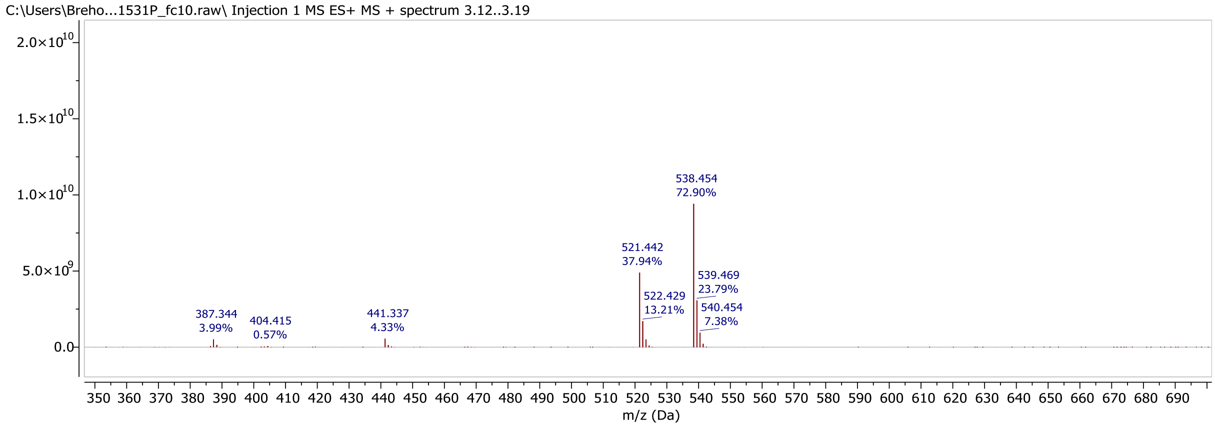
C:\Users\Breho...1531P_fc10.raw\ Injection 1 MS ES+ MS + spectrum 3.12..3.19
387.344
3.99%
404.415
0.57%
441.337
4.33%
521.442
37.94%
522.429
13.21%
538.454
72.90%
539.469
23.79%
540.454
7.38%
m/z (Da)
